# Tunicamycin Potentiates Antifungal Drug Tolerance via Aneuploidy in Candida albicans

**DOI:** 10.1128/mBio.02272-21

**Published:** 2021-08-31

**Authors:** Feng Yang, Vladimir Gritsenko, Yaniv Slor Futterman, Lu Gao, Cheng Zhen, Hui Lu, Yuan-ying Jiang, Judith Berman

**Affiliations:** a Department of Pharmacy, Shanghai Tenth People's Hospital, School of Medicine, Tongji University, Shanghai, China; b Shmunis School of Biomedical and Cancer Research, The George S. Wise Faculty of Life Sciences, Tel Aviv University, Tel Aviv, Israel; University of British Columbia

**Keywords:** antifungal tolerance, cross-adaptation, stress response, trisomy

## Abstract

How cells exposed to one stress are later able to better survive other types of stress is not well understood. In eukaryotic organisms, physiological and pathological stresses can disturb endoplasmic reticulum (ER) function, resulting in “ER stress.” Here, we found that exposure to tunicamycin, an inducer of ER stress, resulted in the acquisition of a specific aneuploidy, chromosome 2 trisomy (Chr2x3), in Candida albicans. Importantly, the resulting aneuploidy also conferred cross-tolerance to caspofungin, a first-line echinocandin antifungal, as well as to hydroxyurea, a common chemotherapeutic agent. Exposure to a range of tunicamycin concentrations induced similar ER stress responses. Extra copies of one Chr2 gene, *MKK2*, affected both tunicamycin and caspofungin tolerance, while at least 3 genes on chromosome 2 (*ALG7*, *RTA2*, and *RTA3*) affected only tunicamycin and not caspofungin responses. Other Chr2 genes (*RNR1* and *RNR21*) affected hydroxyurea tolerance but neither tunicamycin nor caspofungin tolerance. Deletion of components of the protein kinase C (PKC) or calcineurin pathways affected tolerance to both tunicamycin and caspofungin, supporting the idea that the ER stress response and echinocandin tolerance are regulated by overlapping stress response pathways. Thus, antifungal drug tolerance can arise rapidly via ER stress-induced aneuploidy.

## INTRODUCTION

Candida albicans is an opportunistic human fungal pathogen. It is a commensal colonizer of the gastrointestinal tract of approximately 45% to 60% of healthy adults (1); 30% to 50% of people carry C. albicans in the oral cavity (2). C. albicans is also the most common human fungal pathogen and is responsible for about 70% of worldwide fungal infections, causing diseases ranging from mucosal to life-threatening systemic infections (3). Despite consistently low levels of antifungal resistance reported for this organism (0.1% to 0.4%) (4), patient treatment failures are quite high (>30%). Isolates exhibiting antifungal resistance grow well at drug concentrations above the MIC for that drug and species, as measured after 24 h of drug exposure (5). Antifungal tolerance is a feature of susceptible isolates (those with standard MIC values for the species) and is distinct from resistance. Despite being susceptible in standard MIC assays, a subpopulation of cells are able to grow, albeit slowly, in the presence of supra-MIC drug concentrations (5, 6). Because of this slow growth, tolerance is generally measured after longer times of drug exposure (e.g., 48 h) as the fraction of growth (FoG) within the zone of inhibition (7). High tolerance levels may contribute to the treatment failures of C. albicans infections (6, 8).

Because C. albicans is a eukaryotic pathogen, the antifungal arsenal is very limited. Currently, only three major classes of antifungal agents are used clinically, as follows: azoles, polyenes, and echinocandins (9). Echinocandins, the newest class of established antifungals, inhibit β-(1, 3)-glucan synthesis in the fungal cell wall, causing cell death, although they are only fungistatic in some pathogenic species, such as Aspergillus fumigatus. Echinocandins have become the first-line antifungal drugs for the treatment of systemic candidiasis (10).

Aneuploidy has been associated with drug resistance and tolerance in human-pathogenic fungi. In C. albicans, aneuploidy enables adaptation to fluconazole (11–14) and caspofungin (15–17). In Cryptococcus neoformans, aneuploidy enables adaptation to fluconazole (18, 19) and flucytosine (20). In Candida glabrata, formation of a new minichromosome is associated with fluconazole resistance (21), and in Candida auris, an emerging outbreak pathogen that can acquire resistance to all three major classes of antifungal drugs, duplication of chromosome V is associated with resistance to fluconazole (22).

Importantly, aneuploidy also causes cross-adaptation to unrelated drugs. For example, growth of C. albicans on l-sorbose as the sole carbon sources selects for chromosome 5 monosomy, an aneuploidy that also drives cross-tolerance to caspofungin (CSP) and flucytosine (15). Similarly, exposure to hydroxyurea (HU), a chemotherapeutic drug, selects for chromosome 2 trisomy mutants, which enables cross-tolerance to CSP (17). Therefore, antifungal tolerance to some drugs can arise without any pre-exposure to that drug.

Tunicamycin (TUN), a product of Streptomyces clavuligerus and Streptomyces lysosuperficus, is commonly used to induce endoplasmic reticulum (ER) stress experimentally. It blocks N-linked glycosylation by inhibiting the transfer of UDP-*N*-acetylglucosamine to dolichol phosphate in the ER of eukaryotic cells, thus resulting in misfolded proteins and ER stress (23). Recently, selection with an inhibitory concentration of TUN in Saccharomyces cerevisiae caused aneuploidy of several chromosomes, with recurrent appearance of chromosome 2 disomy, and three genes on chromosome 2 were necessary for the aneuploidy-associated tolerance to TUN (24).

In this study, we asked if aneuploidy is a general mechanism for TUN tolerance in C. albicans. Exposure of lab strain SC5314 to subinhibitory and inhibitory concentrations of TUN for short and long time periods yielded TUN-tolerant colonies/adaptors. A total of 62 tolerant adaptors were sequenced, and most of them had duplication of chromosome 2. Importantly, chromosome 2 trisomy enabled cross tolerance to TUN (ER stress inducer), CSP (first-line fungicidal drug), and HU (chemotherapeutic drug). We identified the following chromosome 2 genes associated with tolerance to the three drugs: *ALG7*, *RTA2*, and *RTA3* were associated with TUN tolerance; *RNR1* and *RNR21* were associated with HU tolerance; and *MKK2* was associated with TUN and CSP tolerance. The calcineurin pathway and the PKC pathway also were associated with both TUN and CSP tolerance. We suggest that aneuploidy arises rapidly under many stress conditions and has the potential to confer multidrug tolerance, largely because of its concerted effect on the copy number of many genes.

## RESULTS

### Obtaining isolates that grow at inhibitory TUN concentrations.

C. albicans lab strain SC5314 is susceptible to TUN, with growth slightly inhibited by 1 μg/ml (*P* > 0.05) and completely inhibited by 4 μg/ml of TUN (*P* < 0.001) in broth microdilution assays (see Fig. S1A in the supplemental material). On agar medium, 1 μg/ml of TUN did not inhibit growth, and 4 μg/ml of TUN completely inhibited growth (Fig. S1B).

10.1128/mBio.02272-21.1 FIG S1 Characterization of tunicamycin adaptors. SC5314 was grown in YPD broth (A) or streaked onto YPD agar (B), tunicamycin was added at the concentrations indicated, and growth was monitored at 37°C. In A, optical density (OD_595nm_) was measured every 15 min using a plate reader (Infinite F200 PRO; Tecan, Switzerland). Data are represented as the mean ± SD of three biological replicates. In B, the plates were photographed after 48 h. In C, approximately 1 million cells of SC5314 were spread onto YPD agar plates supplemented with TUN. The plates were incubated at 37°C for 5 days. In D, 18 and 5 adaptors, picked up from the plates containing 4 μg/ml and 8 μg/ml of TUN, respectively, were compared with the parent for tolerance to TUN using spot assays. In E, SC5314 was grown in YPD broth containing 1 μg/ml of TUN YPD broth without drug (control) for 24 h. A total of 120 colonies were randomly selected and tested using spot assays performed on YPD agar plates containing TUN as indicated. The parent strain is indicated by a blue circle. The tolerant colony identified is indicated by a red circle. In F, SC5314 was grown in YPD broth containing 1 μg/ml of TUN YPD broth without drug (control) for 24 h as described in Fig. S1E. The culture was diluted 1:1,000 into fresh YPD broth containing the same concentration of TUN and was incubated for another 24 h. A total of 95 randomly selected colonies from the culture with TUN were tested using spot assays on YPD agar plates supplemented with TUN. The parent strain is indicated by a blue circle. The three tolerant colonies are indicated by red circles. In G, SC5314 was passaged daily in YPD broth containing the concentrations of TUN indicated. On day 10, 18 colonies randomly selected from each culture were tested using spot assays. Download FIG S1, PDF file, 2.3 MB. Copyright © 2021 Yang et al. 2021 Yang et al. https://creativecommons.org/licenses/by/4.0/ This content is distributed under the terms of the Creative Commons Attribution 4.0 International license.

We used three approaches to obtain TUN adaptors, which are defined here as colonies that appeared and grew at TUN concentrations of ≥4 μg/ml. The first approach was to select on inhibitory TUN concentrations by plating ∼1 × 10^6^ cells onto yeast extract-peptone-dextrose (YPD)-agar plates containing 4 μg/ml or 8 μg/ml TUN. After 5 days, ∼2,300 colonies (0.23%) appeared on plates with 4 μg/ml TUN, and only 6 colonies appeared on 8 μg/ml TUN (Fig. S1C). We randomly selected 18 colonies from the 4-μg/ml TUN plate (6 smaller, 6 moderate, and 6 larger colony sizes). All 6 colonies that appeared on 8 μg/ml TUN were selected, but 1 colony grew very slowly and was excluded from further analysis.

In spot assays, 17 of the 18 4-μg/ml TUN adaptors grew on 4 μg/ml but not on 8 μg/ml TUN; the remaining adaptor grew on both 4 μg/ml and 8 μg/ml TUN. All 5 colonies selected on a 8-μg/ml TUN plate were tolerant to 8 μg/ml of TUN (Fig. S1D). Thus, exposure to inhibitory TUN concentrations usually selected for progeny adapted to the level of TUN selection.

The second approach was to select for adaptors following a short time exposure to subinhibitory TUN concentrations. TUN at 1 μg/ml slightly inhibited the growth of SC5314 in YPD broth. In another experiment, SC5314 at low density (2.5 × 10^3^ cells/ml) was grown in 1.5 ml YPD broth containing 1 μg/ml of TUN for 24 h (24-h exposure) and then diluted 1:1,000 to another tube at the same TUN concentration for an additional 24 h (48-h exposure). Each culture was washed, diluted with distilled water, and plated onto YPD agar plates (no drug selection) and 120 (24 h) and 95 (48 h) colonies were tested for TUN susceptibility using spot assays. Only one of the 24-h colonies (Fig. S1E) and three of the 48-h colonies were TUN tolerant (Fig. S1F). Thus, the short time exposure to a subinhibitory concentration of TUN was sufficient to allow tolerant cells to be selected at a frequency of ∼1% to 3%, and a longer exposure time increased the proportion of cells that were TUN tolerant.

The third approach was to select colonies following 10 days of exposure to subinhibitory TUN concentrations. SC5314 was passaged daily in YPD broth (control) or YPD broth supplemented with subinhibitory concentrations of TUN (0.5 μg/ml, 1 μg/ml, and 2 μg/ml). After the 10th passage, cells were plated onto YPD plates (no drug selection), and 18 colonies from each culture were randomly chosen for further analysis. Among them, 0 (0%), 2 (11.1%), 15 (83.3%), and 18 (100%) colonies, derived from passage in YPD broth containing 0 μg/ml, 0.5 μg/ml, 1 μg/ml, and 2 μg/ml, respectively, yielded isolates that had acquired TUN tolerance to TUN (Fig. S1G).

**FIG 1:**
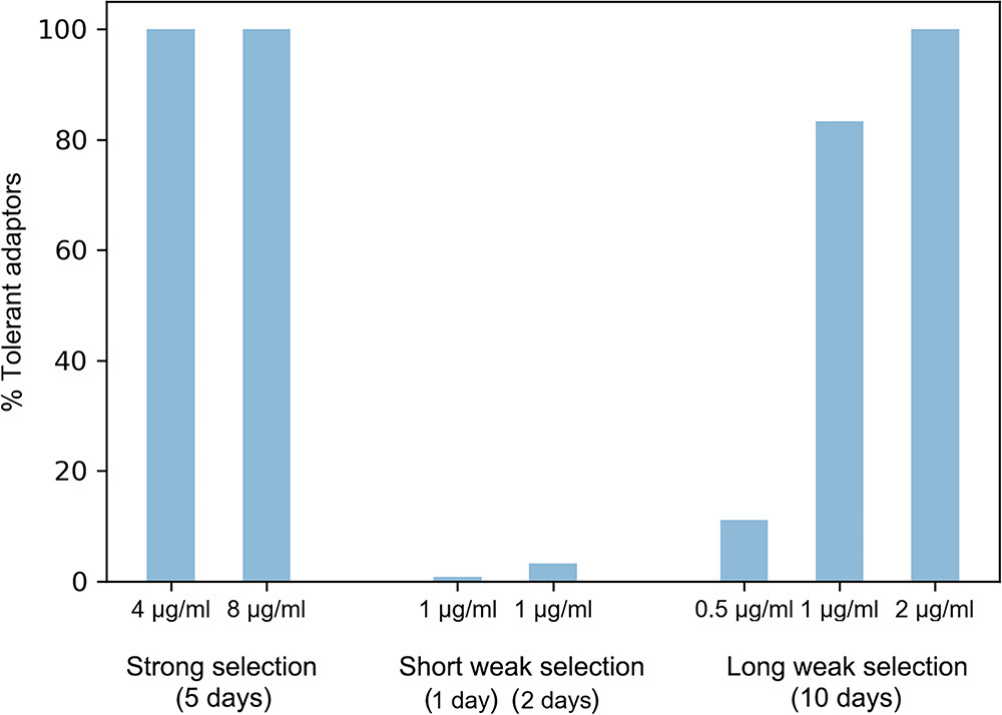
Proportion of TUN-tolerant adaptors as a function of tunicamycin selection conditions. Proportion of tolerant adaptors, out of the total colonies tested, under the range of different selection conditions used in this study. Under each selection condition, some colonies were randomly chosen and tested for tolerance to TUN by spot assay. The number of adaptors tested was 18 and 5 adaptors from YPD+TUN plates for 5 days in 4 μg/ml and 8 μg/ml TUN, respectively; 120 and 95 adaptors from 1 μg/ml TUN liquid cultures for 1 and 2 days, respectively; and 18 adaptors from 10-day passage in 0.5 μg/ml, 1 μg/ml, and 2 μg/ml liquid cultures.

Taken together, these results show that under strong selection using inhibitory concentrations of TUN (4 μg/ml and 8 μg/m), all the adaptors (100%) obtained tolerance to TUN. Subinhibitory concentrations of TUN (1 μg/ml) yielded only a small proportion of colonies (1.1% at 24 h and 3.2% at 48 h) with TUN tolerance when the exposure time was short (1 to 2 days), while a higher proportion of selected colonies were adaptors that resulted when exposure time was 5 to 10 times longer (Fig. 1).

### Chr2 duplication is the major mechanism of tolerance to tunicamycin.

**FIG 2:**
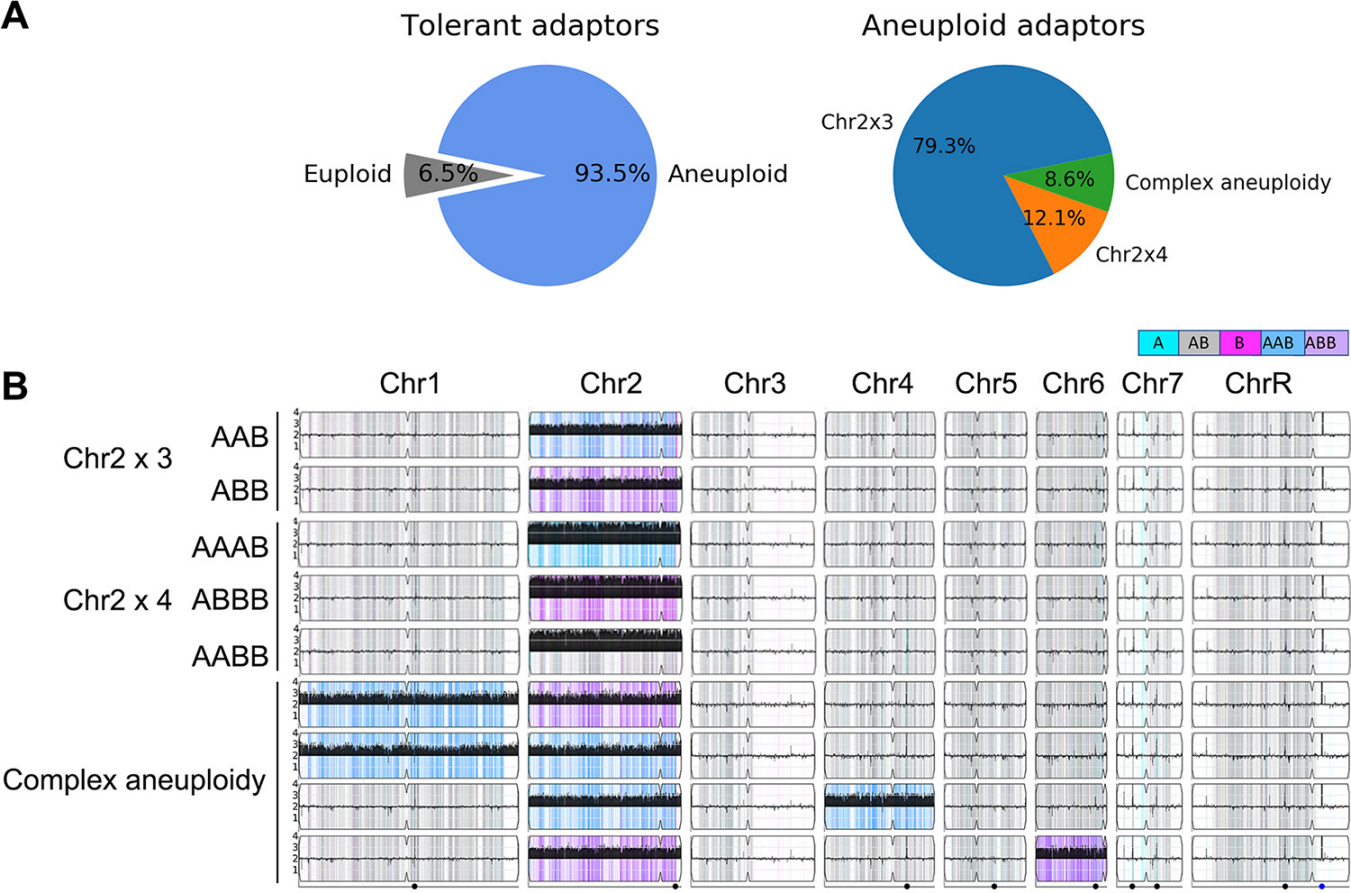
Distribution of tunicamycin-tolerant adaptors. Karyotypes of all 62 tolerant adaptor colonies were sequenced, and their copy number was visualized using Ymap (25). (A) The proportion of adaptors that were aneuploid (left) and the proportion of aneuploid adaptors with different karyotypes (right). (B) Representatives of the different recurrent karyotypes identified.

All the tolerant (*n* = 62) adaptors obtained from the 3 approaches were sequenced and analyzed by Ymap to detect ploidy and allele specificity (25) (Fig. 2). Among the 18 adaptors obtained from 4 μg/ml TUN, 17 acquired Chr2x3 and 1 acquired Chr2x4. For Chr2, duplication of either homolog (AAB or ABB) appeared with a similar frequency (9 versus 8, respectively). All 5 adaptors from 8 μg/ml TUN acquired tetrasomy of Chr2 (Chr2x4) with a similarly random distribution of homologs, as follows: 1 AAAB, 2 AABB, and 2 ABBB (see Fig. S2A in the supplemental material). The Chr2x3 adaptors were tolerant to 4 μg/ml but not to 8 μg/ml of TUN, and the Chr2x4 adaptors all were tolerant to 8 μg/ml of TUN (Fig. S1C, D), indicating that the extent of Chr2 duplication correlated with the level of tolerance to TUN and that there was no obvious allele specificity affecting the growth of adaptors on TUN.

10.1128/mBio.02272-21.2 FIG S2 Ymap analysis of tunicamycin-tolerant adaptors. Karyotypes of all sequenced TUN-tolerant adaptors obtained from YPD plates supplemented with 4 μg/ml or 8 μg/ml tunicamycin (A); from 24 h or 48 h evolution in YPD broth supplemented with 1 μg/ml tunicamycin (B); or from 10-day passage in YPD broth supplemented with 0.5 μg/ml, 1 μg/ml, and 2 μg/ml tunicamycin (C) were visualized using Ymap (25). Download FIG S2, PDF file, 2.0 MB. Copyright © 2021 Yang et al. 2021 Yang et al. https://creativecommons.org/licenses/by/4.0/ This content is distributed under the terms of the Creative Commons Attribution 4.0 International license.

The 24-h and 48-h TUN tolerant adaptors from 1 μg/ml of TUN also were Chr2x3 (AAB) (Fig. S2B). Among the 10-day (0.5, 1.0, and 2.0 μg/ml TUN) adaptors, 4 were euploid and the others were aneuploid for Chr2; 29 had Chr2x3, including 5 that had trisomy of either Chr1 or Chr6 in addition to Chr2x3; and 1 was Chr2x4 (ABBB) (Fig. S2C). Thus, all aneuploids exhibiting TUN tolerance carried least one extra copy of Chr2. To ask about the contribution of Chr1, Chr4, or Chr6 to TUN tolerance in the absence of Chr2x3, we utilized our C. albicans aneuploid collection, in which each strain is trisomic for a single chromosome (26). Importantly, the Chr2x3 isolate, which had never been exposed to TUN, was tolerant to TUN. Trisomies of all other chromosomes, including Chr1x3, Chr4x3, or Chr6x3, could not grow at 4 μg/ml TUN (see Fig. S3 in the supplemental material). Whether these extra chromosomes have an additional benefit or are neutral or detrimental “hitchhikers” along with Chr2x3 remains to be determined. Taken together, 93.5% (58 out of 62) of the tolerant adaptors had Chr2 duplication, of which 91.4% (53 out of 58) had only Chr2 trisomy or tetrasomy without another chromosome change. Notably, Chr2 duplication alone was sufficient to cause TUN tolerance with or without prior exposure to TUN.

10.1128/mBio.02272-21.3 FIG S3 Test of aneuploid strains for tolerance to tunicamycin. TUN tolerance was tested using a collection of aneuploid strains carrying one trisomic chromosome per strain (26). Only the Chr2x3 strain exhibited tolerance to TUN. Download FIG S3, PDF file, 2.8 MB. Copyright © 2021 Yang et al. 2021 Yang et al. https://creativecommons.org/licenses/by/4.0/ This content is distributed under the terms of the Creative Commons Attribution 4.0 International license.

### Transcriptome changes in response to tunicamycin treatment.

**FIG 3:**
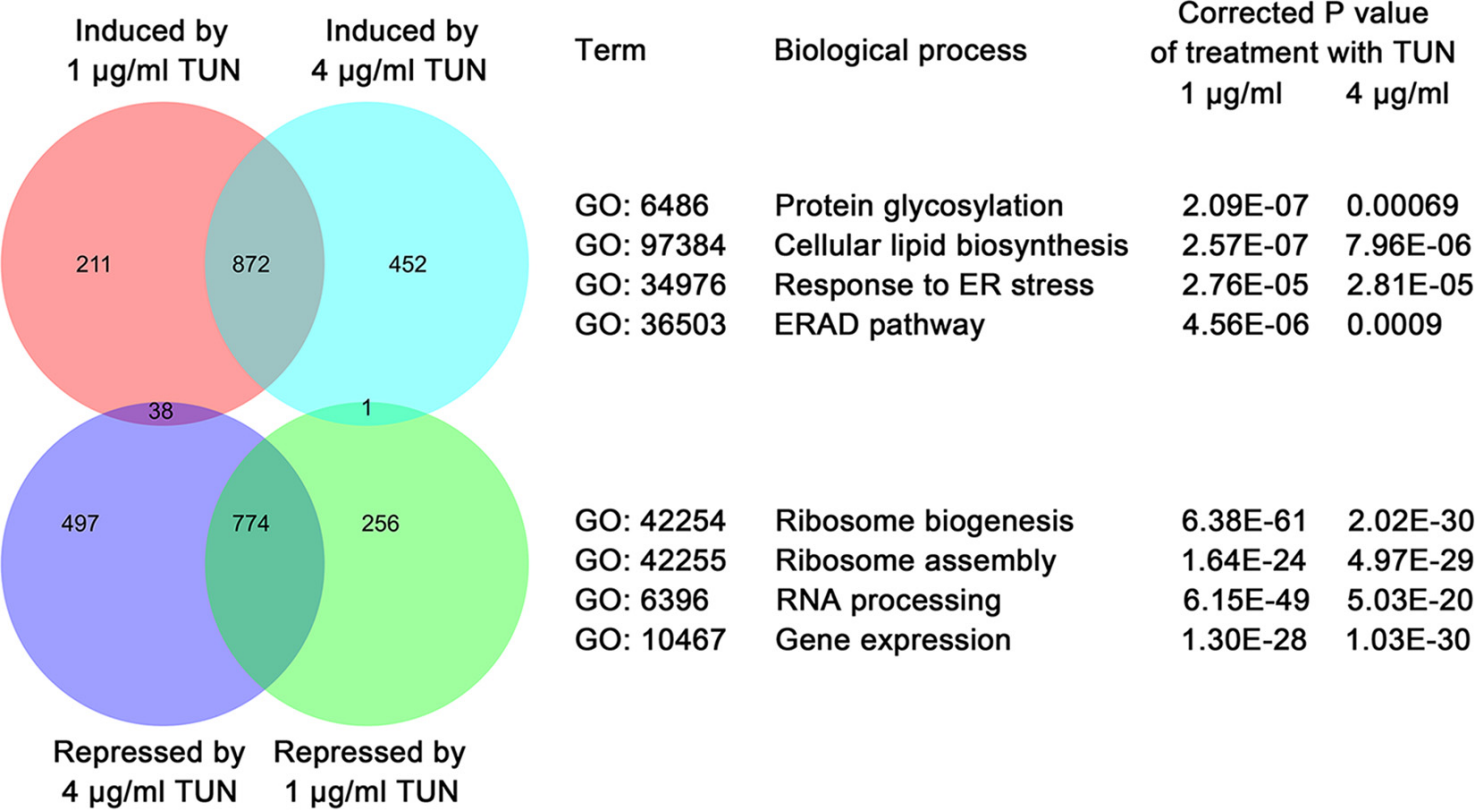
Transcriptomic analysis of cells grown in subinhibitory and inhibitory TUN. TUN concentrations relative to no TUN. Log-phase cells of SC5314 were treated with 1 μg/ml (subinhibitory) and 4 μg/ml (inhibitory) TUN for 1 h and analyzed as detailed in the Materials and Methods. The number of transcripts with expression increases or decreases, relative to the no-drug control, and overlaps between the two drug treatments, are shown (left). Gene Ontology (GO) enrichment analysis was performed for both induced and repressed genes, separately, for each treatment (right). GO process terms enriched (top lists) or depleted (bottom list) relative to no-drug treatment in both 1-μg/ml and 4-μg/ml TUN treatments.

To ask why both subinhibitory and inhibitory concentrations of TUN yielded the same aneuploid adaptors, we investigated the transcriptomes of cells treated with subinhibitory (1 μg/ml) and inhibitory (4 μg/ml) concentrations of TUN using transcriptome sequencing (RNA-seq). Cells treated with 1 μg/ml had 2,152 differentially expressed genes compared with the no-drug treatment control; 1,121 genes were more abundant and 1,031 genes were less abundant than in the no-drug control cells. In the 4-μg/ml TUN-treated cells, there were 2,634 differentially expressed genes, including 1,325 genes that were more abundant and 1,309 genes that were less abundant than in the control cells (see Table S3 in the supplemental material). Of these, 872 genes were induced and 774 genes were repressed in both TUN concentrations (Fig. 3, left).

10.1128/mBio.02272-21.9 TABLE S3 Differentially expressed genes in TUN treatment Table S3, PDF file, 0.7 MB. Copyright © 2021 Yang et al. 2021 Yang et al. https://creativecommons.org/licenses/by/4.0/ This content is distributed under the terms of the Creative Commons Attribution 4.0 International license.

Genes involved in several biological processes reminiscent of ER stress were commonly enriched in the two treatments. Induced genes were enriched in processes such as protein glycosylation, cellular lipid biosynthesis, response to endoplasmic reticulum stress, and the endoplasmic-reticulum-associated protein degradation (ERAD) pathway. Repressed genes were enriched in ribosome biogenesis, ribosome assembly, RNA processing, and gene expression (Fig. 3, right; see Table S4 in the supplemental material). Thus, both subinhibitory and inhibitory concentrations of TUN induced similar cellular responses, including UPR.

10.1128/mBio.02272-21.10 TABLE S4 GO enrichment of differentially expressed genes Table S4, PDF file, 0.1 MB. Copyright © 2021 Yang et al. 2021 Yang et al. https://creativecommons.org/licenses/by/4.0/ This content is distributed under the terms of the Creative Commons Attribution 4.0 International license.

### Aneuploid, but not euploid, adaptors conferred cross-adaptation to other drugs.

**FIG 4:**
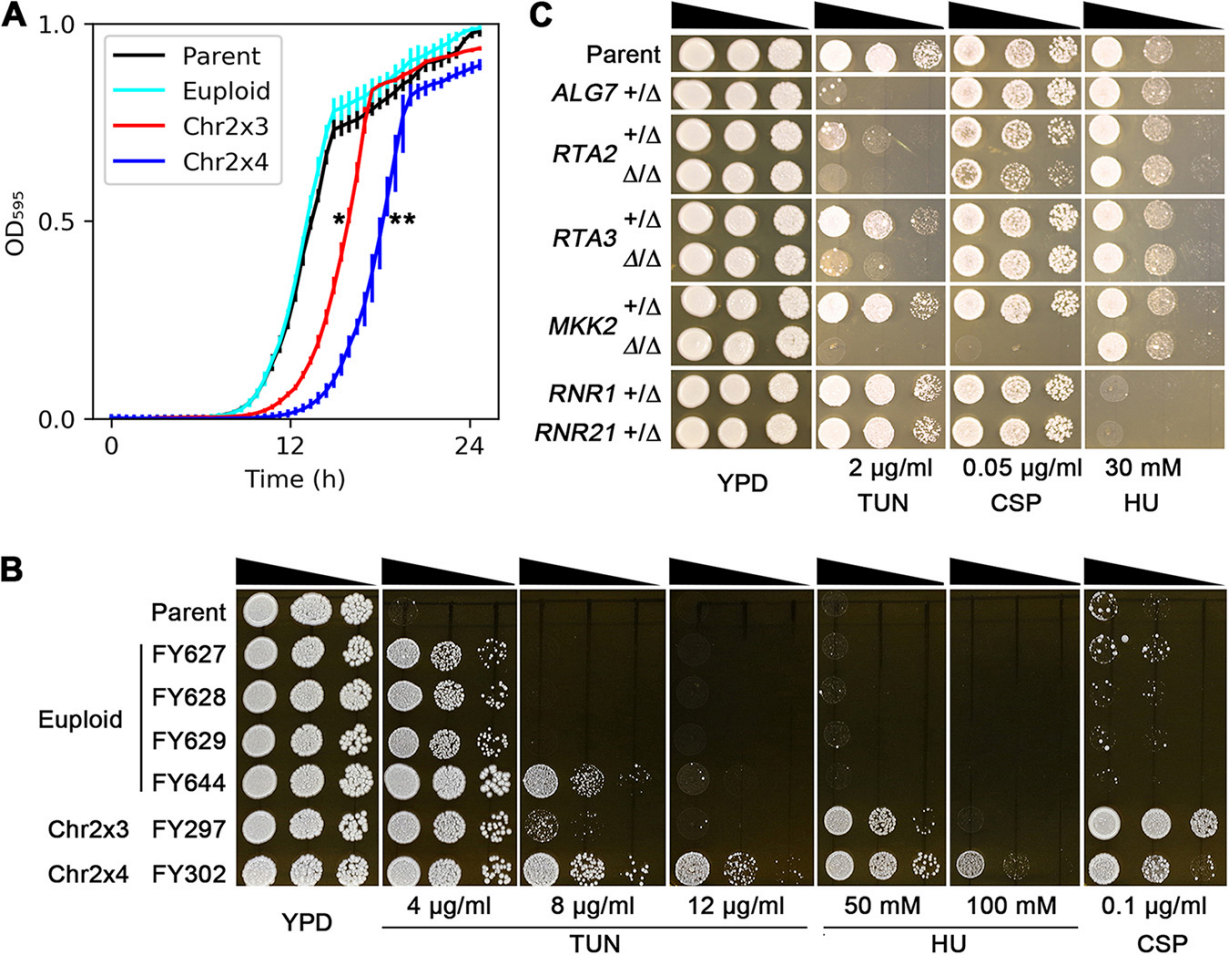
Fitness cost of adaptation to tunicamycin is karyotype dependent. (A) Fitness of euploid and aneuploid colonies was measured in YPD liquid at 37°C without drug stress. Optical density (OD_595nm_) was measured every 30 min using a plate reader (Infinite F200 PRO; Tecan, Switzerland). Data are represented as the mean ± SD of three biological replicates; *, *P* < 0.05; **, *P* < 0.001; determined by two-tailed tests. The plot was generated using a seaborn library (74). (B) Growth in the presence of tunicamycin (TUN), caspofungin (CSP), or hydroxyurea (HU) on YPD agar medium at 37°C for 48 h. (C) One or both copies of essential genes (+/Δ) and nonessential genes (Δ/Δ), respectively, were deleted as indicated. Images recorded growth in the presence of the indicated drug on YPD after 48 h of incubation at 37°C.

C. albicans aneuploid strains usually have at least a minor fitness cost in rich medium, and specific aneuploid isolates confer a fitness benefit under particular stress(es) (26). Consistent with this research, here, aneuploid TUN adaptors were less fit in YPD medium than euploid adaptor isolates (Fig. 4A) and were more fit than the parent in the presence of TUN (Fig. 4B, and Fig. S2 to S5). Furthermore, Chr2x4 isolates were less fit than Chr2x3 isolates. The improved fitness of the aneuploid isolates on a drug is relative to the parent that cannot grow on the drug. These results imply that the fitness benefit of being able to grow in the presence of a drug is higher than the cost incurred by the extra chromosomal DNA, the many RNAs, and/or proteins expressed from across the aneuploid chromosome.

Earlier work (17) found that Chr2 trisomy promoted cross-adaptation to both CSP, a first-line antifungal, and to HU, a chemotherapeutic drug. Accordingly, we tested the ability of the TUN-tolerant Chr2x3 and Chr2x4 isolates to grow in TUN, HU, and CSP using spot assays. As expected, all adaptors with extra Chr2 copies were able to grow on TUN, HU, and CSP at concentrations that inhibited the growth of the parent. Furthermore, euploid TUN-tolerant isolates were not tolerant to either HU or CSP (Fig. 4B). We next analyzed genes on Chr2 that were associated with tolerance to TUN, CSP, and HU. *ALG7* (24) and *RTA2* (27) are associated with TUN tolerance. *ALG7* encodes a UDP-GlcNAc:dolichol phosphate *N*-acetylglucosamine-1-phosphate transferase that mediates the first step in the protein *N*-glycosylation pathway (28). *RTA2* encodes a downstream effector of the calcineurin pathway. *RTA2* is required for the expression of TUN-induced *UPR* genes (27). In addition, *RTA3*, a paralog of *RTA2*, maps immediately upstream of *RTA2* on Chr2; no role of *RTA3* in ER stress responses has been reported. *MKK2* encodes a component of the PKC pathway that is required for CSP tolerance (29). *RNR1* and *RNR21*, which encode ribonucleotide reductases, are associated with HU tolerance (17). Deletion of *ALG7*, *RTA2*, and *RTA3* decreased TUN tolerance and did not affect growth on CSP or HU. Interestingly, deletion of *MKK2* decreased both TUN and CSP tolerance but not HU tolerance. Deletion of *RNR1* and *RNR21* decreased HU tolerance but not tolerance to TUN or CSP (Fig. 4C). Thus, aneuploidy conferred adaptation to additional stresses, primarily by increasing the number of copies of different genes on a single chromosome.

In broth microdilution assays interpreted at 24 h of TUN exposure (see Fig. S4 in the supplemental material), the euploid as well as the aneuploid TUN adaptors had MIC levels indistinguishable from that of the parent strain. However, after 48 h of TUN exposure, all the adaptors had higher TUN MICs, and the aneuploid adaptors had higher CSP MICs as well. Thus, consistent with the definition of drug resistance as robust growth appearing within 24 h of drug exposure and antifungal tolerance as delayed growth appearing at or after 48 h (5), we use the term tolerance to refer to the TUN adaptors.

10.1128/mBio.02272-21.4 FIG S4 Broth microdilution assay. Growth of tunicamycin adaptor colonies and the parent strain in YPD broth supplemented with caspofungin (CSP, A) or tunicamycin (TUN, B) using drug concentrations as indicated. For each strain, approximately 2.5 × 10^3^ cells/ml were grown in 150 μl YPD with or without the drugs in a 96-well plate at 37°C. OD_595_ was monitored using a plate reader (Infinite F200 PRO; Tecan, Switzerland) at 15-min time intervals for 48 h. Download FIG S4, PDF file, 0.9 MB. Copyright © 2021 Yang et al. 2021 Yang et al. https://creativecommons.org/licenses/by/4.0/ This content is distributed under the terms of the Creative Commons Attribution 4.0 International license.

We also asked if aneuploidy generally caused cross-tolerance to azole antifungals, the most widely used antifungal drugs. Neither the Chr2x3 nor the Chr2x4 adaptors grew well; in fact, both aneuploids were more susceptible to fluconazole (see Fig. S5 in the supplemental material). Therefore, aneuploidy per se does not cause all types of antifungal tolerance. Rather, aneuploidy of a particular chromosome underpins the ability to tolerate exposure to particular drugs.

10.1128/mBio.02272-21.5 FIG S5 Tolerance of tunicamycin adaptors to fluconazole. Tolerance to fluconazole was measured using disk diffusion assays. Each disk contained 25 μg fluconazole incubated at 30°C. Images were collected after 48 h of growth. Note that the euploid adaptor has a resistance level (zone of inhibition radius) similar to the parent, while both aneuploid strains had reduced resistance (larger zone of inhibition radii) relative to the parent. Download FIG S5, PDF file, 2.7 MB. Copyright © 2021 Yang et al. 2021 Yang et al. https://creativecommons.org/licenses/by/4.0/ This content is distributed under the terms of the Creative Commons Attribution 4.0 International license.

Aneuploid strains are generally less stable than euploid mutants, as they can rapidly revert to euploidy when grown on rich medium (17, 26). Aneuploid colonies generally grow more slowly than euploid colonies; accordingly, colony size variations when aneuploid strains were grown without drug selection is a common feature of unstable aneuploid isolates (26), with the large colonies having lost the aneuploidy and the improved ability to grow on the drug. Tolerance to CSP and HU was lost in Chr2x3 isolates reverted to Chr2x2 (17). In this current study, Chr2x3 adaptors also were unstable (see Fig. S6A in the supplemental material), producing small- and large-sized colonies on nonselective medium. Consistent with prior studies, on selective medium, the small colonies were tolerant to TUN, CSP, and HU, and the large colonies lost tolerance to all three compounds (Fig. S6B). Therefore, extra copies of Chr2 confer the ability to grow slowly in TUN, CSP, and HU.

10.1128/mBio.02272-21.6 FIG S6 Tolerance to drugs is unstable under nonselective conditions. Approximately 100 cells from a Chr2x3 strain were spread onto YPD without drug and incubated at 37°C for 24 h. Yellow arrow, a small colony; cyan arrow, large colony (A). The small and large colonies and the parent were assayed for tolerance to tunicamycin (TUN), caspofungin (CSP), and hydroxyurea (HU) (B). The plates were photographed after 48 h at 37°C. Download FIG S6, PDF file, 1.5 MB. Copyright © 2021 Yang et al. 2021 Yang et al. https://creativecommons.org/licenses/by/4.0/ This content is distributed under the terms of the Creative Commons Attribution 4.0 International license.

### Aneuploidy-mediated tolerance to tunicamycin and caspofungin relies on the PKC and calcineurin pathways.

**FIG 5:**
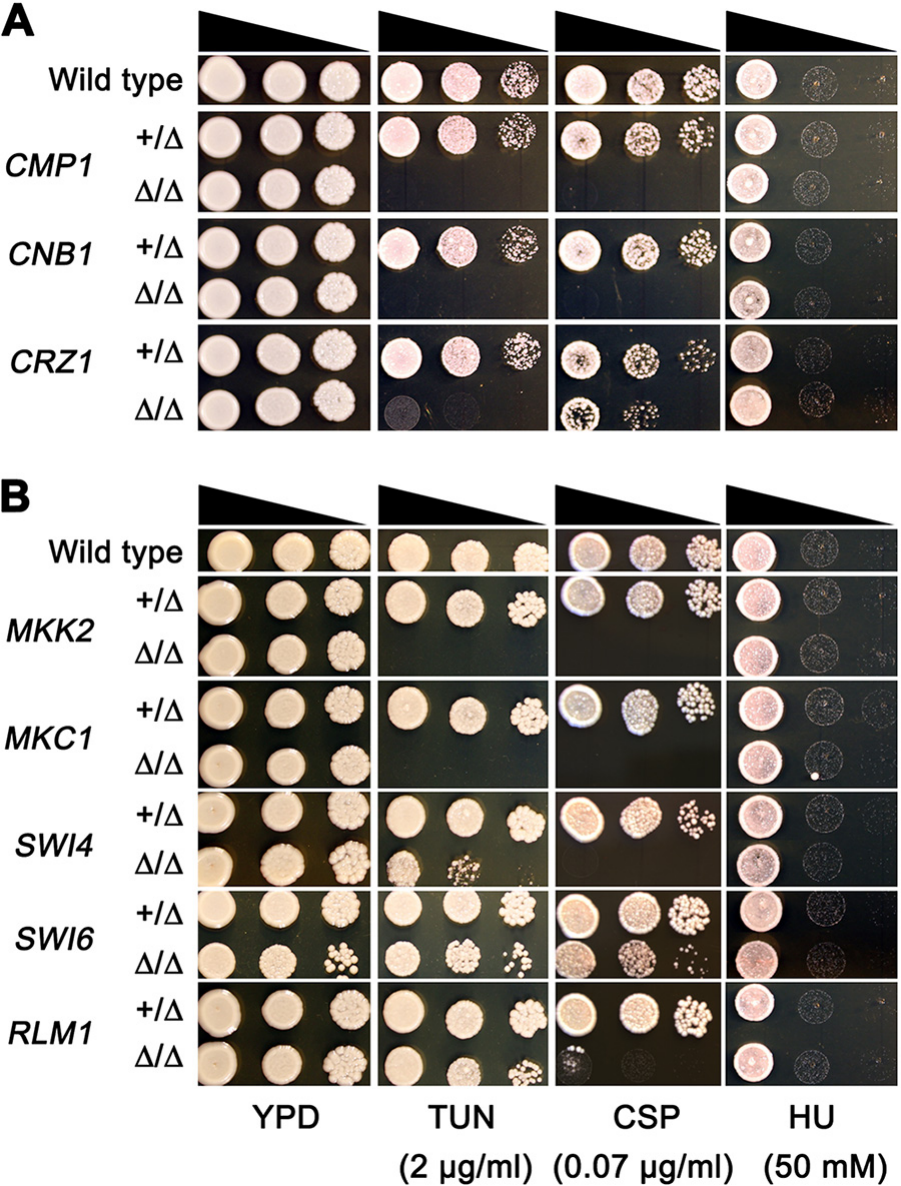
Calcineurin and PKC pathways are associated with tolerance to tunicamycin and caspofungin. Genes encoding components of the calcineurin pathway (A) and the PKC pathway (B) were deleted in SC5314. Spot assays were performed to compare the deletion strains and the wild-type parent for susceptibility to tunicamycin (TUN), caspofungin (CSP), and hydroxyurea (HU). Plates were incubated at 37°C for 48 h and then photographed.

*RTA2* is a downstream effector of the calcineurin pathway and is required for the TUN-induced ER stress response (27). The calcineurin pathway is also required for tolerance to echinocandins (30). To ask if the calcineurin pathway was required for cross tolerance to TUN, CSP, and HU, we deleted *CMP1* and *CNB1*, which encode the catalytic and regulatory subunits of calcineurin, respectively, and *CRZ1*, which encodes a downstream transcription factor. Of note, *cmp1Δ/Δ*, *cnb1Δ/Δ*, and *crz1Δ/Δ* strains were hypersensitive to TUN and CSP but not to HU (Fig. 5A).

*MKK2* is a component of the PKC pathway, which is required for CSP tolerance in S. cerevisiae (31) and C. albicans (29). The PKC pathway is also required for autophagy in response to TUN -induced ER stress in hepatocytes (32). To ask if the PKC pathway was required for cross tolerance to TUN, CSP, and HU, we deleted *MKK2* and *MKC1*, components of PKC pathway, as well as genes encoding downstream transcription factors, including *RLM1*, *SWI4*, and *SWI6*. Indeed, *mkk2Δ/Δ*, *mkc1Δ/Δ*, and *swi4Δ/Δ* strains were hypersensitive to TUN and CSP but not to HU. The *swi6Δ/Δ* and *rlm1Δ/Δ* strains were only sensitive to CSP (Fig. 5B). Thus, both calcineurin and the PKC pathways were required for tolerance to TUN and CSP.

## DISCUSSION

The endoplasmic reticulum, a feature of all eukaryotic cells, is the major site of protein folding and maturation (33). ER homeostasis can be perturbed by a broad spectrum of stresses, including a wide variety of physiological fluctuations (reviewed in references 34 and 35). Here, we studied how cells adapt to short and long time exposure to sublethal ER and how it affects their responses to other drugs. Specifically, we asked how C. albicans adapts to TUN-induced ER stress and whether adaptation to ER stress affects the ability of this common fungal pathogen to adapt to other antifungal drugs. We found that the most recurrent response to TUN is duplication of Chr2 and that Chr2x3 can be detected after a short time under strong TUN selection, as well as after short or longer time frames when TUN selection is weaker. Extra copies of Chr2 alleviate the ER stress, at least partially, with the level of TUN tolerance being proportional to the copy number of Chr2. Furthermore, Chr2x3 was selected irrespective of the TUN concentration or the time of TUN exposure. Furthermore, RNA-seq indicated that both weak and strong ER stress induced similar transcriptome changes, including induction of genes and biological processes reminiscent of the ER stress response which is highly conserved from yeast to human (36). Therefore, in C. albicans, aneuploidy appears rapidly in response to even weak ER stress. Finally, we identified several genes on Chr2 that are likely to affect TUN adaptation as well as other genes on the same chromosome, which facilitate adaptation to echinocandin drug CSP and to HU, a drug used for cancer chemotherapy.

We assume that, as in S. cerevisiae, TUN has two roles in these studies. First, it induces ER stress in C. albicans, which drives chromosome missegregation. Second, the selective pressure of growth in TUN plays a role in the length of time required to detect adaptor colonies, with a higher TUN concentration exerting a higher selection coefficient and thus a more frequent and rapid appearance of colonies that we selected as TUN adaptors. What is very clear is that extra copies of Chr2 provide a selective advantage under a very broad range of TUN stress conditions.

In S. cerevisiae, ChrII disomy was the major mechanism of adaptation to TUN. Three genes on ChrII (*ALG7*, *PRE7*, and YBR085C-A) were associated with TUN tolerance. In the C. albicans genome, *CaALG7* is on Chr2, *CaPRE7* is on Chr4, and no obvious homolog of YBR085C-A is evident. In C. albicans, since Chr2 duplication was the major mechanism of TUN tolerance and Chr4x3 was not associated with TUN tolerance, we focused on identifying genes on Chr2 that affect TUN tolerance. The essential gene *ALG7* was haploinsufficient; deleting one *ALG7* allele was sufficient to cause TUN hypersensitivity. *ALG7* encodes a UDP-GlcNAc:dolichol phosphate *N*-acetylglucosamine-1-phosphate transferase that mediates the first step in the protein N-glycosylation pathway (28). *RTA2*, also on Chr2, is a downstream effector of the calcineurin pathway. *RTA2* is required for the expression of TUN-induced UPR genes (27). *RTA2* encodes a member of the Rta1-family proteins involved in the lipid translocation (37). C. albicans has three Rta1-family proteins, namely, orf19.6224, *RTA3*, and *RTA4*, which reside on chromosomes 1, 2, and 4, respectively, with both *RTA2* and *RTA3* being associated with TUN tolerance (Fig. 4C). Like *ALG7*, *RTA2* was haploinsufficient for TUN hypersensitivity, while *RTA3* was hypersensitive to TUN only after deletion of both alleles. In addition, the PKC pathway component gene *MKK2*, also on Chr2, was associated with TUN tolerance (Fig. 4C). Therefore, Chr2 carries at least four genes, namely, *ALG7*, *RTA2*, *RTA3*, and *MKK2*, that affect TUN tolerance.

Chr2 is a large chromosome with 1,017 predicted ORFs providing many genes on the same chromosome with the potential to affect adaptation to different stresses. Because aneuploidy simultaneously alters the copy number of many genes and thus has the potential to alter stress tolerance to many stresses, it provides a rapid (and reversible) stress response, gene copy number, and transcription and translation levels that are generally proportional to gene copy number variations (38, 39). Although, dosage compensation at the transcriptional (40–43) and translational levels (44, 45) affects a subset of the gene products (reviewed in references 46–48). Aneuploidy often incurs a general fitness cost (26, 49, 50). Yet, aneuploidy can confer better fitness under particular stresses, and the same aneuploid chromosome can affect different stress responses either directly by regulating specific genes on the aneuploid chromosome (15, 17, 18, 51–54) or indirectly via regulatory circuits that affect genes on other chromosomes (55).

On Chr2, in addition to the presence of the four genes associated with TUN tolerance, there are genes associated with responses to other stresses. For example, *MKK2* is associated with tolerance to both TUN and CSP, and as shown previously (17), *RNR1* and *RNR21* are associated with HU tolerance. Therefore, Chr2x3 confers cross-tolerance to TUN, CSP, and HU, irrespective of the original conditions used to select for the aneuploid state. Furthermore, in addition to Mkk2, Mkc1, another component of the PKC pathway, is also required for both TUN and CSP tolerance. Two transcription factors, namely, Rlm1 and SBF (Swi4/Swi6), are activated by the PKC pathway (56). Rlm1 is a transcriptional activator of many cell wall protein genes and is required for resistance to numerous cell wall-perturbing treatments (57). Swi4 is required for both TUN and CSP tolerance. Swi6 and Rlm1 are required only for CSP tolerance and not for TUN tolerance. Similarly, calcineurin is required for tolerance to both TUN and CSP; yet, Rta2, a downstream effector of calcineurin, is associated only with TUN tolerance, and not with CSP tolerance. Therefore, both the PKC pathway and calcineurin are required for tolerance to TUN and CSP, but divergence of the downstream transcription factors contribute to their differential role in tolerance to TUN and CSP. Interestingly, our results on cross-tolerance to TUN and CSP are consistent with the observation that defects in the ER stress response pathway also cause hypersensitivity to cell wall-targeting agents (58).

In C. albicans, as well as in many other organisms, aneuploidy is a prevalent strategy of stress adaptation (59, 60; reviewed in references 61–64). Specifically, aneuploidy is often a rapid mechanism of adaption to antifungal stress (11–13, 16, 17; reviewed in reference 65) and also facilitates adaptation to other stresses *in vitro*, e.g., alternative carbon sources (66–68; reviewed in reference 69), and *in vivo*, e.g., passage in mouse blood (70), the oral cavity (71, 72), and the gastrointestinal tract (73). We posit that antifungal tolerance can appear due to direct selection by commonly used antifungal drugs or due to a hitchhiking effect, caused by other stresses, including ER stress. A critical question that needs to be addressed in future studies is whether short or chronic exposure to weak or strong physiological stresses would result in the emergence of aneuploidy and associated cross-adaptation to antifungals. We also cannot rule out that aneuploidy may confer altered virulence and pathogenesis of human fungal pathogens.

In conclusion, one or more extra copies of Chr2 facilitate rapid adaptation to TUN-induced ER stress in C. albicans. The acquisition of an extra chromosome exerts a hitchhiking effect by enabling cross-tolerance to at least two other drugs. Cross tolerance is mediated, in part, by multiple genes with distinct functions that confer tolerance to different drugs and by genes involved in pathways important for tolerance to more than one drug class. Finally, the hitchhiking effect can promote antifungal tolerance in a commensal organism like C. albicans such that, despite no prior antifungal drug exposure, a patient may be carrying aneuploid isolates tolerant to antifungal drugs.

## MATERIALS AND METHODS

### Growth curves.

Approximately 2.5 × 10^3^ cells/ml of SC5314 in 150 μl YPD with or without TUN were incubated in a 96-well plate at 37°C. The optical density at 595 nm (OD_595_) was monitored by a plate reader (Infinite F200 PRO; Tecan, Switzerland) at 30-min time intervals for 24 h. Data are represented as the mean ± SD of three biological replicates.

### Obtaining TUN adaptors from plates.

A total of 100 μl of 1 × 10^7^ cells/ml was spread onto YPD plates supplemented with 4 μg/ml and 8 μg/ml TUN or without TUN (control) and incubated at 37°C for 5 days. Adaptors were randomly chosen, streaked onto YPD plates and incubated at 37°C for 36 h. For each adaptor, several colonies with similar sizes were selected and frozen at −80°C.

### Obtaining progeny from short-term evolution in YPD broth supplemented with TUN.

A total of 2.5 × 10^3^ cells/ml of SC5314 were inoculated into 1.5 ml of YPD broth containing 1 μg/ml TUN and incubated for 24 h at 37°C (24-h exposure), and 1.5 μl of the culture was transferred to another 1.5-ml YPD broth aliquot supplemented with 1 μg/ml TUN and incubated for another 24 h (48 h exposure total). The 24 h- and 48 h-exposure cultures were washed and diluted with distilled water, and then ∼300 cells were spread onto YPD plates and incubated at 37°C for 36 h. Randomly, 120 colonies (24-h exposure) or 95 colonies (48-h exposure) from each plate were chosen for further analysis.

### Daily passage in YPD broth supplemented with TUN.

A total of 2.5 × 10^3^ cells/ml of SC5314 were inoculated into 1.5 ml of YPD broth supplemented with 0 μg/ml (control), 0.5 μg/ml, 1 μg/ml, or 2 μg/ml TUN. After 24 h of incubation at 37°C, a 1.5-μl culture was transferred to another 1.5-ml YPD broth aliquot containing the same TUN concentration. After 10 passages over 10 days, the cultures were washed and diluted with sterile water. Approximately 100 cells were spread onto YPD plates and incubated at 37°C for 36 h, and 18 colonies were randomly chosen from each plate for further analysis.

### RNA-Seq.

SC5314 was inoculated to a starting OD_600_ of 0.2 in 50 ml of YPD broth. The culture was incubated in a shaker at 37°C until the OD_600_ reached 1.0. The culture was divided into three batches, namely, control (only dimethyl sulfoxide [DMSO] was added), subinhibitory treatment (1 μg/ml TUN), and inhibitory treatment (4 μg/ml TUN) and then incubated with shaking for 3 h at 37°C. Cultures were collected by centrifugation, washed, and flash frozen in liquid nitrogen.

The total RNA was extracted for 9 independent samples, corresponding to 3 conditions and 3 biological replicates. Total RNA extraction and purification, library construction, and sequencing were performed as described previously (15).

### Statistical analysis.

The significance of differences between growth curves was determined using a two-tailed paired *t* test in GraphPad Prism (version 5.01).

### Miscellaneous.

Strains used in this study are listed in Table S1 in the supplemental material. Primers are listed in Table S2 in the supplemental material. Drugs were dissolved in dimethyl sulfoxide (DMSO) and stored at −20°C. Disk diffusion assays, spot assays, gene deletions, and next-generation sequencing (NGS) were performed as described previously (26).

10.1128/mBio.02272-21.7 TABLE S1 Strains used in this study Table S1, DOCX file, 0.02 MB. Copyright © 2021 Yang et al. 2021 Yang et al. https://creativecommons.org/licenses/by/4.0/ This content is distributed under the terms of the Creative Commons Attribution 4.0 International license.

10.1128/mBio.02272-21.8 TABLE S2 Primers used in this study Table S2, DOCX file, 0.02 MB. Copyright © 2021 Yang et al. 2021 Yang et al. https://creativecommons.org/licenses/by/4.0/ This content is distributed under the terms of the Creative Commons Attribution 4.0 International license.

### Data availability.

All sequence data are available in the ArrayExpress database at EMBL-EBI (www.ebi.ac.uk/arrayexpress) under accession number E-MTAB-10358 (DNA sequences) and E-MTAB-10391 (RNA sequences).
