# Supplementary material for: Tunicamycin Potentiates Antifungal Drug Tolerance via Aneuploidy in Candida albicans
Source: mBio. 2021 Aug 31;12(4):e02272-21. doi: 10.1128/mBio.02272-21 (PMC8406271; doi:10.1128/mBio.02272-21)
Supplement: FIG S1 [file mbio.02272-21-sf001.pdf]

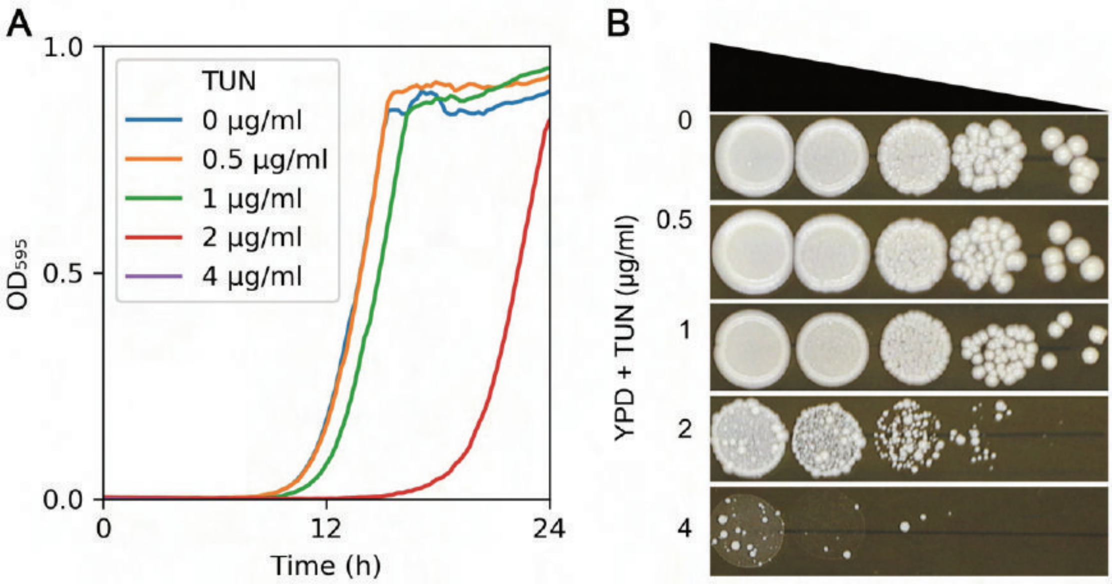
A
B
TUN
0 μg/ml
0.5 μg/ml
1 μg/ml
2 μg/ml
4 μg/ml
OD595
1.0
0.5
0.0
0
12
24
Time (h)
YPD + TUN (μg/ml)
0
0.5
1
2
4

C

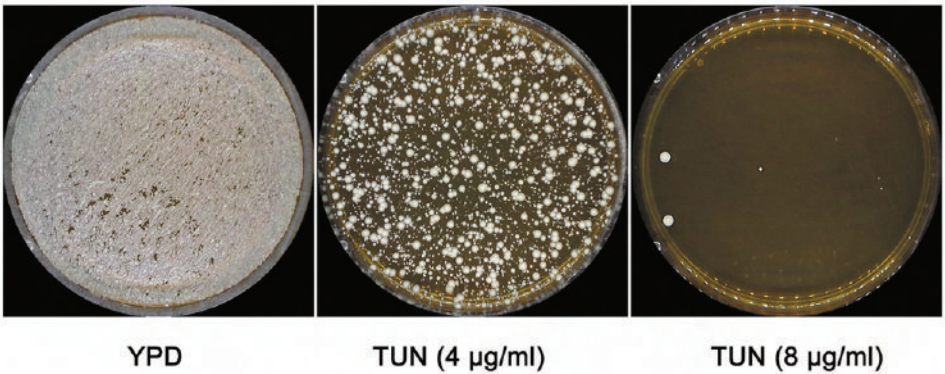
YPD
TUN (4 μg/ml)
TUN (8 μg/ml)

D

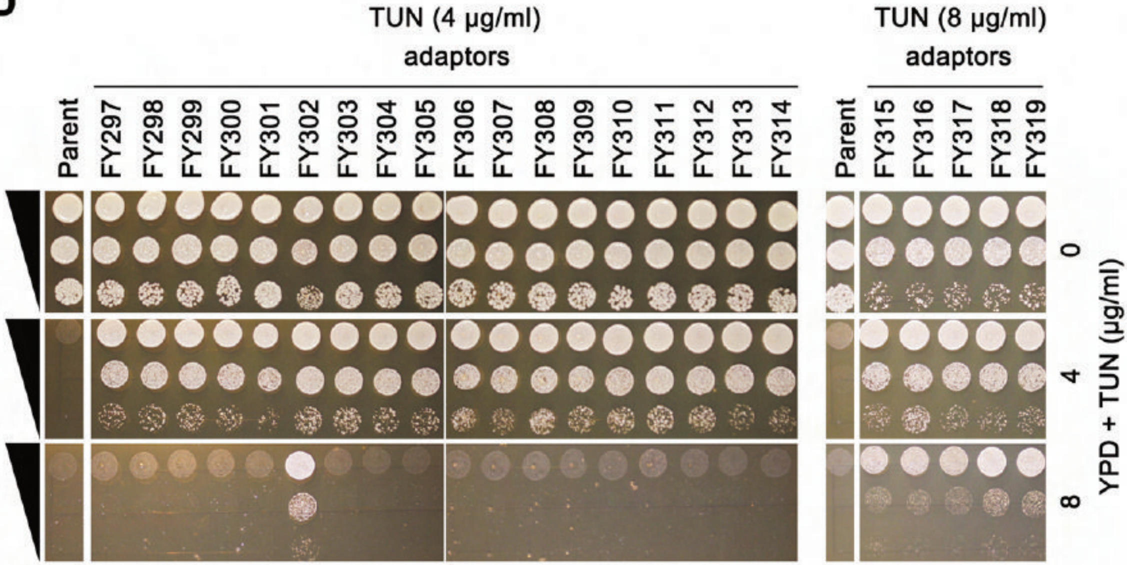
TUN (4 μg/ml)
adaptors
TUN (8 μg/ml)
adaptors
Parent
FY297
FY298
FY299
FY300
FY301
FY302
FY303
FY304
FY305
FY306
FY307
FY308
FY309
FY310
FY311
FY312
FY313
FY314
Parent
FY315
FY316
FY317
FY318
FY319
0
4
8
YPD + TUN (μg/ml)

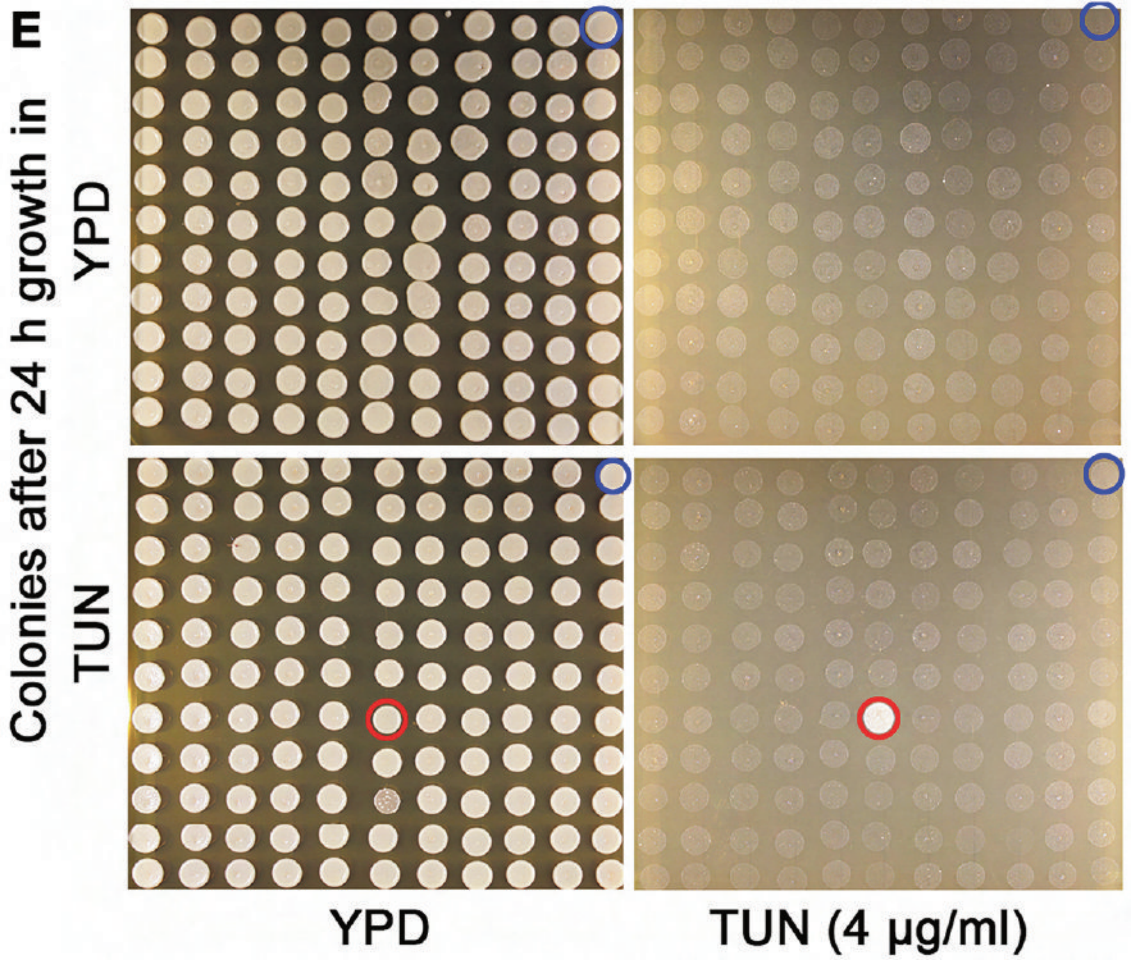
E
Colonies after 24 h growth in
YPD
TUN
YPD
TUN (4 µg/ml)

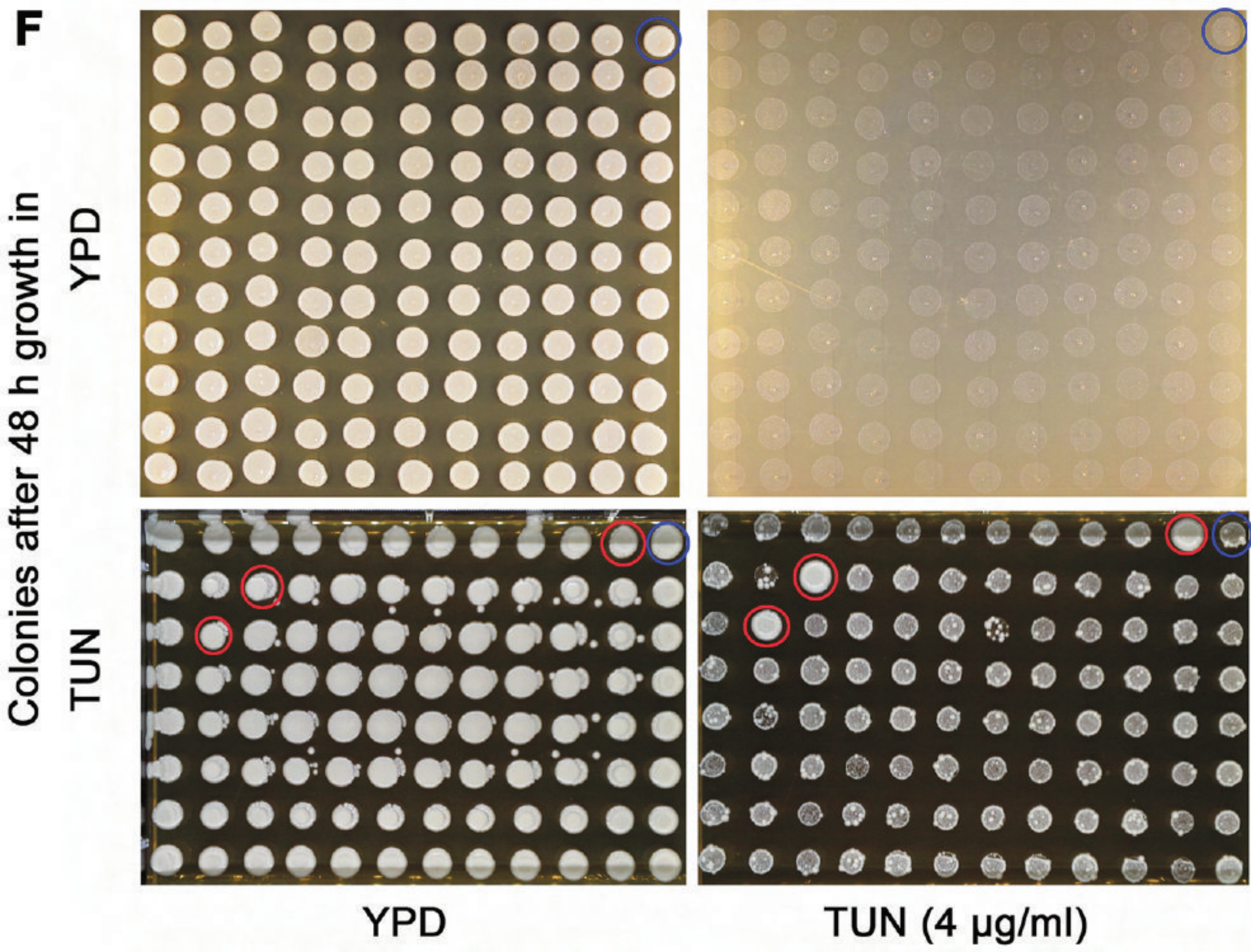
F
Colonies after 48 h growth in
YPD
TUN
YPD
TUN (4 μg/ml)

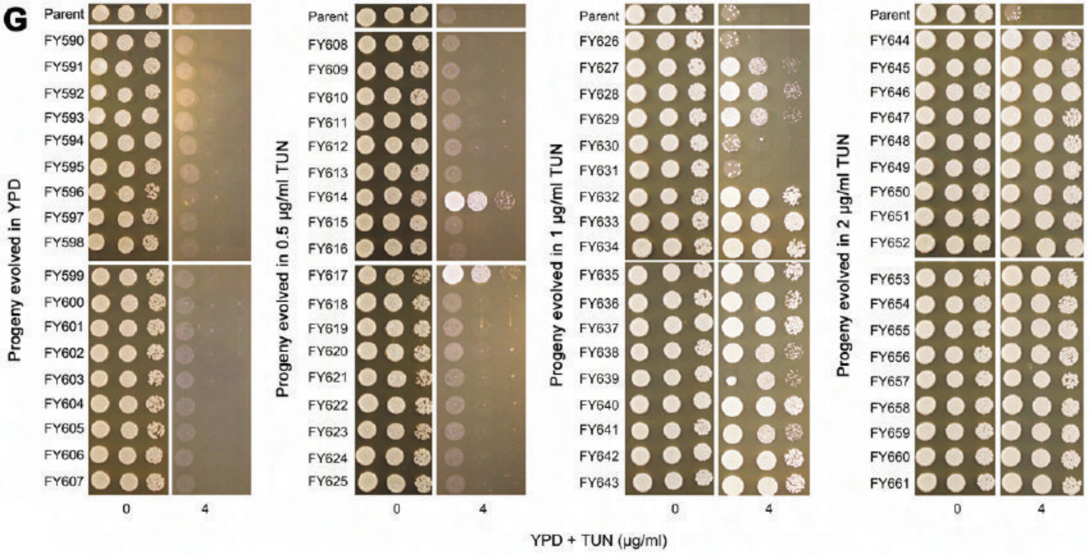
G
Progeny evolved in YPD
Progeny evolved in 0.5 µg/ml TUN
Progeny evolved in 1 µg/ml TUN
Progeny evolved in 2 µg/ml TUN
Parent
FY590
FY591
FY592
FY593
FY594
FY595
FY596
FY597
FY598
FY599
FY600
FY601
FY602
FY603
FY604
FY605
FY606
FY607
FY608
FY609
FY610
FY611
FY612
FY613
FY614
FY615
FY616
FY617
FY618
FY619
FY620
FY621
FY622
FY623
FY624
FY625
FY626
FY627
FY628
FY629
FY630
FY631
FY632
FY633
FY634
FY635
FY636
FY637
FY638
FY639
FY640
FY641
FY642
FY643
FY644
FY645
FY646
FY647
FY648
FY649
FY650
FY651
FY652
FY653
FY654
FY655
FY656
FY657
FY658
FY659
FY660
FY661
0
4
YPD + TUN (µg/ml)
