# Supplementary material for: Tunicamycin Potentiates Antifungal Drug Tolerance via Aneuploidy in Candida albicans
Source: mBio. 2021 Aug 31;12(4):e02272-21. doi: 10.1128/mBio.02272-21 (PMC8406271; doi:10.1128/mBio.02272-21)
Supplement: FIG S2 [file mbio.02272-21-sf002.pdf]

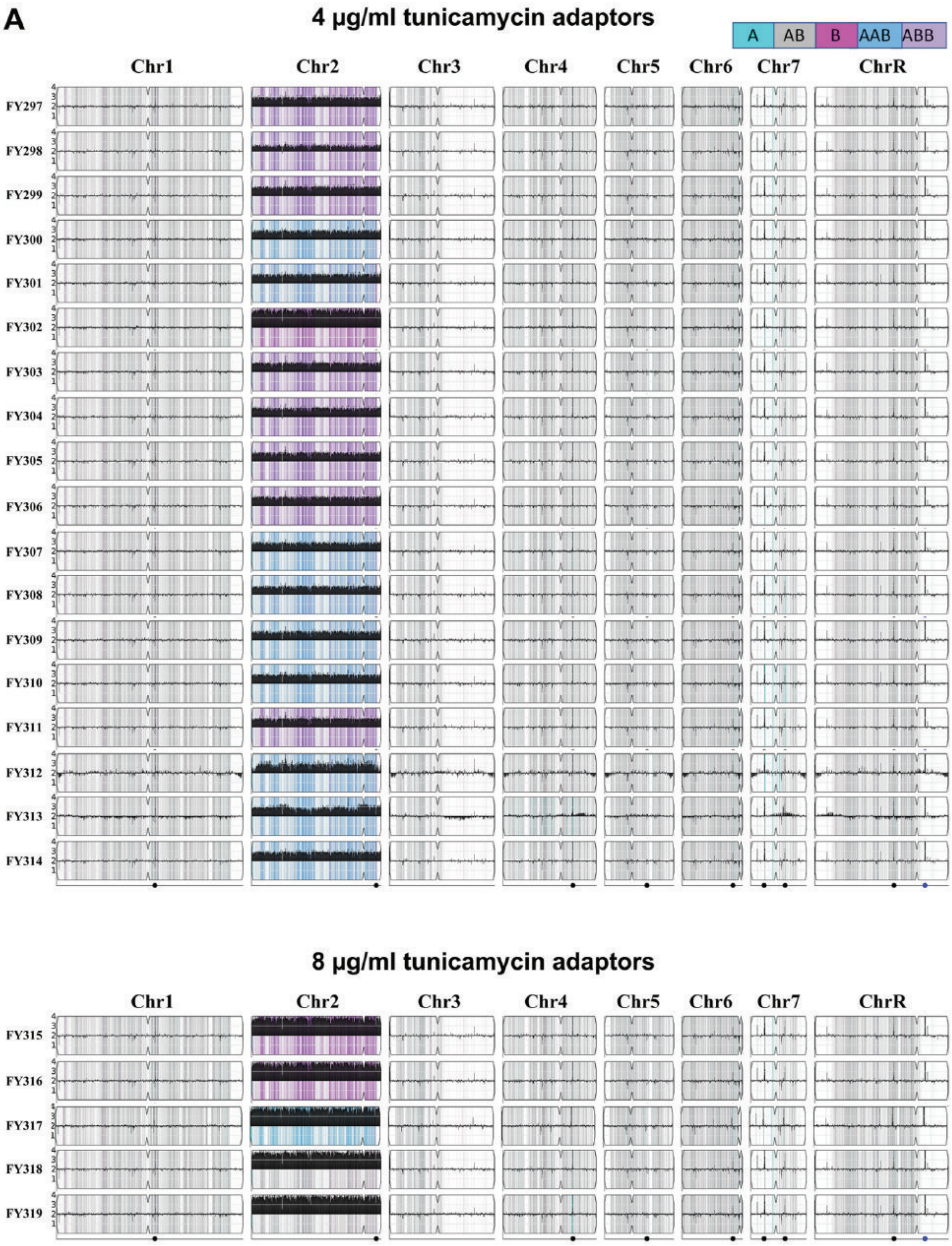

A
4 μg/ml tunicamycin adaptors
A
AB
B
AAB
ABB
Chr1
Chr2
Chr3
Chr4
Chr5
Chr6
Chr7
ChrR
FY297
FY298
FY299
FY300
FY301
FY302
FY303
FY304
FY305
FY306
FY307
FY308
FY309
FY310
FY311
FY312
FY313
FY314
8 μg/ml tunicamycin adaptors
Chr1
Chr2
Chr3
Chr4
Chr5
Chr6
Chr7
ChrR
FY315
FY316
FY317
FY318
FY319

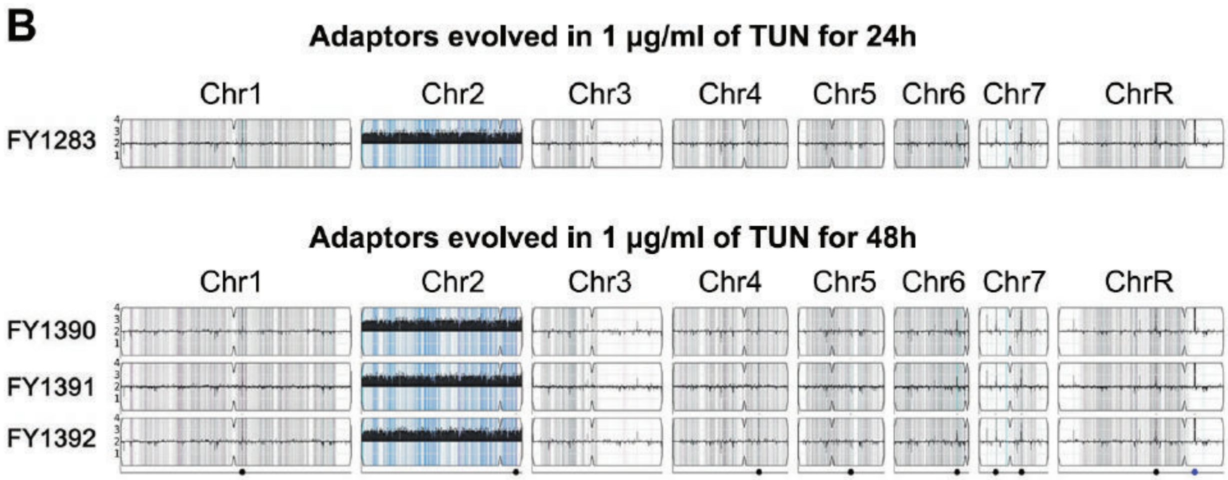
B
Adaptors evolved in 1 μg/ml of TUN for 24h
Chr1
Chr2
Chr3
Chr4
Chr5
Chr6
Chr7
ChrR
FY1283
Adaptors evolved in 1 μg/ml of TUN for 48h
FY1390
FY1391
FY1392

C

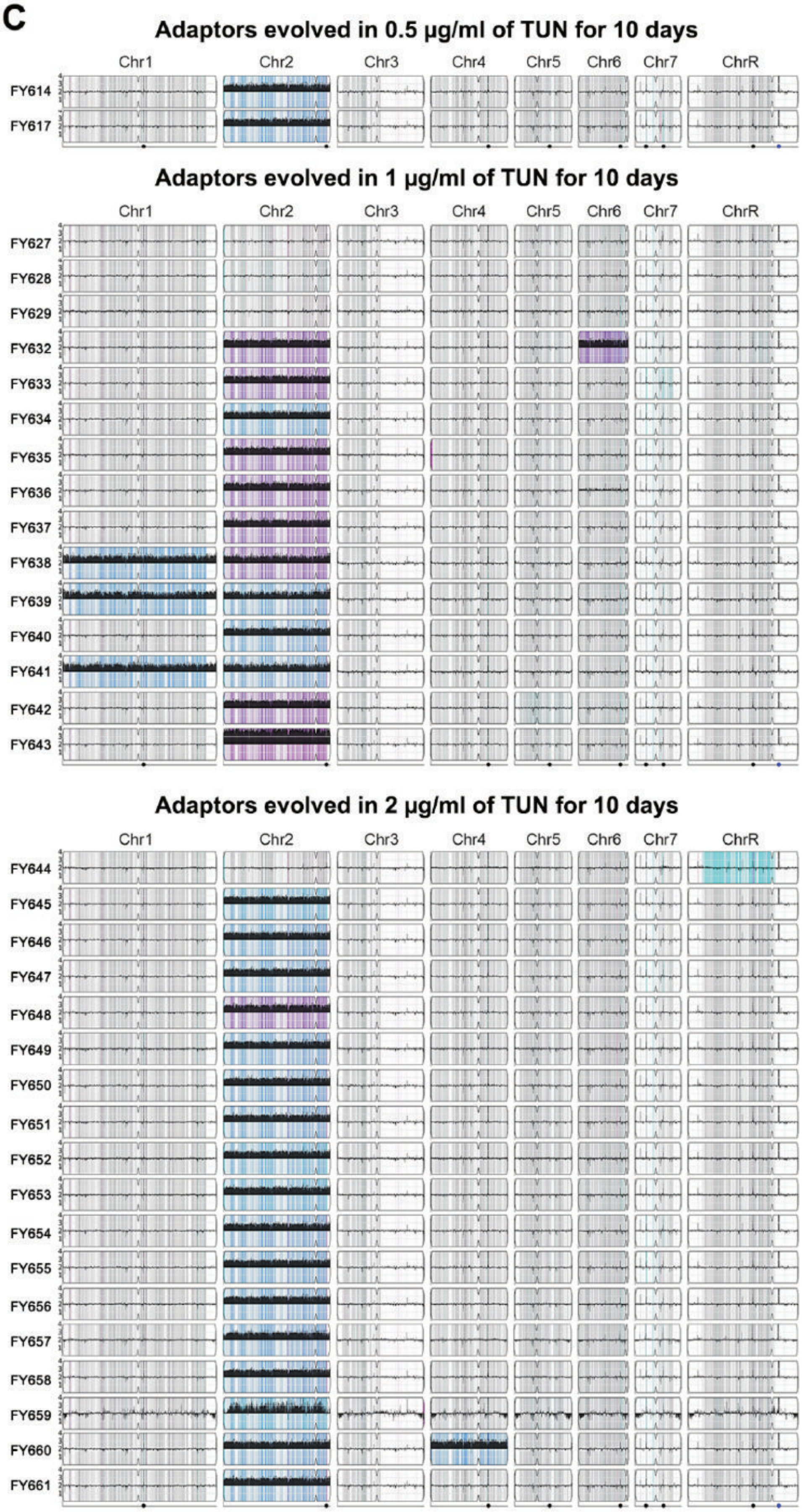

Adaptors evolved in 0.5 µg/ml of TUN for 10 days
Chr1
Chr2
Chr3
Chr4
Chr5
Chr6
Chr7
ChrR
FY614
FY617
Adaptors evolved in 1 µg/ml of TUN for 10 days
Chr1
Chr2
Chr3
Chr4
Chr5
Chr6
Chr7
ChrR
FY627
FY628
FY629
FY632
FY633
FY634
FY635
FY636
FY637
FY638
FY639
FY640
FY641
FY642
FY643
Adaptors evolved in 2 µg/ml of TUN for 10 days
Chr1
Chr2
Chr3
Chr4
Chr5
Chr6
Chr7
ChrR
FY644
FY645
FY646
FY647
FY648
FY649
FY650
FY651
FY652
FY653
FY654
FY655
FY656
FY657
FY658
FY659
FY660
FY661
