# Supplementary figures and images for: Tunicamycin Potentiates Antifungal Drug Tolerance via Aneuploidy in Candida albicans
Source: mBio. 2021 Aug 31;12(4):e02272-21. doi: 10.1128/mBio.02272-21 (PMC8406271; doi:10.1128/mBio.02272-21)

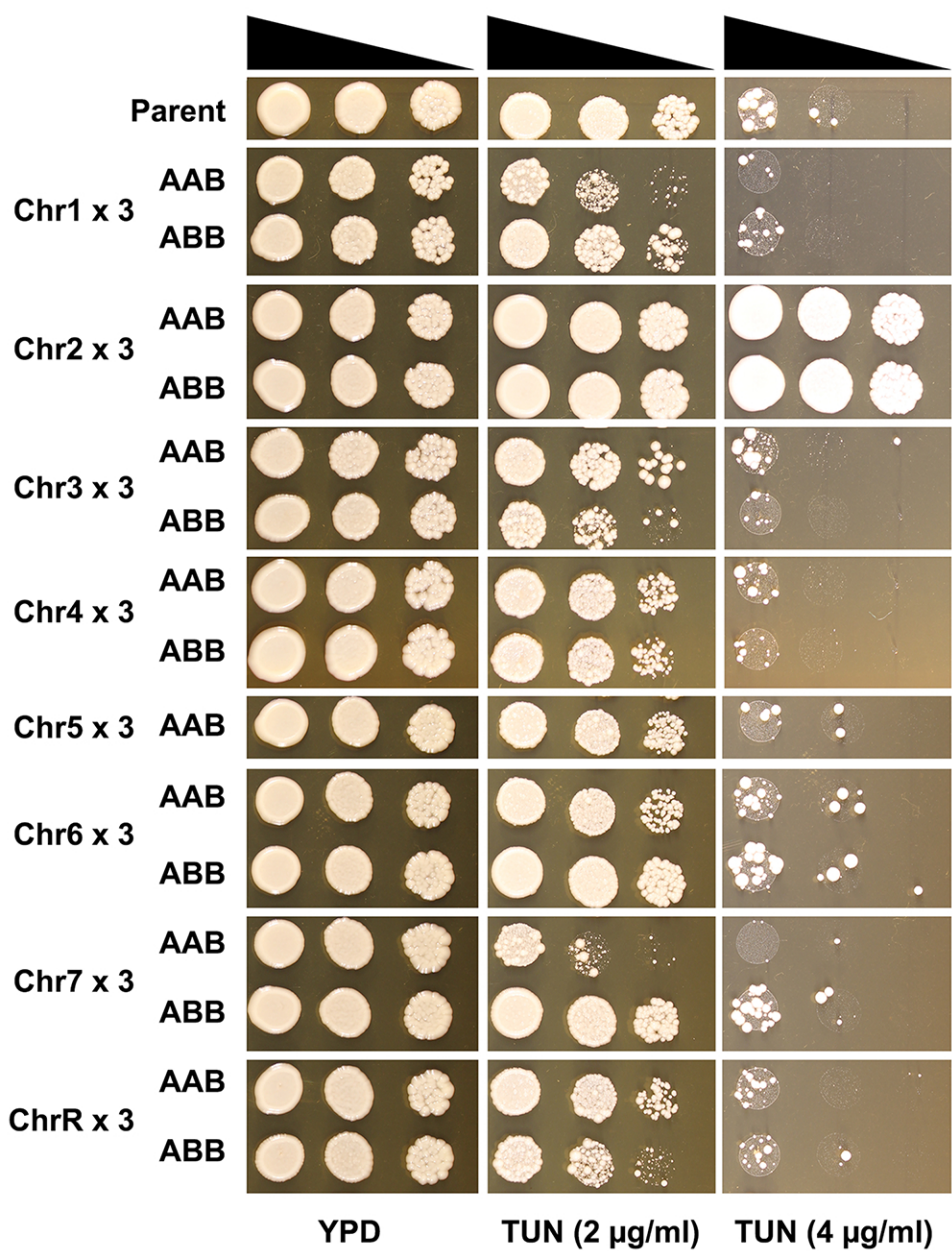

Supplement: FIG S3 [file mbio.02272-21-sf003.pdf]

**A**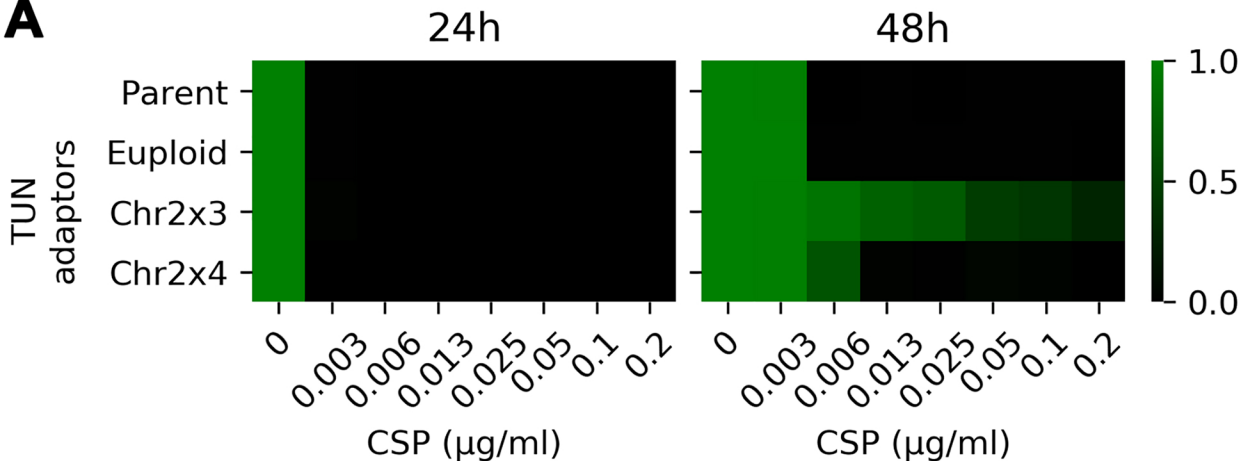**B**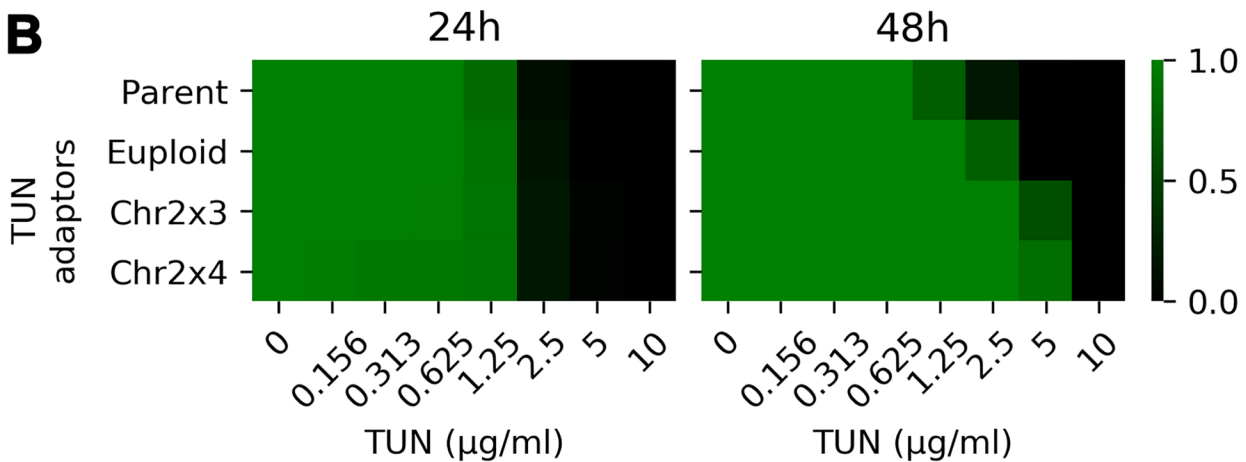

Supplement: FIG S4 [file mbio.02272-21-sf004.pdf]

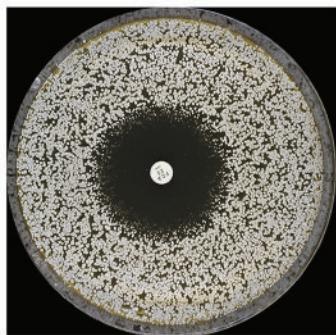

Parent

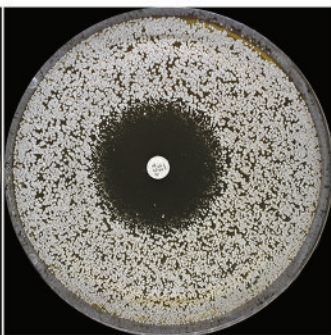

Euploid

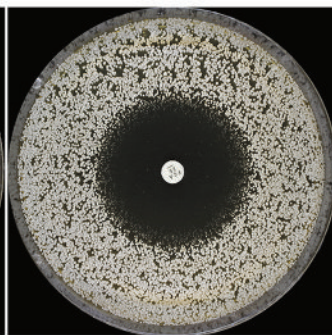

Chr2x3

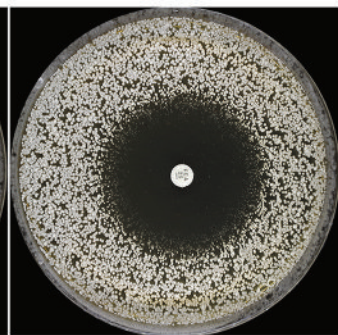

Chr2x4

---

TUN adaptors

Supplement: FIG S5 [file mbio.02272-21-sf005.pdf]

**A**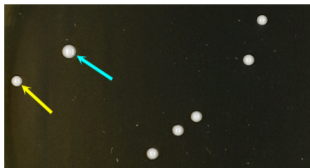**B**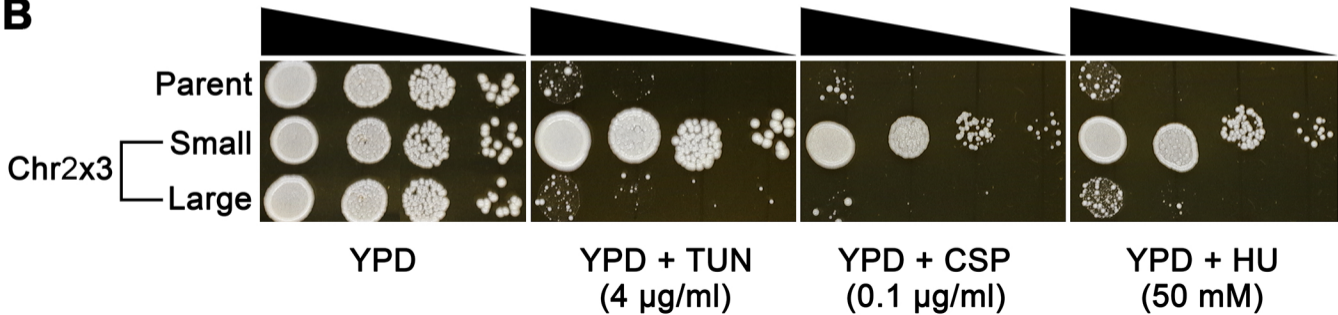

Supplement: FIG S6 [file mbio.02272-21-sf006.pdf]
