# Supplementary material for: Tunicamycin Potentiates Antifungal Drug Tolerance via Aneuploidy in Candida albicans
Source: mBio. 2021 Aug 31;12(4):e02272-21. doi: 10.1128/mBio.02272-21 (PMC8406271; doi:10.1128/mBio.02272-21)
Supplement: TABLE S3 [file mbio.02272-21-st003.pdf]

Table S3. Genes differentially expressed upon TUN treatment

	1 µg/ml treatment/control			4 µg/ml treatment/control		
**Gene**	**log ratio**	**q value**	**p value**	**log ratio**	**q value**	**p value**
C6_02310W_A	-0.31	0.03	0.01	-0.32	0.03	0.01
C7_03890C_A	-0.23	0.02	0.01	-0.06	0.64	0.51
C3_07290W_A	-0.33	0.04	0.02	-0.34	0.04	0.02
C6_03780C_A	0.04	0.82	0.71	0.32	0.02	0.01
C4_02330C_A	-0.4	0.2	0.1	-0.21	0.54	0.4
C1_09190C_A	0.01	0.87	0.78	-0.03	0.76	0.66
C4_06720W_A	-0.42	0	0	-1.13	0	0
C1_12930C_A	0.23	0.51	0.34	0.57	0.04	0.02
C3_03030C_A	-0.12	0.43	0.27	-1.13	0	0
C1_06400C_A	-0.24	0.04	0.02	-0.02	0.91	0.85
C2_09810C_A	1.14	0	0	1.66	0	0
C7_02380C_A	0.15	0.13	0.06	0.15	0.15	0.08
C3_01950C_A	-0.44	0	0	-0.76	0	0
C5_04380C_A	0	1	0.99	0.09	0.9	0.84
C1_00590W_A	0.19	0	0	-0.21	0.02	0.01
C2_07550W_A	0.52	0	0	0.52	0	0
C3_07900C_A	0.18	0.12	0.05	0.34	0.01	0
C3_03590W_A	0.14	0.11	0.05	0.19	0.08	0.04
C2_04870C_A	-0.08	0.5	0.34	-0.13	0.26	0.15
C3_06390W_A	NA	NA	NA	NA	NA	NA
CR_03070W_A	0.02	0.92	0.85	-0.16	0.17	0.09
C4_00560C_A	0.19	0.18	0.09	0.21	0.18	0.1
C1_13680C_A	-0.28	0	0	-0.51	0	0
C3_04810C_A	-0.04	0.64	0.48	-0.28	0	0
C2_07840W_A	-1.14	0	0	-2.06	0	0
C1_12180C_A	0.58	0	0	0.58	0	0
CR_10550W_A	-1.37	0	0	-1.38	0	0
C5_02770W_A	0.23	0.05	0.02	0.1	0.49	0.35
C1_04230W_A	-0.09	0.44	0.28	0.23	0.03	0.01
C3_03170W_A	0.65	0	0	1.04	0	0
C2_05830C_A	-0.38	0.13	0.06	-0.31	0.23	0.13
C4_05580C_A	0.07	0.67	0.52	NA	NA	NA
C3_00950C_A	-0.52	0.01	0	-0.14	0.61	0.48
C7_00970C_A	0.08	0.58	0.42	0.73	0	0
CR_03160W_A	-0.27	0.33	0.19	0.09	0.76	0.66
CR_00070W_A	0.47	0	0	0.49	0	0
C2_00550W_A	-0.38	0.09	0.04	-0.61	0.01	0
C3_04490W_A	-0.02	0.88	0.8	0.18	0.12	0.06
C2_05700W_A	0.56	0	0	0.51	0	0
C3_02040C_A	-1.17	0	0	-0.94	0	0

C3_04250W_A	0.25	0.4	0.25	0.61	0.01	0
CR_05800C_A	1.58	0	0	1.73	0	0
C4_06730C_A	0.26	0.02	0.01	0.26	0.03	0.01
C4_03320W_A	-0.36	0.03	0.01	0	1	0.99
C1_09780C_A	0.08	0.76	0.63	0	0.99	0.98
C1_08510W_A	-0.06	0.76	0.64	0.1	0.53	0.39
C3_05370C_A	-0.17	0	0	-0.54	0	0
C4_06480C_A	1.65	0	0	2.6	0	0
C3_02910W_A	0.03	0.94	0.88	-0.46	0.06	0.03
CR_00460C_A	-0.85	0.01	0	-0.45	0.21	0.12
C1_05850W_A	-0.14	0.33	0.19	-0.05	0.75	0.64
C2_04000C_A	-0.07	0.67	0.52	-0.13	0.65	0.52
CR_06910W_A	-0.23	0.16	0.08	0.26	0.15	0.08
C1_08910C_A	-0.13	0.47	0.31	-0.1	0.59	0.46
CR_03630W_A	0.02	0.94	0.88	-0.28	0.05	0.02
C2_09970C_A	0.12	0.43	0.27	0.19	0.18	0.1
C5_04900C_A	0.03	0.88	0.81	0.29	0.05	0.02
C5_01410C_A	0.17	0.15	0.07	-0.36	0	0
CR_03620C_A	0.16	0.07	0.03	0.16	0.1	0.05
C7_01690W_A	0	1	0.99	0.05	0.95	0.92
C7_00780W_A	-0.14	0.39	0.24	0.11	0.48	0.34
C2_09610W_A	-0.27	0.24	0.12	-0.05	0.86	0.79
C7_00460W_A	-0.04	0.94	0.89	0.09	0.84	0.78
C7_02070W_A	0.25	0.64	0.47	0.13	0.85	0.79
CR_03700C_A	-0.17	0.2	0.1	-0.35	0.01	0
C3_00490W_A	0.02	0.91	0.84	0.3	0.01	0
C3_00760W_A	-0.38	0	0	-1.28	0	0
C4_02860W_A	-0.09	0.51	0.35	0.04	0.81	0.73
C1_11310C_A	0.1	0.44	0.28	0.04	0.81	0.73
C1_13780W_A	0.61	0	0	0.65	0	0
C7_03970C_A	0.2	0.35	0.21	0.03	0.92	0.87
CR_08920W_A	0.12	0.61	0.44	0.37	0.07	0.03
C3_02500W_A	0.19	0.13	0.06	0.32	0.01	0
C4_06460C_A	0.04	0.84	0.75	0.67	0	0
C4_05320W_A	0.24	0.02	0.01	0.18	0.12	0.06
C2_05560W_A	0.18	0.2	0.1	0	1	0.99
C2_00930C_A	0.19	0.41	0.25	-0.04	0.89	0.83
C1_07590C_A	0.02	0.9	0.83	0.16	0.25	0.15
C4_04800W_A	0.04	0.73	0.59	-0.23	0.06	0.02
C4_04030W_A	NA	NA	NA	0.46	0.3	0.19
CR_04500C_A	-1.05	0	0	-1.71	0	0
C2_09740W_A	-0.11	0.47	0.31	-0.23	0.11	0.06
C3_06940W_A	0.12	0.35	0.2	0.28	0.01	0

C2_09400C_A	-0.58	0	0	-1.06	0	0
C2_02280W_A	-0.11	0.43	0.27	-0.4	0	0
C7_02320W_A	-0.01	0.94	0.9	0.23	0.05	0.02
C6_04570W_A	0.55	0	0	0.51	0	0
C1_08880W_A	0.13	0.13	0.06	-0.08	0.44	0.3
CR_08860W_A	-0.23	0.18	0.09	0.04	0.85	0.78
C2_07010W_A	0.05	0.59	0.42	-0.23	0.01	0
C4_03720C_A	-0.83	0	0	-1.65	0	0
C2_10580W_A	0	0.99	0.98	0.15	0.33	0.21
C2_04450W_A	0.29	0.48	0.32	0.37	0.38	0.25
C1_00930C_A	-0.06	0.62	0.46	-0.15	0.16	0.08
CR_02680W_A	-0.55	0.01	0	-0.54	0.03	0.01
C1_01870C_A	-0.47	0	0	-0.82	0	0
C3_07050W_A	-0.34	0.3	0.17	-0.46	0.16	0.08
C1_00820W_A	-0.29	0.14	0.06	-0.19	0.36	0.24
CR_08470W_A	-0.14	0.57	0.4	0.48	0.01	0
C4_05920C_A	0.47	0.03	0.01	0.35	0.14	0.08
C5_01840C_A	-0.09	0.67	0.51	0.23	0.14	0.07
CR_02460W_A	0.11	0.47	0.31	-0.14	0.37	0.24
C1_04590W_A	0.11	0.67	0.52	-0.15	0.52	0.38
C1_12670C_A	-0.33	0.25	0.13	0.07	0.83	0.75
CR_03030C_A	0.05	0.59	0.43	-0.48	0	0
C2_04100W_A	0.36	0.04	0.01	0.67	0	0
C4_06120W_A	0.16	0.02	0.01	-0.29	0	0
C5_03540C_A	0.03	0.71	0.57	-0.51	0	0
C5_03810C_A	-1.31	0	0	-1.25	0	0
C2_01370C_A	-0.53	0	0	-0.19	0.3	0.19
C4_02240C_A	0.07	0.67	0.52	0.26	0.49	0.35
C7_01940C_A	0.01	0.97	0.95	0.38	0	0
C2_00210W_A	-0.12	0.16	0.07	-0.55	0	0
C3_03840C_A	-0.03	0.88	0.79	0.12	0.39	0.26
C6_03990C_A	0.1	0.68	0.53	0.54	0	0
C3_03410C_A	-0.14	0.07	0.03	0	0.97	0.95
C2_08550C_A	-0.53	0	0	-0.65	0	0
C6_02640C_A	0.48	0	0	0.69	0	0
C1_06500W_A	0.16	0.43	0.27	0.33	0.03	0.01
BGI_novel_G000	-0.18	0.16	0.07	-0.23	0.07	0.03
CR_08660W_A	-0.05	0.7	0.55	-0.24	0.02	0.01
C2_09490W_A	0.08	0.77	0.64	0.42	0.05	0.02
C3_03690W_A	0.76	0.05	0.02	1.89	0	0
C4_03800C_A	-0.21	0.16	0.07	-0.07	0.69	0.57
C6_02990W_A	0.2	0.15	0.07	0.23	0.1	0.05
C4_04300C_A	-0.56	0	0	-0.71	0	0

C4_02820W_A	0.21	0.69	0.54	0.19	0.8	0.71
CR_07650W_A	0.05	0.69	0.55	-0.11	0.34	0.22
C2_07070W_A	-0.05	0.7	0.56	-0.21	0.08	0.04
C6_04430W_A	0.13	0.48	0.32	0.17	0.35	0.23
CR_10380C_A	0.69	0.13	0.06	1.27	0.01	0
C1_11440C_A	-0.63	0.04	0.01	-0.09	0.86	0.8
BGI_novel_G000(	0.08	0.63	0.47	-0.08	0.66	0.53
C5_00420C_A	-0.4	0.05	0.02	-0.19	0.35	0.23
CR_08300C_A	0.07	0.56	0.39	-0.49	0	0
C1_11710C_A	0	0.99	0.98	-0.14	0.78	0.68
C6_00570C_A	0.28	0.01	0	0.05	0.73	0.62
C1_08640W_A	-0.16	0.34	0.2	-0.12	0.5	0.36
C5_01880C_A	0.5	0	0	0.65	0	0
CR_01730W_A	0.77	0	0	1.4	0	0
C2_00450C_A	-0.08	0.65	0.49	0.01	0.97	0.95
C1_01390C_A	-0.1	0.55	0.38	0.38	0.01	0
C1_05900W_A	0.03	0.88	0.8	0.47	0	0
C5_04840C_A	-0.83	0	0	-0.42	0.18	0.1
C1_05210C_A	-0.25	0.02	0.01	-0.33	0	0
C5_03670C_A	0.32	0.2	0.1	0.15	0.61	0.48
C4_00440C_A	-0.68	0	0	-1.69	0	0
C1_13180W_A	-0.17	0.77	0.64	-0.28	0.68	0.56
C3_05700W_A	-0.16	0.09	0.04	0.09	0.4	0.27
C4_05760W_A	0.01	0.95	0.91	0.15	0.28	0.17
C6_04130C_A	0.84	0	0	-1.03	0	0
C5_05180W_A	0	0.99	0.99	0.34	0.53	0.39
C2_08080C_A	0.24	0.29	0.16	-0.01	0.97	0.94
C2_01710W_A	-0.35	0.27	0.15	-0.28	0.45	0.31
CR_01210C_A	0.39	0	0	0.77	0	0
CR_01990C_A	-0.38	0.05	0.02	-0.37	0.06	0.03
C4_02430W_A	0.04	0.79	0.68	0.08	0.65	0.51
C6_00950C_A	-0.35	0.01	0	-0.06	0.79	0.69
C1_07970C_A	-0.36	0.02	0	0	1	0.99
C2_00630C_A	0.63	0	0	0.86	0	0
C6_01330C_A	-0.03	0.9	0.82	-0.14	0.41	0.28
C7_04240C_A	-0.08	0.28	0.15	-0.13	0.12	0.06
CR_01310W_A	0.73	0	0	0.91	0	0
C1_05980W_A	0.19	0.33	0.19	0.45	0	0
CR_05890C_A	-0.47	0	0	-0.35	0.01	0
C2_08660C_A	0.04	0.93	0.87	-0.19	0.66	0.53
C1_03670C_A	0.39	0	0	0.62	0	0
C5_03150W_A	-0.16	0.21	0.11	-0.12	0.37	0.24
C6_00860W_A	0.09	0.83	0.73	0.29	0.45	0.31

C3_01090W_A	-0.1	0.87	0.78	-0.39	0.47	0.33
C2_06590C_A	0.06	0.89	0.81	-0.12	0.79	0.7
C4_05010W_A	-0.26	0.11	0.04	-0.32	0.04	0.02
C1_10080W_A	-0.2	0.61	0.44	0.18	0.65	0.52
CR_06640C_A	-0.31	0.06	0.02	-0.19	0.25	0.15
C3_07180C_A	-0.68	0	0	-1.02	0	0
C1_07890C_A	-0.71	0	0	-1.57	0	0
C2_00540W_A	0.33	0.02	0	0.12	0.46	0.33
C1_03080C_A	-0.15	0.61	0.45	-0.14	0.58	0.45
C2_04740C_A	0.02	0.96	0.93	0.43	0.18	0.1
C1_10160W_A	0.32	0	0	0.33	0	0
C3_06990W_A	0.18	0.42	0.26	-0.06	0.83	0.75
C7_02640W_A	0.08	0.37	0.22	-0.12	0.22	0.13
C1_03060C_A	-0.46	0.01	0	-0.43	0.02	0.01
C1_04930C_A	1.38	0	0	1.79	0	0
C1_11610C_A	-0.79	0	0	-0.84	0	0
C1_05350W_A	-0.06	0.78	0.66	-0.21	0.27	0.16
CR_06690C_A	-0.1	0.5	0.34	-0.2	0.13	0.07
C3_04560W_A	-0.96	0	0	-1.65	0	0
C4_01510W_A	0.25	0.01	0	0.45	0	0
C2_00270C_A	0.04	0.82	0.71	-0.15	0.34	0.22
CR_07310W_A	-0.45	0.01	0	-0.56	0	0
C2_09290W_A	-0.23	0.47	0.3	-0.27	0.4	0.27
C1_00540C_A	0.07	0.66	0.5	0.24	0.06	0.03
C4_01020C_A	0.04	0.76	0.63	0.05	0.68	0.55
C1_07050C_A	0.09	0.64	0.48	0.15	0.37	0.24
C2_02070W_A	0.14	0.76	0.63	0.49	0.45	0.32
C3_07600W_A	-0.26	0.26	0.14	-0.26	0.25	0.15
C4_07080C_A	-0.63	0	0	-0.73	0	0
C2_01670C_A	0.97	0	0	1	0	0
C6_00690W_A	-0.22	0.25	0.13	-0.07	0.75	0.64
C5_00610C_A	-0.05	0.82	0.71	0.16	0.37	0.25
C5_02600W_A	-0.44	0.22	0.11	-0.21	0.6	0.47
C1_00340W_A	-0.47	0	0	-0.37	0	0
C5_02340C_A	-0.08	0.62	0.46	-0.26	0.09	0.04
CR_05100W_A	-0.15	0.48	0.31	0.19	0.36	0.24
C1_14340C_A	0.36	0	0	0.25	0.04	0.02
C3_06610W_A	0.38	0	0	0.58	0	0
C6_00640C_A	-0.04	0.82	0.71	0.01	0.96	0.93
CR_08580C_A	-0.5	0.05	0.02	-0.06	0.85	0.78
C2_05720C_A	-0.03	0.79	0.67	0.14	0.13	0.07
C2_08740W_A	-0.82	0	0	-0.42	0.06	0.03
C1_08240C_A	-0.02	0.89	0.82	-0.34	0.01	0

CR_07770C_A	-0.17	0.26	0.14	-0.1	0.55	0.41
C1_12860C_A	-0.14	0.63	0.47	-0.31	0.23	0.13
C6_00820W_A	0.3	0	0	-0.01	0.91	0.87
CR_01360W_A	-0.31	0.13	0.06	-0.57	0	0
C7_01050W_A	0.04	0.94	0.88	0.22	0.62	0.49
C1_00890W_A	-0.2	0.11	0.05	-0.26	0.04	0.02
C1_00990C_A	-0.28	0.03	0.01	-0.27	0.06	0.03
C1_06320W_A	-0.1	0.74	0.61	-0.4	0.15	0.08
C6_03130W_A	0.32	0.1	0.04	0.65	0	0
C3_03000W_A	0.2	0.65	0.49	-0.35	0.45	0.32
C2_01030W_A	-0.23	0	0	-0.35	0	0
C2_02010C_A	1.03	0	0	1.53	0	0
C5_00760W_A	-0.36	0.07	0.03	-0.08	0.74	0.63
C6_00910C_A	0.32	0	0	0.53	0	0
C1_03150C_A	0.24	0.09	0.03	0.49	0	0
C1_00980W_A	-0.33	0.01	0	-0.45	0	0
C2_07980W_A	0.26	0.04	0.02	0.37	0	0
C1_13130C_A	-0.26	0.13	0.05	-0.15	0.43	0.3
C6_02510C_A	-0.13	0.77	0.65	-0.58	0.29	0.18
C5_02430W_A	0.18	0.66	0.5	0.16	0.69	0.57
C1_14420W_A	0.68	0	0	0.87	0	0
C6_02790C_A	0.21	0.37	0.22	0.06	0.81	0.72
C1_03990W_A	-0.15	0.53	0.36	0.49	0	0
C4_04930C_A	-0.33	0.22	0.11	0.02	0.94	0.91
C1_08950W_A	-0.07	0.57	0.41	-0.05	0.77	0.67
C1_09120W_A	-0.22	0.33	0.19	-0.21	0.35	0.23
CR_06720W_A	-0.69	0	0	-0.68	0	0
C2_01900C_A	0.28	0.1	0.04	0.47	0.01	0
C2_10820C_A	-0.21	0.1	0.04	-0.47	0	0
CR_09070C_A	0.05	0.72	0.59	-0.72	0	0
C3_02450W_A	0.22	0.42	0.26	0.25	0.35	0.23
CR_02360W_A	-0.04	0.88	0.8	0.07	0.79	0.7
C4_00460C_A	0.08	0.56	0.39	0.11	0.43	0.29
C5_03920C_A	-0.34	0.21	0.1	-0.46	0.06	0.03
C1_14270W_A	0.15	0.5	0.34	0.1	0.69	0.57
C4_00780C_A	0.03	0.86	0.77	-0.15	0.25	0.15
C1_01790W_A	-0.11	0.37	0.22	0.02	0.91	0.86
C1_11730W_A	-0.11	0.4	0.24	0.14	0.26	0.16
C5_05350W_A	0.07	0.65	0.49	0.01	0.97	0.95
C6_01210W_A	-0.19	0.16	0.07	-0.2	0.16	0.08
C6_02890C_A	-0.06	0.93	0.88	0.52	0.39	0.27
C7_00150W_A	0.36	0	0	0.48	0	0
C2_07430C_A	0.13	0.48	0.31	0.18	0.28	0.17

C1_08710W_A	0.06	0.83	0.73	0.18	0.45	0.32
C3_05110W_A	-0.22	0.3	0.17	0.07	0.75	0.64
CR_02050C_A	-0.28	0.03	0.01	-0.18	0.2	0.12
C3_00530C_A	-0.16	0.45	0.29	0.35	0.04	0.01
C6_03370W_A	0.62	0	0	0.68	0	0
CR_10010C_A	0.03	0.79	0.67	-0.18	0.06	0.03
C4_05360C_A	-0.19	0.46	0.3	-0.07	0.81	0.72
CR_10200W_A	0.05	0.92	0.85	-0.48	0.16	0.08
C3_04970C_A	0.37	0	0	0.41	0	0
CR_07150W_A	-0.18	0.01	0	-0.06	0.58	0.45
CR_02510W_A	-0.11	0.55	0.39	-0.3	0.11	0.05
C1_04180W_A	-0.05	0.66	0.5	-0.62	0	0
C1_04870W_A	0.42	0.38	0.23	0.47	0.41	0.28
C2_08430C_A	0.18	0.68	0.53	0.53	0.4	0.27
C2_10600W_A	-0.06	0.93	0.87	0.48	0.43	0.3
C4_04750W_A	0.09	0.58	0.42	0.25	0.09	0.04
C4_06000W_A	0.13	0.48	0.31	0.17	0.35	0.23
CR_01810C_A	-0.25	0.5	0.34	-0.56	0.12	0.06
C1_05280W_A	0.1	0.62	0.46	-0.05	0.8	0.71
C6_03940C_A	0.03	0.8	0.68	-0.06	0.6	0.46
C2_02050C_A	-1.25	0	0	-1.27	0	0
CR_08090W_A	0.07	0.47	0.31	-0.59	0	0
C2_06820C_A	0.12	0.2	0.1	0.29	0	0
C6_04110W_A	-0.02	0.92	0.85	-0.18	0.27	0.16
C6_00490W_A	-0.14	0.63	0.47	0.12	0.65	0.52
C1_03960C_A	-0.31	0.11	0.05	0.01	0.99	0.98
C1_00170W_A	0.63	0	0	0.53	0	0
C2_06010W_A	0.27	0.02	0.01	0.42	0	0
C4_07060W_A	-0.14	0.18	0.09	-0.29	0	0
C7_01590C_A	0	0.98	0.97	-0.11	0.49	0.35
C2_00070C_A	-0.74	0	0	-0.65	0	0
C3_02680C_A	-0.48	0	0	-0.59	0	0
C4_02780W_A	0.07	0.47	0.3	0.1	0.3	0.19
C1_06620C_A	1.19	0	0	2.12	0	0
C2_09180W_A	0	1	0.99	0.14	0.28	0.17
C2_04380C_A	-0.11	0.42	0.26	0.13	0.31	0.19
C1_13450W_A	-0.02	0.95	0.9	0.08	0.78	0.68
C7_03880C_A	0.04	0.89	0.81	0.57	0.01	0
C1_07500C_A	-0.05	0.76	0.62	-0.28	0.03	0.01
C3_04140C_A	-0.15	0.07	0.03	-0.28	0	0
C6_00460C_A	-0.17	0.51	0.34	-0.17	0.5	0.36
C7_00230W_A	-0.44	0	0	-0.99	0	0
C1_11580W_A	-0.2	0.19	0.09	-0.51	0	0

C5_02730C_A	-0.11	0.44	0.27	-0.07	0.67	0.55
C7_01410C_A	0.07	0.81	0.69	0.27	0.27	0.16
C1_11380W_A	-0.31	0.07	0.03	-0.24	0.17	0.09
C5_00380W_A	-0.25	0.28	0.15	-0.26	0.25	0.15
CR_05550C_A	-0.2	0.1	0.04	-0.38	0	0
C2_10680W_A	-0.15	0.24	0.12	-0.5	0	0
C6_04650W_A	0.08	0.39	0.23	-0.75	0	0
CR_04450C_A	-0.23	0.02	0.01	-0.17	0.15	0.08
C2_02400W_A	0.11	0.41	0.26	-0.53	0	0
C1_08340C_A	0.2	0.34	0.2	0.32	0.11	0.05
CR_01600C_A	-0.29	0.29	0.16	0.17	0.56	0.42
C7_04120W_A	0.68	0	0	0.74	0	0
C5_05430W_A	0.08	0.82	0.71	0.03	0.94	0.91
C4_00290C_A	0.49	0.28	0.15	0.99	0.03	0.01
C4_03950C_A	-0.6	0.19	0.09	-1.42	0.01	0
C4_05820W_A	0.36	0.2	0.09	0.37	0.19	0.1
C2_09340W_A	0	1	0.99	-0.13	0.65	0.52
C1_03290W_A	-0.2	0.18	0.09	0.08	0.63	0.5
C4_04590W_A	0.12	0.09	0.04	0.01	0.93	0.88
C3_03530W_A	-0.22	0.66	0.5	-0.2	0.71	0.6
C1_01930W_A	-0.03	0.77	0.64	0.34	0	0
C4_01830C_A	-0.15	0.33	0.19	-1.23	0	0
CR_04080C_A	0.98	0	0	0.73	0	0
CR_04770C_A	-1.11	0	0	-0.42	0.07	0.03
C6_00170C_A	-0.01	0.93	0.87	0.14	0.12	0.06
C2_09110C_A	-0.19	0.7	0.55	0.04	0.96	0.93
C6_00310W_A	0.41	0	0	0.22	0.04	0.02
C5_01740C_A	0.01	0.99	0.98	0.41	0.04	0.02
C7_04210C_A	-0.1	0.22	0.11	-0.37	0	0
C1_04670W_A	0.01	0.97	0.94	-0.01	0.98	0.96
C1_07000W_A	-0.36	0	0	-0.61	0	0
C1_09520C_A	-0.73	0	0	-1.35	0	0
C2_09660W_A	-0.98	0	0	-0.63	0.03	0.01
C7_03930C_A	0.08	0.42	0.26	-0.13	0.19	0.11
C1_12380C_A	-0.01	0.94	0.89	-0.3	0	0
C2_00520W_A	0.29	0.01	0	-0.03	0.83	0.76
C2_01930C_A	-0.34	0.01	0	-0.39	0.01	0
C3_04060C_A	0.58	0	0	0.31	0	0
C6_04160C_A	-1.67	0	0	-1.45	0	0
BGI_novel_G000	-0.19	0.24	0.12	-0.33	0.03	0.01
C3_01440C_A	-0.28	0.5	0.33	0.04	0.93	0.89
C2_05960C_A	-0.42	0.27	0.14	-0.08	0.86	0.8
C4_04620C_A	0.27	0.12	0.05	-0.02	0.92	0.87

CR_00810W_A	-0.46	0.29	0.16	-0.11	0.83	0.76
C3_01610W_A	0.6	0	0	0.69	0	0
C6_00280W_A	-0.02	0.91	0.84	-0.25	0.01	0
C5_00080C_A	0.62	0	0	0.79	0	0
C1_09340C_A	-0.01	0.97	0.93	0.45	0.02	0.01
C5_03100C_A	-0.32	0.04	0.01	-0.12	0.51	0.37
C2_00050C_A	-0.19	0.44	0.27	-0.12	0.82	0.74
CR_00910W_A	-0.03	0.93	0.87	0.62	0	0
C2_01820C_A	-0.42	0.06	0.02	-0.39	0.08	0.04
C7_00660W_A	-0.15	0.12	0.05	0.1	0.38	0.26
C7_02790C_A	0	0.99	0.98	0.04	0.74	0.63
CR_03810W_A	0.11	0.28	0.15	0.19	0.05	0.02
C2_08620W_A	0.14	0.23	0.11	0.8	0	0
CR_06940W_A	-0.1	0.73	0.59	0.1	0.72	0.61
CR_06090W_A	0.03	0.84	0.74	0.16	0.25	0.15
C2_06610C_A	NA	NA	NA	0.13	0.66	0.53
C1_04540C_A	-0.07	0.65	0.49	0.04	0.8	0.71
C7_02680W_A	-0.19	0.11	0.05	-0.29	0	0
C1_00690W_A	0.32	0.02	0.01	0.21	0.19	0.1
C6_01280W_A	0.08	0.68	0.53	0.25	0.17	0.09
CR_00880W_A	0.42	0.02	0.01	0.52	0	0
C4_06610C_A	0.33	0	0	0.06	0.57	0.44
C2_02840C_A	0.18	0.18	0.08	0.12	0.42	0.29
C3_00690C_A	-0.09	0.61	0.44	-0.14	0.42	0.29
C5_04070C_A	0.08	0.8	0.68	0.12	0.69	0.57
C2_01500W_A	-0.1	0.85	0.75	-0.26	0.63	0.5
C5_01600C_A	-0.82	0.01	0	-0.43	0.2	0.11
C7_01730C_A	0.43	0.21	0.1	0.61	0.05	0.02
C1_08680C_A	0	1	1	0.16	0.28	0.17
C1_01340C_A	0.03	0.94	0.88	0.47	0.02	0.01
C7_03170W_A	0.02	0.95	0.91	0.09	0.66	0.53
C1_06920C_A	0.21	0.24	0.12	0.3	0.05	0.02
C1_05800C_A	-0.26	0.37	0.22	-0.07	0.84	0.77
C1_04680W_A	-0.32	0	0	-0.85	0	0
C2_00190C_A	0.05	0.7	0.56	-0.49	0	0
C2_10080W_A	-0.09	0.62	0.46	-0.32	0.04	0.02
C2_10860C_A	0.23	0	0	0.18	0.1	0.05
C3_02510C_A	0.47	0.15	0.07	-0.1	0.81	0.72
C5_04580C_A	-0.29	0.12	0.05	-0.16	0.42	0.29
C1_04630C_A	0.59	0	0	0.77	0	0
C2_05260W_A	0.12	0.37	0.22	0.6	0	0
C1_06690W_A	-0.68	0.02	0	-0.59	0.06	0.03
C1_08080C_A	0.08	0.6	0.44	0.16	0.26	0.16

C3_02790W_A	1.97	0	0	2.85	0	0
C2_09730C_A	-0.06	0.77	0.65	-0.06	0.78	0.68
C3_07420W_A	-0.21	0.02	0	-0.32	0	0
C4_00220C_A	0.36	0	0	0.2	0.04	0.01
C3_03980C_A	0.01	0.99	0.98	-0.39	0.3	0.18
C2_08780W_A	0.26	0	0	0.36	0	0
C6_00720C_A	0	0.99	0.98	-0.4	0	0
C4_06360C_A	0.52	0.26	0.14	0.23	0.72	0.61
C7_04090C_A	0.6	0	0	0.62	0	0
C1_01170C_A	-0.35	0.04	0.01	-0.33	0.07	0.03
CR_05170C_A	0.38	0.34	0.19	0.56	0.16	0.09
C5_00140C_A	0.23	0.35	0.21	-0.12	0.69	0.57
C1_02690C_A	NA	NA	NA	0.26	0.49	0.35
C7_01670W_A	-0.18	0.57	0.4	0.03	0.92	0.87
C7_03350C_A	-0.21	0.17	0.08	-0.56	0	0
C6_00090W_A	-0.27	0.07	0.03	-0.53	0	0
C4_05750C_A	-0.19	0.38	0.23	-0.26	0.2	0.11
C2_02500W_A	-0.25	0.07	0.03	-0.05	0.78	0.69
C1_08840W_A	-0.11	0.78	0.66	-0.25	0.51	0.37
C2_06740W_A	-0.15	0.42	0.26	-0.08	0.73	0.62
C3_07840C_A	NA	NA	NA	NA	NA	NA
C5_00330C_A	0.02	0.91	0.85	-0.05	0.77	0.67
C1_02370C_A	-0.16	0.22	0.11	0.25	0.11	0.05
C5_01920C_A	0.24	0.13	0.06	0.21	0.19	0.1
C2_07320W_A	0.33	0	0	0.4	0	0
C1_08520C_A	-0.07	0.46	0.3	-0.32	0	0
C2_03720W_A	NA	NA	NA	NA	NA	NA
C5_02210W_A	0.02	0.87	0.78	0.28	0	0
CR_03120W_A	0.15	0.15	0.07	0.1	0.36	0.24
CR_04460C_A	0.32	0.12	0.05	0.45	0.02	0.01
C5_02830W_A	-0.15	0.36	0.21	0	0.99	0.98
CR_09100C_A	0.24	0.1	0.04	0.73	0	0
CR_03170W_A	0.18	0.02	0	-0.08	0.42	0.29
C1_06600W_A	0.11	0.24	0.12	0.11	0.24	0.14
C4_01230C_A	0.81	0.02	0.01	1.51	0	0
C3_03310C_A	-0.13	0.58	0.41	0.06	0.79	0.7
C2_08420W_A	0.11	0.6	0.44	0.17	0.42	0.29
C1_08560W_A	0.18	0.03	0.01	-0.1	0.38	0.25
C1_11420W_A	-1.4	0	0	-1.26	0	0
C6_03020W_A	0.3	0.02	0.01	0.31	0.03	0.01
C6_03700W_A	0.6	0.01	0	-0.14	0.65	0.53
C1_01600W_A	-0.49	0	0	-0.36	0.01	0
C6_03140C_A	0.04	0.83	0.73	0.14	0.32	0.2

C7_02950C_A	-0.34	0.01	0	-0.08	0.62	0.49
C1_00110W_A	0.06	0.59	0.42	-0.79	0	0
C1_03460C_A	0.23	0.2	0.09	0.93	0	0
C1_09640W_A	-0.24	0	0	-0.58	0	0
C5_04550W_A	0.01	0.94	0.88	0.11	0.4	0.27
C3_03090W_A	-0.22	0.29	0.16	-0.09	0.65	0.52
C5_00690C_A	-0.57	0.02	0.01	-0.17	0.59	0.46
C7_00220W_A	0.12	0.6	0.43	0.11	0.65	0.52
CR_01100C_A	-0.05	0.75	0.62	-0.05	0.76	0.66
C2_03390C_A	-0.24	0.05	0.02	-0.2	0.09	0.04
CR_09660W_A	-0.67	0	0	-0.39	0.14	0.07
C1_12240C_A	0.25	0.03	0.01	0.32	0	0
C7_00320C_A	0.4	0	0	-0.01	0.93	0.88
C4_01460C_A	0.06	0.77	0.65	0.19	0.21	0.12
C3_00290W_A	0.16	0.01	0	0.32	0	0
C3_01710C_A	-0.38	0.11	0.04	-0.1	0.74	0.63
C5_01270W_A	0.18	0.22	0.11	0.37	0.01	0
C1_06080C_A	0.2	0.04	0.01	0.53	0	0
C4_01390W_A	-0.4	0.3	0.17	-0.13	0.76	0.66
C3_06370C_A	-0.85	0	0	-0.81	0	0
C1_02910C_A	0.07	0.77	0.65	0.44	0.02	0.01
C6_04210C_A	0.04	0.84	0.74	0.05	0.78	0.68
CR_10810C_A	0.64	0	0	0.74	0	0
CR_05700C_A	0.35	0	0	0.43	0	0
C6_03620C_A	-0.11	0.4	0.24	-0.33	0.01	0
C3_07060W_A	0.14	0.28	0.15	0.57	0	0
C2_06370C_A	0.65	0	0	1.23	0	0
CR_09320C_A	0.05	0.67	0.51	-0.12	0.31	0.19
C7_03700C_A	-0.96	0	0	-0.7	0	0
CR_00310C_A	1.04	0	0	1.15	0	0
C6_04280W_A	0.06	0.57	0.41	0.03	0.79	0.69
CR_04860C_A	-0.64	0.08	0.03	-1.19	0	0
C2_05160C_A	-0.76	0	0	-1.36	0	0
C2_09350W_A	-0.05	0.86	0.78	-0.05	0.86	0.79
C1_02110C_A	0	1	0.99	0.07	0.91	0.86
C2_07920W_A	-0.09	0.66	0.5	-0.2	0.26	0.15
C1_09750W_A	0.01	0.94	0.9	-0.32	0.03	0.01
C4_06150C_A	1.39	0	0	3.12	0	0
C6_02610C_A	-0.11	0.32	0.18	-0.07	0.59	0.46
C4_00810C_A	-0.62	0	0	-0.24	0.26	0.15
C7_02270W_A	NA	NA	NA	NA	NA	NA
C6_02170C_A	-0.07	0.77	0.65	-0.12	0.61	0.48
C2_06330C_A	0.28	0.07	0.03	0.02	0.91	0.86

C1_01900C_A	0.17	0.18	0.09	0.09	0.56	0.42
C2_06320W_A	0.41	0	0	0.72	0	0
CR_10170C_A	-0.3	0.13	0.06	-0.26	0.2	0.11
CR_00380W_A	0.54	0.01	0	1.27	0	0
C5_02650C_A	-0.52	0	0	-0.3	0.08	0.04
C4_01890C_A	0.2	0.27	0.15	0.38	0.02	0.01
C7_00570W_A	-0.8	0	0	-1.56	0	0
C2_02850W_A	0.54	0.04	0.01	0.67	0.01	0
C2_04270W_A	0.46	0	0	0.46	0	0
C6_04410C_A	0.08	0.73	0.6	-0.35	0.09	0.04
C5_01810W_A	0.23	0.22	0.11	0.02	0.94	0.91
C2_04520C_A	-0.07	0.86	0.77	-0.15	0.68	0.56
CR_01770C_A	0.21	0.03	0.01	0.4	0	0
C1_09300C_A	0.06	0.9	0.83	-0.03	0.97	0.95
C1_03320C_A	0.49	0	0	0.71	0	0
C7_00270W_A	0.05	0.82	0.71	-0.42	0	0
C7_02930C_A	-0.08	0.56	0.4	-0.16	0.22	0.13
C1_11160C_A	0.27	0.09	0.04	0.17	0.33	0.21
C6_02020C_A	-0.13	0.55	0.38	-0.11	0.6	0.47
C6_01070C_A	-0.27	0.2	0.1	-0.06	0.81	0.72
C5_02910C_A	-0.51	0.02	0.01	-0.64	0	0
C1_03040W_A	0.54	0	0	0.94	0	0
C3_01250W_A	0.4	0	0	0.5	0	0
C1_02930C_A	0.12	0.32	0.18	-0.21	0.09	0.04
C2_10590W_A	-0.21	0.02	0.01	-0.07	0.55	0.42
C6_01200W_A	-0.26	0.47	0.3	-0.16	0.67	0.55
C1_09930W_A	0.38	0.36	0.21	0.61	0.14	0.07
C1_10470W_A	-0.11	0.22	0.11	-0.36	0	0
C7_00190W_A	-0.39	0	0	-0.36	0	0
CR_10070C_A	0.4	0	0	0.66	0	0
C1_10690W_A	0.15	0.3	0.17	0.06	0.75	0.65
C3_03680W_A	-0.25	0.01	0	-0.37	0	0
CR_09980W_A	0	1	0.99	0.11	0.51	0.37
CR_02520W_A	-0.62	0.01	0	-0.77	0	0
C1_01670C_A	-0.21	0.21	0.1	-0.22	0.2	0.11
C2_10540W_A	0.71	0	0	0.81	0	0
C7_01480W_A	-0.14	0.43	0.27	-0.05	0.81	0.72
C6_01820C_A	-0.09	0.57	0.4	0.05	0.76	0.66
C6_00660C_A	-0.03	0.87	0.79	-0.1	0.52	0.38
C6_03480W_A	-0.48	0.06	0.02	-0.3	0.25	0.15
C3_00910W_A	-0.14	0.67	0.52	0.02	0.95	0.91
C6_01990W_A	0.62	0	0	-0.21	0.04	0.02
C1_08300W_A	-0.01	0.93	0.87	0.16	0.16	0.08

CR_04170W_A	-0.77	0	0	-1.4	0	0
C1_10310W_A	-0.11	0.75	0.61	0.13	0.65	0.52
C2_06150C_A	0.27	0	0	0.46	0	0
C2_08270C_A	-0.42	0.01	0	-0.34	0.07	0.03
C5_03270W_A	-0.22	0.68	0.53	0.02	0.98	0.96
C1_09000W_A	-0.42	0.01	0	-0.47	0.01	0
C1_04610W_A	0.73	0	0	1.02	0	0
CR_10160W_A	0.07	0.4	0.24	-0.03	0.79	0.7
C1_12430W_A	0	1	0.99	0.14	0.36	0.24
CR_09020C_A	0.45	0	0	1.1	0	0
C2_04350C_A	-0.37	0	0	-0.6	0	0
C1_03690W_A	0.32	0	0	0.27	0.01	0
CR_00110W_A	0.45	0	0	0.43	0	0
C3_02990C_A	0.33	0.03	0.01	-0.14	0.45	0.31
CR_07250C_A	0.3	0.26	0.14	0.52	0.03	0.01
C1_13710C_A	-0.13	0.7	0.56	0.11	0.72	0.61
C3_00600W_A	1.66	0	0	0.46	0.17	0.09
C2_08610W_A	-0.4	0	0	-0.62	0	0
C3_07910W_A	-0.19	0.67	0.51	-0.32	0.46	0.33
C1_10840C_A	-0.27	0	0	-0.24	0.01	0
C4_03520C_A	0.63	0	0	0	1	1
C5_02990W_A	0.16	0.55	0.39	-0.01	0.98	0.96
CR_01480W_A	-0.36	0	0	-0.36	0	0
CR_07120C_A	-0.73	0	0	-1.14	0	0
C2_02660W_A	-0.45	0	0	-0.99	0	0
C3_06190C_A	-0.01	0.96	0.92	-0.16	0.39	0.26
C4_03880W_A	-0.49	0.14	0.06	0.28	0.47	0.34
C6_03090W_A	0.22	0.69	0.55	-0.01	0.99	0.99
C4_06250C_A	-0.15	0.22	0.11	-0.01	0.96	0.93
C1_03220C_A	0.32	0	0	0.22	0.03	0.01
C1_07100C_A	0.25	0.32	0.18	0.42	0.07	0.03
C3_02180C_A	-0.16	0.1	0.04	-0.46	0	0
C2_10240W_A	0.43	0	0	0.07	0.61	0.47
C6_02250W_A	-0.52	0.01	0	0.11	0.64	0.51
C2_08240W_A	-0.13	0.22	0.11	-0.63	0	0
C5_03590W_A	-0.01	0.95	0.91	0.05	0.74	0.64
CR_02040W_A	0.47	0	0	0.71	0	0
C4_06870W_A	0.33	0	0	0.5	0	0
C2_00300C_A	-0.05	0.76	0.63	-0.07	0.66	0.53
C3_03900C_A	-0.1	0.28	0.15	-0.79	0	0
C1_10650W_A	-0.27	0.24	0.12	0.26	0.21	0.12
C1_06750W_A	-0.22	0.48	0.31	0.15	0.65	0.52
C1_06900C_A	-0.19	0.05	0.02	-0.39	0	0

CR_10770W_A	0.03	0.91	0.84	0.04	0.86	0.8
C1_07210C_A	0.07	0.67	0.51	-0.35	0.01	0
C2_04810W_A	0.35	0.49	0.33	0.58	0.29	0.18
C3_03480C_A	0.28	0.01	0	0.36	0	0
C5_00270W_A	-0.42	0	0	-0.86	0	0
C3_05060W_A	0.1	0.73	0.59	0.35	0.12	0.06
C2_02620W_A	0.11	0.48	0.32	0.33	0.01	0
C7_00050C_A	0.38	0	0	0.47	0	0
C3_06200C_A	0.07	0.73	0.59	0.1	0.6	0.47
C7_02000C_A	-1.57	0	0	-2.12	0	0
C3_00050C_A	0.36	0.47	0.31	0.66	0.21	0.12
CR_03000C_A	0.15	0.28	0.15	0.62	0	0
C3_02940C_A	0.34	0	0	0.4	0	0
C4_05090C_A	0.25	0.02	0.01	0.25	0.03	0.01
C1_01250W_A	0.14	0.12	0.05	0	0.98	0.96
C1_08400C_A	-1.65	0	0	-2.92	0	0
C5_02550C_A	-0.8	0	0	-0.81	0	0
C1_09590C_A	-0.07	0.68	0.53	0	1	0.99
C3_04930C_A	0.12	0.68	0.53	0.33	0.2	0.11
C6_01730W_A	NA	NA	NA	NA	NA	NA
C1_09160W_A	-0.51	0.01	0	-0.53	0.01	0
C7_01230C_A	1.06	0	0	1.27	0	0
C2_03110W_A	-1.48	0	0	-2.73	0	0
C1_13900C_A	-0.09	0.53	0.36	-0.44	0	0
C1_13760W_A	0.51	0	0	0.74	0	0
C6_00200C_A	0.3	0	0	0.27	0.03	0.01
C3_01790C_A	0.22	0.16	0.07	0.3	0.05	0.02
C2_06080C_A	0.02	0.91	0.85	0.08	0.57	0.43
C3_06890W_A	0.98	0	0	0.02	0.85	0.78
C6_01590W_A	-0.19	0.15	0.07	-0.21	0.12	0.06
C2_03550C_A	0	0.98	0.97	0.03	0.84	0.76
C7_00610C_A	0.28	0.01	0	0.07	0.65	0.52
C4_01170C_A	-0.04	0.96	0.92	-0.4	0.5	0.36
C5_03250W_A	-0.8	0	0	-0.87	0	0
CR_08890C_A	-1.87	0	0	-1.94	0	0
C2_02820C_A	0.12	0.61	0.45	0.25	0.24	0.14
C3_01880W_A	0.35	0.02	0.01	0.33	0.04	0.02
C6_03440W_A	-0.54	0	0	-0.33	0.03	0.01
C5_04650C_A	-0.04	0.8	0.68	-0.12	0.41	0.28
CR_03780C_A	-0.11	0.79	0.67	0.31	0.31	0.2
C6_02650C_A	-0.85	0	0	-1.15	0	0
C4_02470C_A	0.08	0.5	0.34	0.43	0	0
C5_05370C_A	-0.01	0.98	0.95	-0.05	0.76	0.66

C1_04080W_A	-0.16	0.62	0.46	-0.29	0.35	0.23
C4_00960W_A	-0.47	0.03	0.01	-0.87	0	0
C2_03170W_A	-0.35	0.06	0.02	-0.46	0.01	0
BGI_novel_G000(	0.22	0.07	0.02	0.12	0.37	0.24
C4_02720C_A	-0.01	0.98	0.97	0.75	0	0
C2_03750W_A	0.07	0.62	0.46	0.23	0.06	0.03
C5_00940C_A	-0.12	0.41	0.25	-0.14	0.35	0.23
C2_04640C_A	-0.43	0.01	0	-0.15	0.43	0.3
C1_05290W_A	0.11	0.15	0.07	0.02	0.82	0.75
C3_05520C_A	0.06	0.55	0.38	0.02	0.84	0.76
C1_04430C_A	0.3	0.01	0	0.47	0	0
C4_04200C_A	0.38	0.27	0.14	0.91	0.08	0.04
CR_06800C_A	-0.07	0.69	0.54	-0.46	0	0
C2_05000C_A	-0.11	0.2	0.1	-0.33	0	0
C2_04580W_A	0.16	0.04	0.02	0.22	0.01	0.01
C1_13870W_A	-0.44	0	0	-0.56	0	0
C3_02030W_A	0.05	0.72	0.58	-0.11	0.35	0.23
C2_04700C_A	-0.54	0.07	0.03	-0.22	0.53	0.39
C2_04090W_A	0.53	0.26	0.14	1.18	0.02	0.01
C7_01570C_A	-0.41	0.02	0.01	-1.17	0	0
C7_02140W_A	0.26	0.57	0.41	0.69	0.09	0.05
C3_06250W_A	0.55	0	0	0.54	0	0
C1_11150W_A	0.2	0.15	0.07	0.51	0	0
CR_02400W_A	-0.27	0.33	0.19	-0.48	0.28	0.17
C4_05880W_A	0.24	0.03	0.01	-0.19	0.16	0.08
C1_09530W_A	-0.01	0.95	0.9	-0.03	0.89	0.83
CR_07960C_A	0.08	0.69	0.55	0.08	0.71	0.59
C1_12870C_A	-0.64	0.13	0.06	-0.64	0.2	0.11
C2_00140W_A	0.46	0	0	0.49	0	0
C1_11120C_A	0.26	0.02	0	0.26	0.01	0
C6_03830W_A	0.43	0	0	0.58	0	0
C1_01290C_A	-0.3	0.17	0.08	-0.1	0.69	0.57
C4_00430W_A	0.1	0.57	0.4	0.14	0.38	0.26
C3_02850C_A	-0.25	0.35	0.2	-0.07	0.82	0.74
C4_02020W_A	0.49	0	0	0.56	0	0
CR_06970C_A	-0.81	0	0	-0.65	0	0
C1_03510C_A	0.1	0.35	0.2	0	1	1
C5_01390C_A	0.13	0.73	0.6	0.58	0.03	0.01
C5_03410C_A	-0.03	0.88	0.8	-0.3	0.05	0.02
CR_07220C_A	-0.06	0.88	0.8	-0.02	0.97	0.95
C4_03990C_A	0.32	0.09	0.04	0.59	0	0
C1_04220C_A	0.16	0.36	0.21	0.13	0.51	0.38
C1_02270C_A	-0.15	0.65	0.49	0.38	0.17	0.09

C4_05280W_A	0.12	0.79	0.67	0.57	0.24	0.14
C1_07440W_A	-0.3	0.01	0	-0.25	0.05	0.02
C6_01760W_A	1.01	0	0	0.59	0	0
C5_05100C_A	-0.41	0	0	-0.17	0.29	0.18
C2_01260W_A	-0.19	0.19	0.09	-0.26	0.1	0.05
CR_10390W_A	0.12	0.62	0.45	0.34	0.06	0.03
C1_11540C_A	0.07	0.51	0.35	-0.22	0.01	0.01
C2_03250W_A	0.05	0.73	0.59	-0.18	0.14	0.07
C3_07680W_A	-0.06	0.67	0.52	-0.07	0.64	0.51
C3_07200C_A	0.52	0.01	0	1	0	0
C1_11090C_A	-0.06	0.72	0.58	0.04	0.82	0.74
C2_07040W_A	-0.24	0.2	0.1	0.16	0.45	0.32
C4_03920W_A	-0.29	0.09	0.04	-0.07	0.74	0.64
CR_07060C_A	0.31	0.01	0	1.17	0	0
C4_00160C_A	0.37	0	0	0.07	0.51	0.37
CR_07890W_A	-0.05	0.73	0.59	0.21	0.05	0.02
C5_02310C_A	0.41	0.13	0.06	0.72	0	0
C7_00310C_A	-0.12	0.46	0.29	0	0.99	0.98
C6_03540W_A	-0.05	0.84	0.74	-0.52	0.01	0
C5_01460W_A	0.11	0.57	0.4	0.15	0.44	0.31
C3_02020W_A	-1.17	0	0	-0.91	0	0
CR_00140W_A	-0.23	0.42	0.26	-0.2	0.44	0.31
C4_03690C_A	0.07	0.81	0.7	-0.07	0.81	0.72
C5_05230C_A	0.5	0	0	0.57	0	0
C2_03360W_A	-0.05	0.66	0.5	-0.1	0.31	0.2
CR_09510C_A	-0.33	0	0	-0.43	0	0
CR_10730C_A	0.04	0.81	0.7	-0.08	0.6	0.46
C1_03940W_A	0.32	0	0	0.19	0.08	0.04
C1_03680W_A	-0.8	0	0	-1.49	0	0
C1_02220C_A	-0.44	0	0	-0.27	0.04	0.02
C1_02850W_A	-0.32	0	0	0	0.99	0.99
C7_03000C_A	-0.14	0.22	0.11	-0.33	0	0
C6_04490W_A	0.26	0.29	0.16	0.36	0.11	0.05
C4_01520C_A	0.13	0.02	0.01	-0.43	0	0
C7_02610C_A	-0.1	0.26	0.13	-0.15	0.11	0.05
C7_04030C_A	0.25	0.02	0	0.28	0.01	0
C2_03810C_A	0.02	0.81	0.7	-0.4	0	0
CR_04260W_A	-0.06	0.76	0.63	-0.28	0.12	0.06
CR_09190C_A	0.1	0.72	0.58	0.08	0.76	0.66
C4_01010C_A	0.42	0	0	1.9	0	0
C2_08050C_A	-0.52	0.18	0.08	-1.35	0	0
C1_02810W_A	0.16	0.07	0.03	-0.08	0.48	0.34
C2_07810W_A	-0.27	0.03	0.01	-0.8	0	0

C1_03820W_A	-0.44	0	0	-1.02	0	0
CR_08060C_A	0.34	0	0	0.51	0	0
CR_04990C_A	-0.23	0.64	0.48	-0.28	0.69	0.57
C4_03550W_A	-0.2	0.21	0.1	-0.04	0.83	0.75
C4_00130W_A	-2.88	0	0	-5.1	0	0
C2_08630C_A	-0.3	0.03	0.01	-0.2	0.27	0.17
C1_06190C_A	-0.06	0.75	0.62	-0.25	0.08	0.04
C2_01210C_A	-0.17	0.57	0.4	0.33	0.13	0.07
CR_10840C_A	0.3	0	0	0.36	0	0
CR_03150W_A	0.02	0.97	0.93	0.4	0.02	0.01
C2_07140W_A	0.09	0.53	0.37	0.5	0	0
CR_09560C_A	0.32	0.21	0.11	0.55	0.02	0.01
C4_00070C_A	0.7	0	0	0.72	0	0
C3_00390W_A	0.47	0.04	0.01	0.33	0.19	0.1
C3_05560W_A	0.19	0.06	0.02	0.28	0.01	0
C6_02140W_A	0.49	0	0	0.56	0	0
CR_09730C_A	-0.58	0	0	-0.36	0.03	0.01
C7_00980W_A	-0.23	0.03	0.01	-0.55	0	0
C3_00880W_A	0.61	0	0	0.5	0	0
C5_00790C_A	0.33	0	0	0.35	0	0
C4_03160C_A	-0.02	0.94	0.9	-0.13	0.55	0.41
C6_01740C_A	0.46	0	0	0.21	0.08	0.04
C2_07880W_A	-0.05	0.77	0.65	0.16	0.29	0.18
CR_09700W_A	-0.11	0.7	0.56	-0.1	0.72	0.61
C3_06050C_A	0.33	0	0	0.15	0.21	0.12
C2_09700W_A	0.06	0.86	0.77	0.19	0.53	0.4
C2_00380C_A	0.02	0.85	0.75	0.15	0.09	0.04
C4_05810W_A	0.19	0.14	0.06	0.69	0	0
C4_00910C_A	-0.59	0	0	-0.45	0	0
C2_01760C_A	-0.1	0.84	0.74	-0.29	0.49	0.36
C7_04140C_A	-0.47	0	0	-0.45	0	0
C7_04230W_A	0.26	0	0	0.25	0	0
C1_05270C_A	-0.14	0.11	0.05	-0.25	0	0
C1_02980W_A	-0.66	0	0	-0.9	0	0
C2_07540W_A	0.3	0.05	0.02	0.42	0	0
CR_03080C_A	0.08	0.74	0.6	0.17	0.44	0.31
C1_01050C_A	0.23	0.03	0.01	0.05	0.7	0.58
CR_03990C_A	-0.28	0	0	-0.3	0	0
C6_02520W_A	-0.19	0.19	0.09	-0.15	0.34	0.22
C1_13400C_A	-0.1	0.54	0.37	-0.33	0.02	0.01
C7_02060W_A	0.02	0.9	0.83	-0.05	0.75	0.64
C1_03790C_A	-0.4	0	0	-1.45	0	0
C4_05600W_A	0.02	0.94	0.89	0.17	0.23	0.13

C1_11700C_A	-0.19	0.05	0.02	0.3	0	0
C5_00850C_A	0.45	0.31	0.17	0.61	0.18	0.1
C2_05060C_A	0.13	0.8	0.68	0.3	0.53	0.39
C3_05390C_A	0.17	0.38	0.23	-0.09	0.68	0.56
C2_02340C_A	0.34	0	0	0.5	0	0
C3_04480C_A	0.14	0.71	0.57	0.31	0.34	0.22
C3_01180C_A	-0.18	0.2	0.1	-0.35	0.03	0.01
C3_03790W_A	0.13	0.48	0.32	0.33	0.03	0.01
C2_08060W_A	0.13	0.79	0.67	0.32	0.47	0.33
C6_03900W_A	-0.02	0.87	0.78	-0.2	0.05	0.02
C2_07270W_A	0.47	0	0	0.46	0	0
C1_00370W_A	0.1	0.8	0.68	0.08	0.84	0.77
C1_07610C_A	0.22	0.43	0.27	0.23	0.4	0.27
C2_07490W_A	0.36	0	0	0.37	0	0
C7_00700W_A	0	1	1	-0.23	0.11	0.05
C1_08820C_A	0.03	0.94	0.89	0.11	0.71	0.6
C1_11330C_A	0.25	0.01	0	0.26	0.01	0
C5_05250C_A	-0.21	0.01	0	-0.42	0	0
CR_02790C_A	0.02	0.95	0.9	-0.17	0.42	0.29
CR_09740W_A	-0.53	0.01	0	-0.07	0.79	0.7
C4_03820C_A	0.29	0.52	0.36	0.92	0.01	0.01
C3_06350W_A	0.14	0.35	0.2	0.05	0.79	0.7
C3_05210C_A	0.36	0	0	0.28	0.02	0.01
C6_00970C_A	-0.36	0	0	-0.31	0.01	0
C2_00920W_A	0.27	0.16	0.08	0.31	0.11	0.06
C1_01740W_A	-0.21	0.68	0.52	0.15	0.83	0.76
C3_06650C_A	-0.14	0.42	0.26	-0.31	0.04	0.02
C1_08800W_A	-0.1	0.39	0.24	-0.35	0	0
C4_05830W_A	0.12	0.69	0.55	0.22	0.66	0.53
CR_08480C_A	-0.4	0.04	0.01	-0.66	0	0
CR_04120C_A	-0.26	0.02	0.01	-0.41	0	0
C5_01180W_A	-0.14	0.44	0.27	-0.31	0.05	0.02
C1_07490C_A	-0.04	0.79	0.67	-0.09	0.56	0.42
C1_10120C_A	0.01	0.97	0.94	0.19	0.08	0.04
C6_00340C_A	0.19	0.04	0.01	0.12	0.28	0.17
C2_00710W_A	-0.2	0.06	0.02	-0.21	0.07	0.03
C3_02060W_A	0.24	0.59	0.42	0.48	0.26	0.16
C1_07830C_A	0.02	0.95	0.92	-0.1	0.7	0.58
C2_05920C_A	0.43	0	0	0.37	0	0
C5_02440C_A	-0.08	0.78	0.66	0.03	0.93	0.88
C4_06560W_A	0.16	0.75	0.62	0.47	0.32	0.21
C3_02370C_A	-0.1	0.34	0.2	0.12	0.28	0.17
C3_04770C_A	0.37	0.04	0.01	0.5	0	0

C1_07230W_A	0.09	0.26	0.14	-0.02	0.84	0.77
CR_05460W_A	-0.09	0.66	0.51	-0.02	0.93	0.88
C4_03350C_A	-0.45	0.02	0.01	-0.41	0.04	0.02
CR_07420W_A	0.51	0	0	0.67	0	0
CR_02940C_A	0.23	0.41	0.26	0.41	0.1	0.05
C4_02180C_A	0.47	0.09	0.03	0.91	0	0
C1_00660C_A	0.26	0.03	0.01	0.57	0	0
C2_06780C_A	-0.07	0.8	0.68	0.24	0.28	0.17
C4_05290W_A	-0.28	0	0	-0.23	0.07	0.03
CR_08330W_A	-0.06	0.69	0.54	-0.12	0.39	0.26
C2_04110W_A	-0.39	0.13	0.06	-0.43	0.11	0.05
C3_05420W_A	-0.31	0.03	0.01	-0.04	0.84	0.76
C3_00110C_A	0.23	0.01	0	0.12	0.22	0.13
C3_04120C_A	-0.06	0.82	0.71	-0.06	0.8	0.72
C2_09500W_A	-0.18	0.34	0.2	-0.01	0.96	0.93
C4_03730C_A	-0.46	0	0	-0.69	0	0
C2_03540W_A	-0.12	0.37	0.22	-0.3	0.01	0
CR_03090C_A	0.52	0	0	0.78	0	0
C4_02070W_A	-0.22	0.45	0.29	0.23	0.37	0.24
C4_03120C_A	-0.09	0.57	0.41	-0.13	0.39	0.26
C1_04720W_A	-0.05	0.92	0.86	0.26	0.54	0.4
BGI_novel_G000(	0.03	0.87	0.79	-0.07	0.69	0.58
C5_02510C_A	0.01	0.99	0.97	-0.05	0.89	0.83
CR_07640C_A	-0.17	0.19	0.09	-0.36	0	0
C1_11870W_A	-0.58	0	0	-1.17	0	0
C3_00540C_A	0.13	0.57	0.4	0.14	0.56	0.42
C2_06920C_A	-0.59	0	0	-0.45	0.01	0
C1_13190W_A	0.2	0.05	0.02	0.29	0.01	0
CR_01650W_A	-0.1	0.41	0.25	-0.01	0.92	0.88
C5_01870W_A	-0.07	0.48	0.31	-0.19	0.05	0.02
C1_08600C_A	-0.11	0.28	0.15	-0.09	0.38	0.25
C5_04370C_A	0.49	0.28	0.15	0.53	0.42	0.29
C6_02980C_A	-0.72	0	0	-0.25	0.06	0.03
C6_04170C_A	-0.22	0.06	0.02	-0.33	0.01	0
C3_01130C_A	0.05	0.91	0.84	0.85	0	0
C1_08850C_A	0.71	0.01	0	0.93	0	0
C1_11250W_A	0.31	0.01	0	0.71	0	0
C5_05050W_A	0.03	0.78	0.66	0.01	0.9	0.85
C4_04310W_A	-0.32	0	0	-0.61	0	0
C2_06640C_A	-0.09	0.31	0.18	-0.36	0	0
CR_06500C_A	-1.19	0	0	-0.68	0.01	0
C1_04240C_A	-0.35	0	0	-0.6	0	0
C5_00090C_A	-0.18	0.07	0.03	-0.14	0.24	0.14

C5_04830W_A	0.42	0	0	0.35	0.01	0
CR_08450C_A	-0.18	0.23	0.12	0.22	0.12	0.06
C1_08110W_A	0.19	0.03	0.01	-0.04	0.74	0.63
C2_00250W_A	0.18	0.15	0.07	0.56	0	0
C1_00430W_A	0.08	0.57	0.4	0.2	0.09	0.05
C1_10340W_A	0.02	0.96	0.93	-0.05	0.86	0.79
C1_06570W_A	0.36	0.08	0.03	0.64	0	0
C3_04640W_A	0.1	0.63	0.47	0.28	0.11	0.05
CR_05900W_A	0.2	0.32	0.18	0.3	0.12	0.06
C1_13700W_A	0.67	0	0	0.79	0	0
C5_05170W_A	0.05	0.74	0.61	-0.07	0.61	0.47
C1_05360C_A	-0.37	0.03	0.01	-0.33	0.07	0.03
CR_08010W_A	-0.44	0.09	0.04	-0.78	0	0
CR_00060C_A	0.3	0	0	0.12	0.28	0.17
C1_10630C_A	0.17	0.35	0.2	0.25	0.12	0.06
C5_03350W_A	0.75	0	0	0.91	0	0
CR_09640C_A	-0.05	0.94	0.88	0.35	0.53	0.39
CR_03660C_A	-0.11	0.61	0.44	-0.29	0.11	0.05
C1_12390C_A	-0.21	0	0	-0.58	0	0
C7_02170C_A	-0.02	0.91	0.85	-0.33	0.02	0.01
C1_12750C_A	-0.35	0.14	0.07	-0.11	0.68	0.56
C3_07430W_A	-0.29	0	0	-0.53	0	0
C1_10070C_A	-0.33	0.01	0	-0.06	0.73	0.62
C3_02710W_A	0.95	0	0	1.79	0	0
C5_04600C_A	0.28	0	0	0.32	0	0
C7_01950W_A	-0.45	0.01	0	-0.43	0.02	0.01
CR_09800C_A	-1.03	0	0	-1.56	0	0
C2_03450W_A	0.01	0.94	0.9	-0.1	0.46	0.33
C1_09690W_A	-0.13	0.29	0.16	-0.16	0.22	0.13
C2_06660W_A	-0.64	0.03	0.01	-0.12	0.72	0.61
C2_08670C_A	-0.43	0	0	-0.43	0.01	0
C1_07960W_A	-0.37	0.4	0.25	-0.37	0.38	0.26
C5_05460C_A	0.25	0.05	0.02	0.24	0.09	0.04
C4_02480C_A	0.16	0.42	0.26	0.14	0.47	0.34
C6_00920W_A	0.58	0	0	1	0	0
CR_02990C_A	-0.06	0.67	0.52	-0.05	0.75	0.64
C3_05910W_A	-0.26	0.32	0.18	0.15	0.56	0.42
C4_05300W_A	-0.12	0.2	0.1	-0.27	0	0
C1_13290W_A	-0.38	0.46	0.29	-0.18	0.78	0.68
C5_02740W_A	-0.06	0.73	0.59	-0.15	0.35	0.23
C6_03270C_A	0.21	0.07	0.03	0.37	0	0
C1_07620C_A	0.23	0.67	0.52	0.04	0.96	0.94
C1_00550W_A	0.25	0.04	0.01	0.11	0.42	0.29

CR_07860C_A	-0.8	0	0	-0.55	0.01	0
C4_00420C_A	-0.13	0.13	0.06	-0.33	0	0
C4_05340W_A	0.6	0	0	0.67	0	0
C4_06930C_A	-0.16	0.52	0.35	-0.07	0.78	0.68
C7_02850W_A	0.2	0.11	0.04	-0.17	0.19	0.1
C2_01560W_A	0.53	0	0	0.5	0	0
C1_04910C_A	-0.08	0.52	0.36	-0.19	0.1	0.05
C2_00870W_A	-0.31	0.1	0.04	-0.5	0.01	0
C3_02360C_A	-2.37	0	0	-2.91	0	0
C3_00630W_A	0.46	0	0	0.41	0	0
C2_08510W_A	0.28	0.46	0.3	0.14	0.74	0.63
C3_07570C_A	-0.01	0.99	0.98	0.02	0.96	0.94
C5_04540C_A	-0.55	0.02	0.01	-0.26	0.32	0.2
C2_08810C_A	0.12	0.15	0.07	0.06	0.59	0.45
C6_03330C_A	-0.07	0.68	0.52	0.23	0.08	0.04
C4_00840W_A	0.1	0.5	0.33	0.48	0	0
C4_00080C_A	0.76	0	0	0.99	0	0
CR_01580C_A	-0.28	0.08	0.03	-0.22	0.25	0.15
C7_03490W_A	0.27	0.01	0	0.49	0	0
C1_09180W_A	0.17	0.55	0.39	0.43	0.08	0.04
C3_04500C_A	-0.08	0.28	0.15	-0.56	0	0
C6_03790C_A	-1.64	0	0	-2.03	0	0
C2_05690C_A	0.69	0	0	0.62	0	0
CR_02630C_A	-0.19	0.44	0.28	0.03	0.92	0.87
C7_04040C_A	0.21	0.02	0.01	0.16	0.13	0.07
C6_04560W_A	0.3	0	0	0.17	0.13	0.06
C4_05430C_A	0.09	0.49	0.32	-0.32	0	0
C5_00930C_A	-0.15	0.07	0.03	0.07	0.56	0.42
C2_00560W_A	-0.21	0.53	0.37	0.26	0.7	0.58
C2_05570C_A	-0.45	0.03	0.01	-0.82	0	0
C7_01610W_A	-0.09	0.63	0.47	-0.2	0.21	0.12
C3_07360W_A	0.21	0.16	0.08	0.52	0	0
C5_03550W_A	-0.17	0.04	0.01	-0.49	0	0
C4_06740C_A	0.17	0.08	0.03	0.15	0.13	0.07
C1_01400C_A	0.26	0.02	0	0.03	0.83	0.76
C3_07280C_A	-0.3	0	0	-0.5	0	0
CR_03710C_A	0.24	0.21	0.1	0.62	0	0
C1_10010C_A	0.3	0.01	0	0.54	0	0
C4_02310W_A	0.17	0.2	0.1	0.38	0	0
C1_04330W_A	0.54	0	0	0.87	0	0
C7_00690W_A	-0.99	0	0	-1.05	0	0
CR_00430C_A	-0.09	0.69	0.54	-0.15	0.49	0.35
C2_06210C_A	-0.28	0	0	-0.6	0	0

C2_00160C_A	0.09	0.49	0.32	-0.08	0.58	0.44
C4_07230C_A	0.31	0	0	0.11	0.41	0.28
C1_06740C_A	-0.87	0	0	-1.3	0	0
C5_02350C_A	-0.16	0.69	0.54	0.21	0.59	0.45
C1_12120W_A	0.33	0.06	0.02	0.42	0.02	0.01
C2_08190W_A	0.23	0.03	0.01	0.22	0.04	0.02
C7_00670C_A	0.51	0	0	0.67	0	0
C3_00780W_A	-0.37	0.05	0.02	-0.13	0.57	0.43
C3_00750W_A	0	1	0.99	0.3	0.12	0.06
C2_05840W_A	-0.34	0.21	0.1	0.38	0.11	0.06
C7_02560W_A	-0.01	0.97	0.94	0.33	0.06	0.03
CR_08930C_A	-0.1	0.39	0.24	-0.14	0.24	0.14
C7_02330W_A	0.09	0.54	0.37	-0.18	0.19	0.11
C3_03390C_A	-0.35	0.04	0.01	-0.57	0	0
C5_00550C_A	-0.24	0.49	0.32	-0.2	0.57	0.44
C6_02110W_A	-0.21	0.49	0.33	-0.01	0.99	0.98
C4_05590W_A	-0.33	0	0	-0.82	0	0
C2_09960W_A	0.09	0.75	0.62	0.24	0.3	0.19
C7_00140C_A	0.55	0	0	0.92	0	0
C4_00610W_A	0.37	0.03	0.01	0.15	0.5	0.36
C4_04480C_A	-0.03	0.75	0.62	-0.4	0	0
C1_03330C_A	0.26	0.14	0.06	0.62	0	0
C1_00100C_A	0.13	0.4	0.24	0.05	0.79	0.69
C5_01320W_A	0.55	0	0	0.6	0	0
CR_00470W_A	0.34	0	0	0.35	0	0
C7_03720C_A	0.12	0.17	0.08	-0.13	0.17	0.09
C4_04250W_A	0.1	0.7	0.56	0.1	0.69	0.57
C7_04300W_A	0.05	0.62	0.45	-0.09	0.33	0.21
C6_01720C_A	-0.34	0	0	-0.83	0	0
C6_04530C_A	-0.36	0	0	-0.59	0	0
C1_01780C_A	-0.06	0.78	0.66	-0.6	0	0
CR_05670C_A	0.07	0.44	0.28	-0.34	0	0
C6_04580W_A	0.38	0	0	0.52	0	0
C3_03580C_A	-0.14	0.72	0.58	0.33	0.62	0.49
C7_03920C_A	-0.15	0.04	0.01	-0.57	0	0
CR_06100C_A	0.46	0.3	0.16	1.22	0	0
C2_01270W_A	-1.19	0	0	-2.32	0	0
C2_01220W_A	-0.22	0.04	0.02	-0.33	0.01	0
C2_01240C_A	-0.52	0.02	0	-0.23	0.33	0.21
C3_05320W_A	-0.3	0.05	0.02	-0.16	0.31	0.2
C5_01910W_A	-0.13	0.15	0.07	-0.45	0	0
C1_07600W_A	0.53	0	0	1	0	0
C3_00920W_A	0.24	0.54	0.38	-0.27	0.47	0.33

C1_06710W_A	0.31	0.05	0.02	0.4	0.01	0
C2_10020C_A	0.05	0.89	0.81	0.48	0.42	0.29
C4_06450W_A	0.06	0.81	0.7	-0.01	0.98	0.96
CR_04200W_A	0.03	0.77	0.64	-0.01	0.96	0.93
C7_02600C_A	-0.4	0	0	-0.89	0	0
C2_02450C_A	-0.49	0	0	-0.63	0	0
C3_04590W_A	0.24	0	0	-0.07	0.58	0.45
C4_01820C_A	0.16	0.18	0.08	0.24	0.03	0.01
C7_01910C_A	-0.34	0.37	0.22	-0.11	0.79	0.7
C3_03340C_A	0.2	0.37	0.22	0.56	0	0
C7_00550C_A	0.03	0.83	0.73	-0.09	0.45	0.32
C6_02870W_A	-0.1	0.53	0.37	-0.14	0.38	0.25
C4_05910C_A	0.12	0.4	0.25	0.17	0.24	0.14
C2_02370C_A	0.23	0	0	-0.04	0.69	0.57
C1_11390W_A	-0.09	0.89	0.81	-0.06	0.93	0.89
C5_00390C_A	0.09	0.51	0.35	0.18	0.2	0.12
C1_10770W_A	0.25	0.24	0.12	0.37	0.07	0.03
C1_14300C_A	-0.12	0.34	0.2	-0.91	0	0
C2_09860C_A	-0.43	0.01	0	-0.39	0.03	0.01
C4_02690W_A	0.32	0	0	0.09	0.48	0.34
C4_06600W_A	-0.1	0.45	0.28	-0.31	0.01	0
CR_04440C_A	-0.06	0.83	0.73	0.29	0.62	0.49
C5_00300C_A	-0.25	0.09	0.03	-0.12	0.57	0.43
CR_02690W_A	-0.06	0.8	0.68	-0.07	0.74	0.64
C3_02780W_A	0.19	0.01	0	0.31	0	0
CR_03210C_A	-0.15	0.52	0.36	0.01	0.97	0.95
C1_00480C_A	-0.03	0.79	0.67	-0.06	0.65	0.51
C1_14170W_A	0.53	0	0	0.59	0	0
C1_10400C_A	-0.54	0	0	-1.1	0	0
C4_04600C_A	0.28	0.06	0.02	0.26	0.12	0.06
C2_10670W_A	0.44	0	0	0.54	0	0
C6_04400W_A	-0.16	0.78	0.65	0.1	0.87	0.81
C3_07610W_A	0.51	0	0	0.43	0	0
C5_01070C_A	-0.5	0	0	-0.73	0	0
C1_12820C_A	-0.32	0.05	0.02	-0.33	0.04	0.02
C1_12370W_A	0.44	0.03	0.01	0.52	0.01	0
C1_06050C_A	0.46	0	0	0.49	0	0
CR_06120W_A	-1.13	0	0	-0.91	0	0
C6_00420W_A	1	0	0	1.21	0	0
C6_00430C_A	0.03	0.84	0.74	0.07	0.66	0.53
C2_01920C_A	-0.31	0.01	0	-0.14	0.42	0.29
CR_03800C_A	-0.23	0.29	0.16	-0.35	0.09	0.04
C2_02240C_A	0.09	0.59	0.43	0.1	0.52	0.38

C5_02810W_A	-0.49	0.18	0.09	-0.78	0.04	0.02
C6_00320C_A	-0.12	0.44	0.28	-0.01	0.94	0.9
CR_05440W_A	-0.05	0.7	0.56	-0.17	0.15	0.08
CR_09780C_A	0.64	0.06	0.02	0.57	0.15	0.08
C4_02270C_A	0.46	0	0	0.6	0	0
C3_03560W_A	0.15	0.21	0.11	0.27	0.02	0.01
C7_03580C_A	-0.05	0.8	0.68	-0.43	0	0
C2_02190C_A	-0.07	0.87	0.78	-0.35	0.32	0.2
C5_03650C_A	0.62	0.11	0.05	0.57	0.2	0.11
C1_04640W_A	0.59	0	0	1.18	0	0
C3_01700W_A	0.06	0.71	0.57	0.22	0.09	0.04
C1_01010W_A	0.32	0	0	0.31	0	0
C6_00960W_A	0.19	0.33	0.19	-0.39	0.05	0.02
C4_06370C_A	0.07	0.84	0.75	0.22	0.43	0.3
C5_04060C_A	0.06	0.71	0.57	0.01	0.94	0.9
C4_03070W_A	0.82	0	0	1.06	0	0
C3_01600W_A	0.22	0.07	0.03	0.09	0.52	0.38
CR_10540C_A	-0.34	0.02	0.01	-0.22	0.15	0.08
C3_03300C_A	0.29	0.01	0	0.33	0.01	0
C3_00380C_A	-0.04	0.89	0.81	-0.05	0.81	0.73
C3_06060W_A	-0.45	0.01	0	-0.14	0.49	0.35
CR_01190C_A	0.42	0.28	0.15	0.57	0.14	0.07
C1_08040W_A	0.33	0.51	0.35	0.53	0.34	0.22
C3_00670C_A	-0.18	0.15	0.07	-0.47	0	0
CR_07850W_A	-0.02	0.92	0.86	0.2	0.25	0.15
C1_00680W_A	-0.11	0.62	0.45	-0.54	0.01	0
C1_08780W_A	0.21	0.01	0	-0.49	0	0
C4_00240C_A	0.59	0	0	0.54	0	0
C5_04280C_A	-0.3	0.03	0.01	0.06	0.7	0.58
C1_08670W_A	0	0.99	0.97	0.1	0.44	0.31
C2_00180C_A	0.41	0	0	0.75	0	0
C3_00300W_A	0.32	0.01	0	0.52	0	0
C6_04070C_A	-0.49	0.22	0.11	0.04	0.93	0.89
CR_03490W_A	0.12	0.53	0.37	0	1	1
C2_07760W_A	0.18	0.29	0.16	0.37	0.02	0.01
C3_02120W_A	0.12	0.49	0.32	-0.01	0.96	0.93
C5_02290W_A	-0.18	0.56	0.4	-0.07	0.82	0.73
CR_10660W_A	0.32	0.01	0	0.3	0.02	0.01
C5_04660C_A	-0.05	0.83	0.73	0.06	0.75	0.64
C4_05200C_A	0.22	0.66	0.5	0.06	0.94	0.9
C7_02670W_A	0.11	0.59	0.43	0.2	0.34	0.22
C2_01630W_A	-0.72	0.07	0.03	-0.86	0.04	0.02
C4_00620C_A	0.83	0	0	0.95	0	0

C3_07040C_A	0.16	0.1	0.04	0.13	0.3	0.19
C1_06910C_A	-0.13	0.37	0.22	0.07	0.66	0.53
C4_01560C_A	-0.8	0	0	-0.86	0	0
C5_00490C_A	-0.16	0.37	0.22	-0.04	0.82	0.74
C2_04730W_A	-0.48	0.01	0	-0.09	0.69	0.57
C3_00810C_A	0.71	0	0	0.99	0	0
CR_10510W_A	0.06	0.74	0.6	0.15	0.41	0.28
CR_07780W_A	-0.11	0.44	0.28	-0.2	0.15	0.08
C1_13570W_A	0.36	0.02	0.01	0.66	0	0
C5_02710W_A	0.32	0	0	0.34	0	0
C5_00560W_A	0.05	0.75	0.61	0.31	0.01	0
C4_00190W_A	0.14	0.67	0.52	0	1	0.99
C1_01360C_A	0.1	0.23	0.12	-0.18	0.04	0.01
CR_06300C_A	0.3	0.04	0.01	0.11	0.54	0.4
C1_00030C_A	-0.03	0.96	0.92	0.08	0.89	0.83
CR_05720W_A	0.23	0	0	0.13	0.12	0.06
C5_03930C_A	-0.17	0.45	0.29	0.24	0.21	0.12
C1_07040C_A	-0.56	0.21	0.1	-0.8	0.1	0.05
C4_01060W_A	-0.26	0.01	0	-0.32	0.01	0
C4_03910W_A	0	1	0.99	-0.11	0.67	0.54
C2_09030W_A	-0.03	0.92	0.85	0.16	0.54	0.4
C4_05350W_A	0.09	0.74	0.61	0.27	0.21	0.12
C3_03020W_A	-0.26	0.62	0.46	-1.24	0.03	0.01
C3_06600C_A	-0.35	0.15	0.07	-0.22	0.38	0.26
C3_01640C_A	0.14	0.8	0.69	0.39	0.55	0.41
C1_12070C_A	0.59	0	0	0.75	0	0
C1_02680C_A	-0.07	0.78	0.65	-0.06	0.8	0.7
CR_08130W_A	0.03	0.81	0.7	-0.16	0.15	0.08
C6_02690C_A	-0.1	0.59	0.42	-0.02	0.92	0.87
C3_07250W_A	-0.11	0.3	0.17	-0.19	0.07	0.03
C4_06050W_A	0.03	0.92	0.86	-0.22	0.43	0.3
C2_07960C_A	-1.31	0	0	-1.47	0	0
C7_00410C_A	-0.36	0	0	-0.48	0	0
C6_04300W_A	-0.31	0.15	0.07	0.01	0.99	0.97
C2_02090W_A	-0.17	0.39	0.24	0.38	0.01	0.01
C6_03150C_A	-0.16	0.32	0.18	-0.15	0.39	0.26
C2_02690W_A	-0.46	0	0	-0.9	0	0
C7_00810W_A	-0.31	0.06	0.02	-0.19	0.27	0.16
C7_03390C_A	0.2	0.04	0.01	0.39	0	0
C1_08310W_A	0.31	0.03	0.01	0.98	0	0
C2_01680C_A	-0.32	0.02	0.01	-0.38	0.01	0
CR_07400C_A	0.26	0	0	0.25	0.01	0
CR_05580C_A	-0.07	0.67	0.52	-0.13	0.65	0.52

C2_04010C_A	-0.78	0	0	-0.5	0.02	0.01
C1_03180W_A	0.09	0.72	0.58	0.07	0.79	0.69
C1_04390C_A	-1.4	0	0	-1.53	0	0
CR_06860C_A	-0.66	0	0	-0.91	0	0
C2_01890W_A	-0.69	0	0	-0.42	0.01	0
C3_06290W_A	0.1	0.52	0.35	0.12	0.42	0.29
C1_14520W_A	0.15	0.21	0.1	0.37	0	0
C2_02060C_A	0.52	0.25	0.13	0.85	0.08	0.04
C3_06110C_A	0.05	0.73	0.6	0.15	0.25	0.15
CR_03510W_A	0.24	0.65	0.49	1.34	0.01	0
C1_00380C_A	-0.04	0.76	0.63	0.07	0.59	0.45
CR_08250C_A	-0.71	0	0	-0.69	0	0
C1_03980W_A	-0.21	0.5	0.33	-0.24	0.43	0.3
C3_06310C_A	-0.26	0.12	0.05	0.09	0.65	0.52
C6_01380C_A	-0.51	0.01	0	-0.43	0.04	0.02
C4_04970C_A	-0.09	0.72	0.58	0.15	0.5	0.36
C3_02670W_A	0.04	0.92	0.85	0.06	0.83	0.75
C1_11720W_A	-0.33	0.52	0.35	-0.4	0.51	0.37
C7_01200C_A	-0.88	0	0	-0.94	0	0
C1_08700W_A	-0.06	0.68	0.53	0.09	0.52	0.38
C5_02670W_A	-0.13	0.55	0.38	-0.2	0.28	0.17
CR_08640C_A	0.05	0.74	0.61	-0.15	0.23	0.13
C2_04250W_A	-0.03	0.89	0.81	-0.02	0.93	0.88
C6_04450W_A	0.13	0.37	0.22	0.05	0.79	0.69
C2_10420W_A	0.4	0.01	0	0.54	0	0
C1_01970W_A	NA	NA	NA	NA	NA	NA
C2_05980C_A	-0.46	0.03	0.01	-0.27	0.25	0.15
C2_09620W_A	0.38	0	0	0.3	0.01	0
CR_02250C_A	0.74	0	0	0.95	0	0
C5_03820C_A	0.58	0	0	0.71	0	0
C7_01420W_A	-0.38	0.26	0.14	-0.11	0.79	0.7
C6_00630W_A	0.29	0.52	0.36	0.54	0.22	0.13
C2_03560C_A	0.03	0.79	0.67	-0.13	0.21	0.12
C4_03890W_A	0.86	0	0	1.23	0	0
C4_01550C_A	0.78	0	0	1.18	0	0
CR_07380C_A	0.12	0.42	0.26	0.23	0.13	0.07
CR_06650C_A	-0.89	0.02	0	-1.53	0	0
C2_07420W_A	0.21	0.01	0	0.46	0	0
C1_03950C_A	-0.36	0.37	0.22	-0.44	0.32	0.2
C1_04420C_A	0.33	0	0	0.31	0.01	0
C1_13550C_A	-0.24	0.04	0.01	-0.13	0.31	0.2
C4_00770C_A	-0.47	0	0	-0.28	0.39	0.26
BGI_novel_G000(	-0.04	0.88	0.8	-0.27	0.22	0.12

C4_04870C_A	-0.01	0.96	0.93	-0.14	0.18	0.1
CR_10240W_A	-0.67	0	0	-0.72	0	0
CR_07320C_A	-0.14	0.22	0.11	-0.29	0.01	0.01
C6_02880W_A	0.33	0	0	0.32	0.01	0
C5_05080W_A	-0.29	0.2	0.1	0.06	0.81	0.72
CR_09090C_A	0.31	0.18	0.09	0.41	0.08	0.04
CR_10000C_A	-0.05	0.89	0.81	0.13	0.69	0.58
C6_00470C_A	0.65	0.15	0.07	0.65	0.26	0.16
C2_10610W_A	0.8	0	0	0.89	0	0
C6_01060C_A	-0.02	0.9	0.82	-0.03	0.83	0.76
C2_09170W_A	0.24	0.03	0.01	0.31	0	0
C5_00910C_A	-0.13	0.82	0.72	0.16	0.79	0.7
C1_05820C_A	0.1	0.65	0.49	0.21	0.27	0.16
C7_00260C_A	0.1	0.47	0.3	-0.31	0.02	0.01
C2_08090W_A	0.15	0.72	0.58	-0.22	0.59	0.46
CR_05210W_A	-0.26	0.12	0.05	-0.64	0	0
CR_01370C_A	-0.18	0.07	0.03	-0.32	0	0
C4_01280C_A	-0.16	0.26	0.13	0.31	0.02	0.01
C2_08330W_A	0.2	0.58	0.42	0.8	0	0
C3_05640W_A	-0.05	0.93	0.87	-0.12	0.86	0.79
C4_02230C_A	0.15	0.5	0.33	0.45	0.31	0.2
CR_04000W_A	0.19	0.01	0	0.27	0	0
C3_05280C_A	-0.05	0.77	0.64	0.12	0.37	0.24
C3_06340W_A	-0.02	0.94	0.89	0.47	0	0
C3_04990W_A	0.03	0.85	0.75	0.33	0	0
C1_02860C_A	0.55	0	0	0.77	0	0
C1_07220W_A	0.05	0.91	0.84	0.73	0.02	0.01
CR_08310C_A	-1.37	0	0	-1.72	0	0
C6_04290W_A	-0.08	0.49	0.32	-0.1	0.38	0.25
CR_01820W_A	-0.29	0.03	0.01	-0.26	0.08	0.04
C1_03830C_A	-0.65	0	0	-0.42	0	0
CR_09200C_A	0.01	0.97	0.95	-0.13	0.63	0.5
CR_10680W_A	0.24	0	0	-0.04	0.76	0.66
C1_10450W_A	0.07	0.37	0.22	0.32	0	0
C4_04530C_A	0.05	0.77	0.64	-0.79	0	0
C6_00850W_A	-0.11	0.49	0.33	0.26	0.02	0.01
C2_10440C_A	0.18	0.1	0.04	0.03	0.81	0.72
C1_09950C_A	0.07	0.76	0.64	-0.02	0.94	0.91
CR_04510W_A	0.21	0	0	0.1	0.22	0.12
C3_04300C_A	-0.06	0.66	0.5	0.04	0.8	0.71
C2_09250W_A	0.12	0.57	0.41	0.36	0.04	0.02
C2_01880C_A	-0.48	0.26	0.14	-0.5	0.28	0.17
C2_04480W_A	0.31	0.32	0.18	0.35	0.27	0.16

C3_00900C_A	0.13	0.37	0.22	0.25	0.07	0.03
C1_13640W_A	-0.04	0.79	0.67	0.26	0.03	0.01
C5_02570W_A	0.05	0.84	0.75	0.05	0.87	0.8
CR_09970W_A	0.54	0	0	0.54	0	0
C1_09170W_A	-0.85	0	0	-0.8	0	0
C4_03250C_A	-0.17	0.5	0.33	-0.31	0.15	0.08
C3_05400C_A	-0.1	0.85	0.76	-0.2	0.71	0.59
C2_03290W_A	0.72	0	0	0.88	0	0
C3_03180C_A	0.28	0	0	-0.11	0.38	0.25
C3_00820W_A	0.05	0.68	0.53	-0.68	0	0
C1_07860W_A	0	1	0.99	0.35	0.22	0.13
C1_10850W_A	-0.07	0.82	0.72	0.31	0.61	0.47
C1_00630W_A	0.71	0	0	0.83	0	0
C7_00420C_A	-0.12	0.65	0.48	-0.48	0.03	0.01
C4_05680W_A	-0.23	0.06	0.02	-0.69	0	0
C7_00840C_A	-0.1	0.74	0.61	-0.3	0.25	0.15
C2_10140W_A	0	1	0.99	0.34	0.13	0.07
CR_04720C_A	0.06	0.71	0.57	0.12	0.42	0.29
C4_05140C_A	0.05	0.61	0.44	0.06	0.51	0.37
CR_09890C_A	0.1	0.53	0.36	0.32	0.01	0
C2_06700W_A	0.33	0.01	0	0.32	0.02	0.01
C7_04170W_A	-0.33	0.43	0.27	-0.26	0.58	0.44
C2_08600W_A	-0.2	0.35	0.2	-0.39	0.04	0.02
C1_09850C_A	-0.11	0.44	0.28	-0.1	0.76	0.66
C1_14330W_A	-0.22	0.12	0.05	-0.21	0.19	0.1
C4_00020W_A	0.42	0	0	0.41	0	0
C3_04350C_A	0.62	0.09	0.04	1.15	0	0
C1_03750W_A	-0.07	0.53	0.36	-0.25	0.01	0
C3_03520C_A	0.27	0	0	0.38	0	0
C3_04920C_A	0.19	0.12	0.05	0.3	0.01	0
C3_03670W_A	-0.02	0.9	0.82	-0.1	0.54	0.4
CR_00220W_A	0.31	0.22	0.11	0.2	0.47	0.33
C5_03170C_A	-0.64	0	0	-0.55	0	0
C1_03860C_A	0.04	0.78	0.66	0.27	0.03	0.01
C1_12440W_A	-0.4	0.28	0.15	-0.21	0.62	0.48
C3_00180C_A	0.49	0	0	-0.13	0.61	0.47
C1_13750C_A	-0.77	0.04	0.01	-0.62	0.15	0.08
C5_01930W_A	0.09	0.67	0.52	0.26	0.15	0.08
C3_06030W_A	-1.51	0	0	-2.01	0	0
CR_04210C_A	-0.31	0	0	-0.69	0	0
C5_00860W_A	-0.38	0.08	0.03	-0.29	0.18	0.1
CR_02370W_A	-0.59	0	0	-0.46	0.02	0.01
C2_07460W_A	-0.26	0.03	0.01	-0.2	0.18	0.1

C2_10250C_A	-0.33	0.02	0.01	0.14	0.45	0.32
C2_01700C_A	-0.38	0.01	0	-0.44	0	0
C5_00620W_A	-0.12	0.74	0.6	0.52	0.05	0.02
C1_00310W_A	-0.06	0.74	0.6	0.01	0.95	0.92
C3_04650W_A	0.09	0.86	0.78	0.39	0.42	0.29
C4_07190W_A	0.18	0.05	0.02	0.19	0.06	0.03
CR_09670C_A	0.04	0.71	0.57	0.28	0	0
CR_06030C_A	0.27	0.19	0.09	0.22	0.33	0.21
C1_09580C_A	-0.17	0.75	0.62	-0.3	0.62	0.48
C1_05320C_A	0.39	0.01	0	0.65	0	0
C3_00330W_A	-0.17	0.1	0.04	-0.39	0	0
C1_02000W_A	0.24	0.07	0.03	0.06	0.75	0.65
C1_02030C_A	0.38	0.24	0.12	0.39	0.22	0.12
C4_06690C_A	0.13	0.32	0.18	0.49	0	0
C3_02590W_A	0.06	0.74	0.6	0.25	0.11	0.05
C4_06240W_A	0.13	0.49	0.33	0.04	0.83	0.76
C4_00380W_A	0.16	0.14	0.06	0.08	0.51	0.37
C3_02950C_A	-0.41	0.01	0	-0.01	0.98	0.96
C2_06090W_A	-0.06	0.83	0.73	0	1	0.99
C1_12110C_A	-0.41	0.22	0.11	-0.23	0.53	0.39
CR_09750C_A	-0.31	0.04	0.01	-0.53	0	0
C6_00180C_A	0.51	0.11	0.04	0.66	0.04	0.02
C3_01770C_A	-0.2	0.2	0.1	-0.22	0.14	0.07
C2_08040C_A	-0.16	0.06	0.02	-0.5	0	0
C3_05650W_A	-0.42	0	0	-0.49	0	0
C7_00240W_A	-0.07	0.72	0.59	-0.28	0.11	0.05
C1_01270W_A	0.13	0.32	0.18	-0.08	0.6	0.46
C2_10640C_A	0.12	0.38	0.23	0.18	0.14	0.07
C4_03480C_A	NA	NA	NA	NA	NA	NA
C1_12250C_A	-0.24	0.07	0.02	-0.25	0.04	0.02
C2_10050W_A	0.11	0.29	0.16	0.2	0.04	0.02
CR_03110W_A	-0.13	0.31	0.17	-0.34	0	0
C1_02750C_A	-0.14	0.13	0.05	-0.29	0	0
C6_00510C_A	0.14	0.36	0.21	0.35	0.01	0
C1_08530W_A	0.56	0	0	0.81	0	0
CR_05160C_A	-0.32	0.21	0.1	-0.33	0.2	0.11
CR_07710W_A	-0.15	0.08	0.03	-0.39	0	0
C5_02200W_A	0.2	0.62	0.46	0.41	0.24	0.14
C6_03460W_A	0.03	0.83	0.73	-0.01	0.93	0.88
C1_02400C_A	-0.17	0.45	0.29	-0.01	0.97	0.95
C7_00580C_A	0.02	0.94	0.89	0.14	0.49	0.35
C1_01280C_A	-0.11	0.81	0.7	-0.09	0.83	0.76
C7_00040C_A	-0.33	0.07	0.02	-0.43	0.02	0.01

CR_08820C_A	NA	NA	NA	NA	NA	NA
C2_04240C_A	0.45	0	0	0.42	0	0
C5_02070C_A	-0.25	0.11	0.05	-0.15	0.37	0.24
C4_00950C_A	0.04	0.83	0.73	-0.17	0.28	0.17
C3_03800W_A	0.34	0	0	-0.2	0.03	0.01
C1_06940C_A	0.17	0.12	0.05	-1.07	0	0
C7_04100C_A	0.15	0.37	0.22	0.06	0.77	0.67
C1_11630C_A	-0.34	0.48	0.32	-0.81	0.13	0.07
C2_05140W_A	0.2	0.16	0.08	-0.2	0.2	0.11
C7_01510W_A	-0.51	0.06	0.02	-0.16	0.62	0.49
C1_08830C_A	-0.37	0.23	0.11	-0.35	0.55	0.42
C4_06980W_A	0.94	0.01	0	0.65	0.19	0.11
C2_01490C_A	0.13	0.32	0.18	0.3	0.01	0
CR_00830W_A	-0.15	0.45	0.29	-0.22	0.32	0.2
C6_00080C_A	-0.18	0.38	0.22	-0.31	0.11	0.05
C5_00340W_A	-0.54	0	0	-0.57	0	0
C4_04380C_A	0.9	0.01	0	1.33	0	0
C1_13390W_A	-0.24	0.45	0.29	-0.2	0.55	0.42
C1_09510W_A	-0.99	0	0	-1.4	0	0
CR_00300W_A	0.1	0.87	0.78	0	1	1
C5_03700C_A	-0.09	0.54	0.37	-0.3	0.03	0.01
C1_03110W_A	-0.1	0.2	0.1	-0.56	0	0
C2_02640C_A	NA	NA	NA	NA	NA	NA
C6_03610W_A	0.4	0.01	0	0.28	0.11	0.05
C2_10690W_A	0.1	0.35	0.2	-0.25	0	0
CR_06960W_A	-0.46	0	0	-0.42	0	0
C1_10300W_A	0.26	0.07	0.03	0.53	0	0
C5_05310W_A	0.15	0.11	0.05	0.33	0	0
C7_03690W_A	0.26	0.49	0.33	0.34	0.36	0.23
CR_00640W_A	0.18	0.01	0	0.24	0	0
C3_01320C_A	-0.4	0.09	0.04	-0.74	0	0
C2_07210C_A	-0.07	0.5	0.34	-0.14	0.16	0.08
C3_04530C_A	0.08	0.64	0.47	-0.02	0.93	0.88
C5_04470C_A	1.28	0	0	2.4	0	0
C6_01030W_A	-0.82	0	0	-1.12	0	0
CR_05290W_A	0.18	0.28	0.15	0.1	0.62	0.48
C3_06710W_A	0.02	0.89	0.82	-0.06	0.69	0.57
C1_10640C_A	-0.05	0.79	0.67	0.4	0	0
C2_05230C_A	-0.55	0	0	-1.06	0	0
C2_04260W_A	0.02	0.93	0.87	0.24	0.21	0.12
C2_05170W_A	0.09	0.49	0.33	0.11	0.38	0.25
C2_08830W_A	0.17	0.37	0.22	0.29	0.07	0.03
C6_02420W_A	-0.24	0.34	0.2	0.12	0.66	0.54

C1_04880C_A	-0.21	0.32	0.18	-0.47	0.02	0.01
C7_01790C_A	-0.09	0.14	0.06	-0.6	0	0
C3_02640C_A	-0.38	0.1	0.04	-0.12	0.68	0.56
C7_01500W_A	0.24	0.19	0.09	0.14	0.48	0.34
C1_06110C_A	0.38	0	0	0.44	0	0
CR_01240C_A	-0.48	0	0	-0.2	0.11	0.05
C2_10650W_A	-0.3	0.55	0.38	-0.41	0.55	0.41
C2_09380W_A	-1.29	0	0	-1.07	0	0
C1_04490W_A	-0.06	0.73	0.59	0.07	0.62	0.49
C1_05200C_A	0.34	0.01	0	0.42	0	0
C3_06780C_A	0.13	0.68	0.53	0.17	0.58	0.44
CR_08150W_A	-0.14	0.05	0.02	-0.77	0	0
C2_03880C_A	-0.36	0.27	0.14	-0.41	0.21	0.12
C2_06340W_A	0.51	0.18	0.09	0.55	0.17	0.09
C1_06350W_A	-0.43	0	0	-0.79	0	0
CR_04740C_A	-0.93	0	0	-1.81	0	0
C5_05240C_A	0.35	0.49	0.32	0.62	0.25	0.15
C2_09360W_A	-0.28	0.17	0.08	0.21	0.29	0.18
C2_02740C_A	0.04	0.81	0.69	-0.79	0	0
C4_04510W_A	-0.19	0.14	0.06	-0.17	0.24	0.14
C3_07450C_A	0.05	0.82	0.71	0.1	0.65	0.52
C3_06440W_A	0.14	0.08	0.03	-0.01	0.91	0.87
C1_02920W_A	-0.14	0.22	0.11	-0.1	0.39	0.26
C4_00570C_A	-0.08	0.54	0.37	-0.03	0.84	0.76
C1_09290C_A	0.5	0.01	0	1.09	0	0
C1_04660W_A	0.7	0	0	0.12	0.39	0.26
C6_04270W_A	-0.16	0.08	0.03	-0.15	0.16	0.09
C2_05280C_A	-0.11	0.77	0.64	0.15	0.66	0.53
C4_06140C_A	0.71	0	0	0.88	0	0
C7_03200C_A	0.49	0	0	-0.09	0.52	0.38
C2_01450C_A	0.05	0.93	0.87	-0.45	0.42	0.29
CR_10420W_A	-0.48	0.02	0	-0.54	0.01	0
C3_04030C_A	0.07	0.72	0.59	0.15	0.41	0.28
C6_01190C_A	0.11	0.58	0.41	0.14	0.44	0.31
CR_09720W_A	-0.22	0.55	0.39	-0.02	0.96	0.94
CR_03100W_A	-0.16	0.36	0.21	-0.11	0.57	0.44
C7_02460C_A	-0.24	0.04	0.01	-0.3	0.01	0
CR_07630C_A	-0.39	0	0	-0.77	0	0
C4_02560C_A	-0.14	0.81	0.7	0.61	0.23	0.13
C4_03540C_A	0.21	0.3	0.17	0.09	0.69	0.57
C3_02220W_A	-0.15	0.33	0.19	0.05	0.74	0.63
CR_08120C_A	0	1	0.99	0.14	0.17	0.09
C2_07160W_A	0.05	0.75	0.62	0.05	0.76	0.65

CR_07210W_A	-0.01	0.96	0.93	0.35	0.01	0
C6_02800W_A	0.17	0.54	0.37	0.09	0.78	0.68
C3_05870C_A	-0.25	0.06	0.02	-0.09	0.55	0.41
C5_05260W_A	0.13	0.56	0.4	0.02	0.94	0.9
C1_03010W_A	-0.19	0	0	-0.76	0	0
C5_00750C_A	0.1	0.3	0.17	0.22	0.02	0.01
C4_05800C_A	0.38	0.32	0.18	0.21	0.66	0.53
C7_01540W_A	0.4	0.43	0.27	-0.03	0.97	0.95
CR_09330C_A	0.75	0	0	0.91	0	0
C7_00470C_A	0.21	0.09	0.04	0.5	0	0
C2_00390C_A	-0.41	0	0	-0.46	0	0
C3_03080W_A	0.3	0.06	0.02	0.32	0.05	0.02
C6_03690W_A	-0.07	0.74	0.61	-0.08	0.7	0.58
CR_10580W_A	0.28	0	0	0.14	0.2	0.12
C1_06030C_A	-0.34	0.24	0.12	0.2	0.48	0.35
CR_01590C_A	-0.23	0.03	0.01	-0.41	0	0
CR_07140C_A	-0.21	0.68	0.54	0.37	0.44	0.31
C6_03080C_A	-0.05	0.76	0.63	0	1	1
C3_07150C_A	-0.09	0.32	0.18	-0.7	0	0
C5_01540W_A	-0.06	0.42	0.26	-0.61	0	0
C1_07790C_A	-1.03	0	0	-0.6	0.07	0.03
C2_05320W_A	0.3	0.04	0.01	0.32	0.04	0.02
CR_04390C_A	0.09	0.56	0.39	0.21	0.12	0.06
C2_07570W_A	0.74	0	0	0.56	0	0
C2_03570C_A	0.78	0.03	0.01	1.42	0	0
C3_05220W_A	0.02	0.93	0.87	-0.14	0.38	0.25
CR_03020C_A	NA	NA	NA	NA	NA	NA
C2_10850C_A	0.4	0	0	0.5	0	0
C4_02030W_A	0.09	0.85	0.76	0.73	0.03	0.01
C2_02470C_A	NA	NA	NA	NA	NA	NA
C2_03070C_A	-0.2	0.39	0.24	-0.12	0.62	0.48
C1_02190W_A	-0.71	0.01	0	-0.79	0	0
CR_02800C_A	-0.37	0.05	0.02	-0.55	0	0
CR_04600W_A	-0.69	0	0	-0.83	0	0
CR_07580C_A	0.31	0.03	0.01	0.5	0	0
CR_09610C_A	-0.16	0.5	0.33	0.09	0.76	0.66
C3_06260C_A	0.22	0.14	0.06	0.33	0.04	0.02
CR_07680C_A	-0.39	0.08	0.03	-0.68	0	0
C7_03060C_A	-0.07	0.67	0.52	-0.12	0.44	0.3
C4_04190C_A	-0.18	0.68	0.54	0.48	0.16	0.08
C1_06820W_A	-0.32	0.04	0.01	0.23	0.09	0.04
C2_01000W_A	-0.19	0	0	-0.84	0	0
C4_04340C_A	-0.04	0.81	0.7	-0.22	0.11	0.06

C2_05970C_A	-0.25	0.37	0.22	-0.31	0.26	0.15
C4_06640C_A	0.14	0.53	0.36	0.26	0.49	0.35
C2_00720C_A	-0.27	0.08	0.03	-0.14	0.44	0.3
C3_04780C_A	0.34	0	0	-0.99	0	0
C1_12970C_A	0.37	0	0	0.3	0.02	0.01
CR_09010C_A	-0.17	0.05	0.02	-0.18	0.03	0.01
C5_03850W_A	-0.25	0.11	0.04	-0.04	0.83	0.76
C2_04030C_A	-0.11	0.5	0.33	-0.06	0.67	0.54
C2_07660W_A	0.04	0.8	0.68	-0.16	0.22	0.13
CR_10470C_A	-0.71	0	0	-0.47	0	0
C1_09870W_A	-0.42	0.15	0.07	0.22	0.48	0.34
C1_12030W_A	-0.22	0	0	-0.7	0	0
C3_05070W_A	-0.59	0.02	0.01	-0.56	0.03	0.01
C1_10720C_A	-0.14	0.3	0.17	-0.93	0	0
C2_05800C_A	0.1	0.6	0.44	0.54	0	0
C5_00040C_A	0.54	0	0	0.14	0.17	0.09
C6_03530C_A	-0.14	0.76	0.63	0.29	0.47	0.33
C1_08810C_A	-0.71	0	0	-0.81	0	0
C4_01740W_A	-0.08	0.85	0.76	-0.27	0.51	0.37
C4_02170C_A	-0.17	0.63	0.47	-0.13	0.73	0.62
C4_03180W_A	-0.2	0.01	0	-0.65	0	0
C1_09350W_A	-0.04	0.78	0.65	0.18	0.09	0.05
C2_05400W_A	-0.3	0.2	0.1	-0.27	0.25	0.14
C1_01110C_A	-0.59	0	0	-1.3	0	0
CR_01760C_A	0.04	0.67	0.52	-0.19	0.02	0.01
C3_07160W_A	-0.64	0	0	-0.76	0	0
C1_02090C_A	-0.21	0.32	0.18	-0.11	0.64	0.51
C7_03220C_A	0.06	0.62	0.45	-0.29	0	0
C2_03530W_A	0.45	0	0	0.51	0	0
CR_04190W_A	0.46	0	0	0.58	0	0
CR_00210W_A	-0.02	0.96	0.92	-0.09	0.74	0.63
C7_04070C_A	0.17	0.65	0.49	0.84	0.15	0.08
C4_06540W_A	-0.31	0.01	0	-0.31	0.02	0.01
C7_03340C_A	-0.52	0.07	0.03	-1.19	0	0
BGI_novel_G000	0.45	0	0	1.08	0	0
C5_03260C_A	0.08	0.75	0.62	0.14	0.55	0.41
C4_03300C_A	-0.27	0.43	0.27	-0.23	0.72	0.6
C1_11260C_A	NA	NA	NA	NA	NA	NA
C2_08720W_A	-0.6	0	0	-0.12	0.81	0.73
C7_01290W_A	0.21	0.31	0.17	0.45	0.01	0
C3_02840W_A	-0.4	0.36	0.21	-0.59	0.19	0.11
C1_02650W_A	0.46	0	0	0.54	0	0
C2_01780W_A	0.14	0.24	0.13	-0.51	0	0

CR_00200W_A	0.22	0.02	0.01	0.05	0.74	0.63
C2_10770W_A	-0.07	0.81	0.7	-0.18	0.52	0.38
C5_03390C_A	0.07	0.58	0.42	-0.12	0.27	0.17
C1_06610C_A	0.56	0.15	0.07	0.6	0.16	0.08
C1_01160C_A	-0.61	0	0	-0.44	0.03	0.01
C2_00440W_A	-0.06	0.78	0.66	0.11	0.59	0.46
CR_06020W_A	-0.15	0.68	0.53	-0.02	0.96	0.93
C1_07430W_A	-0.42	0.29	0.16	-0.57	0.35	0.23
C1_07820W_A	-0.13	0.54	0.38	-0.37	0.05	0.02
C1_02670C_A	-0.05	0.94	0.88	1.09	0.03	0.01
C2_09800C_A	-0.01	0.98	0.96	-0.42	0.33	0.21
C1_03230C_A	-1.02	0	0	-1.63	0	0
C1_07340W_A	-0.27	0	0	-0.39	0	0
C2_06260W_A	-0.01	0.99	0.97	0.23	0.59	0.46
C1_04280C_A	0.31	0.11	0.05	0.24	0.24	0.14
C2_02970C_A	0.22	0.02	0	0.42	0	0
C5_03040W_A	0.07	0.79	0.68	0.31	0.14	0.07
C1_08170C_A	0.37	0.05	0.02	1.03	0	0
C1_07780W_A	0.14	0.25	0.13	0.11	0.42	0.29
C2_03760C_A	0.21	0.06	0.02	-0.06	0.69	0.57
C5_02450W_A	0.11	0.56	0.39	0.05	0.8	0.71
C1_03520W_A	0.25	0	0	0.38	0	0
C4_02420C_A	-0.41	0.03	0.01	-0.49	0.01	0
CR_10360C_A	0.77	0	0	0.79	0	0
C2_03400C_A	0.38	0.01	0	-0.08	0.71	0.59
C3_07210W_A	0.11	0.44	0.27	0.13	0.32	0.21
C1_02820W_A	0.45	0	0	0.42	0	0
C2_04710C_A	-0.07	0.69	0.54	-0.14	0.37	0.24
C2_07000W_A	-0.15	0.42	0.26	-0.27	0.12	0.06
C5_05090W_A	-0.18	0.38	0.23	-0.03	0.91	0.86
C1_00470C_A	0.24	0.18	0.08	0.59	0	0
C1_02210W_A	-0.42	0	0	-0.58	0	0
C5_00540C_A	-0.07	0.68	0.53	-0.13	0.65	0.52
C1_02150W_A	0.91	0	0	1.08	0	0
CR_08650C_A	0.15	0.15	0.07	-0.08	0.54	0.4
C4_03680C_A	-0.42	0.16	0.08	-0.59	0.08	0.04
C7_03980W_A	-0.04	0.84	0.74	-0.23	0.21	0.12
CR_07340C_A	0	0.99	0.97	-0.09	0.57	0.43
CR_02970C_A	-0.16	0.16	0.07	-0.16	0.2	0.11
CR_09790W_A	0.34	0	0	0.24	0	0
C1_10390C_A	-0.45	0	0	-0.6	0	0
C1_05760C_A	-0.05	0.93	0.87	-0.11	0.89	0.83
CR_08370W_A	-0.49	0	0	-0.37	0	0

C7_00300W_A	-0.02	0.9	0.82	-0.04	0.81	0.73
CR_06290C_A	-0.16	0.19	0.09	-0.29	0.02	0.01
C2_07900W_A	-0.13	0.33	0.19	-0.31	0	0
C5_04590C_A	-0.09	0.71	0.57	-0.6	0	0
C2_09000C_A	-0.28	0.52	0.36	0.31	0.44	0.31
C2_05540C_A	-0.25	0.1	0.04	-0.23	0.18	0.1
C4_05870C_A	0.97	0.02	0.01	1.45	0	0
C3_07370W_A	-0.2	0.07	0.03	-0.41	0	0
C2_07350W_A	-0.11	0.28	0.15	0.05	0.71	0.59
C2_08020C_A	0.31	0.01	0	0.49	0	0
C7_02150C_A	0.61	0	0	1.27	0	0
C7_01560C_A	-0.92	0	0	-3.05	0	0
C2_03260W_A	-0.07	0.63	0.47	-0.13	0.29	0.18
C1_13690C_A	0.31	0.23	0.12	0.5	0.04	0.02
C5_05000C_A	-0.35	0.01	0	-0.3	0.02	0.01
CR_07240C_A	0.32	0.01	0	0.23	0.1	0.05
C3_03420C_A	0.23	0	0	0.24	0.01	0
CR_00150C_A	0.66	0	0	0.92	0	0
C1_01710W_A	0.14	0.5	0.34	0.28	0.15	0.08
C1_11080W_A	0.07	0.61	0.45	0.35	0	0
C5_02940C_A	0.16	0.08	0.03	0.26	0.01	0
C1_00880W_A	-0.86	0	0	-0.98	0	0
C1_04090C_A	-0.11	0.64	0.48	0.04	0.85	0.78
C1_08210C_A	-0.26	0.01	0	-0.32	0	0
C5_00780C_A	-0.41	0.04	0.01	-0.11	0.65	0.52
C1_13860C_A	0.25	0	0	0.26	0	0
C3_07660W_A	-0.43	0.01	0	-0.3	0.08	0.04
C4_03710C_A	0.26	0.48	0.32	0.69	0.02	0.01
C3_05340W_A	-0.01	0.98	0.96	-0.23	0.42	0.28
C7_01320W_A	0.13	0.42	0.26	0.2	0.1	0.05
CR_07910C_A	-0.11	0.5	0.33	-0.14	0.38	0.26
BGI_novel_G000	-0.02	0.92	0.86	-0.24	0.12	0.06
C1_13060C_A	0.19	0.01	0	-0.07	0.55	0.41
C2_07820C_A	-0.25	0.11	0.05	-0.52	0	0
C3_06510C_A	0.42	0.4	0.25	0.47	0.45	0.31
C2_03800C_A	0.28	0.01	0	0.71	0	0
C3_01890C_A	-0.12	0.28	0.15	0.15	0.22	0.12
C2_00170C_A	-0.08	0.67	0.52	-0.52	0	0
CR_05620C_A	-0.34	0.02	0.01	-0.35	0.02	0.01
C1_05810W_A	-0.13	0.44	0.28	-0.18	0.26	0.16
C2_09160W_A	-0.34	0.08	0.03	-0.2	0.37	0.25
C5_04430C_A	0.1	0.35	0.2	0.09	0.42	0.28
C1_13830C_A	-0.11	0.18	0.08	-0.13	0.15	0.08

C1_00280C_A	NA	NA	NA	NA	NA	NA
C2_08760C_A	-0.33	0	0	-0.31	0.22	0.13
C2_02890W_A	0.09	0.52	0.35	0.08	0.61	0.48
CR_01340W_A	0.16	0.78	0.65	0.05	0.94	0.9
C1_14220C_A	0.24	0.04	0.02	-0.82	0	0
C2_04370W_A	-0.19	0.08	0.03	-0.33	0	0
C1_14360C_A	0.03	0.85	0.76	-0.15	0.23	0.13
C5_04260W_A	0.57	0	0	-0.06	0.83	0.75
CR_00560W_A	0.7	0	0	0.44	0	0
C1_12270W_A	0.09	0.81	0.69	0.35	0.24	0.14
C2_00860C_A	0.13	0.8	0.69	0.55	0.2	0.11
C1_05410C_A	0.2	0.14	0.06	0.27	0.04	0.02
C2_02680W_A	-0.38	0	0	-0.56	0	0
CR_06080W_A	-0.25	0	0	-0.21	0.02	0.01
C5_04270C_A	0.1	0.66	0.5	-0.23	0.03	0.01
CR_01790C_A	-0.07	0.78	0.66	0.25	0.17	0.09
C1_12510W_A	0.15	0.35	0.2	-0.16	0.36	0.24
C4_07110C_A	0.52	0	0	0.43	0	0
C1_01630W_A	-0.2	0.34	0.2	0.17	0.35	0.23
C2_00850W_A	-0.05	0.68	0.53	-0.17	0.12	0.06
C3_00370C_A	-0.25	0.18	0.09	-0.01	0.99	0.97
C1_03160C_A	-0.13	0.22	0.11	-0.15	0.16	0.08
C3_02200W_A	0.27	0.33	0.19	0.12	0.67	0.55
C2_04790C_A	-0.09	0.49	0.32	-0.2	0.09	0.04
C7_04320W_A	0.43	0.37	0.22	0.74	0.13	0.07
CR_10180W_A	-0.15	0.74	0.6	-0.24	0.59	0.45
C7_01030C_A	-1.02	0	0	-1.57	0	0
C6_01950C_A	-0.26	0.03	0.01	0.13	0.34	0.22
C6_02030C_A	-0.66	0	0	-0.53	0	0
CR_02550C_A	-0.46	0.01	0	-0.08	0.72	0.61
C5_00590W_A	0.48	0	0	0.58	0	0
C1_04010C_A	1.44	0	0	3.53	0	0
CR_03750C_A	0.15	0.41	0.26	0.25	0.18	0.1
C2_00060C_A	0.17	0.03	0.01	-0.15	0.13	0.07
C2_06250C_A	-0.02	0.92	0.85	0.04	0.8	0.71
C1_14410W_A	0.29	0.23	0.12	0.23	0.31	0.2
CR_08070W_A	-0.38	0	0	-0.57	0	0
C7_00070C_A	0.03	0.82	0.71	-0.01	0.92	0.88
C7_03710C_A	-0.06	0.78	0.65	0.1	0.63	0.5
C5_03860W_A	-0.16	0.2	0.1	-0.14	0.26	0.16
C4_00410W_A	0.37	0	0	0.78	0	0
C3_06410C_A	0.16	0.39	0.24	-0.15	0.43	0.3
C2_01610C_A	-0.02	0.81	0.7	-0.4	0	0

C5_03510C_A	0.11	0.23	0.12	-0.28	0	0
C3_00710W_A	-0.37	0	0	-0.35	0	0
C2_00150W_A	0.29	0.05	0.02	0.59	0	0
C2_08320C_A	-0.9	0.01	0	-1.08	0	0
C2_04650C_A	-0.24	0.47	0.3	-0.09	0.82	0.75
C4_05040W_A	-0.19	0.71	0.57	-0.05	0.94	0.91
C5_02640W_A	-0.3	0.07	0.03	-0.41	0.01	0
CR_01690C_A	0.07	0.37	0.22	-0.22	0.01	0
C2_01020W_A	0.22	0	0	-0.39	0	0
C1_06650W_A	-0.17	0.32	0.18	-0.11	0.49	0.35
C1_09470C_A	0.22	0.03	0.01	0.13	0.27	0.16
C2_06030W_A	-0.07	0.7	0.55	-0.01	0.95	0.91
C7_01580W_A	-0.5	0	0	-0.23	0.2	0.11
C3_06300W_A	-0.33	0	0	-0.33	0.03	0.01
C1_11550W_A	-0.14	0.11	0.05	-0.91	0	0
C3_03550C_A	-0.27	0.14	0.06	-0.43	0.01	0
C3_01780C_A	-0.02	0.94	0.9	0.04	0.88	0.82
C6_01530C_A	NA	NA	NA	NA	NA	NA
CR_02270C_A	-0.37	0.28	0.15	-0.57	0.08	0.04
BGI_novel_G000	-0.07	0.87	0.78	0.35	0.58	0.45
C1_10790W_A	0.69	0	0	0.87	0	0
C1_07480C_A	-0.04	0.94	0.89	-0.14	0.8	0.71
C3_00860W_A	-0.33	0.1	0.04	-0.55	0	0
C1_12600C_A	-0.73	0	0	-0.26	0.22	0.13
C1_05060W_A	-1.34	0	0	-1.29	0	0
CR_09420C_A	0.06	0.8	0.69	-0.11	0.62	0.49
C3_01530C_A	-0.16	0.11	0.05	0.2	0.04	0.02
C3_07970C_A	-0.12	0.42	0.26	-0.13	0.4	0.27
C4_01160W_A	0.81	0	0	0.39	0.03	0.01
C7_03850W_A	-1.76	0	0	-1.66	0	0
C3_00250C_A	0.07	0.35	0.2	-0.2	0.01	0
C1_02060W_A	-0.26	0.45	0.29	0.12	0.73	0.62
C3_02830W_A	-0.37	0.01	0	-0.39	0	0
C1_05940W_A	0.54	0	0	0.13	0.23	0.13
CR_02700W_A	-0.56	0.01	0	-0.44	0.06	0.03
CR_02020C_A	0.25	0.01	0	-1.45	0	0
C1_13610C_A	0.05	0.74	0.61	-0.17	0.21	0.12
C2_03280W_A	-0.05	0.64	0.47	-0.12	0.22	0.13
C2_09880C_A	-0.22	0.63	0.47	0.51	0.44	0.31
C5_04950C_A	-0.04	0.81	0.69	-0.16	0.23	0.14
CR_10290C_A	0.06	0.68	0.54	0.35	0	0
C1_05260C_A	0.04	0.75	0.62	-0.1	0.43	0.3
C1_13470W_A	0.1	0.73	0.59	0.53	0.01	0

C4_06430C_A	-0.18	0.49	0.32	-0.03	0.92	0.87
C1_04840C_A	-0.2	0.52	0.35	-0.48	0.09	0.04
C1_04410C_A	-0.25	0.03	0.01	-0.26	0.03	0.01
CR_01180W_A	0.04	0.77	0.65	0.28	0.02	0.01
C5_04010C_A	-0.12	0.84	0.74	0.27	0.69	0.57
C6_00330C_A	0.34	0	0	-0.28	0	0
C4_00300C_A	0.1	0.44	0.28	0.08	0.56	0.42
C3_02750W_A	0.13	0.77	0.64	0.65	0.29	0.18
C7_01170C_A	-0.28	0.58	0.41	-0.58	0.36	0.24
C6_02240C_A	-0.04	0.67	0.52	-0.53	0	0
C7_02660C_A	-0.27	0.23	0.12	0	0.99	0.98
CR_05300C_A	0.57	0	0	0.61	0	0
C6_01870C_A	0.23	0.19	0.09	-0.28	0.12	0.06
C1_14180W_A	0.04	0.87	0.78	0.01	0.98	0.96
C2_01950C_A	-0.89	0	0	-0.83	0	0
C3_00320W_A	0.47	0	0	0.42	0	0
C4_04860W_A	-0.8	0	0	-0.6	0	0
C1_00190C_A	0.17	0.68	0.53	0.19	0.78	0.68
C1_06440C_A	-0.2	0.13	0.06	0.01	0.95	0.91
C6_01040C_A	-0.64	0	0	-0.38	0.08	0.04
C7_04190C_A	-1.08	0	0	-0.94	0	0
C3_07790W_A	0.08	0.72	0.58	0.02	0.93	0.89
C2_06910W_A	-0.25	0.09	0.03	-0.37	0.01	0
C1_13560W_A	-0.36	0.06	0.02	-0.26	0.18	0.1
C2_06790W_A	-0.04	0.94	0.9	-0.06	0.91	0.86
C5_05290C_A	-0.01	0.95	0.91	-0.11	0.53	0.39
C4_01650C_A	-0.01	0.97	0.94	0.13	0.42	0.28
C1_03400W_A	-0.41	0	0	-0.96	0	0
CR_09580C_A	-0.18	0.18	0.09	-0.05	0.77	0.67
C5_03460C_A	0.16	0.44	0.28	-0.04	0.87	0.81
C3_06740W_A	-0.46	0	0	-0.03	0.89	0.83
C1_01980W_A	-0.08	0.83	0.73	-0.09	0.79	0.7
C3_01410C_A	0.23	0.09	0.04	0.07	0.69	0.58
C2_01800W_A	0.05	0.94	0.9	-0.05	0.95	0.91
C2_03970W_A	0.24	0.22	0.11	0.67	0	0
C3_00680C_A	NA	NA	NA	NA	NA	NA
C1_06360W_A	-0.09	0.69	0.54	-0.12	0.55	0.42
C6_02260C_A	0.28	0	0	0.53	0	0
C2_03320W_A	0.31	0.04	0.01	0.68	0	0
C3_02610C_A	-0.11	0.22	0.11	0.65	0	0
CR_09460C_A	0.13	0.71	0.56	0.22	0.49	0.36
C5_05040W_A	0.48	0	0	0.19	0.17	0.09
CR_08840C_A	-0.49	0	0	-0.7	0	0

C7_00170W_A	-0.01	0.95	0.9	-1.11	0	0
C4_03490C_A	0.13	0.82	0.71	0.24	0.74	0.63
CR_07840C_A	0.25	0.33	0.19	0.2	0.46	0.33
C6_03920W_A	0.16	0.14	0.06	0.25	0.01	0
C3_01590W_A	0.12	0.53	0.36	0.14	0.4	0.27
C5_03130W_A	-1.02	0	0	-1.85	0	0
C2_08840W_A	0.1	0.66	0.5	-0.01	0.96	0.94
C1_09830W_A	0.13	0.27	0.14	0.09	0.44	0.31
C5_04090C_A	-0.06	0.78	0.66	-0.03	0.87	0.81
C1_05660C_A	0.28	0	0	0.03	0.79	0.69
C1_14130W_A	0.41	0.09	0.04	-0.43	0.14	0.07
C2_09690C_A	-0.01	0.98	0.96	0.22	0.14	0.07
C1_14310W_A	0.05	0.78	0.66	0	0.99	0.98
C7_03590C_A	-0.31	0.12	0.05	-0.45	0.02	0.01
C4_06590W_A	-0.6	0.01	0	-0.39	0.12	0.06
C3_02550C_A	0.45	0.01	0	0.73	0	0
C4_00690C_A	0.22	0.21	0.1	0.35	0.03	0.01
C1_01350C_A	0.05	0.56	0.4	-0.35	0	0
C5_03430W_A	0.03	0.97	0.93	0.82	0.11	0.05
C3_04130W_A	0.73	0	0	0.83	0	0
C5_00680W_A	-0.6	0	0	-0.61	0	0
C7_01360C_A	-0.39	0.34	0.2	-0.48	0.25	0.15
C2_09680W_A	0.46	0	0	0.82	0	0
C4_04880W_A	0.23	0.5	0.33	0.45	0.14	0.07
C1_12140W_A	0.64	0	0	1.08	0	0
C2_10840W_A	0.08	0.67	0.52	-0.01	0.98	0.97
C2_04400W_A	0.24	0.08	0.03	0.17	0.25	0.15
C1_05340C_A	-0.68	0	0	-1.08	0	0
C6_02530C_A	0.15	0.42	0.26	0.19	0.28	0.17
C2_10320C_A	0.03	0.94	0.89	0.09	0.83	0.75
C1_08490W_A	0.38	0	0	0.19	0.06	0.03
C6_02720C_A	0.18	0.2	0.1	0.28	0.03	0.01
C3_04000C_A	0.1	0.84	0.73	0.13	0.8	0.71
C2_02100W_A	-0.18	0.12	0.05	-0.12	0.38	0.25
C4_07150W_A	-0.24	0.55	0.39	0.07	0.87	0.81
C5_00400C_A	-0.84	0	0	-0.68	0	0
C1_12360C_A	0.08	0.35	0.2	-0.19	0.05	0.02
C4_03050C_A	-0.72	0	0	-2.33	0	0
C3_07390C_A	-0.42	0	0	-0.28	0.01	0
CR_02500W_A	-0.11	0.64	0.48	-0.29	0.17	0.09
C3_00240C_A	0.64	0	0	0.89	0	0
CR_09680C_A	2.11	0	0	3.27	0	0
C3_00310C_A	0.54	0	0	0.5	0	0

C2_04590C_A	0.37	0.01	0	0.39	0.01	0
C4_00250W_A	NA	NA	NA	NA	NA	NA
C5_00480C_A	0.09	0.33	0.19	0	0.98	0.96
C2_00500W_A	0.06	0.78	0.66	0.14	0.45	0.32
CR_00280C_A	-0.11	0.54	0.37	0.22	0.16	0.08
C1_01860W_A	-0.04	0.74	0.6	-0.16	0.18	0.1
C2_02180W_A	0.02	0.94	0.9	0.27	0.18	0.1
C6_02710C_A	0.43	0.37	0.22	0.12	0.86	0.79
CR_08900C_A	0.27	0	0	0.25	0.03	0.01
C1_04710C_A	-0.32	0.3	0.17	0.1	0.76	0.65
C2_06710W_A	0.07	0.86	0.77	0.52	0.04	0.02
C2_05590C_A	0.42	0	0	0.41	0	0
C2_07930C_A	0.52	0	0	0.62	0	0
C6_00710W_A	0.18	0.37	0.22	0.04	0.88	0.82
CR_03820C_A	0.01	0.97	0.94	-0.03	0.9	0.85
CR_04140W_A	-0.09	0.29	0.16	-0.27	0	0
C1_04530C_A	-0.04	0.84	0.74	0.25	0.09	0.04
C1_09600C_A	-0.02	0.96	0.92	-0.07	0.74	0.63
CR_06430W_A	-0.1	0.82	0.72	0.21	0.64	0.51
C3_00590W_A	0.1	0.61	0.45	NA	NA	NA
C4_06160W_A	0.46	0.01	0	0.52	0	0
C7_00130W_A	0.63	0	0	0.95	0	0
C1_12340C_A	-0.29	0	0	-0.27	0	0
C1_09630W_A	0.07	0.48	0.31	-0.47	0	0
C2_09570C_A	-0.04	0.94	0.89	0.41	0.28	0.17
C1_12400C_A	-0.18	0.43	0.27	0.3	0.1	0.05
C4_01070W_A	NA	NA	NA	0.26	0.49	0.35
C1_10420C_A	0	0.99	0.98	0.02	0.87	0.81
C2_02290C_A	0.06	0.87	0.78	0.33	0.24	0.14
C6_00150W_A	0.51	0	0	0.59	0	0
C7_00760C_A	0	1	1	-0.36	0.36	0.24
C1_00790W_A	-0.69	0.01	0	-0.94	0	0
C1_06100C_A	0.02	0.82	0.72	-0.36	0	0
C1_09380W_A	0.06	0.67	0.52	-0.11	0.41	0.28
C3_07470W_A	-0.09	0.85	0.76	-0.17	0.73	0.62
C4_06960W_A	-0.11	0.82	0.71	-0.39	0.35	0.23
CR_03140C_A	0.55	0	0	0.67	0	0
CR_04870C_A	-0.16	0.63	0.47	0.35	0.21	0.12
C4_04460C_A	-0.02	0.88	0.8	0.26	0.02	0.01
C1_00850W_A	-0.07	0.8	0.69	0.83	0	0
C4_01000C_A	0.28	0.57	0.41	0.12	0.87	0.8
CR_04660C_A	0.49	0	0	0.7	0	0
C3_01280W_A	0.12	0.57	0.41	0.09	0.7	0.59

C2_09590C_A	-1.01	0	0	-1	0	0
CR_02150W_A	0.28	0.01	0	0.34	0	0
C1_05970W_A	0.18	0.74	0.6	0.04	0.96	0.93
C1_06380C_A	-0.57	0.07	0.03	-0.07	0.85	0.79
CR_01290C_A	0.22	0.01	0	-0.24	0.01	0
C6_02100W_A	1.98	0	0	2.41	0	0
C6_04230W_A	0.58	0.15	0.07	-0.23	0.7	0.58
C2_03920C_A	-0.18	0.32	0.18	0.01	0.97	0.96
CR_03420C_A	0.01	0.97	0.93	0.1	0.35	0.22
C4_06830C_A	0.02	0.81	0.7	-0.11	0.23	0.13
C5_04120C_A	-0.06	0.87	0.78	0.28	0.35	0.23
C7_02350C_A	0.1	0.58	0.42	0.39	0	0
C3_06590W_A	0.26	0	0	0.6	0	0
C4_05840W_A	-0.07	0.84	0.74	-0.43	0.11	0.05
C4_01770W_A	-0.05	0.62	0.46	-0.31	0	0
C3_04080W_A	0.34	0	0	0.51	0	0
C4_04450C_A	0.2	0.27	0.14	0.61	0	0
CR_06750C_A	0.24	0	0	0.53	0	0
C2_10030C_A	0.09	0.28	0.15	-0.74	0	0
C2_10200W_A	0.47	0	0	0.67	0	0
CR_02120C_A	0.01	0.99	0.97	0.35	0.06	0.03
C3_05310W_A	-0.25	0.34	0.2	-0.21	0.64	0.5
C1_12740W_A	-0.09	0.55	0.38	-0.2	0.14	0.07
C1_02320C_A	0.25	0.03	0.01	0.14	0.3	0.19
C2_09140C_A	-0.7	0	0	-1.22	0	0
C1_03580W_A	NA	NA	NA	NA	NA	NA
C3_06690C_A	0.49	0	0	0.7	0	0
C3_04510W_A	-0.69	0.12	0.05	-0.29	0.65	0.52
CR_07170W_A	0.06	0.84	0.75	-0.02	0.94	0.9
C7_02890C_A	1.07	0	0	0.35	0	0
C1_11360W_A	-0.11	0.27	0.14	-0.44	0	0
C2_06200C_A	-0.15	0.34	0.2	-0.05	0.77	0.67
C1_06020W_A	-0.07	0.66	0.5	-0.01	0.97	0.95
C3_03110W_A	-0.41	0.03	0.01	-0.61	0	0
C3_01110C_A	-0.46	0.29	0.16	-0.29	0.57	0.43
C1_06450C_A	-0.01	0.94	0.89	-0.48	0	0
C1_05700W_A	0.42	0.01	0	0.81	0	0
C7_03500W_A	-0.19	0.32	0.18	-0.39	0.03	0.01
C2_09470C_A	-0.23	0.26	0.14	0	0.99	0.98
C5_02760W_A	-0.11	0.59	0.42	-0.16	0.41	0.28
C3_06330W_A	0.44	0	0	0.48	0	0
C1_11170W_A	0.48	0	0	0.07	0.5	0.36
C5_04360C_A	-0.23	0.03	0.01	-0.41	0	0

C7_01430C_A	-0.63	0.13	0.06	-0.44	0.38	0.25
C1_12690C_A	-0.13	0.69	0.54	0.11	0.74	0.63
C1_04460C_A	0.75	0	0	1.39	0	0
C7_00950W_A	-0.14	0.21	0.1	-0.46	0	0
CR_06260W_A	0.18	0.31	0.17	0.19	0.3	0.18
C2_05040C_A	0.45	0	0	1	0	0
CR_09290W_A	-0.22	0.33	0.19	-0.32	0.14	0.07
C5_01000C_A	-0.1	0.54	0.37	0.01	0.94	0.91
C5_02030W_A	0.1	0.31	0.17	0.36	0	0
C7_02970W_A	-0.2	0.08	0.03	-1.18	0	0
CR_05250C_A	0.06	0.89	0.81	-0.09	0.81	0.72
C2_03470C_A	0.13	0.16	0.08	0.06	0.62	0.49
C4_02360W_A	0.7	0	0	1.6	0	0
C3_04440C_A	0.57	0.21	0.1	0.5	0.38	0.25
C1_00960C_A	-0.48	0	0	-0.14	0.5	0.36
C1_05440C_A	-0.07	0.87	0.79	0.32	0.39	0.26
C5_01730W_A	-0.28	0.27	0.14	-0.12	0.67	0.54
C2_00570W_A	0.08	0.44	0.28	-0.08	0.5	0.37
C1_07570C_A	0.04	0.93	0.87	-0.31	0.35	0.23
C3_03600C_A	0.25	0.56	0.4	0.44	0.49	0.35
CR_03590C_A	0.3	0.51	0.34	0.29	0.58	0.44
C7_02520W_A	-0.49	0.14	0.06	-0.58	0.08	0.04
CR_00420W_A	0.37	0.13	0.05	0.54	0.02	0.01
C5_03770C_A	0.12	0.82	0.71	0.36	0.6	0.47
C3_07740W_A	0.02	0.91	0.84	0.14	0.27	0.16
C2_03370W_A	0.95	0	0	1.28	0	0
C1_07710C_A	-0.61	0	0	-1.17	0	0
C7_03730C_A	-0.12	0.54	0.37	0.06	0.79	0.69
C1_05880W_A	-0.22	0.25	0.13	-0.34	0.06	0.03
CR_08280W_A	0.16	0.41	0.25	0.22	0.25	0.15
C3_00980W_A	0.61	0	0	0.95	0	0
CR_07470W_A	-0.3	0.03	0.01	-0.08	0.62	0.49
C6_00290W_A	0.13	0.1	0.04	-0.02	0.84	0.76
C1_03130C_A	0.16	0.03	0.01	0.15	0.08	0.04
C1_02390W_A	-0.21	0.28	0.15	0.18	0.3	0.19
C7_03830C_A	-0.13	0.49	0.33	-0.3	0.07	0.03
C1_10780C_A	0.13	0.35	0.2	0.56	0	0
C2_10260C_A	0.29	0	0	0.42	0	0
CR_04530W_A	0.14	0.13	0.06	0	0.99	0.97
C2_10000C_A	0.27	0.55	0.38	0.08	0.89	0.84
C4_03130W_A	0.66	0	0	0.08	0.56	0.43
BGI_novel_G000	-0.11	0.71	0.57	-0.06	0.84	0.77
CR_01950W_A	-0.32	0.02	0.01	-0.51	0	0

CR_06870C_A	0.44	0.37	0.22	0.27	0.68	0.56
CR_06040W_A	0.09	0.57	0.4	0.49	0	0
C3_03740W_A	-0.51	0.02	0.01	-0.34	0.14	0.07
C5_05160C_A	0.16	0.22	0.11	0.27	0.03	0.01
C1_01890C_A	-0.29	0	0	-0.35	0	0
C3_03450C_A	1.08	0	0	1.82	0	0
C1_00560W_A	-0.38	0.01	0	0.01	0.95	0.92
C1_00640C_A	-0.26	0	0	-0.4	0	0
C1_10330C_A	-0.16	0.42	0.26	-0.09	0.67	0.55
CR_07020W_A	0.08	0.55	0.39	0.02	0.93	0.89
CR_05680C_A	0.06	0.92	0.86	0.01	0.99	0.98
C2_06160W_A	0.06	0.9	0.83	0.44	0.25	0.15
C1_10380C_A	-0.04	0.72	0.59	0.04	0.76	0.65
C6_03290W_A	-0.43	0	0	-0.5	0	0
BGI_novel_G000	0.25	0.26	0.14	0.64	0	0
C5_03370C_A	-0.05	0.86	0.77	0.29	0.24	0.14
C2_02960C_A	0.08	0.67	0.52	0.13	0.48	0.34
C6_04120C_A	-0.46	0.02	0.01	-0.43	0.05	0.02
C1_01520C_A	0.16	0.14	0.06	0.52	0	0
C5_02280C_A	0.01	0.97	0.93	0.09	0.69	0.57
C3_02490C_A	-0.14	0.36	0.21	-0.34	0.04	0.02
CR_01280C_A	-0.04	0.9	0.83	-0.03	0.92	0.88
C2_06440C_A	-0.25	0.44	0.28	-0.62	0.04	0.02
C4_02460W_A	0.84	0	0	0.95	0	0
C1_00510W_A	-0.36	0	0	-0.54	0	0
C7_04010W_A	-0.11	0.31	0.17	-0.03	0.83	0.76
CR_03330W_A	-0.1	0.62	0.46	0.11	0.59	0.45
C2_08890W_A	-0.27	0.32	0.18	-0.23	0.69	0.57
CR_01640C_A	-1.41	0	0	-1.3	0	0
C5_01830C_A	0.09	0.69	0.54	0.05	0.82	0.74
C2_00610C_A	0.31	0.22	0.11	0.9	0	0
C1_03740W_A	-0.15	0.5	0.33	-0.56	0.01	0
C1_12620W_A	-0.26	0.04	0.01	-0.27	0.07	0.03
BGI_novel_G000	0.94	0	0	1.38	0	0
C4_04330C_A	0.22	0.01	0	0.62	0	0
C1_12640W_A	-0.11	0.71	0.56	-0.1	0.73	0.62
CR_05500C_A	0.23	0.04	0.02	0	0.99	0.98
C3_06470W_A	0.53	0	0	0.75	0	0
C1_01690C_A	0.29	0	0	0.15	0.13	0.07
CR_06670W_A	0.17	0.65	0.49	0.29	0.63	0.5
C3_02170C_A	0.06	0.57	0.41	0.21	0.02	0.01
C4_00750C_A	-0.19	0.54	0.38	-0.25	0.46	0.33
C5_01260W_A	0.07	0.9	0.83	0.81	0.07	0.03

CR_05960W_A	-0.02	0.97	0.94	0.07	0.82	0.74
C1_11890W_A	0.44	0	0	0.97	0	0
C7_00290C_A	-0.33	0.51	0.35	0.14	0.81	0.72
C2_04980C_A	-0.31	0.03	0.01	-0.18	0.27	0.16
C4_04410C_A	0.44	0	0	0.5	0	0
C4_04320W_A	-0.01	0.97	0.93	-0.35	0.06	0.03
C1_04150C_A	0.35	0.1	0.04	0.52	0.01	0
C5_01440C_A	0.35	0.06	0.02	1.03	0	0
C1_10710C_A	-0.47	0	0	-0.79	0	0
CR_00920W_A	-0.44	0.22	0.11	-0.6	0.11	0.05
C2_01320W_A	0.22	0.01	0	0.27	0	0
C3_06900W_A	-0.08	0.7	0.55	-0.01	0.98	0.97
C1_09420W_A	0.2	0.43	0.27	NA	NA	NA
C1_08990C_A	0.36	0	0	0.42	0	0
C1_08770W_A	-0.01	0.98	0.95	0.09	0.73	0.62
C4_02440C_A	-0.51	0	0	-1.1	0	0
CR_06240C_A	-0.04	0.95	0.91	-0.01	0.99	0.98
C2_07130C_A	-0.19	0.02	0.01	-0.21	0.02	0.01
CR_07740W_A	0.07	0.67	0.52	0.36	0.38	0.25
C3_05710W_A	1.49	0	0	2.27	0	0
CR_08440W_A	0.21	0.44	0.28	0.12	0.67	0.55
C1_08590C_A	0.02	0.81	0.7	-0.55	0	0
C1_13280C_A	-0.06	0.7	0.55	-0.11	0.44	0.31
C1_03100W_A	0.04	0.69	0.55	-0.28	0	0
C1_14000C_A	0.25	0.02	0.01	0.28	0.01	0
C4_03740W_A	-0.57	0	0	-0.59	0	0
CR_02580W_A	-0.43	0	0	-0.47	0	0
C1_12160W_A	0.22	0.29	0.16	0.38	0.04	0.02
C2_08380C_A	-0.12	0.46	0.3	0.08	0.69	0.58
C3_02480C_A	0.12	0.07	0.03	0.24	0	0
C4_00530C_A	0.09	0.88	0.79	0.08	0.91	0.87
C2_00760C_A	0.7	0	0	1.1	0	0
C4_05560C_A	-0.18	0.42	0.26	0.02	0.93	0.89
C3_04940W_A	0.67	0	0	0.88	0	0
C1_01260C_A	0	0.99	0.98	0	0.99	0.99
C4_07050W_A	-0.76	0	0	-0.72	0	0
C4_01340W_A	NA	NA	NA	NA	NA	NA
CR_00090C_A	0.76	0	0	1.22	0	0
C3_06320W_A	0.32	0.18	0.08	0.08	0.8	0.71
C6_01840C_A	0.24	0.05	0.02	0.08	0.58	0.44
C2_08940C_A	0.11	0.64	0.47	-0.01	0.97	0.95
C3_05920W_A	-0.21	0.22	0.11	0.19	0.26	0.16
C3_07560W_A	-0.07	0.52	0.35	-0.55	0	0

C5_00290W_A	-0.1	0.67	0.51	0.1	0.66	0.54
C3_02110W_A	-0.04	0.66	0.51	-0.63	0	0
C2_07500C_A	-0.17	0.4	0.25	0.04	0.87	0.81
CR_02620C_A	0.08	0.72	0.59	0.12	0.57	0.44
CR_03380W_A	-0.7	0.01	0	-0.36	0.19	0.1
CR_01740W_A	0.33	0	0	0.4	0	0
CR_07720C_A	0.12	0.48	0.31	-0.18	0.26	0.16
C3_06760W_A	-1.22	0	0	-2.21	0	0
C6_02780C_A	-0.12	0.52	0.35	-0.28	0.08	0.04
C3_05760W_A	-0.09	0.56	0.39	-0.21	0.13	0.07
C2_08450W_A	-0.14	0.42	0.26	0.23	0.14	0.08
CR_05910W_A	-0.41	0	0	-0.34	0.01	0.01
C2_08950W_A	0.21	0.48	0.31	0.28	0.3	0.19
C3_03060W_A	-0.09	0.83	0.73	-0.05	0.91	0.86
C1_14500C_A	0.61	0	0	0.5	0	0
C1_03030W_A	-0.04	0.66	0.51	-0.46	0	0
C5_00730W_A	0.29	0.01	0	-0.19	0.15	0.08
C6_03470W_A	0.15	0.25	0.13	-0.07	0.67	0.54
C6_04150W_A	NA	NA	NA	NA	NA	NA
C3_02870C_A	0.1	0.83	0.72	0.39	0.33	0.21
C1_04100C_A	0.5	0	0	0.55	0	0
C1_01480C_A	0.02	0.86	0.76	-0.61	0	0
C1_11520C_A	-0.1	0.38	0.23	-0.73	0	0
CR_09910W_A	-0.34	0.21	0.1	0.07	0.8	0.71
C1_08380W_A	0.26	0	0	-0.1	0.34	0.22
C4_02700W_A	-0.17	0.48	0.32	0.04	0.87	0.81
C1_08160W_A	0	1	0.99	0.09	0.59	0.45
C5_02120C_A	-0.13	0.44	0.28	-0.09	0.59	0.45
C1_08010W_A	1.24	0	0	1.46	0	0
C1_02640C_A	0.17	0.14	0.06	0.14	0.35	0.23
C1_11040W_A	-0.04	0.86	0.77	-0.35	0	0
C6_00800C_A	0.26	0.04	0.01	0.41	0	0
C1_06630W_A	0	1	0.99	-0.14	0.32	0.2
C4_00370W_A	-1.02	0	0	-1.4	0	0
C1_07630W_A	0.12	0.38	0.23	0.1	0.5	0.36
C1_05300C_A	-0.08	0.23	0.12	-0.49	0	0
C3_00800W_A	-0.22	0.05	0.02	-0.8	0	0
C3_06090C_A	0.23	0.03	0.01	0.3	0	0
C3_03810W_A	0.03	0.84	0.74	0.08	0.6	0.46
C1_02660C_A	0.09	0.51	0.34	0.03	0.84	0.77
C6_02540C_A	0.02	0.93	0.87	-0.16	0.43	0.29
CR_07920W_A	-0.07	0.67	0.52	0.14	0.28	0.17
C7_02290W_A	0.26	0.63	0.47	0.32	0.64	0.51

C6_02450W_A	0.39	0.38	0.22	0.88	0.02	0.01
CR_05570C_A	-0.56	0.03	0.01	-0.76	0	0
C3_06220C_A	0.05	0.94	0.89	0.42	0.44	0.31
C2_04850C_A	0.12	0.17	0.08	0.19	0.02	0.01
C1_05500W_A	-0.56	0.01	0	-0.35	0.14	0.07
C1_02240W_A	-0.09	0.48	0.32	-0.07	0.63	0.5
C1_14150C_A	-0.19	0.25	0.13	-0.31	0.05	0.02
C5_00880C_A	0.2	0.17	0.08	0.25	0.07	0.03
C3_06150W_A	-0.32	0.02	0.01	-0.14	0.35	0.23
C3_07300W_A	-0.97	0	0	-1.06	0	0
C6_01880W_A	0.73	0	0	0.84	0	0
CR_10300W_A	0.29	0	0	0.45	0	0
C6_04420W_A	0.93	0	0	2.15	0	0
CR_05490W_A	0.72	0	0	1	0	0
C6_00930C_A	0.5	0.06	0.02	1.59	0	0
C1_13160W_A	-0.05	0.67	0.52	0.51	0	0
C1_11140W_A	0.06	0.74	0.6	0.05	0.8	0.71
C5_05300W_A	0.19	0.03	0.01	0.34	0	0
C1_11230W_A	-0.19	0.44	0.28	-0.16	0.54	0.4
C5_01560C_A	0.63	0	0	0.88	0	0
C1_09220W_A	0.34	0	0	0.55	0	0
C7_03230C_A	-0.17	0.72	0.58	-0.83	0.11	0.05
C1_13740W_A	-0.33	0.07	0.03	0	1	1
CR_06760C_A	0.49	0	0	0.63	0	0
C2_07380W_A	0.09	0.28	0.15	-0.32	0	0
C2_03600W_A	0.02	0.95	0.91	-0.08	0.71	0.6
CR_10120C_A	-0.16	0.55	0.38	-0.08	0.78	0.68
CR_03770C_A	-0.29	0.01	0	-0.37	0	0
C4_04660C_A	0.58	0	0	1.23	0	0
C2_05380W_A	-0.43	0.01	0	-0.43	0.01	0
C1_02790W_A	0.17	0.02	0.01	-0.07	0.49	0.35
C4_05440C_A	-1.05	0	0	-1.15	0	0
C3_02700W_A	-0.73	0	0	-0.57	0.01	0
C5_04560C_A	NA	NA	NA	NA	NA	NA
C2_10530C_A	0.02	0.94	0.89	0.18	0.19	0.1
C2_07220W_A	0.17	0.02	0.01	0.31	0	0
C2_00430C_A	0.1	0.55	0.39	0.15	0.33	0.21
C2_03790C_A	0.03	0.84	0.74	0.41	0	0
C6_00370C_A	-0.61	0	0	-1.05	0	0
CR_10850C_A	0.31	0	0	0.29	0.01	0
CR_05220C_A	-0.4	0.08	0.03	-0.28	0.22	0.13
C6_04470C_A	0.14	0.2	0.1	-0.21	0.07	0.03
C6_03960W_A	0.05	0.56	0.4	-0.06	0.52	0.38

C3_05350C_A	-0.15	0.44	0.27	0.08	0.68	0.56
C7_03960C_A	-0.16	0.15	0.07	-0.1	0.4	0.27
C1_02040C_A	-0.17	0.32	0.18	-0.21	0.21	0.12
C5_03520W_A	0.07	0.85	0.75	0.2	0.73	0.62
CR_07300W_A	-0.53	0.26	0.14	-0.32	0.64	0.5
C4_05020W_A	-0.3	0.04	0.01	-0.5	0	0
C1_13080W_A	0.07	0.67	0.51	0.22	0.53	0.4
C2_03960W_A	-0.41	0	0	-0.61	0	0
C3_01020W_A	-0.31	0.08	0.03	-0.35	0.04	0.02
C2_00360C_A	0.53	0	0	0.21	0.03	0.01
C5_04150C_A	0.46	0.01	0	0.74	0	0
C1_00320W_A	0.32	0	0	0.38	0	0
C1_04790W_A	-0.03	0.9	0.83	-0.09	0.64	0.5
C1_11110C_A	-0.12	0.72	0.58	-0.07	0.83	0.75
C1_03920C_A	-0.01	0.98	0.96	-0.03	0.94	0.9
C2_04720C_A	0.3	0.02	0.01	0.28	0.05	0.02
C1_08420W_A	-0.03	0.8	0.69	-0.25	0.01	0
C1_03780C_A	0.02	0.88	0.79	-0.58	0	0
C5_00600C_A	0.29	0.58	0.41	0.69	0.25	0.15
C3_06820C_A	0.14	0.21	0.1	0.23	0.04	0.02
C2_07790C_A	0.39	0.35	0.2	0.52	0.22	0.13
C2_07120W_A	-0.03	0.86	0.77	0.08	0.57	0.43
C5_05440C_A	0.65	0	0	1.05	0	0
C4_00150C_A	0.19	0.04	0.01	0.19	0.04	0.02
C1_04400C_A	0.07	0.31	0.17	-0.26	0	0
C2_09980W_A	0.11	0.37	0.22	0.4	0	0
C1_02590C_A	-0.94	0	0	-0.87	0	0
C7_04060W_A	0.69	0.03	0.01	1.27	0	0
CR_07790C_A	0.84	0	0	0.51	0	0
C2_06360C_A	-0.84	0	0	-0.69	0.02	0.01
C5_01800C_A	0.64	0	0	0.52	0	0
CR_06950C_A	-1.3	0	0	-1.12	0	0
C1_03650C_A	-0.14	0.16	0.07	-0.32	0	0
C7_01450C_A	-0.24	0	0	-0.35	0	0
C1_07800W_A	-0.54	0.01	0	-0.42	0.04	0.02
C4_00110C_A	-1.29	0	0	-2.02	0	0
C1_10740C_A	-1.08	0	0	-0.49	0	0
C6_02190C_A	0.33	0	0	0.26	0.04	0.02
CR_08550W_A	0.18	0.08	0.03	0.21	0.06	0.03
C3_05100C_A	0.02	0.86	0.77	-0.45	0	0
C6_03640W_A	-0.9	0	0	-0.56	0	0
C1_02970W_A	0.12	0.19	0.09	-0.06	0.63	0.5
C4_04110W_A	-0.07	0.67	0.52	0	0.99	0.99

C4_05700W_A	-0.02	0.97	0.95	-0.26	0.55	0.42
C1_10510W_A	0.11	0.72	0.58	0.08	0.8	0.71
C5_02410C_A	0.11	0.68	0.54	0.31	0.15	0.08
C2_05890C_A	0.36	0	0	0.23	0.01	0
C5_00810C_A	0.15	0.76	0.63	-0.03	0.97	0.95
C4_05670W_A	-0.06	0.62	0.46	0.07	0.65	0.52
C1_02330C_A	-0.15	0.11	0.05	-0.66	0	0
C1_11190W_A	-0.06	0.76	0.63	-0.1	0.55	0.41
C1_03530W_A	-0.07	0.67	0.52	-0.17	0.2	0.12
C4_05900C_A	0.26	0.01	0	0.15	0.21	0.12
C7_04260W_A	0.07	0.44	0.28	0.24	0.01	0
C4_01370W_A	0.26	0	0	-0.08	0.52	0.38
C7_03020C_A	0.03	0.89	0.81	-0.2	0.16	0.09
C6_04060W_A	-0.05	0.69	0.54	0.04	0.81	0.73
C1_12720C_A	-0.05	0.94	0.88	-0.1	0.89	0.83
C4_03700W_A	-0.73	0	0	-0.88	0	0
C4_03270W_A	-0.96	0	0	-0.93	0	0
C2_08260W_A	0.01	0.99	0.98	0.15	0.82	0.74
C2_05810W_A	0.28	0.13	0.06	0.56	0	0
C4_05610C_A	-0.16	0.05	0.02	-0.1	0.34	0.22
CR_04610C_A	-0.13	0.82	0.72	-0.01	0.99	0.98
CR_01170W_A	0.12	0.26	0.14	-0.09	0.42	0.29
C5_05390C_A	-0.22	0.01	0	-2.6	0	0
C1_02020W_A	0.85	0	0	0.85	0	0
C1_02290C_A	-0.14	0.34	0.2	-0.14	0.3	0.19
CR_06330C_A	-0.63	0	0	-0.43	0.08	0.04
C3_00150W_A	NA	NA	NA	NA	NA	NA
CR_04280C_A	-0.11	0.57	0.41	-0.34	0.04	0.02
C4_01500W_A	-0.45	0.18	0.08	0.28	0.41	0.28
C2_08210C_A	NA	NA	NA	NA	NA	NA
C4_01150W_A	-0.31	0.05	0.02	-0.22	0.28	0.17
C4_02790C_A	-1.07	0	0	-0.93	0	0
C6_01090C_A	0.22	0.67	0.52	-0.02	0.98	0.96
C6_03760C_A	-0.65	0	0	-1.17	0	0
C4_01960C_A	0.44	0	0	0.92	0	0
C2_02250C_A	-0.24	0.12	0.05	-0.14	0.39	0.26
C4_04350W_A	0	0.99	0.99	-0.34	0.01	0
C1_11300C_A	0.07	0.69	0.55	0.09	0.63	0.5
CR_01680C_A	0.14	0.23	0.11	0.02	0.91	0.86
C4_01850C_A	0.36	0.34	0.2	0.67	0.06	0.03
C1_05630C_A	-0.18	0.06	0.02	-0.93	0	0
CR_04240C_A	-0.48	0	0	-0.75	0	0
CR_00160C_A	-0.04	0.84	0.75	-0.14	0.38	0.26

C3_06070C_A	-0.23	0.03	0.01	0.17	0.11	0.06
C1_13660W_A	-0.36	0.04	0.01	-0.7	0	0
C3_04790W_A	0.08	0.69	0.54	0.13	0.46	0.32
C1_08360C_A	0.31	0	0	0.23	0.01	0
C3_06640W_A	0.21	0.32	0.18	0.65	0	0
C1_12150C_A	-0.05	0.91	0.83	0.19	0.64	0.51
CR_04750W_A	-0.97	0	0	-1.38	0	0
C6_03170C_A	0.44	0.09	0.04	0.63	0.01	0
C4_05180C_A	-0.31	0.01	0	-0.34	0.01	0
C3_04870W_A	0.44	0.33	0.19	0.07	0.92	0.88
C2_09850C_A	-0.31	0.53	0.37	-0.31	0.6	0.46
CR_10500C_A	0.28	0.1	0.04	-0.23	0.24	0.14
C3_05620W_A	0.35	0.5	0.33	0.61	0.3	0.19
C3_04430W_A	0.23	0.01	0	0.19	0.04	0.02
C2_08520C_A	NA	NA	NA	NA	NA	NA
C6_03850C_A	-0.04	0.8	0.68	-1.99	0	0
CR_03250C_A	0.3	0.06	0.02	0.45	0	0
C6_00010W_A	NA	NA	NA	0.25	0.5	0.36
C2_05740W_A	-0.45	0.01	0	-0.2	0.29	0.18
CR_08510W_A	0.53	0	0	1.78	0	0
C6_04180W_A	-0.63	0	0	-0.46	0.03	0.01
C2_07390C_A	-0.13	0.31	0.17	-0.21	0.07	0.03
C3_07440W_A	0.01	0.94	0.89	-0.14	0.16	0.08
C3_01260C_A	0.33	0.07	0.03	0.1	0.67	0.55
C4_03040W_A	-0.14	0.77	0.64	-0.14	0.75	0.64
C4_00980C_A	0.07	0.91	0.84	1.03	0.05	0.02
C1_12260W_A	-0.25	0.39	0.24	-0.38	0.17	0.09
C1_11790W_A	0.03	0.9	0.83	0.03	0.9	0.85
C2_01510C_A	-0.06	0.82	0.71	0.2	0.38	0.26
C1_01130W_A	-0.34	0	0	-0.51	0	0
C4_07180W_A	-0.05	0.44	0.28	-0.47	0	0
C7_01490W_A	0.11	0.42	0.26	0	0.99	0.98
C1_14010W_A	-0.01	0.98	0.95	-0.12	0.79	0.7
CR_01250C_A	NA	NA	NA	0.12	0.66	0.53
C1_09760C_A	0.04	0.79	0.67	0	0.98	0.96
C5_01220W_A	-0.6	0	0	-0.9	0	0
CR_09600C_A	-0.32	0.01	0	0.18	0.1	0.05
C7_00350C_A	-0.1	0.45	0.29	0.56	0	0
C2_00900W_A	0.07	0.67	0.52	NA	NA	NA
C2_04020C_A	0.13	0.24	0.12	0.17	0.13	0.06
C3_03330C_A	-0.22	0.16	0.07	-0.13	0.43	0.3
C1_07330W_A	-0.82	0.04	0.01	-1.36	0	0
C2_00350W_A	-0.39	0	0	-0.45	0	0

C3_03930W_A	-0.59	0	0	-0.86	0	0
C4_04100C_A	-1.15	0	0	-1.44	0	0
C7_03290C_A	-0.06	0.85	0.76	-0.37	0.13	0.06
C2_03700W_A	0.36	0.1	0.04	0.57	0	0
C5_02140C_A	0.14	0.16	0.08	0.08	0.52	0.38
C7_02870C_A	-0.14	0.39	0.23	-0.25	0.11	0.05
C1_06880C_A	-0.28	0.6	0.43	-0.17	0.8	0.7
C4_05240C_A	0.1	0.3	0.17	-0.16	0.14	0.07
C4_01410W_A	-0.01	0.95	0.91	0.01	0.95	0.92
CR_08210C_A	0.48	0	0	0.71	0	0
C1_09080C_A	0.64	0	0	1.07	0	0
CR_01830C_A	-0.32	0.53	0.37	-0.63	0.26	0.16
C3_02290W_A	-0.03	0.83	0.73	-0.17	0.2	0.11
C2_02030W_A	0.05	0.64	0.48	-0.3	0	0
C7_01650W_A	0.36	0	0	0.2	0.01	0
C2_03410W_A	0.16	0.16	0.07	0.18	0.12	0.06
C5_02330W_A	-0.01	0.95	0.91	-0.1	0.62	0.49
C7_00880C_A	-0.17	0.32	0.19	-0.19	0.28	0.17
C3_02810C_A	0.49	0	0	0.78	0	0
CR_03580C_A	-0.29	0.54	0.37	0.41	0.34	0.22
C1_08460C_A	-0.29	0	0	-0.61	0	0
C3_06910C_A	0.08	0.32	0.18	-0.09	0.4	0.27
C6_00840W_A	0.64	0	0	0.13	0.35	0.23
C1_08260C_A	0.01	0.95	0.91	0.04	0.79	0.69
C5_04520W_A	0.11	0.53	0.37	0.02	0.94	0.9
C5_05150C_A	0	1	1	0.19	0.33	0.21
C2_02920W_A	0.71	0	0	1.2	0	0
CR_06620W_A	0.05	0.77	0.64	-0.11	0.5	0.36
C2_01460C_A	-0.27	0.43	0.27	-0.02	0.98	0.97
C6_02560W_A	-0.1	0.25	0.13	-0.36	0	0
C3_03660W_A	-0.02	0.92	0.85	0.91	0	0
C4_04050C_A	-0.98	0	0	-1.83	0	0
CR_04730W_A	0.53	0	0	0.47	0	0
C1_05720W_A	-0.01	0.92	0.85	-0.59	0	0
CR_04100C_A	-0.05	0.64	0.48	-0.53	0	0
C7_01520W_A	-1.3	0	0	-1.77	0	0
C7_03280C_A	-0.13	0.62	0.46	0.16	0.51	0.38
C1_05000W_A	0.59	0	0	0.76	0	0
C4_03410W_A	-0.32	0	0	-0.53	0	0
C6_02040W_A	0.1	0.28	0.15	0.52	0	0
C6_01710C_A	-0.02	0.91	0.84	0.08	0.55	0.41
C7_01920W_A	0.27	0	0	0.15	0.2	0.11
C1_02160W_A	0.1	0.76	0.63	0.29	0.28	0.17

C2_06830C_A	-0.31	0.03	0.01	-0.31	0.03	0.01
C1_05170C_A	0.2	0.04	0.01	0.6	0	0
C5_01510W_A	-0.16	0.54	0.38	-0.17	0.51	0.37
C2_06390C_A	-0.42	0	0	-1.11	0	0
C2_02440W_A	-0.14	0.39	0.23	-0.07	0.68	0.56
C3_01150C_A	-0.59	0	0	-0.4	0.02	0.01
C1_01300W_A	0.01	0.94	0.9	0.02	0.89	0.83
C4_02990C_A	-0.97	0	0	-1.1	0	0
C1_02900C_A	-0.34	0.06	0.02	-0.18	0.39	0.26
C2_02140C_A	-0.07	0.82	0.71	-0.28	0.47	0.33
C6_02010C_A	0.38	0	0	0.06	0.53	0.39
C1_00620W_A	0.45	0	0	0.58	0	0
C5_01380W_A	0	1	0.99	-0.01	0.99	0.98
C2_07100W_A	0	1	0.99	-0.17	0.07	0.03
C4_00030C_A	0.17	0.27	0.14	0.15	0.34	0.22
C6_00210W_A	0.33	0	0	0.4	0	0
CR_03950W_A	-0.13	0.55	0.38	0.08	0.66	0.53
C5_04720C_A	-0.63	0	0	-0.89	0	0
CR_08160W_A	0.17	0.28	0.15	-0.09	0.63	0.5
C5_04310W_A	-0.56	0	0	-0.59	0	0
C7_02910W_A	-0.65	0	0	-0.56	0	0
C1_02470W_A	-0.78	0.04	0.02	0.18	0.69	0.57
C4_02830C_A	-1.06	0	0	-0.9	0	0
CR_09990W_A	-0.03	0.84	0.75	0	0.99	0.98
C2_05190W_A	-0.41	0	0	-0.58	0	0
C1_08540C_A	0.3	0	0	0.35	0	0
C5_01200W_A	0.34	0.11	0.05	1.13	0	0
C4_03190W_A	-0.51	0	0	-0.32	0.03	0.01
C5_02930C_A	0.06	0.55	0.39	0.1	0.39	0.26
C2_10150W_A	0.55	0.12	0.05	1.42	0	0
C2_04780W_A	-0.41	0.14	0.06	-0.11	0.8	0.71
C1_12940C_A	0.29	0.31	0.17	0.79	0.12	0.06
C4_07100C_A	-0.14	0.31	0.17	-0.37	0	0
C2_03130W_A	-0.06	0.74	0.6	0	0.99	0.98
C2_05130W_A	1.33	0	0	1.84	0	0
C2_04770W_A	-0.58	0.03	0.01	-0.19	0.62	0.49
C2_02550C_A	0.45	0.31	0.17	0.87	0.04	0.02
C6_04190C_A	-0.44	0.24	0.12	-0.73	0.07	0.03
CR_03130W_A	-0.04	0.9	0.83	0.03	0.91	0.86
C2_05580W_A	0.21	0.14	0.06	0.77	0	0
C4_01910W_A	-0.18	0.47	0.3	-0.17	0.48	0.34
C2_03440W_A	0.45	0	0	0.43	0	0
C7_00430W_A	0.26	0.56	0.4	0.07	0.89	0.84

CR_01710W_A	-0.56	0.15	0.07	-0.26	0.58	0.45
CR_07270C_A	0.13	0.75	0.62	0.09	0.83	0.76
CR_08990C_A	0.88	0	0	0.65	0	0
C7_01890C_A	0.19	0.47	0.3	0	1	1
C1_05610W_A	0.05	0.78	0.66	-0.09	0.57	0.44
C3_07880C_A	0.23	0.11	0.05	0.24	0.12	0.06
C3_04210W_A	0.43	0.37	0.22	0.56	0.31	0.19
C4_01870C_A	0.34	0	0	0.32	0	0
C5_01750C_A	0.25	0.58	0.41	1.02	0.08	0.04
C1_09020W_A	-0.07	0.72	0.58	0.05	0.8	0.71
C2_07700C_A	-0.07	0.91	0.84	-0.15	0.83	0.76
CR_04230W_A	-0.17	0.23	0.11	-0.16	0.25	0.15
C7_03140W_A	0.25	0.58	0.41	0.31	0.51	0.37
C1_05690C_A	-0.29	0.5	0.33	-0.13	0.86	0.8
C1_10990C_A	-0.25	0.04	0.01	-0.13	0.37	0.24
C2_05480C_A	0.07	0.88	0.8	0.01	0.99	0.98
C1_09570W_A	0.26	0.02	0.01	0.24	0.05	0.02
C4_02660W_A	0.58	0.11	0.05	0.81	0.02	0.01
C4_03590C_A	0.37	0	0	0.61	0	0
C6_03120W_A	-0.29	0.05	0.02	-0.13	0.47	0.34
CR_07950W_A	-0.57	0	0	-0.4	0.03	0.01
C1_07020C_A	0	0.98	0.97	0.26	0.06	0.03
C4_05460C_A	-0.25	0	0	-0.47	0	0
CR_10750C_A	-0.3	0.03	0.01	-1.2	0	0
CR_02280W_A	2.55	0	0	4.11	0	0
C4_06300C_A	0.01	0.99	0.98	0.29	0.65	0.51
CR_09240C_A	-0.12	0.31	0.18	-0.04	0.78	0.69
C3_05020W_A	0.15	0.53	0.36	-0.42	0.06	0.03
C6_03160C_A	0.4	0	0	0.54	0	0
CR_02290W_A	0.12	0.82	0.71	0.39	0.57	0.43
C4_00540C_A	-0.36	0.01	0	-0.4	0.01	0
C7_00090C_A	0.2	0.21	0.1	-0.16	0.37	0.24
C3_03500W_A	0.03	0.91	0.84	-0.15	0.46	0.32
C2_10330C_A	0.38	0.21	0.1	0.34	0.28	0.17
CR_10570C_A	0.22	0.18	0.09	0.08	0.67	0.55
C1_10100C_A	0.15	0.09	0.03	0	0.99	0.98
C1_02740C_A	-0.22	0.48	0.31	-0.32	0.27	0.17
C1_13200C_A	-0.07	0.84	0.74	-0.03	0.93	0.89
C3_04340W_A	0.26	0.05	0.02	0.22	0.11	0.05
CR_09350C_A	0.75	0.07	0.02	0.81	0.1	0.05
CR_09960C_A	-0.1	0.48	0.32	0.03	0.9	0.84
C7_01270C_A	-0.51	0	0	-0.15	0.41	0.28
C3_02540C_A	-0.28	0.14	0.06	-0.17	0.38	0.25

C2_09240C_A	0.03	0.97	0.94	-0.05	0.93	0.89
C5_05210W_A	0.16	0.17	0.08	-0.06	0.68	0.56
C1_08500C_A	-0.04	0.73	0.59	-0.19	0.05	0.02
CR_02060W_A	0.39	0.04	0.01	0.47	0.01	0
C5_03300C_A	-0.33	0.14	0.06	-0.09	0.75	0.65
C7_01680C_A	-0.48	0.09	0.03	-0.21	0.51	0.37
C6_00240C_A	-0.23	0.11	0.04	-0.3	0.04	0.02
C1_06230C_A	-0.45	0.09	0.03	-0.5	0.06	0.03
C2_08590W_A	-0.02	0.85	0.76	-2.33	0	0
C6_01100W_A	0.01	0.98	0.95	0.5	0.1	0.05
C1_00090W_A	0.09	0.81	0.7	-0.15	0.66	0.53
C4_02050W_A	-0.17	0.14	0.06	-0.48	0	0
C3_06020W_A	0.35	0	0	0.35	0	0
C6_00120W_A	-0.23	0.61	0.45	-0.01	0.98	0.97
CR_08600C_A	0.1	0.38	0.23	0.12	0.33	0.21
C3_00450C_A	-0.07	0.36	0.21	-0.15	0.1	0.05
C1_13490C_A	0.38	0.02	0.01	0.45	0.01	0
C7_04340C_A	0.67	0.08	0.03	0.67	0.12	0.06
C2_07870C_A	NA	NA	NA	NA	NA	NA
C3_01760W_A	0.12	0.18	0.09	0.09	0.39	0.27
C2_06730W_A	0.21	0.02	0	0.03	0.82	0.73
C1_10060C_A	0.36	0.23	0.12	0.6	0.21	0.12
C3_01680C_A	-0.41	0	0	-0.45	0	0
C4_04570W_A	-0.14	0.29	0.16	-0.48	0	0
C1_04800C_A	0.14	0.81	0.7	0.64	0.29	0.18
C3_04180W_A	0.62	0	0	0.58	0	0
C3_04310C_A	0.75	0.09	0.04	2.66	0	0
C1_13920W_A	0.23	0.03	0.01	0.39	0	0
C1_08190C_A	0.82	0.02	0.01	1.12	0	0
C1_07080W_A	0.1	0.79	0.67	-0.05	0.91	0.85
C1_02100W_A	-0.18	0.48	0.31	-0.03	0.92	0.88
CR_04370W_A	-0.01	0.97	0.95	0.32	0.08	0.04
CR_00780C_A	0.53	0	0	0.34	0	0
CR_03410W_A	0.29	0.05	0.02	0.14	0.41	0.28
C3_02920W_A	0.12	0.43	0.27	0.09	0.55	0.41
C4_02950C_A	-0.05	0.92	0.85	-0.12	0.77	0.67
C1_14280C_A	0.06	0.56	0.4	-0.27	0	0
C5_02500C_A	0.17	0.41	0.26	0.21	0.26	0.16
C1_10830W_A	0.01	0.96	0.92	-0.2	0.13	0.07
C1_09140C_A	-0.05	0.87	0.79	-0.09	0.77	0.67
C3_00200C_A	0.07	0.43	0.27	0.19	0.05	0.02
C2_10190C_A	0.16	0.44	0.28	0.24	0.19	0.1
C6_01420C_A	0.2	0.03	0.01	0.24	0.01	0

C2_07650C_A	0.19	0.03	0.01	0.23	0.01	0.01
C2_06140C_A	-0.06	0.92	0.86	0.47	0.41	0.28
C3_04910C_A	-0.45	0	0	-1.3	0	0
C6_03280W_A	0.02	0.97	0.95	0.36	0.55	0.41
CR_08740W_A	0.12	0.69	0.54	0.11	0.81	0.73
CR_00260W_A	0.1	0.32	0.18	0.07	0.55	0.41
C1_09480W_A	-0.14	0.65	0.49	0	1	1
C2_04360W_A	-0.38	0	0	-0.33	0.01	0
C1_09620C_A	0.33	0.01	0	0.2	0.23	0.13
C3_01730C_A	0.3	0	0	0.45	0	0
C4_01660W_A	-0.11	0.4	0.24	-0.18	0.15	0.08
C7_03820C_A	-0.18	0.62	0.45	0.12	0.73	0.61
C6_03930W_A	-0.86	0	0	-1.79	0	0
CR_08030C_A	0.1	0.29	0.16	0.07	0.52	0.38
C7_03070C_A	-0.01	0.93	0.88	0.06	0.62	0.48
C4_07140W_A	-0.57	0	0	-0.35	0.04	0.02
C1_00180W_A	-0.12	0.12	0.05	-0.54	0	0
C3_06770W_A	-0.27	0.46	0.3	-0.21	0.59	0.45
CR_08620C_A	0.06	0.75	0.62	0.01	0.96	0.92
C1_06460C_A	-0.07	0.4	0.25	-0.49	0	0
C4_05940W_A	0	1	0.99	-0.36	0.16	0.08
CR_01010W_A	-0.37	0.45	0.29	-0.31	0.6	0.47
C1_11370C_A	-0.09	0.61	0.45	-0.01	0.96	0.93
C1_00500C_A	0.15	0.06	0.02	0.21	0.01	0
C5_02680W_A	0.37	0.12	0.05	-0.07	0.82	0.75
C6_01080C_A	-0.51	0.06	0.02	0.15	0.63	0.49
C6_02630C_A	-0.13	0.32	0.18	-0.33	0.01	0
C2_04130W_A	0.03	0.85	0.76	0.07	0.66	0.54
C1_03620C_A	-0.39	0	0	-0.48	0	0
C4_05730W_A	0.27	0.14	0.06	0.39	0.02	0.01
C1_10570C_A	-0.17	0.75	0.62	-0.25	0.66	0.53
CR_05310W_A	0.59	0	0	0.87	0	0
C2_02430W_A	-0.89	0	0	-1.06	0	0
C5_04570C_A	-0.21	0.02	0.01	-1.1	0	0
C2_01970C_A	-0.29	0.01	0	-0.16	0.25	0.15
C2_07690W_A	-0.21	0.49	0.33	-0.08	0.81	0.72
C4_01840C_A	0.4	0	0	0.4	0	0
C7_03090C_A	-0.07	0.78	0.66	0.32	0.12	0.06
C5_03360W_A	-0.16	0.34	0.19	-0.47	0	0
C4_03390W_A	0.19	0.06	0.02	0.1	0.42	0.29
C6_00730W_A	0.01	0.99	0.98	-0.77	0.19	0.1
C5_03110C_A	-0.49	0.09	0.04	-0.26	0.44	0.3
C4_00680W_A	0.2	0.25	0.13	0.16	0.4	0.27

C2_06620W_A	-0.2	0.56	0.39	-0.05	0.9	0.85
C2_04420W_A	0.36	0.29	0.15	-0.01	0.99	0.98
C3_04330C_A	0.41	0.07	0.03	1.34	0	0
C6_04370W_A	0.04	0.88	0.8	0.04	0.87	0.8
C4_00310C_A	-0.03	0.85	0.75	-0.45	0	0
C2_03980C_A	-0.02	0.92	0.86	0.1	0.54	0.4
C3_05240C_A	0.07	0.34	0.2	-0.43	0	0
C4_06350C_A	-0.17	0.23	0.12	-0.03	0.86	0.8
C1_00070W_A	0.3	0	0	0.13	0.27	0.17
C4_06580W_A	-0.16	0.2	0.1	-0.7	0	0
C7_00500W_A	NA	NA	NA	NA	NA	NA
C3_00460W_A	0.49	0	0	0.84	0	0
C2_05180W_A	-0.93	0.01	0	0.02	0.96	0.93
CR_05610C_A	0.84	0	0	0.8	0	0
C3_07620C_A	-0.41	0.05	0.02	-0.17	0.47	0.33
C4_03810W_A	0.01	0.98	0.96	0.11	0.65	0.52
CR_07600W_A	-0.23	0.09	0.04	-0.03	0.89	0.83
CR_09550C_A	-0.21	0.09	0.04	-0.06	0.7	0.59
CR_07050C_A	-0.54	0	0	-1.2	0	0
C2_04170C_A	0.22	0.25	0.13	0.74	0	0
C7_02390W_A	0.15	0.2	0.1	0.32	0	0
C3_02140C_A	0.01	0.95	0.91	0.17	0.2	0.11
C7_00120W_A	0.56	0	0	1.16	0	0
C5_04960W_A	-0.3	0.03	0.01	-0.09	0.67	0.55
C1_07280C_A	0.02	0.98	0.96	-0.12	0.83	0.76
C7_03670W_A	0.15	0.02	0.01	-0.31	0	0
CR_02090C_A	-0.04	0.75	0.62	-0.28	0	0
C2_09580W_A	0.24	0.01	0	0.34	0	0
C1_05960W_A	0.04	0.69	0.54	-0.81	0	0
C2_03900C_A	0.25	0.01	0	0.25	0.01	0.01
C2_05610C_A	-0.04	0.7	0.55	-0.45	0	0
C3_06080W_A	0.32	0.02	0	0.49	0	0
C2_02120W_A	-0.2	0.1	0.04	-0.13	0.3	0.19
C3_05410W_A	-0.01	0.97	0.94	-0.26	0.09	0.05
C4_06290W_A	-0.37	0.3	0.17	-0.43	0.24	0.14
C1_12900W_A	-0.1	0.65	0.49	-0.28	0.13	0.07
C2_06600W_A	-0.53	0	0	-0.7	0	0
C3_01580W_A	-0.41	0.26	0.14	-0.27	0.53	0.39
C1_01850C_A	-0.2	0.3	0.16	0.11	0.61	0.47
C5_03640W_A	-0.09	0.54	0.37	-0.3	0.03	0.01
C5_03940C_A	-0.02	0.91	0.84	0.32	0.02	0.01
C1_14570C_A	0.21	0.5	0.34	-0.13	0.7	0.58
C1_12350W_A	-0.46	0	0	-0.49	0	0

C7_00330C_A	-0.18	0.44	0.28	0.37	0.04	0.02
C4_05230C_A	-0.79	0	0	-0.58	0.01	0
C2_07440C_A	-0.77	0	0	-1.57	0	0
C1_10880W_A	-0.37	0.06	0.02	0.02	0.93	0.89
BGI_novel_G000(	0.67	0	0	1.1	0	0
C3_01420C_A	0.25	0.18	0.09	0.28	0.13	0.07
C2_00510W_A	0.21	0.19	0.09	0.35	0.02	0.01
C6_01450C_A	0.76	0.01	0	1.96	0	0
C5_01610W_A	-0.85	0	0	-0.62	0.02	0.01
C1_13670W_A	0.02	0.89	0.82	-0.44	0	0
C2_09650W_A	-0.18	0.04	0.01	0.13	0.18	0.1
CR_10400W_A	0.03	0.83	0.73	0.12	0.34	0.22
C1_07980C_A	0.34	0.5	0.33	0.57	0.37	0.24
C1_04730C_A	0.32	0	0	0.18	0.13	0.06
C4_01080W_A	-0.32	0.01	0	-0.3	0.02	0.01
C2_04830W_A	-0.31	0.11	0.05	0	0.99	0.98
C1_04550W_A	0.19	0.5	0.34	0.33	0.18	0.1
C1_08320W_A	0.18	0.03	0.01	0.07	0.57	0.43
C1_03170C_A	0.02	0.97	0.93	0.06	0.84	0.77
C3_02720W_A	-0.03	0.92	0.86	0.09	0.74	0.63
C2_00790C_A	-0.29	0.07	0.03	-0.39	0.01	0.01
C2_02170W_A	-0.24	0.17	0.08	-0.38	0.04	0.02
C1_04520C_A	0.45	0	0	0.52	0	0
C1_01100W_A	0.77	0	0	0.93	0	0
C4_06170C_A	-0.04	0.8	0.69	0.2	0.07	0.03
C1_12000C_A	0.35	0.42	0.26	0.22	0.67	0.55
C3_02440C_A	-0.12	0.64	0.48	0.23	0.31	0.2
C5_04080C_A	-0.15	0.59	0.42	0.4	0.07	0.03
C1_10660W_A	-0.37	0	0	-0.06	0.67	0.55
C2_02380W_A	0.3	0	0	0.35	0	0
C1_12850W_A	0.52	0.21	0.11	0.39	0.44	0.31
C1_07120W_A	-0.36	0	0	-0.95	0	0
C5_01060C_A	-0.42	0.05	0.02	-0.28	0.22	0.13
C1_13480W_A	-0.43	0	0	-0.21	0	0
C4_00520W_A	-0.34	0.01	0	-0.25	0.08	0.04
C7_00960W_A	-0.31	0	0	-0.57	0	0
C1_14400C_A	0.17	0.22	0.11	0.27	0.06	0.03
C2_07560W_A	-0.08	0.53	0.36	0.05	0.69	0.58
C3_02800W_A	-0.07	0.68	0.53	-0.13	0.65	0.52
C5_04250W_A	0.8	0.06	0.02	1.52	0	0
C1_07260C_A	0.4	0	0	0.49	0	0
C2_02160W_A	-0.37	0.03	0.01	-0.46	0.01	0
C1_03410W_A	-0.86	0	0	-1.01	0	0

C2_05730C_A	0.05	0.72	0.58	-0.23	0.05	0.02
CR_06850C_A	0.34	0.29	0.16	0.43	0.17	0.09
C4_01920W_A	0.75	0	0	1.13	0	0
CR_07070C_A	0.07	0.72	0.58	0.19	0.23	0.13
C6_00140C_A	0.68	0	0	0.87	0	0
C1_12590W_A	0.1	0.56	0.39	0.51	0	0
C3_00360W_A	0.52	0	0	0.87	0	0
C1_01620C_A	0.25	0.6	0.44	0.56	0.23	0.13
C6_00940C_A	-0.37	0.06	0.02	-0.67	0	0
CR_01800C_A	-0.08	0.83	0.73	-0.17	0.6	0.46
CR_00330C_A	0.19	0.4	0.24	0.4	0.03	0.01
CR_10530W_A	0.34	0.01	0	0.38	0	0
C6_00770C_A	-0.21	0.15	0.07	-0.18	0.24	0.14
C6_00130C_A	0.22	0	0	0.26	0	0
C6_01960W_A	0.32	0.01	0	0.71	0	0
C4_05130C_A	0.35	0.35	0.21	0.54	0.12	0.06
C1_03390W_A	-0.09	0.29	0.16	-0.35	0	0
C4_03940C_A	-0.51	0	0	-0.47	0	0
C6_01340C_A	0.01	0.98	0.95	-0.19	0.56	0.43
C1_13600W_A	0.09	0.87	0.79	0.5	0.23	0.14
C2_03420C_A	0.13	0.45	0.29	0.21	0.17	0.09
C1_03770W_A	-0.99	0	0	-0.89	0	0
CR_09110C_A	-0.16	0.43	0.27	-0.12	0.57	0.43
C4_02680C_A	-0.31	0.47	0.31	0.04	0.93	0.89
C1_04000C_A	0.56	0	0	1.21	0	0
C2_06000W_A	-0.06	0.76	0.63	-0.06	0.77	0.66
C2_00840W_A	-0.22	0.17	0.08	0.07	0.7	0.59
C3_00700W_A	-0.07	0.77	0.65	0.18	0.37	0.24
CR_00410W_A	0.02	0.93	0.88	0.03	0.87	0.81
C6_02150C_A	-0.09	0.8	0.69	0.23	0.48	0.34
C7_00080C_A	0.71	0	0	1.35	0	0
C1_11760C_A	-0.09	0.54	0.37	-0.06	0.68	0.55
C3_06270C_A	-0.1	0.62	0.46	-0.45	0.01	0
C1_12700W_A	0.64	0	0	0.68	0	0
C2_02900W_A	0.3	0.43	0.27	0.84	0.01	0
C7_01040C_A	0.26	0.07	0.03	0.44	0	0
C3_00260C_A	-0.18	0.01	0	-0.42	0	0
C2_10210C_A	-0.51	0	0	-0.38	0	0
CR_01350C_A	0.28	0	0	0.36	0	0
C4_04730W_A	-0.51	0	0	-0.48	0	0
CR_00870C_A	0.62	0.01	0	0.61	0.01	0
C3_03460C_A	-0.62	0.18	0.08	-0.99	0.08	0.04
C3_03820C_A	-0.14	0.25	0.13	-0.07	0.59	0.45

C3_02400C_A	-0.11	0.48	0.32	0.02	0.9	0.85
C1_10410W_A	-0.53	0.01	0	-0.26	0.23	0.13
C6_02320C_A	-0.21	0.37	0.22	-0.28	0.21	0.12
C6_00700C_A	0.07	0.78	0.66	0.04	0.87	0.8
C3_06240C_A	-0.19	0.39	0.24	-0.13	0.58	0.44
C5_04740C_A	-0.16	0.25	0.13	-0.13	0.39	0.26
C3_01540W_A	-0.29	0	0	-0.07	0.44	0.3
C4_05980C_A	0.27	0.2	0.1	0.29	0.18	0.1
C3_03130C_A	0.35	0.12	0.05	0.41	0.06	0.03
C1_09720W_A	0.07	0.32	0.18	-0.13	0.17	0.09
C1_00700W_A	0.12	0.59	0.43	0.07	0.77	0.67
CR_00980C_A	0.08	0.5	0.33	0.33	0	0
C3_03160C_A	-0.07	0.83	0.73	-0.19	0.51	0.37
CR_05770W_A	-0.07	0.75	0.62	-0.26	0.19	0.11
C7_01440W_A	0.2	0.68	0.53	0.49	0.26	0.16
C6_01630W_A	-0.43	0	0	-0.52	0	0
C3_05720C_A	-0.07	0.7	0.55	0.15	0.29	0.18
C1_11130W_A	-0.14	0.63	0.47	-0.24	0.33	0.21
C6_01770W_A	-0.15	0.41	0.25	-0.2	0.27	0.16
C2_09640W_A	1.11	0	0	1.4	0	0
C1_08650C_A	0.43	0	0	0.71	0	0
C5_03730W_A	0.55	0.1	0.04	0.96	0.07	0.03
CR_07350W_A	0.54	0	0	0.55	0	0
C5_03010W_A	-0.45	0.08	0.03	-0.45	0.12	0.06
C3_06490W_A	0.64	0	0	0.71	0	0
C1_12310C_A	-0.73	0	0	-1.08	0	0
C1_13010W_A	0.01	0.93	0.87	-0.07	0.61	0.48
C3_03040W_A	-0.02	0.88	0.79	-0.37	0	0
C3_01810C_A	0.17	0.04	0.01	-1.06	0	0
C4_02210W_A	-0.48	0.32	0.18	-0.39	0.53	0.4
CR_09360W_A	1.12	0	0	1.31	0	0
CR_01050C_A	0.11	0.5	0.33	0.65	0	0
C7_03840W_A	-0.73	0	0	-0.88	0	0
C3_03540W_A	0.24	0.07	0.02	0.26	0.04	0.02
C2_09910C_A	0.57	0.04	0.01	0.63	0.03	0.01
C2_04290W_A	0.16	0.12	0.05	-0.2	0.09	0.04
C5_05060C_A	-0.1	0.82	0.72	0.14	0.75	0.64
C1_07660W_A	-0.63	0.16	0.07	-0.26	0.7	0.59
C2_03270W_A	-0.09	0.18	0.09	-0.29	0	0
CR_02710W_A	-0.48	0.01	0	-0.03	0.89	0.83
C6_03000C_A	-0.23	0.48	0.32	0.09	0.8	0.71
C1_00920W_A	0.19	0.24	0.12	0.42	0	0
C4_00390W_A	-0.03	0.87	0.78	0.2	0.09	0.04

C5_01820W_A	0.08	0.43	0.27	0.04	0.69	0.57
C2_05860C_A	-0.14	0.64	0.48	0.06	0.82	0.74
C5_05280C_A	0.09	0.67	0.52	0.05	0.81	0.72
C1_04140W_A	-0.39	0.17	0.08	-0.69	0.01	0
C4_01360W_A	-0.59	0	0	-1.23	0	0
C5_02800C_A	0.31	0.55	0.39	-0.19	0.79	0.69
CR_07490C_A	-0.18	0.03	0.01	-0.22	0.02	0.01
C5_05450C_A	0.53	0	0	0.57	0	0
C2_00690W_A	0.61	0.02	0.01	0.53	0.07	0.03
C2_08960C_A	0.68	0	0	1.3	0	0
C3_03730C_A	0.25	0	0	0.26	0.02	0.01
C5_01450W_A	0.35	0.02	0.01	-0.49	0	0
C4_04850C_A	-0.03	0.93	0.88	-0.13	0.66	0.53
C4_00760W_A	0.22	0.05	0.02	0.64	0	0
CR_01670W_A	-0.37	0.03	0.01	-0.35	0.05	0.02
C1_11470C_A	0.97	0	0	1.16	0	0
C4_02060C_A	-0.26	0.01	0	-0.2	0.07	0.03
C1_01380C_A	-0.26	0.15	0.07	-0.11	0.57	0.43
CR_04090C_A	-0.35	0	0	-1.05	0	0
C1_11880W_A	-0.33	0.01	0	-0.54	0	0
CR_03400W_A	0	0.99	0.97	-0.26	0.01	0
C2_09370C_A	-0.29	0.12	0.05	-0.14	0.53	0.39
C2_04460W_A	0.3	0.01	0	0.58	0	0
C4_00740W_A	-0.46	0.06	0.02	-0.08	0.77	0.67
CR_10130W_A	-0.45	0.01	0	-0.22	0.26	0.16
C1_08100W_A	0	1	1	-0.14	0.2	0.11
CR_03370C_A	-0.72	0	0	-0.55	0.03	0.01
C4_02450W_A	0.15	0.35	0.21	-0.07	0.69	0.57
C3_01030W_A	-0.12	0.28	0.15	-0.31	0.01	0
CR_07760W_A	-0.45	0	0	-0.57	0	0
C2_09940W_A	-0.29	0	0	-0.23	0	0
CR_06450W_A	-0.21	0.38	0.23	-0.02	0.96	0.93
C5_03280W_A	-0.04	0.74	0.61	-0.42	0	0
C3_03400C_A	0.12	0.42	0.26	0.15	0.3	0.18
C5_02720W_A	0.31	0.04	0.01	0.21	0.2	0.12
CR_05690W_A	0.58	0	0	0.64	0	0
CR_00230W_A	0.08	0.53	0.36	-0.08	0.5	0.36
C2_02300W_A	-0.33	0.34	0.2	0.11	0.78	0.69
C4_03200C_A	0.54	0	0	0.4	0	0
C4_02140C_A	0.01	0.97	0.95	-0.07	0.59	0.46
C1_08690W_A	-0.02	0.92	0.86	0.06	0.7	0.59
C3_01340W_A	-0.1	0.85	0.75	-0.22	0.68	0.56
CR_06900C_A	0.46	0	0	0.43	0	0

C5_02470W_A	-0.29	0	0	-0.26	0.02	0.01
C7_02810W_A	-0.12	0.29	0.16	-0.56	0	0
C7_02530C_A	-0.42	0.01	0	-0.67	0	0
C3_07170C_A	-0.28	0.03	0.01	-0.07	0.66	0.54
CR_05920C_A	-0.06	0.66	0.51	-0.07	0.61	0.48
C7_02860C_A	-0.01	0.97	0.94	-0.19	0.05	0.02
CR_10190C_A	0.12	0.6	0.43	0.1	0.62	0.48
C1_05150C_A	0.07	0.85	0.76	-0.27	0.47	0.33
C3_00510W_A	0.08	0.77	0.64	0.32	0.1	0.05
C1_14160W_A	-0.01	0.98	0.96	-0.03	0.91	0.87
C5_01850C_A	0.21	0.08	0.03	0.17	0.19	0.11
C3_00840C_A	-0.27	0.08	0.03	-0.5	0	0
C7_04050W_A	0.38	0	0	0.42	0	0
C2_00640W_A	0.34	0.07	0.03	0.21	0.32	0.2
C1_06220C_A	-0.01	0.99	0.98	0.32	0.65	0.52
C5_04530W_A	-0.36	0	0	-0.31	0	0
C4_03750C_A	-0.11	0.64	0.48	0.25	0.19	0.1
C2_02860W_A	0.12	0.11	0.04	-0.08	0.4	0.27
CR_08710W_A	0.43	0	0	0.4	0	0
C1_12130C_A	-0.16	0.49	0.33	0.08	0.75	0.65
C5_00160W_A	0.04	0.83	0.73	-0.04	0.82	0.74
C5_03890C_A	0.07	0.67	0.51	NA	NA	NA
C6_00870C_A	0.06	0.52	0.35	0.17	0.04	0.02
C1_04440W_A	-0.07	0.67	0.52	0.41	0.44	0.31
C3_07550C_A	-0.7	0	0	-1.04	0	0
C4_03280W_A	0.25	0	0	0.14	0.14	0.07
C3_00930W_A	-0.23	0.23	0.12	-0.22	0.2	0.12
C2_03520C_A	-0.17	0.04	0.01	-0.57	0	0
C1_10150W_A	0.6	0.19	0.09	1.45	0	0
C1_08630W_A	-0.5	0	0	-0.56	0	0
C7_03600W_A	-0.23	0.02	0.01	-0.58	0	0
C2_03040W_A	1.47	0	0	2.3	0	0
C5_04460C_A	NA	NA	NA	NA	NA	NA
C1_08870C_A	-0.95	0	0	-1.04	0	0
CR_00620C_A	0.79	0	0	1.62	0	0
C1_09430W_A	0.12	0.48	0.31	-0.22	0.19	0.1
C3_04860W_A	-0.01	0.94	0.9	-0.13	0.37	0.24
C6_02680W_A	0.18	0.34	0.2	0.55	0	0
CR_00170W_A	-0.55	0	0	-1.01	0	0
CR_04930W_A	-0.13	0.54	0.38	0.02	0.95	0.92
C1_08150C_A	-0.25	0.58	0.41	-0.37	0.42	0.29
C1_08790W_A	0.31	0	0	-0.13	0.12	0.06
C2_08790W_A	1.46	0	0	1.69	0	0

CR_05090C_A	0.07	0.85	0.75	0.1	0.86	0.79
C2_09820W_A	-0.73	0	0	-0.58	0	0
C6_00760W_A	0.02	0.93	0.87	-0.19	0.13	0.07
C2_01040W_A	-0.26	0.47	0.3	0.11	0.77	0.67
C2_06890C_A	-0.45	0	0	-0.03	0.84	0.76
C3_00170C_A	0.08	0.64	0.48	-0.46	0	0
C2_05870W_A	0.17	0.44	0.27	0.12	0.66	0.54
CR_10860C_A	0.15	0.11	0.05	0.5	0	0
C5_04110W_A	-0.5	0	0	-0.57	0	0
CR_01110W_A	-0.15	0.09	0.03	-0.12	0.23	0.14
CR_08320W_A	-0.39	0	0	-0.32	0.03	0.01
C2_03020C_A	-0.1	0.32	0.18	0.22	0.04	0.02
C2_05460W_A	0.07	0.55	0.39	-0.32	0	0
C2_09150W_A	0.02	0.93	0.88	0.22	0.08	0.04
CR_01440C_A	-0.19	0.29	0.16	-0.21	0.31	0.19
C7_00740W_A	-0.11	0.36	0.21	0.22	0.05	0.02
C1_06010W_A	0.26	0.07	0.03	1	0	0
C1_13410W_A	-0.08	0.71	0.57	-0.2	0.29	0.18
C1_10430W_A	-0.01	1	0.99	0.07	0.92	0.87
C1_07400C_A	0.05	0.84	0.74	0.36	0.04	0.02
C1_08120W_A	0.02	0.93	0.88	-0.09	0.55	0.41
C2_08540C_A	-0.34	0.23	0.12	-0.09	0.81	0.72
C1_13140C_A	0.02	0.82	0.71	-0.03	0.75	0.65
C1_10610W_A	-0.15	0.4	0.24	-0.21	0.22	0.13
C3_07380W_A	-0.02	0.88	0.8	0.27	0.01	0
C4_04370C_A	0.01	0.97	0.95	-0.11	0.57	0.43
C3_05300C_A	-0.12	0.47	0.31	-0.17	0.25	0.15
C5_01720C_A	-0.18	0.26	0.14	-0.08	0.67	0.55
C5_03830C_A	-0.18	0.24	0.12	-0.11	0.52	0.38
C4_00830W_A	0	1	0.99	0.33	0.04	0.02
C5_00530W_A	0.14	0.72	0.58	0.31	0.61	0.48
C4_00990W_A	0.46	0.21	0.1	1.91	0	0
C5_01310W_A	0.19	0.5	0.34	0.34	0.17	0.09
C1_02960C_A	-0.55	0	0	-0.69	0	0
C4_04020C_A	NA	NA	NA	0.12	0.67	0.55
C4_06710W_A	-0.05	0.71	0.57	-0.37	0.01	0
C1_06280C_A	-0.03	0.96	0.93	0.29	0.62	0.49
C1_10030W_A	-0.49	0	0	-0.46	0	0
CR_02140W_A	-0.4	0	0	-0.27	0.16	0.08
C1_11480W_A	0.55	0	0	-0.29	0.2	0.11
C4_05570C_A	-0.53	0	0	-0.55	0	0
C1_04960C_A	-0.26	0.44	0.28	-0.12	0.8	0.71
C1_06390W_A	-0.11	0.57	0.4	0.03	0.89	0.83

C2_06630C_A	0.47	0.3	0.16	1.68	0	0
C2_02790C_A	-0.17	0.76	0.63	0.61	0.22	0.12
C1_02350W_A	0.02	0.92	0.85	0.14	0.33	0.21
CR_03430W_A	-0.34	0.14	0.07	-0.57	0.02	0.01
C1_10700C_A	0.04	0.71	0.56	0.22	0.02	0.01
C2_02490C_A	-0.14	0.2	0.1	-0.2	0.08	0.04
C1_14480W_A	0.01	0.98	0.96	-0.02	0.96	0.93
C3_02390W_A	-0.14	0.67	0.52	-0.17	0.61	0.48
C3_06400C_A	0.05	0.74	0.6	0.2	0.13	0.06
CR_03840C_A	-0.75	0.03	0.01	-1.18	0	0
C7_01760C_A	-0.34	0.06	0.02	-0.14	0.5	0.36
C2_10270W_A	-0.66	0	0	-0.67	0	0
C5_01950C_A	0.18	0.28	0.15	0.17	0.34	0.22
CR_03600C_A	0.42	0	0	0.65	0	0
C1_04170C_A	-0.12	0.2	0.1	-0.79	0	0
C2_00580C_A	0.36	0	0	0.08	0.45	0.31
C2_09750W_A	-0.01	0.95	0.91	0.02	0.91	0.85
C3_03910W_A	-0.18	0.06	0.02	-0.57	0	0
C1_09900W_A	-0.2	0.7	0.55	0.24	0.74	0.63
C3_03120C_A	0.27	0.5	0.33	0.29	0.48	0.34
CR_00440C_A	0.12	0.28	0.15	0.28	0.01	0
C2_09390W_A	-0.02	0.87	0.78	-0.32	0.01	0
C2_00030W_A	-0.23	0.1	0.04	0.31	0.02	0.01
C5_03530C_A	-0.46	0.18	0.09	-0.23	0.54	0.41
C1_11320C_A	-0.24	0.57	0.41	-0.21	0.76	0.66
C5_03580C_A	0.35	0.01	0	0.52	0	0
C6_03380W_A	-0.65	0	0	-0.45	0	0
C5_01130W_A	0.01	0.94	0.9	-0.25	0.14	0.07
CR_09270C_A	0.25	0	0	-0.05	0.64	0.51
CR_01270C_A	-0.38	0.08	0.03	-0.2	0.33	0.21
C4_01690C_A	-0.05	0.6	0.43	-0.19	0.05	0.02
C3_04520C_A	-0.65	0	0	-1.12	0	0
C4_04420W_A	0.1	0.58	0.42	0.25	0.1	0.05
C1_14320C_A	0.11	0.27	0.14	0.07	0.65	0.52
C5_04440C_A	0.11	0.82	0.72	0.39	0.39	0.26
C5_04350C_A	-0.09	0.55	0.39	-0.13	0.32	0.2
CR_10430C_A	-0.18	0.6	0.44	-0.08	0.81	0.73
C1_08090C_A	-0.29	0	0	-0.44	0	0
C2_08370C_A	0.25	0	0	-0.08	0.45	0.32
C7_01100C_A	0.47	0	0	1.01	0	0
CR_09160C_A	0.15	0.06	0.02	-0.18	0.04	0.02
C1_02620C_A	0.39	0	0	0.11	0.27	0.16
CR_03010C_A	0.11	0.14	0.06	-0.14	0.15	0.08

C5_02920W_A	0.15	0.32	0.18	0.38	0.01	0
C2_10280C_A	0.15	0.09	0.04	0.18	0.09	0.04
C6_03100W_A	0.17	0.15	0.07	0.42	0	0
C3_07730W_A	-0.66	0	0	-0.93	0	0
C6_01560W_A	0.12	0.74	0.61	-0.61	0.06	0.03
C6_02620C_A	0.13	0.82	0.71	0.03	0.97	0.95
C1_01570C_A	-0.38	0.02	0.01	-0.43	0.01	0
C4_02770C_A	-0.24	0.21	0.1	0.13	0.52	0.39
C1_02630C_A	-0.07	0.81	0.7	-1.45	0	0
CR_03900W_A	0.07	0.61	0.45	0.21	0.07	0.03
C7_03940C_A	0	1	1	0.12	0.5	0.36
C1_07580C_A	0.12	0.8	0.69	-0.23	0.69	0.57
C5_04020C_A	0.13	0.68	0.53	0.22	0.66	0.54
C2_06270W_A	-0.21	0.58	0.42	-0.22	0.56	0.42
C1_05460W_A	0.18	0.11	0.04	0.46	0	0
C7_02550C_A	-0.29	0.18	0.08	-0.04	0.87	0.81
CR_05840W_A	-0.16	0.46	0.29	-0.29	0.15	0.08
C3_00580W_A	0.22	0.11	0.05	0.39	0	0
C1_00800C_A	-1.21	0	0	-2.5	0	0
C2_06510W_A	0.22	0.27	0.14	0.28	0.14	0.07
C7_01660C_A	-0.47	0	0	-0.19	0.12	0.06
CR_01070W_A	-0.04	0.89	0.81	-0.19	0.35	0.23
C4_02340W_A	0.02	0.91	0.85	0.63	0	0
C1_03340C_A	1.12	0	0	1.24	0	0
C2_03380W_A	0.09	0.31	0.17	0.03	0.8	0.71
C7_02050C_A	-0.11	0.51	0.35	-0.02	0.92	0.87
CR_08540C_A	-0.11	0.44	0.28	-0.06	0.7	0.58
C2_03890W_A	-0.27	0.28	0.15	-0.22	0.41	0.28
C4_04360W_A	0.22	0.63	0.46	0.2	0.69	0.57
C1_04470C_A	1.2	0	0	2.35	0	0
C5_05380W_A	0.24	0	0	0.38	0	0
C2_02260W_A	0.18	0.11	0.04	0.41	0	0
C5_00570W_A	0.15	0.23	0.11	0.41	0	0
C1_02300W_A	-0.13	0.77	0.64	0.1	0.81	0.72
C2_07740W_A	0.08	0.46	0.3	0.27	0.01	0.01
C3_00890C_A	-0.15	0.4	0.24	0.35	0.01	0
C1_02010C_A	0.76	0	0	0.49	0	0
CR_04270C_A	0.4	0.37	0.22	0.63	0.16	0.08
CR_04620C_A	0.07	0.85	0.75	0.11	0.86	0.79
CR_10440W_A	-0.38	0.05	0.02	-0.1	0.65	0.52
C2_00370W_A	-0.23	0.04	0.01	-0.55	0	0
C1_10930C_A	0.32	0	0	0.06	0.65	0.52
CR_08340W_A	-0.76	0	0	-1.2	0	0

C3_04040W_A	NA	NA	NA	0.24	0.51	0.38
C1_07850C_A	-0.14	0.68	0.53	0.07	0.83	0.76
CR_09710W_A	-0.41	0.04	0.01	-0.2	0.35	0.23
C4_02600C_A	-0.45	0	0	-0.12	0.57	0.43
C1_03640C_A	0.09	0.68	0.53	0.3	0.12	0.06
C4_02550C_A	0.25	0.04	0.01	0.19	0.14	0.07
C4_00040W_A	0.18	0.02	0.01	-0.25	0.01	0
C5_00310C_A	-1.01	0	0	-1.29	0	0
C1_05620C_A	0	1	0.99	0.46	0.14	0.07
CR_07260C_A	NA	NA	NA	0.12	0.67	0.55
C3_00140W_A	0.55	0.24	0.13	0.76	0.2	0.11
C6_01980C_A	-0.09	0.31	0.17	-0.26	0	0
CR_07190W_A	0.75	0	0	0.93	0	0
C1_01040W_A	0.56	0	0	0.87	0	0
C1_14060W_A	0	0.98	0.97	0.7	0	0
C1_04860W_A	0	0.98	0.97	0.06	0.64	0.51
C7_01060W_A	-0.13	0.54	0.38	-0.24	0.5	0.37
C7_03680W_A	-0.04	0.88	0.79	0.07	0.75	0.64
C5_00800C_A	-0.18	0.04	0.01	-0.42	0	0
C7_00280W_A	0.42	0.18	0.08	0.71	0.16	0.08
C5_02190C_A	0.28	0.12	0.05	0.23	0.24	0.14
C4_01490W_A	-0.29	0	0	-0.43	0	0
C5_01710C_A	-0.34	0.19	0.09	0.05	0.86	0.8
C2_05530C_A	0.07	0.58	0.42	0.09	0.49	0.35
C2_05640W_A	0.52	0	0	0.63	0	0
C4_00120W_A	-1.65	0	0	-2.85	0	0
C3_00010C_A	0.74	0	0	0.61	0	0
CR_10740W_A	0.08	0.68	0.54	0.12	0.55	0.41
C3_05190C_A	0.67	0	0	1.08	0	0
C5_00030W_A	0.59	0	0	0.83	0	0
C6_03630W_A	-0.75	0	0	-0.8	0	0
C3_01910C_A	-0.28	0.59	0.43	-0.38	0.58	0.45
C7_00890C_A	0.08	0.55	0.39	-0.06	0.66	0.53
C5_00840W_A	0.32	0.05	0.02	0.56	0	0
C7_02030W_A	0.12	0.17	0.08	0.06	0.53	0.39
C7_01130C_A	NA	NA	NA	NA	NA	NA
C1_08760W_A	0.38	0.01	0	0.08	0.7	0.59
C7_02490W_A	-0.15	0.42	0.26	0.08	0.71	0.59
C1_02990C_A	-1.03	0	0	-1.33	0	0
CR_04940W_A	-0.07	0.89	0.81	-0.17	0.81	0.72
C2_08400C_A	NA	NA	NA	NA	NA	NA
C1_13270W_A	-0.05	0.62	0.45	-0.07	0.54	0.4
C1_13630W_A	-0.18	0.25	0.13	-0.11	0.52	0.38

C3_02190C_A	-0.12	0.45	0.29	0.09	0.55	0.41
C2_06130W_A	-0.21	0.16	0.08	-0.27	0.08	0.04
C3_06630W_A	-0.22	0.43	0.27	0.25	0.33	0.21
C3_07120W_A	0.14	0.68	0.53	0.3	0.3	0.19
C2_10090C_A	-0.93	0	0	-0.92	0	0
C1_10530W_A	0.04	0.83	0.73	-0.05	0.81	0.73
C1_05490C_A	0.48	0	0	0.98	0	0
C2_04620W_A	-0.42	0.05	0.02	-0.3	0.42	0.29
C1_06850W_A	-0.12	0.35	0.21	-0.53	0	0
C7_01460C_A	-0.4	0.37	0.22	-0.09	0.79	0.7
C6_02810C_A	0.11	0.41	0.25	0.31	0.01	0
C6_00560W_A	0.01	0.97	0.94	-0.31	0.01	0
C6_01110W_A	0.11	0.24	0.12	-0.07	0.47	0.34
C5_05070W_A	-0.6	0	0	-0.24	0.32	0.21
C1_07810C_A	-0.53	0.01	0	-0.8	0	0
C3_04800C_A	0.31	0.03	0.01	0.2	0.22	0.13
C1_09840C_A	-0.11	0.46	0.3	0.27	0.03	0.01
CR_03270W_A	-0.13	0.67	0.51	-0.16	0.59	0.46
C7_00520W_A	0.06	0.62	0.46	0.04	0.77	0.67
C2_08160C_A	0.24	0	0	0.08	0.56	0.42
C2_07480W_A	-0.21	0.08	0.03	-0.66	0	0
C3_05290C_A	-0.77	0	0	-0.83	0	0
C2_00810C_A	-0.03	0.91	0.84	-0.21	0.34	0.22
C6_03180C_A	-0.04	0.74	0.61	0.16	0.14	0.07
C2_03120W_A	-0.38	0	0	-1.42	0	0
C1_10960W_A	-0.14	0.55	0.38	-0.14	0.52	0.39
C3_05050W_A	-0.07	0.68	0.53	0	1	0.99
C1_08370W_A	0.12	0.16	0.08	0.19	0.06	0.03
C7_00600C_A	-0.02	0.91	0.85	0.05	0.76	0.66
C6_02180W_A	0.09	0.74	0.61	0.16	0.51	0.37
C4_02800W_A	-0.49	0	0	0	1	1
C4_01950W_A	0.22	0.01	0	0.24	0.01	0.01
CR_03850W_A	-0.19	0.52	0.35	-0.11	0.72	0.61
C2_07640W_A	-0.36	0	0	-0.47	0	0
C2_05820W_A	-0.05	0.8	0.69	-0.23	0.17	0.09
CR_10490W_A	0.13	0.13	0.06	0.1	0.35	0.23
C6_01570C_A	-0.29	0.21	0.1	0.2	0.42	0.29
CR_02070C_A	0.26	0	0	0.72	0	0
CR_10640W_A	0.02	0.96	0.92	0.33	0.17	0.09
C2_02310W_A	0.08	0.62	0.46	0.11	0.45	0.32
C3_02600C_A	0.31	0	0	0.07	0.62	0.49
C5_03030W_A	-0.09	0.73	0.6	-0.03	0.92	0.88
C3_07490W_A	0.13	0.15	0.07	0.13	0.18	0.1

CR_01660C_A	-0.26	0.01	0	-0.27	0.01	0
CR_04130C_A	-0.24	0.05	0.02	-0.11	0.41	0.28
C1_11290W_A	-0.01	0.98	0.96	0.1	0.73	0.62
C2_09950W_A	0.43	0	0	0.16	0.1	0.05
C1_07090C_A	-0.23	0.08	0.03	0.11	0.47	0.34
C1_00490C_A	0.19	0.14	0.06	0.64	0	0
C5_00150C_A	0.05	0.74	0.6	0	0.99	0.98
C4_06510C_A	0.39	0.02	0.01	0.6	0	0
C5_04880C_A	-0.2	0.32	0.18	-0.02	0.93	0.88
C3_02100W_A	0.13	0.38	0.23	0.41	0	0
C1_04110W_A	0.1	0.72	0.58	-0.02	0.94	0.9
C1_00350C_A	0.52	0	0	0.87	0	0
C3_00610W_A	-0.21	0.22	0.11	-0.14	0.43	0.29
C1_09030C_A	0.29	0	0	0.41	0	0
C3_06880W_A	0.94	0	0	1.21	0	0
C2_06750C_A	0.17	0.53	0.36	0.03	0.91	0.87
C2_04570W_A	-1.19	0	0	-1.34	0	0
C3_03890W_A	-0.02	0.92	0.85	0.1	0.47	0.33
C2_00700W_A	0.14	0.11	0.05	0.03	0.78	0.68
C5_01550C_A	-0.05	0.57	0.41	-0.54	0	0
C2_03610W_A	0.25	0.59	0.42	1.07	0	0
CR_07810W_A	-0.22	0.62	0.46	0.61	0.08	0.04
C4_01470W_A	-0.13	0.54	0.37	-0.02	0.93	0.89
C5_01280C_A	-0.31	0.12	0.05	-0.59	0	0
C1_02800W_A	-0.34	0.46	0.29	-0.62	0.18	0.1
C5_02820C_A	-0.12	0.42	0.26	-0.27	0.06	0.03
C5_01080C_A	0.03	0.79	0.67	-0.03	0.81	0.72
CR_02720C_A	-0.41	0.06	0.02	-0.59	0	0
C2_00910W_A	0.17	0.65	0.49	0.43	0.46	0.33
CR_01150C_A	-0.21	0.17	0.08	-0.01	0.98	0.96
C2_08000C_A	-0.13	0.14	0.06	-0.66	0	0
C1_11530C_A	-0.66	0	0	-0.98	0	0
C2_03710W_A	0.07	0.67	0.51	1.3	0.02	0.01
C1_07670W_A	0.06	0.91	0.84	0.49	0.44	0.31
C3_06230W_A	0.29	0	0	0.25	0.02	0.01
C3_02070C_A	0.06	0.92	0.86	0.73	0.13	0.07
C7_01600W_A	-0.02	0.89	0.81	-0.29	0	0
CR_10340W_A	-0.63	0	0	-0.29	0	0
C5_05140W_A	-0.04	0.78	0.65	-0.28	0.02	0.01
CR_08000C_A	0.32	0	0	0.26	0.01	0
C1_06960W_A	-0.21	0.01	0	-0.27	0	0
C6_02090C_A	0.06	0.77	0.64	0.61	0	0
C4_00140C_A	0.1	0.24	0.12	-0.35	0	0

C1_06480C_A	0.42	0	0	0.69	0	0
C1_03450C_A	-0.11	0.33	0.19	-0.34	0	0
C1_01140C_A	0.1	0.33	0.19	-0.15	0.14	0.08
C3_04410C_A	0.18	0.14	0.06	-0.12	0.37	0.24
C2_00880W_A	-0.16	0.36	0.21	-0.06	0.75	0.64
C2_07800W_A	-0.08	0.73	0.6	-0.35	0.07	0.03
C2_01310W_A	0	0.99	0.97	-0.01	0.98	0.96
C2_04860W_A	-0.32	0.35	0.21	0.11	0.77	0.67
CR_07800W_A	-0.15	0.73	0.59	0.01	0.99	0.98
C1_07540C_A	0.24	0.01	0	0.1	0.37	0.24
C5_00770C_A	0.05	0.78	0.65	0.23	0.12	0.06
C4_05450C_A	-0.01	0.96	0.93	-0.32	0	0
C2_03620W_A	0.08	0.72	0.58	0.1	0.64	0.51
C1_04290C_A	0.26	0	0	0.17	0.03	0.01
C6_00880W_A	-0.22	0.2	0.1	-0.2	0.2	0.11
C1_02230W_A	0.2	0.07	0.03	0.19	0.12	0.06
CR_00120C_A	0.34	0	0	0.81	0	0
C4_05890W_A	-0.17	0.53	0.36	0.03	0.93	0.89
C2_09900C_A	-0.31	0	0	-0.6	0	0
C1_02170C_A	-0.01	0.98	0.96	0.15	0.58	0.44
CR_05710C_A	-0.34	0.18	0.08	-0.28	0.28	0.17
C2_03030W_A	-0.36	0.01	0	-0.54	0	0
C2_01830W_A	-0.88	0	0	-0.16	0.53	0.39
CR_04630C_A	0.13	0.67	0.51	0.18	0.54	0.4
C5_04480C_A	0.26	0.44	0.28	1.32	0.02	0.01
C2_07510W_A	0.2	0.48	0.31	0.08	0.78	0.69
C1_08350C_A	0.4	0.01	0	0.32	0.06	0.03
C4_04670C_A	0.21	0.2	0.1	0.09	0.65	0.51
C7_04020C_A	0.25	0	0	0.53	0	0
C2_00420W_A	0.24	0.11	0.05	0.34	0.03	0.01
C5_04170W_A	-0.29	0.01	0	-0.46	0	0
CR_05050W_A	-0.17	0	0	-0.43	0	0
CR_05630W_A	-0.55	0.01	0	-0.37	0.13	0.06
C4_00170W_A	0.22	0.03	0.01	0.21	0.03	0.01
C6_02440C_A	-0.27	0.37	0.22	-0.03	0.93	0.89
C1_13730C_A	-1.36	0	0	-1.05	0.01	0
C1_01070C_A	-0.52	0.01	0	-0.67	0	0
C2_08030W_A	0.24	0.5	0.33	0.44	0.12	0.06
C4_02940W_A	0.45	0	0	0.81	0	0
C3_02860W_A	-0.15	0.4	0.25	-0.16	0.4	0.27
C2_09020W_A	-0.18	0.06	0.02	-0.12	0.27	0.16
CR_00630W_A	-0.1	0.79	0.67	0.33	0.28	0.17
C3_06160C_A	-1.18	0	0	-1.47	0	0

C1_13810W_A	-0.46	0	0	-0.18	0.19	0.11
C5_05030C_A	0.24	0.07	0.03	0.19	0.18	0.1
C6_04330W_A	-1.25	0	0	-0.53	0.11	0.06
C2_06230W_A	0.08	0.5	0.33	0.06	0.65	0.52
C3_06830C_A	-0.17	0.29	0.16	-0.26	0.05	0.02
C3_06500W_A	0.35	0	0	0.36	0	0
CR_07330W_A	-0.65	0.01	0	-0.32	0.26	0.16
CR_07830C_A	0.31	0.07	0.03	0.48	0	0
C1_11100W_A	0.26	0.25	0.13	0.52	0.01	0
C4_06070C_A	-0.05	0.83	0.73	-0.07	0.74	0.64
C6_04480C_A	0.34	0	0	0.45	0	0
C3_02690C_A	-0.2	0.1	0.04	-0.09	0.54	0.4
C2_09420W_A	-0.44	0	0	-0.3	0.06	0.03
C2_10340W_A	-0.15	0.44	0.28	-0.34	0.06	0.03
CR_04380C_A	0.75	0	0	0.92	0	0
C7_00100W_A	-0.09	0.55	0.39	0.06	0.71	0.6
C1_09550W_A	-0.07	0.67	0.52	0.12	0.81	0.73
C3_05630W_A	0.28	0.59	0.43	-0.05	0.95	0.92
C5_02380W_A	-0.43	0	0	-0.23	0	0
C4_05660C_A	-0.46	0	0	-0.48	0	0
C2_09230C_A	0.29	0	0	0.49	0	0
CR_04220C_A	-0.43	0.01	0	-0.48	0	0
C1_12880C_A	-0.24	0.27	0.14	-0.11	0.64	0.51
C4_03580W_A	-0.15	0.57	0.41	0.01	0.99	0.98
C2_09990C_A	0.28	0.02	0	0.16	0.28	0.17
C6_00300C_A	0.57	0	0	0.61	0	0
C4_06390W_A	-1.69	0	0	-1.45	0	0
C6_01410C_A	-0.3	0.02	0	-0.41	0	0
C3_00190W_A	0.2	0.43	0.27	NA	NA	NA
C6_03520C_A	0.15	0.37	0.22	0.19	0.25	0.15
CR_08720W_A	0.54	0.22	0.11	0.39	0.49	0.35
C2_02630W_A	-0.12	0.69	0.55	-0.15	0.62	0.49
C3_04900W_A	-1.24	0	0	-0.88	0	0
C1_07130C_A	-0.47	0	0	-0.25	0.16	0.09
C1_08200W_A	-0.31	0.26	0.14	0.13	0.66	0.54
C2_08580W_A	-0.04	0.95	0.91	0.14	0.82	0.74
C1_08430W_A	0.31	0	0	0.17	0.08	0.04
C3_02960C_A	0.68	0	0	0.03	0.86	0.79
C2_09480W_A	-0.06	0.82	0.72	0.22	0.29	0.18
C1_14350W_A	-0.03	0.88	0.8	-0.05	0.75	0.65
C3_00660W_A	-0.11	0.6	0.43	-0.02	0.93	0.89
C5_05470W_A	-0.03	0.83	0.73	0.09	0.49	0.35
C1_04940C_A	0.02	0.97	0.95	0.38	0.56	0.42

C4_07040W_A	-0.34	0.35	0.21	-0.12	0.77	0.67
C1_11590W_A	-0.02	0.84	0.75	-0.2	0.02	0.01
C2_03010C_A	0.11	0.13	0.06	-0.04	0.73	0.61
C1_02730W_A	0.31	0.29	0.16	0.96	0	0
CR_09150W_A	-0.42	0.11	0.05	-0.28	0.32	0.2
C6_01650C_A	-0.12	0.42	0.26	-0.25	0.04	0.02
C7_02960C_A	0.21	0.18	0.08	0.13	0.44	0.31
C2_08200W_A	-0.29	0.1	0.04	-0.34	0.06	0.03
C4_01270W_A	-0.29	0	0	-0.67	0	0
C3_04200W_A	0.45	0.25	0.13	0.89	0.01	0
C1_04340C_A	0.11	0.71	0.56	0.46	0.05	0.02
CR_09850C_A	-0.07	0.82	0.71	-0.28	0.47	0.33
CR_09950C_A	-0.55	0	0	-1.1	0	0
C1_10440W_A	-0.45	0	0	-0.13	0.5	0.36
C5_02630C_A	0.26	0.01	0	0.7	0	0
BGI_novel_G000	0.37	0.01	0	-0.38	0.02	0.01
C6_01360W_A	-0.72	0	0	-1.05	0	0
C1_09010W_A	-0.1	0.69	0.54	-0.36	0.11	0.05
C4_03530W_A	0.21	0.34	0.2	0.3	0.18	0.1
C4_03400W_A	-0.01	0.96	0.92	-0.04	0.84	0.76
C5_01760C_A	0.47	0	0	0.62	0	0
C1_12790C_A	-0.63	0	0	-0.55	0.01	0
C2_03930C_A	-0.67	0	0	-0.39	0.04	0.02
C2_02770W_A	0	0.98	0.95	0	1	0.99
C3_03050C_A	0.42	0	0	0.3	0.05	0.02
C5_03470C_A	0.26	0.05	0.02	0.31	0.02	0.01
CR_04520W_A	-0.05	0.83	0.73	-0.04	0.86	0.8
C1_09150W_A	-0.88	0.03	0.01	-1.56	0	0
C4_07220C_A	0.71	0	0	0.91	0	0
C4_04580W_A	0.31	0	0	0.56	0	0
C2_06100W_A	1.18	0	0	0.77	0	0
CR_04410W_A	-0.48	0.1	0.04	-0.37	0.22	0.12
CR_06680C_A	-0.4	0.02	0.01	-0.08	0.72	0.61
CR_04680C_A	0.69	0.05	0.02	1.35	0	0
C2_04820W_A	-0.39	0.02	0	-0.37	0.01	0
CR_06050W_A	0.25	0.02	0.01	0.45	0	0
C7_01780W_A	-0.31	0	0	-0.28	0.01	0
C3_00030C_A	0.74	0	0	0.56	0	0
C7_04290W_A	0.02	0.94	0.88	-0.02	0.92	0.87
C3_04720C_A	0.26	0.06	0.02	0.2	0.15	0.08
C7_01220W_A	0.04	0.79	0.68	-0.01	0.95	0.92
C2_09630C_A	1.2	0	0	1.41	0	0
C1_02700C_A	1.03	0	0	2.02	0	0

C1_07060C_A	-0.03	0.94	0.88	0.03	0.92	0.88
C6_00230W_A	0.05	0.88	0.8	-0.03	0.93	0.89
C2_02540W_A	-0.67	0	0	-1.08	0	0
C7_04150W_A	0.2	0.32	0.18	0.3	0.1	0.05
C1_02770W_A	-0.23	0.55	0.39	0.03	0.96	0.93
C2_06720W_A	0.07	0.71	0.57	0.1	0.59	0.45
C1_10920W_A	-0.37	0.03	0.01	-0.4	0.02	0.01
C6_03770C_A	0.02	0.95	0.92	0.11	0.65	0.52
C7_03410C_A	-0.51	0	0	-0.82	0	0
C6_03840C_A	-0.88	0	0	-0.99	0	0
C2_05070W_A	-0.52	0.26	0.14	-0.07	0.91	0.85
C2_00770W_A	0.11	0.49	0.32	0.04	0.83	0.76
C1_08660C_A	-0.19	0.47	0.3	-0.01	0.97	0.94
C4_00490W_A	0.09	0.71	0.57	0.42	0.05	0.02
C2_07230C_A	-0.07	0.74	0.61	0.11	0.56	0.42
C1_13260W_A	0.02	0.91	0.85	0.16	0.16	0.09
CR_07440W_A	-0.79	0	0	-1.11	0	0
C7_03130C_A	0.18	0.41	0.25	0.47	0.01	0
C1_08390C_A	0.02	0.91	0.84	0.09	0.53	0.39
C2_10710W_A	-0.08	0.43	0.27	-0.29	0	0
C2_01010W_A	-0.39	0	0	-0.94	0	0
C5_01400W_A	0.4	0	0	0.33	0.01	0
C3_07110W_A	-0.81	0	0	-1.1	0	0
C1_06590C_A	-0.09	0.41	0.25	-0.13	0.23	0.14
C3_04170W_A	-0.51	0.2	0.1	-0.75	0.07	0.03
C2_00750W_A	1.51	0	0	3.23	0	0
C4_05690W_A	1.06	0	0	1.32	0	0
C7_02650W_A	-0.08	0.82	0.71	0.18	0.56	0.43
C2_05020W_A	-0.23	0.28	0.15	0.1	0.66	0.54
C1_08930C_A	0.1	0.68	0.54	0.35	0.08	0.04
CR_03760W_A	-0.17	0.21	0.11	0.01	0.96	0.94
C5_02690W_A	0.49	0	0	0.42	0	0
C3_01000W_A	-1.17	0	0	-1.53	0	0
C1_03070C_A	0.15	0.22	0.11	0.28	0.02	0.01
C1_02310C_A	-0.21	0.5	0.33	-0.04	0.9	0.85
C3_03100C_A	0.14	0.23	0.12	0.21	0.09	0.04
C7_01840W_A	-0.72	0	0	-0.92	0	0
C1_12170C_A	0.02	0.92	0.85	0.18	0.2	0.12
C5_02320C_A	-0.24	0.24	0.13	-0.1	0.64	0.5
C3_01070C_A	0.01	0.97	0.93	-0.01	0.97	0.95
C4_04090C_A	0.22	0.08	0.03	-0.18	0.2	0.11
C4_02220C_A	-0.29	0.58	0.42	-0.98	0.09	0.04
C1_13910C_A	-0.13	0.54	0.37	0.06	0.76	0.66

C1_00150C_A	0.41	0	0	0.97	0	0
CR_02610C_A	-0.11	0.32	0.18	-0.19	0.06	0.03
C2_05200C_A	-0.12	0.56	0.39	-0.06	0.77	0.67
C3_06380W_A	-1.18	0	0	-1.47	0	0
C6_03710W_A	0.14	0.29	0.16	-0.41	0	0
C6_03200W_A	-0.13	0.38	0.23	0.02	0.9	0.85
CR_05180C_A	0.3	0	0	0.25	0.02	0.01
C4_04890C_A	-0.04	0.68	0.53	-0.42	0	0
C5_03780C_A	0.21	0.18	0.09	-0.02	0.93	0.89
C6_04590C_A	0.27	0.03	0.01	0.41	0	0
CR_04340W_A	0.21	0	0	0.29	0	0
C1_02480W_A	0.32	0	0	0.18	0.05	0.02
C2_01470W_A	0.18	0.09	0.04	0.13	0.27	0.17
CR_00570W_A	-0.4	0	0	-0.41	0	0
C3_05250C_A	0	1	0.99	-0.15	0.36	0.24
C1_04970W_A	-0.29	0	0	-0.4	0	0
C2_01520W_A	0.34	0	0	0.28	0.03	0.01
CR_10590W_A	0.32	0	0	0.28	0.01	0
C2_02870W_A	0.23	0.13	0.05	0.38	0.01	0
C6_02550W_A	0.41	0.39	0.24	0.1	0.88	0.82
C5_04920C_A	0.37	0	0	0.54	0	0
C3_07810C_A	1.39	0	0	1	0	0
C2_06070W_A	0.65	0	0	1.18	0	0
C4_01400W_A	-0.07	0.67	0.52	-0.13	0.65	0.52
C3_02300W_A	0.01	0.97	0.94	-0.2	0.21	0.12
CR_04810W_A	0.03	0.78	0.65	-0.59	0	0
C1_09770W_A	-0.33	0.07	0.03	-0.21	0.29	0.18
C4_01710C_A	-0.08	0.69	0.55	-0.15	0.43	0.3
C1_02420C_A	0.77	0	0	0.95	0	0
C7_02130W_A	0.17	0.75	0.62	0.51	0.43	0.3
C1_09250W_A	-0.5	0	0	-1.13	0	0
C1_06540C_A	-0.13	0.51	0.35	-0.11	0.56	0.42
CR_08170C_A	0	1	0.99	0.01	0.95	0.92
C2_07890W_A	-0.36	0.01	0	-0.37	0.01	0
C1_06370C_A	0.07	0.67	0.51	NA	NA	NA
C4_03440C_A	-0.29	0.11	0.05	0.56	0	0
C4_01900C_A	0.34	0	0	0.36	0	0
C1_04990C_A	-0.31	0.11	0.05	-0.07	0.78	0.68
C1_01950C_A	-0.09	0.6	0.43	-0.41	0.01	0
C1_12760W_A	-0.4	0.01	0	-0.27	0.16	0.09
C4_02670W_A	0.09	0.7	0.55	0.12	0.6	0.47
C6_03580W_A	0.4	0.01	0	0.49	0	0
C6_00360C_A	-0.11	0.54	0.37	-0.14	0.42	0.29

C3_06660C_A	-0.16	0.77	0.64	0.14	0.84	0.77
C2_04530W_A	-0.23	0.44	0.28	0.02	0.97	0.94
CR_00350W_A	0.16	0.05	0.02	0	0.98	0.96
CR_02380C_A	-0.37	0.11	0.05	-0.58	0.01	0
C2_06420C_A	0.28	0	0	0.48	0	0
C1_03370W_A	-0.23	0	0	-0.79	0	0
CR_02540W_A	0.15	0.55	0.39	0.62	0	0
C1_07030C_A	0.99	0	0	2.03	0	0
CR_01220W_A	-0.05	0.91	0.84	0.07	0.92	0.87
C1_08550C_A	0.07	0.51	0.35	0.01	0.97	0.95
C7_02920W_A	0.26	0.52	0.35	0.4	0.31	0.19
C7_04360C_A	-0.2	0.24	0.13	-0.54	0	0
C7_00360W_A	0.47	0	0	1.29	0	0
C7_03010W_A	-0.22	0.05	0.02	-0.07	0.69	0.57
CR_04010C_A	-0.18	0.03	0.01	-0.23	0.02	0.01
C2_10430C_A	-0.66	0	0	0.07	0.78	0.68
CR_04840C_A	-0.16	0.47	0.31	-0.31	0.11	0.06
C2_08530C_A	-0.55	0.07	0.03	-0.45	0.19	0.1
C7_02100W_A	0.09	0.28	0.15	0.16	0.09	0.04
C3_02430W_A	0.44	0	0	0.6	0	0
CR_00660W_A	0.56	0.09	0.04	0.26	0.54	0.4
C1_13850C_A	0.94	0.03	0.01	1.17	0.03	0.01
C7_00830C_A	0.06	0.62	0.45	0.04	0.72	0.61
CR_00320C_A	0.26	0	0	0.54	0	0
C4_06380W_A	0.12	0.69	0.55	-0.01	0.98	0.96
C2_02110C_A	-0.05	0.89	0.82	0.35	0.18	0.1
C1_01800W_A	-0.13	0.45	0.29	-0.35	0.02	0.01
C1_00610W_A	0.19	0.02	0.01	0.18	0.06	0.03
C2_07110C_A	-0.35	0.01	0	0.07	0.67	0.55
C5_03790W_A	-0.39	0.01	0	-0.64	0	0
C3_03370C_A	-0.41	0.03	0.01	-0.23	0.24	0.14
C7_02210W_A	0.3	0.52	0.35	0.9	0.13	0.07
CR_09500C_A	0.29	0.03	0.01	0.36	0.01	0
C2_08180C_A	-0.69	0	0	-0.36	0.19	0.11
C2_05850C_A	0.1	0.55	0.38	0.34	0.02	0.01
C3_07800C_A	-0.34	0.09	0.03	-0.16	0.47	0.34
C2_03430W_A	-0.15	0.49	0.32	0	1	1
C4_03560W_A	-0.02	0.92	0.86	0.05	0.77	0.67
CR_07110C_A	-0.01	0.98	0.97	-0.17	0.52	0.38
C7_01250W_A	0	0.99	0.98	-0.61	0	0
C7_01830W_A	-0.16	0.6	0.44	-0.57	0.03	0.01
C1_06700W_A	-0.32	0.01	0	-0.27	0.02	0.01
C2_05410W_A	-0.2	0	0	-0.57	0	0

CR_08980C_A	0.15	0.28	0.15	0.24	0.06	0.03
C2_02810C_A	-0.33	0.02	0.01	-0.47	0	0
C2_00410C_A	-0.7	0	0	-0.5	0.01	0
C1_05090W_A	-0.06	0.78	0.66	0.09	0.65	0.52
C3_03830W_A	0.2	0.28	0.15	0.36	0.04	0.02
CR_06780W_A	-0.44	0	0	-0.55	0	0
C6_01390W_A	-0.04	0.78	0.65	-0.55	0	0
C3_00650W_A	0.19	0.65	0.49	0.32	0.43	0.3
C1_10050W_A	0.12	0.75	0.62	0.63	0.29	0.18
C6_02850W_A	0.03	0.9	0.83	-0.03	0.88	0.82
C4_00350W_A	0.42	0.01	0	0.53	0	0
C4_01090C_A	-0.31	0.28	0.15	-0.24	0.42	0.29
C6_00680C_A	-0.42	0.01	0	-0.43	0.01	0
C4_06850C_A	0	0.98	0.95	0.13	0.22	0.13
C1_00600W_A	0.22	0.07	0.03	0.12	0.44	0.31
CR_08430W_A	0.08	0.49	0.33	-0.1	0.36	0.24
CR_04650W_A	-0.1	0.65	0.49	0.04	0.86	0.79
C1_00040W_A	-0.06	0.62	0.45	-0.1	0.38	0.25
C5_01300C_A	0.08	0.8	0.69	0.3	0.23	0.14
C1_08440C_A	-0.33	0.28	0.15	-0.45	0.14	0.08
C4_04910C_A	-0.29	0.19	0.09	-0.44	0.05	0.02
CR_01120C_A	0.24	0.04	0.01	0.37	0	0
C3_03720W_A	0.71	0	0	1.35	0	0
C7_03400C_A	-0.75	0	0	-0.65	0	0
C1_01550W_A	0.34	0.01	0	0.49	0	0
C1_09210C_A	-0.02	0.86	0.76	0	0.99	0.97
C1_01510W_A	1.3	0	0	1.29	0.01	0
CR_00240W_A	0.07	0.74	0.6	0.11	0.55	0.41
C1_05020C_A	-0.08	0.63	0.47	-0.12	0.49	0.35
C5_03950W_A	-0.34	0.04	0.02	0.23	0.22	0.13
C3_07260C_A	0.31	0	0	-0.02	0.89	0.83
C2_05210W_A	-0.03	0.95	0.9	-0.13	0.7	0.59
C1_12280C_A	-0.47	0.01	0	-0.32	0.07	0.03
C1_09730W_A	-0.09	0.66	0.5	-0.08	0.69	0.57
C4_06200W_A	-0.17	0.76	0.62	0.42	0.4	0.27
C2_01130W_A	-0.22	0.03	0.01	-0.14	0.23	0.14
C2_05110W_A	-0.12	0.2	0.1	-1.1	0	0
C6_03350C_A	-0.28	0.01	0	-0.07	0.66	0.54
CR_05830C_A	0.17	0.42	0.26	0.06	0.79	0.69
CR_09170C_A	-0.11	0.58	0.42	0.38	0.01	0
CR_04470W_A	0.15	0.63	0.47	0.34	0.19	0.1
C1_10000C_A	0.23	0.09	0.04	0.77	0	0
C6_03010W_A	-0.19	0.14	0.06	-0.07	0.64	0.51

C5_04400W_A	-0.2	0.72	0.57	0.35	0.6	0.47
C2_10830W_A	-0.16	0.62	0.45	-0.35	0.28	0.17
C4_04070C_A	-0.07	0.82	0.72	-0.27	0.47	0.33
C6_01440C_A	-0.1	0.81	0.7	-0.43	0.42	0.29
C2_06680W_A	-0.31	0.17	0.08	-0.17	0.5	0.36
CR_00400C_A	-0.1	0.4	0.24	0.09	0.49	0.35
CR_02870W_A	0.57	0	0	0.7	0	0
C2_08070C_A	0.11	0.46	0.29	0.16	0.32	0.21
CR_05340C_A	0.15	0.05	0.02	-0.46	0	0
C1_14290C_A	-0.16	0.02	0.01	-0.68	0	0
C3_07830W_A	1.17	0	0	1.35	0	0
C2_01230W_A	0.12	0.57	0.4	0.26	0.16	0.09
C2_05150W_A	0.16	0.38	0.23	0.14	0.44	0.31
C7_00450C_A	-0.03	0.91	0.85	-0.03	0.91	0.86
CR_07080W_A	0.17	0.22	0.11	0	0.99	0.98
C2_09830C_A	0.31	0.52	0.35	0.48	0.34	0.22
CR_08260C_A	0.07	0.67	0.51	0.12	0.66	0.53
C2_07080C_A	-0.27	0.09	0.03	0.08	0.69	0.57
CR_04980C_A	-0.07	0.67	0.52	-0.13	0.65	0.52
C1_06530C_A	-0.06	0.62	0.46	0.09	0.43	0.3
CR_10610C_A	0.09	0.82	0.71	0.14	0.7	0.59
C1_09240C_A	-0.49	0.05	0.02	-1	0	0
C5_01230C_A	0.4	0	0	0.62	0	0
C3_06540C_A	0.04	0.87	0.79	0.09	0.72	0.61
CR_03470W_A	-0.54	0	0	-0.6	0	0
C3_01670W_A	-0.16	0.14	0.06	-0.27	0.01	0
C5_00650C_A	0.44	0	0	0.47	0	0
C6_02580W_A	0.51	0	0	0.48	0	0
C3_04690C_A	-0.24	0.23	0.12	-0.33	0.06	0.03
C3_02970C_A	0.01	0.94	0.89	0.3	0.01	0
C2_10620W_A	-0.05	0.57	0.4	-0.54	0	0
C1_02880C_A	-0.12	0.25	0.13	-0.21	0.03	0.01
C3_07230W_A	0.54	0	0	0.7	0	0
C5_01530C_A	-0.43	0	0	-0.32	0.04	0.02
C1_11030W_A	-0.22	0	0	-0.54	0	0
C2_04550C_A	0.19	0.13	0.05	0.42	0	0
C7_00370W_A	0.39	0.01	0	0.54	0	0
C4_05620C_A	0.01	0.97	0.93	0	1	1
C3_06570C_A	0.16	0.29	0.16	0.12	0.43	0.3
C2_10100W_A	-0.7	0	0	-0.31	0.21	0.12
CR_10210W_A	0.06	0.88	0.81	-0.25	0.62	0.49
C1_05950C_A	-0.26	0	0	-0.79	0	0
C1_00740C_A	-0.31	0	0	-0.35	0	0

C4_06100W_A	0.32	0	0	-0.31	0	0
C4_07170C_A	-0.18	0.74	0.6	0.12	0.84	0.77
C2_07180W_A	-0.09	0.83	0.72	0.04	0.93	0.88
C1_14100W_A	0.23	0.07	0.03	0.38	0	0
C2_10480W_A	0.71	0	0	0.46	0	0
C6_03490C_A	0.32	0.37	0.22	0.15	0.71	0.6
C5_02300C_A	0.62	0	0	0.76	0	0
C5_00230C_A	0.53	0.18	0.08	1.17	0	0
C2_04930C_A	-0.04	0.78	0.66	-0.1	0.43	0.3
C2_08150W_A	-0.13	0.54	0.37	-0.25	0.5	0.36
C1_02260C_A	-0.03	0.82	0.71	0.04	0.81	0.72
C2_03090C_A	-1.03	0	0	-1.47	0	0
CR_01410C_A	-0.56	0	0	-0.89	0	0
C7_01630W_A	-0.16	0.36	0.21	-0.17	0.31	0.2
C7_04080C_A	NA	NA	NA	0.39	0.36	0.23
CR_10800C_A	-0.1	0.34	0.2	-0.71	0	0
C1_09260C_A	0.3	0.05	0.02	0.34	0.01	0
C2_04150C_A	0.18	0.39	0.24	0.23	0.27	0.16
C2_08570W_A	-0.15	0.38	0.23	-0.29	0.07	0.03
C4_05640C_A	-0.15	0.4	0.24	-0.18	0.31	0.19
C2_05270W_A	-0.36	0.01	0	-0.16	0.3	0.19
C3_01330W_A	-0.48	0	0	-0.38	0.03	0.01
CR_03230W_A	0.06	0.76	0.63	0.11	0.62	0.49
CR_02300C_A	0.28	0	0	0.63	0	0
C7_02800W_A	0.03	0.82	0.71	0.46	0	0
C4_04810C_A	-0.74	0	0	-0.41	0.06	0.02
C5_04680W_A	-0.37	0	0	-0.21	0.15	0.08
C1_10810W_A	-0.24	0.65	0.5	-0.66	0.28	0.17
CR_03570C_A	-0.87	0	0	-1.38	0	0
C1_13950C_A	-0.27	0.52	0.35	-0.21	0.64	0.51
C5_02130W_A	-0.26	0.61	0.45	-0.1	0.87	0.8
C5_02000C_A	0.25	0.01	0	0.33	0	0
C1_13880C_A	-0.17	0.35	0.21	-0.12	0.53	0.39
C1_13360C_A	-0.94	0.02	0.01	-0.12	0.82	0.75
CR_05430W_A	0.14	0.78	0.65	0.52	0.21	0.12
C2_02780C_A	-0.01	0.99	0.97	-0.05	0.87	0.81
C2_07950W_A	-0.03	0.86	0.76	-0.07	0.68	0.56
C4_06280C_A	-0.19	0.53	0.36	-0.15	0.64	0.51
CR_03390C_A	0.45	0	0	0.44	0	0
C2_08700C_A	-0.68	0	0	-0.69	0	0
CR_07460C_A	-0.61	0	0	-0.92	0	0
C6_04050W_A	-0.23	0.66	0.5	-0.12	0.83	0.76
C1_10490W_A	0.21	0.01	0	0.22	0.01	0

CR_00370W_A	-0.04	0.83	0.72	0	0.98	0.97
C2_10720C_A	0.66	0	0	0.9	0	0
C2_04120C_A	-1.35	0	0	-1.24	0	0
C4_01940W_A	0.09	0.87	0.78	-0.26	0.64	0.51
C3_01820W_A	0.32	0.07	0.03	0.05	0.82	0.74
C1_03120W_A	0.16	0.2	0.1	-0.15	0.3	0.19
C4_07020C_A	0.1	0.85	0.75	0.37	0.59	0.45
C3_07100C_A	-0.41	0.16	0.08	-0.22	0.49	0.36
CR_01380W_A	0.2	0.25	0.13	0.01	0.96	0.92
C3_05090C_A	-0.18	0.18	0.09	-0.04	0.82	0.74
C5_01770C_A	-0.11	0.24	0.12	-0.1	0.36	0.24
C1_11020W_A	-0.38	0.06	0.02	-0.23	0.29	0.18
C4_02390W_A	0.1	0.23	0.12	-0.84	0	0
C5_00320W_A	-0.21	0.11	0.04	-0.49	0	0
C1_10170W_A	NA	NA	NA	0.26	0.49	0.35
C7_03160W_A	0.21	0.05	0.02	0.24	0.03	0.01
C2_08680W_A	-0.17	0.31	0.17	-0.18	0.32	0.2
C3_06420C_A	-0.29	0.04	0.01	-0.04	0.82	0.74
C3_04160W_A	0.07	0.87	0.79	0.18	0.64	0.51
C1_09450C_A	0.1	0.46	0.3	-0.08	0.57	0.43
C1_14230C_A	-0.1	0.5	0.33	-0.38	0	0
C1_02510W_A	0.13	0.59	0.43	0.46	0.01	0
C3_04620C_A	-0.53	0	0	-0.56	0	0
C6_02360W_A	-0.69	0.01	0	-0.93	0	0
C7_01400C_A	-0.4	0.05	0.02	-0.23	0.32	0.2
CR_01780W_A	-0.26	0.35	0.21	-0.36	0.18	0.1
C1_10230C_A	0.18	0.21	0.1	0.2	0.18	0.1
C1_10350C_A	-0.33	0	0	-0.09	0.52	0.38
CR_07870W_A	0.17	0.38	0.23	0.08	0.73	0.62
C4_04150C_A	0.42	0	0	0.39	0	0
C2_07240C_A	0.48	0.07	0.02	0.73	0	0
CR_05010W_A	-0.04	0.85	0.75	0.29	0.13	0.07
C4_00480W_A	-0.23	0.4	0.25	-0.21	0.47	0.34
C5_02220C_A	0.32	0.24	0.13	0.75	0	0
C1_06270W_A	0.38	0.03	0.01	0.37	0.04	0.02
C1_05240C_A	0.23	0	0	0.4	0	0
CR_00390W_A	0.03	0.92	0.86	0.25	0.24	0.14
CR_05120W_A	-0.49	0	0	-0.46	0	0
C1_04500W_A	0.62	0.16	0.08	1.19	0.01	0
C1_05600W_A	0.01	0.98	0.96	-0.11	0.62	0.48
C3_04110C_A	-0.17	0.1	0.04	-0.23	0.04	0.02
BGI_novel_G000(	-1.49	0	0	-1.66	0	0
C4_02980W_A	0.04	0.67	0.51	-0.18	0.05	0.02

C3_00400C_A	-0.02	0.95	0.91	0.24	0.23	0.13
C1_04560W_A	-0.14	0.45	0.29	-0.21	0.24	0.14
C1_07190W_A	0.11	0.34	0.2	0.16	0.17	0.09
CR_04920W_A	0.08	0.69	0.55	0.22	0.17	0.09
C6_02940C_A	-0.28	0.02	0.01	-0.21	0.1	0.05
C6_01160W_A	0.42	0.2	0.1	1.08	0	0
C2_08130W_A	-0.64	0	0	-0.12	0.47	0.33
C4_01200C_A	-0.61	0	0	-1.4	0	0
C1_10620W_A	-0.62	0	0	-1.03	0	0
C6_00650C_A	-0.05	0.68	0.52	-0.57	0	0
C5_00890C_A	-0.13	0.61	0.45	-0.48	0.03	0.01
C6_04500C_A	0.04	0.74	0.61	0.05	0.68	0.55
CR_01080W_A	0.54	0	0	0.67	0	0
C3_02880W_A	0.19	0.72	0.57	0.12	0.87	0.81
C2_02700C_A	-0.08	0.7	0.56	-0.3	0.1	0.05
C2_09430W_A	0.02	0.84	0.74	-0.51	0	0
C2_10160W_A	0.21	0.37	0.22	1.5	0	0
C3_05970C_A	0.15	0.17	0.08	0.14	0.19	0.1
C4_05050C_A	-0.15	0.55	0.39	-0.09	0.71	0.59
C6_02050W_A	-0.21	0.4	0.24	-0.09	0.72	0.6
C1_11750W_A	0.47	0.01	0	0.51	0	0
CR_10690W_A	-0.6	0.02	0	-0.36	0.17	0.09
C3_07130W_A	-0.23	0.11	0.05	-0.59	0	0
C5_00100C_A	-0.45	0	0	-0.13	0.33	0.21
C3_05800W_A	-0.17	0.32	0.18	-0.11	0.57	0.43
C5_04160W_A	0.06	0.84	0.74	-0.29	0.29	0.18
C7_00010C_A	0.29	0.19	0.09	0.47	0.02	0.01
C1_11010C_A	0.21	0.2	0.1	-0.21	0.18	0.1
C6_01890C_A	-0.61	0	0	-0.47	0	0
C5_03840W_A	0.24	0.27	0.15	0.11	0.66	0.54
C1_03210C_A	0.41	0	0	0.77	0	0
C2_06430C_A	0	1	1	0.39	0.11	0.05
C5_04230W_A	0	1	0.99	0.11	0.82	0.74
C4_00850C_A	-0.05	0.94	0.88	0.03	0.96	0.93
CR_09390C_A	-0.07	0.87	0.79	0.24	0.72	0.61
C3_04540C_A	-0.13	0.61	0.45	0.21	0.31	0.2
C3_01400W_A	0.03	0.88	0.8	0.3	0.02	0.01
C2_05450C_A	0.42	0.17	0.08	-0.12	0.76	0.66
C6_03210C_A	-0.45	0.13	0.06	-0.43	0.16	0.09
CR_04020C_A	-0.09	0.68	0.53	0.07	0.73	0.62
C1_07690C_A	0.06	0.62	0.45	-0.05	0.7	0.59
C3_05540C_A	-0.82	0.06	0.02	-0.94	0.09	0.04
C6_03720W_A	0.28	0	0	0.04	0.71	0.59

C2_09040W_A	-0.03	0.84	0.74	0.06	0.68	0.55
C2_03640W_A	0.19	0.43	0.27	0.64	0	0
C4_02370C_A	-0.19	0.04	0.01	-1.92	0	0
C3_02230C_A	-0.52	0	0	-0.39	0.02	0.01
C6_04360C_A	0.04	0.79	0.67	0.07	0.61	0.47
C2_09890W_A	0.08	0.82	0.72	0.6	0.02	0.01
C1_03550C_A	-0.13	0.17	0.08	-0.21	0.03	0.01
C6_02460C_A	-0.17	0.16	0.07	-0.28	0.02	0.01
C5_05130C_A	0.04	0.86	0.77	0.24	0.15	0.08
C1_09740C_A	-0.5	0.27	0.14	0.29	0.67	0.55
C7_01190W_A	0.24	0.19	0.09	0.19	0.32	0.2
CR_06590C_A	-0.74	0.03	0.01	-0.81	0.03	0.01
C5_05020C_A	0.93	0	0	0.76	0	0
C5_01650C_A	-0.38	0.28	0.15	0.06	0.92	0.88
CR_09480W_A	-0.3	0.1	0.04	0.07	0.78	0.68
C4_04270W_A	0.19	0.46	0.3	0.38	0.1	0.05
C6_02470W_A	-0.15	0.16	0.07	-0.15	0.33	0.21
C5_02530W_A	0.03	0.82	0.71	0.15	0.2	0.12
C7_00400W_A	0.37	0	0	0.37	0	0
C1_09540W_A	-0.17	0.53	0.37	0.24	0.31	0.2
CR_01060W_A	-0.55	0	0	-0.67	0	0
C6_00830C_A	0.5	0	0	0.1	0.45	0.31
C4_02540W_A	-0.18	0.42	0.26	-0.23	0.29	0.18
C5_02620C_A	-0.16	0.33	0.19	0.07	0.67	0.54
C1_13220C_A	0.65	0	0	0.97	0	0
C2_08500W_A	0.04	0.73	0.59	0.07	0.55	0.42
C1_03630W_A	-0.14	0.38	0.23	-0.04	0.83	0.76
CR_06730W_A	0.18	0.46	0.3	0.15	0.48	0.34
C6_03060W_A	0.13	0.82	0.71	0.15	0.83	0.76
CR_05730C_A	-0.22	0.16	0.07	-0.35	0.01	0
C1_07520C_A	0.09	0.59	0.42	-0.24	0.15	0.08
C3_07540C_A	-0.23	0.11	0.05	-0.28	0.06	0.03
C2_00320W_A	0.19	0.17	0.08	-0.14	0.37	0.25
C2_10290W_A	0.21	0	0	0.08	0.42	0.29
CR_03910C_A	0	0.98	0.96	0.15	0.35	0.23
C1_10680C_A	-0.26	0	0	-0.71	0	0
CR_08050C_A	0.27	0.01	0	0.51	0	0
C2_04040C_A	-0.09	0.64	0.48	-0.17	0.32	0.2
C6_01750C_A	0.55	0	0	0.35	0.04	0.02
CR_08190W_A	0.08	0.75	0.62	0.29	0.23	0.13
C1_12060C_A	0.71	0	0	0.92	0	0
C1_10970W_A	-0.77	0	0	-0.53	0.01	0
C1_05830W_A	-0.07	0.68	0.53	-0.13	0.65	0.52

C1_05740C_A	-0.63	0.01	0	-0.07	0.79	0.7
CR_10250C_A	0.02	0.86	0.78	0.16	0.07	0.03
CR_09140C_A	-1.38	0	0	-1.5	0	0
CR_03460W_A	-0.45	0	0	-0.37	0	0
C3_03750C_A	0.03	0.83	0.72	0.01	0.96	0.94
C2_07280W_A	0.2	0.59	0.43	0.25	0.49	0.35
C4_02150C_A	0.35	0.22	0.11	0.4	0.18	0.1
CR_06510W_A	-1.03	0	0	-0.67	0.1	0.05
C5_04130C_A	-0.36	0	0	-1.77	0	0
C4_06570C_A	0.17	0	0	-0.08	0.39	0.26
C1_01330C_A	-0.46	0	0	-0.28	0.05	0.02
CR_03970C_A	0.89	0	0	0.92	0	0
C2_01640W_A	-0.03	0.79	0.67	-0.08	0.44	0.3
C1_11650W_A	-0.02	0.87	0.79	-0.16	0.16	0.08
CR_07040W_A	-0.23	0.21	0.1	0.06	0.78	0.68
C4_02590C_A	-0.11	0.59	0.43	-0.03	0.91	0.86
C4_01980C_A	0.1	0.47	0.3	0.54	0	0
C4_05550C_A	-0.02	0.91	0.84	-0.12	0.27	0.17
C3_01630W_A	0.26	0	0	0.12	0.27	0.17
C4_02840C_A	0.02	0.94	0.89	-0.03	0.93	0.88
C1_08020W_A	0.3	0	0	0.37	0	0
C2_10360C_A	-0.03	0.93	0.88	-0.08	0.75	0.65
C7_04180W_A	0.06	0.8	0.69	0.19	0.37	0.25
CR_07010W_A	-0.44	0	0	-1.11	0	0
C1_08230C_A	-0.33	0.01	0	-0.84	0	0
C4_05160C_A	-0.37	0.08	0.03	0	1	1
C7_02840C_A	-1.07	0	0	-3.01	0	0
CR_09530C_A	0.85	0	0	1.14	0	0
C1_01460W_A	0.13	0.45	0.29	0.37	0.01	0
CR_06550C_A	2.79	0	0	4.44	0	0
C3_07310C_A	-0.3	0	0	-0.34	0	0
C3_05180W_A	0.37	0.12	0.05	0.43	0.07	0.03
C1_02070W_A	-0.06	0.88	0.8	-0.38	0.36	0.24
C1_03660W_A	-0.17	0.7	0.55	0.09	0.83	0.75
C4_06780C_A	0.02	0.89	0.81	0.12	0.35	0.23
C4_00940W_A	-0.49	0.16	0.08	-0.07	0.86	0.79
C5_03490C_A	0.24	0	0	0.72	0	0
C1_09710C_A	-0.52	0	0	-0.55	0	0
C7_00480W_A	-0.12	0.69	0.55	-0.23	0.64	0.51
C6_03570W_A	-0.18	0.72	0.58	-0.39	0.56	0.43
C1_01210W_A	0.45	0	0	0.55	0	0
C1_12220W_A	-0.56	0	0	-0.39	0.01	0
C5_00180W_A	-0.43	0	0	-0.83	0	0

C4_04120C_A	-0.21	0.28	0.15	-0.04	0.87	0.8
C2_04220C_A	0.02	0.89	0.81	-0.17	0.08	0.04
C2_06980W_A	-0.07	0.67	0.52	0.11	0.82	0.74
C4_02610C_A	-0.1	0.59	0.43	-0.61	0	0
C4_02290W_A	-0.32	0	0	-0.32	0	0
CR_00710C_A	-0.1	0.77	0.64	0.18	0.54	0.4
CR_02770C_A	0.11	0.38	0.23	-0.12	0.32	0.2
CR_02890C_A	-0.11	0.58	0.42	-0.18	0.33	0.21
C2_07590W_A	0.28	0	0	0.47	0	0
C1_02780W_A	-0.03	0.81	0.7	0.04	0.75	0.65
C4_01430C_A	-0.51	0.05	0.02	0.07	0.91	0.86
CR_07690W_A	-0.29	0.37	0.22	0.09	0.81	0.72
C2_02330W_A	0.7	0.1	0.04	0.7	0.18	0.1
C3_04400C_A	0.06	0.65	0.5	0.05	0.69	0.57
C4_03850W_A	-0.42	0	0	-0.32	0.01	0
C6_03740W_A	0.12	0.81	0.7	0.44	0.33	0.21
C5_00830C_A	0.93	0	0	0.37	0	0
C7_03800W_A	0.11	0.44	0.28	0.36	0	0
C6_03030W_A	-0.16	0.72	0.58	-0.3	0.44	0.31
C6_03810W_A	0.02	0.95	0.92	0.09	0.76	0.66
C3_01980C_A	-0.18	0.52	0.35	-0.08	0.78	0.68
C7_03660C_A	0.26	0	0	-0.01	0.96	0.93
C3_05850W_A	-0.29	0.09	0.04	-0.05	0.81	0.72
C1_10940C_A	0.41	0	0	0.31	0	0
C3_04840C_A	0.14	0.63	0.47	0.59	0	0
C4_06530C_A	0.13	0.8	0.68	0.02	0.98	0.96
C1_09090C_A	-0.22	0.2	0.09	-0.06	0.77	0.67
C1_03730C_A	0.55	0	0	0.16	0.11	0.05
C4_03430W_A	0.21	0.58	0.41	0.55	0.09	0.04
CR_06440C_A	-0.18	0.27	0.15	0.27	0.06	0.03
C4_03020W_A	-0.05	0.71	0.56	0	1	0.99
C4_07120C_A	-0.18	0.12	0.05	-0.32	0.01	0
C2_00970C_A	-0.02	0.89	0.81	-0.45	0	0
C6_01310W_A	-0.04	0.94	0.89	0.52	0.43	0.29
C3_05600W_A	0.32	0.37	0.22	-0.52	0.2	0.11
C1_02490C_A	-0.2	0.19	0.09	-0.26	0.1	0.05
C4_01780C_A	-0.45	0.01	0	-0.02	0.94	0.9
C6_00550W_A	-0.24	0.04	0.02	-0.02	0.9	0.84
C1_11500C_A	-0.24	0.16	0.07	-0.17	0.37	0.24
C1_03470C_A	0.19	0.72	0.57	-0.03	0.97	0.94
C1_11850W_A	0.53	0	0	-0.35	0	0
C7_03640C_A	-0.15	0.32	0.18	-0.14	0.35	0.23
CR_00600C_A	-0.36	0.01	0	-0.42	0	0

C2_06970W_A	-0.07	0.7	0.55	-0.47	0	0
CR_08570W_A	-0.07	0.51	0.34	-0.16	0.11	0.06
C4_00090W_A	0.12	0.45	0.29	0.07	0.73	0.62
C1_10750C_A	0.18	0.3	0.17	0.22	0.18	0.1
CR_04310C_A	-0.15	0.48	0.31	0.1	0.63	0.5
C1_07270W_A	0.56	0	0	0.58	0	0
C1_06640C_A	0.07	0.9	0.83	0.19	0.76	0.66
C5_04870W_A	0.16	0.29	0.16	0.12	0.47	0.34
C4_01320C_A	0.3	0.17	0.08	0.4	0.04	0.02
C2_10730W_A	1.61	0	0	2.43	0	0
C6_01790C_A	0.16	0.39	0.23	0.01	0.97	0.94
C2_08860W_A	0.17	0.04	0.02	0.27	0	0
C6_04240W_A	-0.56	0.09	0.04	-0.2	0.61	0.48
C1_08470W_A	-0.11	0.4	0.25	-0.35	0	0
C1_11970C_A	-0.13	0.56	0.4	-0.17	0.46	0.32
C1_12320C_A	0.16	0.66	0.51	0.31	0.35	0.22
CR_01320C_A	-0.67	0	0	-1.29	0	0
C5_04730C_A	0.5	0	0	0.43	0	0
C4_07250C_A	-0.06	0.56	0.4	0	0.99	0.97
C3_07500W_A	0.05	0.71	0.57	-0.21	0.06	0.03
C1_10200C_A	-0.09	0.62	0.46	-0.04	0.81	0.73
C1_00160C_A	-0.4	0	0	-1.48	0	0
C2_00740C_A	-0.23	0.02	0.01	-0.33	0	0
C1_05860W_A	-0.14	0.61	0.44	-0.22	0.39	0.26
C1_14470W_A	0.01	0.97	0.95	0.01	0.92	0.88
C3_07340W_A	0.36	0	0	0.86	0	0
C4_03470C_A	0.23	0.66	0.5	-1.14	0.03	0.01
C1_10250C_A	-0.31	0.33	0.19	-0.22	0.52	0.39
C1_06420C_A	-0.27	0.08	0.03	0.11	0.53	0.4
CR_01470W_A	-0.8	0	0	-0.11	0.69	0.57
C2_02940W_A	-1.44	0	0	-2.14	0	0
C6_00750C_A	-0.02	0.81	0.7	-0.18	0.03	0.01
C3_07010W_A	-0.08	0.52	0.35	-0.21	0.06	0.03
C4_06420W_A	-0.15	0.23	0.12	0.01	0.97	0.95
CR_09840C_A	-0.27	0.19	0.09	-0.12	0.61	0.47
CR_06840W_A	-0.21	0.22	0.11	-0.2	0.26	0.16
CR_00970W_A	-0.36	0.31	0.17	-0.12	0.87	0.81
C1_04980C_A	-0.17	0.15	0.07	-0.29	0.02	0.01
C1_10090C_A	0.01	0.93	0.87	0.05	0.69	0.58
C2_09560C_A	0.12	0.4	0.24	0.23	0.1	0.05
C6_01970C_A	-0.08	0.31	0.17	-0.52	0	0
C4_02320C_A	0.38	0	0	0.76	0	0
CR_00900W_A	-0.52	0.02	0.01	-0.56	0.02	0.01

C3_02470C_A	-0.22	0	0	-0.62	0	0
C3_06980W_A	0.8	0	0	1.24	0	0
C1_05100W_A	0.32	0.1	0.04	0.36	0.06	0.03
C1_14260C_A	-0.02	0.9	0.82	-0.26	0	0
C1_11270W_A	0.73	0	0	2.09	0	0
C4_06790W_A	0.05	0.79	0.67	0.17	0.28	0.17
C4_06190C_A	-0.25	0.05	0.02	-0.67	0	0
C3_00480C_A	0.49	0	0	1.26	0	0
C7_03610C_A	-0.08	0.62	0.45	-0.05	0.79	0.7
C4_02520C_A	-0.2	0.39	0.24	-0.28	0.23	0.13
C1_09910C_A	0.06	0.85	0.76	0.33	0.16	0.09
C3_05880C_A	-0.06	0.61	0.45	0.01	0.92	0.87
C3_02090C_A	0.24	0.04	0.01	0.44	0	0
CR_05190W_A	-0.23	0.64	0.48	-0.28	0.69	0.57
C1_09100W_A	-0.15	0.79	0.68	-0.2	0.74	0.63
C7_00930W_A	0.37	0	0	0.39	0	0
C2_05770W_A	1.02	0	0	1.72	0	0
C3_03880C_A	0.14	0.29	0.16	0.19	0.14	0.07
CR_08960C_A	-0.29	0.05	0.02	-0.32	0.03	0.01
CR_06110C_A	0.16	0.47	0.31	1	0	0
C1_09970C_A	-0.04	0.84	0.74	0.17	0.35	0.23
C4_05220C_A	-0.31	0.07	0.03	-0.28	0.12	0.06
CR_01570W_A	-0.26	0.24	0.12	-0.1	0.69	0.57
CR_03240C_A	-0.15	0.54	0.37	0.17	0.51	0.37
C7_03650W_A	0.1	0.6	0.44	0.19	0.23	0.14
C3_05960W_A	-0.61	0	0	-1.1	0	0
C7_02110W_A	0.02	0.9	0.82	0.16	0.3	0.19
C2_02420C_A	-0.17	0.13	0.06	-0.13	0.3	0.19
C2_00890W_A	-0.1	0.87	0.78	0.17	0.81	0.72
CR_09210W_A	-0.43	0.02	0.01	-0.32	0.1	0.05
C2_06480W_A	-0.51	0	0	-0.39	0.01	0
CR_02760C_A	-0.42	0	0	-0.23	0.22	0.13
C2_02600C_A	0.53	0.18	0.08	1.07	0	0
C1_07380C_A	-0.29	0.29	0.16	-0.14	0.63	0.49
C1_11640C_A	-0.05	0.8	0.68	-0.39	0.01	0
C6_03730C_A	-0.01	0.92	0.86	-0.34	0	0
C2_06470W_A	2.39	0	0	2.95	0	0
C1_14450C_A	0.37	0	0	0.34	0.01	0
C1_06260W_A	-0.06	0.87	0.79	-0.11	0.84	0.77
CR_10100C_A	0.52	0.23	0.12	1.37	0.01	0.01
C4_00670W_A	0.09	0.64	0.48	0.08	0.65	0.53
C2_02510W_A	-0.24	0.11	0.05	-0.64	0	0
CR_05940W_A	0.21	0.65	0.49	0.64	0.1	0.05

C1_11910W_A	-0.14	0.57	0.41	-0.18	0.44	0.3
C3_01300C_A	-0.01	0.98	0.95	0.39	0.15	0.08
C1_11570W_A	-0.25	0.58	0.41	-0.3	0.54	0.4
C3_05730C_A	0.09	0.67	0.52	0.72	0	0
C1_00950C_A	-0.7	0	0	-0.84	0	0
C3_07580W_A	-0.1	0.77	0.64	-0.03	0.93	0.89
C1_00420W_A	0.42	0	0	0.56	0	0
C1_12780W_A	0.29	0.01	0	0.72	0	0
C2_06990W_A	-0.11	0.83	0.73	-0.12	0.84	0.76
C3_00990C_A	-0.11	0.85	0.75	0.39	0.52	0.38
C2_02570W_A	0.53	0.03	0.01	0.63	0.01	0
C2_09770C_A	0.27	0.08	0.03	0.65	0	0
CR_03310C_A	-0.28	0.01	0	-0.18	0.16	0.09
CR_07820W_A	0.19	0.7	0.55	0.01	0.99	0.97
CR_04850C_A	-0.36	0.27	0.14	-0.7	0.03	0.01
C6_02590C_A	-0.37	0	0	-0.46	0	0
C3_03440C_A	-0.12	0.52	0.35	-0.59	0	0
CR_02100C_A	-0.23	0.13	0.06	0	0.99	0.98
C5_04340W_A	-0.14	0.27	0.14	-0.1	0.47	0.33
C3_04100W_A	-0.24	0.63	0.47	0.53	0.21	0.12
C1_05470W_A	-0.31	0.02	0.01	-0.44	0	0
C2_06550W_A	0.16	0.53	0.37	0.14	0.59	0.46
C2_08460C_A	0.02	0.96	0.92	0.36	0.27	0.17
C1_07650W_A	0.6	0.16	0.08	-0.46	0.45	0.31
C2_00490W_A	0.18	0.2	0.1	0.19	0.2	0.11
C1_05930C_A	-0.21	0.33	0.19	-0.28	0.11	0.06
C3_00850C_A	-0.49	0	0	-0.59	0	0
CR_03740C_A	0.06	0.76	0.63	0.26	0.07	0.03
C3_01470W_A	-0.24	0.25	0.13	0.03	0.9	0.84
C5_01970C_A	-0.15	0.3	0.17	0.15	0.3	0.18
C3_02560W_A	0.42	0.03	0.01	0.47	0.02	0.01
C2_09050C_A	0.41	0.01	0	0.55	0	0
C3_05590C_A	0.39	0	0	0.28	0.01	0
C3_00090W_A	-0.14	0.04	0.01	-0.56	0	0
CR_01490C_A	0.59	0	0	0.87	0	0
C7_01350C_A	-0.23	0.28	0.15	-0.13	0.56	0.43
C6_02970C_A	-0.2	0.53	0.36	-0.44	0.15	0.08
C2_05670C_A	-0.08	0.79	0.67	0.09	0.73	0.62
C5_00110C_A	0.69	0	0	0.56	0	0
C6_03410C_A	-0.03	0.78	0.66	0.11	0.28	0.17
C4_02410C_A	-0.62	0	0	-0.52	0	0
C1_04260W_A	0.01	0.92	0.85	-0.64	0	0
C4_07030W_A	-0.7	0	0	-0.84	0	0

C2_10350C_A	0.28	0	0	0.04	0.74	0.63
CR_09220C_A	0.33	0.46	0.3	0.32	0.53	0.39
C2_08970C_A	0.72	0	0	1.08	0	0
CR_05520W_A	-1.07	0	0	-1.58	0	0
C3_06750W_A	0.23	0.26	0.13	0.61	0	0
C2_07680W_A	0.05	0.66	0.5	-0.16	0.15	0.08
C1_09400C_A	0.42	0.2	0.1	1.13	0	0
C1_02950W_A	0.27	0.62	0.45	0.61	0.27	0.16
C1_01490W_A	-0.44	0	0	-0.67	0	0
CR_10040W_A	-0.14	0.48	0.32	-0.17	0.43	0.29
C2_04490W_A	-0.05	0.68	0.53	-0.03	0.84	0.77
C1_07910C_A	-0.15	0.24	0.12	-0.33	0	0
CR_02420W_A	-0.58	0	0	-0.37	0.04	0.02
C5_01340W_A	-0.38	0.01	0	-0.28	0.04	0.01
C4_06030W_A	0.38	0	0	0.44	0	0
C4_03230C_A	0.19	0.21	0.1	-0.09	0.61	0.47
C5_00470C_A	0.4	0.42	0.26	-0.01	0.99	0.98
C3_05780C_A	1.92	0	0	2.15	0	0
C1_07920W_A	-0.02	0.98	0.96	0.09	0.91	0.86
C5_01700W_A	-0.72	0	0	-0.7	0	0
C1_11810W_A	0.12	0.5	0.33	0.14	0.42	0.29
C1_04130W_A	-0.67	0	0	-1.04	0	0
C1_08750W_A	-0.49	0	0	-0.77	0	0
C2_03500W_A	-0.36	0	0	-0.2	0.14	0.07
C1_10360C_A	-1.52	0	0	-2.55	0	0
C1_11990W_A	-0.45	0	0	-1.12	0	0
C5_03800W_A	-0.64	0	0	-0.41	0	0
C2_10220C_A	-0.44	0.01	0	-0.17	0.41	0.28
C1_00210C_A	0.07	0.38	0.23	0.03	0.76	0.65
C1_13250W_A	0.54	0	0	0.42	0.04	0.02
C2_03210W_A	-0.04	0.91	0.84	0.14	0.6	0.47
C4_04260C_A	-0.1	0.29	0.16	-0.22	0.02	0.01
C3_06800C_A	-0.28	0.04	0.01	-0.09	0.6	0.47
C1_06210W_A	-0.7	0	0	-0.73	0	0
C1_07990C_A	-0.64	0	0	0.2	0.24	0.14
C6_03400C_A	0.07	0.67	0.51	0.38	0	0
C1_12660W_A	-0.48	0.02	0.01	-0.27	0.29	0.18
C1_08900W_A	-0.43	0	0	-1.28	0	0
C4_06230C_A	-0.03	0.85	0.75	-0.1	0.48	0.34
C4_03310C_A	-0.07	0.68	0.53	-0.13	0.65	0.52
C6_03230W_A	0.3	0.29	0.16	0.53	0.04	0.02
C7_02040C_A	0.71	0	0	0.97	0	0
C6_02900C_A	-0.36	0.15	0.07	-0.35	0.17	0.09

C4_00730C_A	0.28	0.55	0.38	-0.1	0.87	0.81
C2_01050W_A	-0.01	0.99	0.98	0.22	0.75	0.65
C5_01890W_A	0.42	0	0	0.55	0	0
CR_03860C_A	-0.02	0.94	0.9	0.15	0.43	0.3
C7_02690C_A	0.06	0.8	0.69	-0.23	0.27	0.16
C5_04970C_A	-0.24	0.51	0.35	-0.7	0.04	0.02
C2_05520W_A	-0.41	0.01	0	-0.22	0.21	0.12
C1_00120C_A	0.23	0.08	0.03	0.03	0.84	0.77
C6_01510W_A	0.77	0	0	0.32	0.02	0.01
C4_00320C_A	0.27	0.13	0.06	0.31	0.08	0.04
C1_02410C_A	-0.03	0.82	0.72	0	0.99	0.99
C5_02390C_A	0.17	0.18	0.08	0.24	0.06	0.03
CR_01520W_A	-0.07	0.59	0.43	-0.13	0.3	0.19
C7_03480W_A	0.11	0.57	0.41	0.03	0.91	0.85
CR_00510C_A	-0.36	0	0	-0.77	0	0
CR_10410C_A	-0.38	0.01	0	-0.37	0.01	0
C6_04350C_A	0.36	0	0	-0.2	0.14	0.07
C3_05200W_A	-0.03	0.72	0.57	-0.48	0	0
CR_01630C_A	-0.75	0.02	0.01	-0.86	0.02	0.01
CR_03650W_A	-0.79	0	0	-0.73	0	0
C6_01120C_A	-0.58	0.04	0.01	-0.52	0.09	0.04
C5_04780W_A	-0.07	0.68	0.53	0.04	0.84	0.77
CR_06230W_A	-0.6	0	0	-0.37	0.04	0.02
CR_00800C_A	-0.44	0	0	-0.63	0	0
C5_03910C_A	-0.37	0.29	0.16	-0.3	0.42	0.29
CR_06580W_A	NA	NA	NA	NA	NA	NA
CR_08700C_A	0.22	0.04	0.01	0.24	0.04	0.02
C2_10760C_A	0.14	0.19	0.09	0.08	0.54	0.4
C1_06470W_A	-0.13	0.73	0.59	-0.32	0.35	0.23
C1_03760C_A	-0.18	0.12	0.05	-0.33	0.01	0
C2_06350C_A	0.01	0.99	0.98	0.19	0.77	0.67
C1_07180W_A	0.35	0	0	0.33	0	0
C1_14120C_A	-0.78	0	0	-1.34	0	0
C5_05190W_A	0.44	0.07	0.03	0.43	0.09	0.04
C1_07560W_A	-0.01	0.97	0.94	0.07	0.81	0.72
CR_00580W_A	-0.1	0.51	0.35	-0.31	0.01	0
CR_02560C_A	-0.04	0.83	0.73	0.03	0.9	0.85
CR_07230W_A	-0.23	0.04	0.01	-0.29	0.01	0
CR_01020C_A	0	1	1	0.59	0.01	0
C7_03540C_A	-1.04	0	0	-1.3	0	0
C7_02770W_A	-0.87	0	0	-0.85	0	0
C7_03870W_A	0	1	1	0.14	0.32	0.2
C6_01050W_A	-0.01	0.98	0.95	-0.17	0.22	0.12

C4_03080W_A	-0.04	0.88	0.81	0.3	0.39	0.26
C3_01210C_A	0.06	0.64	0.48	0.18	0.17	0.09
CR_05760C_A	0.16	0.34	0.2	0.18	0.28	0.17
C1_06510C_A	-0.22	0.16	0.07	-0.55	0	0
CR_07660C_A	-0.36	0	0	-0.36	0	0
CR_02810W_A	-0.11	0.45	0.29	-0.31	0.01	0
CR_02740W_A	0.3	0.01	0	0.44	0	0
C1_04190C_A	-0.18	0.3	0.16	-0.17	0.28	0.17
C4_04080C_A	-0.2	0.64	0.48	-0.43	0.47	0.34
C5_04410C_A	0.42	0.01	0	0.67	0	0
CR_02180W_A	0.36	0.02	0.01	0.09	0.65	0.52
C5_03740W_A	0.13	0.57	0.4	0.01	0.96	0.93
C6_01920C_A	0.05	0.76	0.62	0.14	0.33	0.21
C1_03880C_A	NA	NA	NA	NA	NA	NA
C3_04830C_A	0.25	0	0	0.22	0	0
C5_01780W_A	-0.14	0.35	0.2	-0.24	0.08	0.04
C5_00280C_A	-0.06	0.68	0.53	-0.38	0	0
C1_06340W_A	-0.37	0.32	0.18	-0.52	0.17	0.09
C1_01240W_A	0.29	0.36	0.21	0.6	0.03	0.01
C1_00140W_A	0.27	0	0	0.21	0.02	0.01
C5_02590C_A	-1.66	0	0	-1.31	0	0
C1_05160C_A	0.31	0.02	0	0.21	0.16	0.09
C6_00390W_A	0.25	0	0	0.21	0.04	0.02
CR_01040C_A	0.45	0.03	0.01	0.16	0.57	0.44
C2_04050C_A	0.07	0.57	0.41	-0.29	0	0
CR_02260C_A	0.01	0.96	0.93	-0.4	0.02	0.01
C4_06060W_A	0.07	0.62	0.46	-0.05	0.71	0.6
CR_02490W_A	0.67	0	0	-0.88	0	0
CR_07520C_A	0.42	0	0	0.81	0	0
C3_02130W_A	-0.88	0	0	-0.88	0	0
CR_10270C_A	0.22	0.02	0	0.22	0.03	0.01
C2_01600C_A	-0.08	0.5	0.33	0.08	0.48	0.34
CR_03670W_A	0.24	0.66	0.5	-0.19	0.79	0.7
CR_04050C_A	-0.21	0.03	0.01	-0.15	0.15	0.08
C2_08710W_A	-0.24	0.44	0.27	0.1	0.79	0.69
CR_06000W_A	0.06	0.67	0.52	0.17	0.2	0.11
C3_06450W_A	0.01	0.99	0.98	1.13	0.03	0.01
C1_07420C_A	-0.03	0.89	0.82	0.08	0.72	0.6
C1_03360W_A	0.19	0.06	0.02	0.07	0.61	0.47
C3_02010C_A	0.12	0.84	0.74	0.14	0.82	0.74
C5_04990W_A	-0.66	0	0	-0.82	0	0
C4_04000W_A	-0.46	0	0	-0.79	0	0
C2_01650W_A	1.24	0	0	2.28	0	0

C2_06840W_A	0.03	0.97	0.93	-0.37	0.51	0.37
C6_01700W_A	-0.13	0.06	0.02	-0.52	0	0
C1_00860W_A	-0.4	0.13	0.06	-0.01	0.96	0.94
C2_01360C_A	-0.06	0.66	0.5	-1.12	0	0
C5_05270C_A	0.01	0.93	0.87	-0.1	0.4	0.27
C5_03060C_A	0.06	0.87	0.78	0.55	0.35	0.23
C5_04030W_A	-1.6	0	0	-1.12	0	0
C4_01990W_A	0.11	0.86	0.77	0.21	0.76	0.66
C1_03900W_A	-0.24	0.35	0.21	0.08	0.78	0.68
C2_09870W_A	-0.29	0.03	0.01	-0.45	0	0
C2_05370C_A	-0.36	0.03	0.01	-0.36	0.04	0.02
C5_04220W_A	0.01	0.98	0.97	-0.21	0.74	0.63
C6_01430C_A	0.22	0.14	0.06	0.42	0	0
C2_03870W_A	0.17	0.29	0.16	0.24	0.1	0.05
C5_04630W_A	0.15	0.49	0.32	-0.23	0.28	0.17
C1_01060W_A	0.12	0.09	0.03	0.27	0	0
C1_04810W_A	0.17	0.13	0.06	0.29	0.01	0
CR_09620C_A	-0.27	0.16	0.07	-0.49	0.01	0
C5_00060C_A	0.66	0	0	0.81	0	0
CR_02860W_A	-0.01	0.93	0.87	0.21	0.03	0.01
C1_04030W_A	0.28	0	0	0.08	0.5	0.36
C5_00240W_A	0.43	0	0	0.63	0	0
C1_09610W_A	0.09	0.54	0.38	0.12	0.33	0.21
C1_09370W_A	-0.22	0.14	0.06	-0.41	0.01	0
C6_03310W_A	0.39	0	0	0.31	0.01	0
C5_05480W_A	0.2	0.04	0.01	-0.08	0.49	0.35
C5_03080C_A	-0.3	0.11	0.05	-0.41	0.03	0.01
C6_02770W_A	-0.84	0	0	-1.32	0	0
C3_01570W_A	-0.06	0.77	0.65	-0.05	0.78	0.68
C3_05140C_A	-0.19	0.27	0.14	-0.11	0.55	0.42
CR_06150C_A	-0.66	0	0	-1.43	0	0
C1_03430W_A	-0.23	0.14	0.06	-0.17	0.27	0.17
C4_03790W_A	0.19	0.09	0.04	0.15	0.21	0.12
C2_07530C_A	0.33	0.46	0.3	0.16	0.81	0.72
C1_13030C_A	0.07	0.47	0.31	0.11	0.22	0.13
C2_07520C_A	0.3	0.01	0	0.47	0	0
C1_10500W_A	-0.46	0	0	-0.06	0.69	0.57
CR_00840C_A	0.04	0.88	0.79	0.17	0.76	0.66
C2_03630W_A	-0.24	0.64	0.48	-0.05	0.95	0.92
C2_01110C_A	0.67	0	0	0.69	0	0
C2_10460C_A	-0.24	0.01	0	-0.24	0.02	0.01
C1_04570C_A	-0.75	0	0	-1.01	0	0
C1_08220W_A	0.1	0.35	0.2	0.13	0.22	0.13

C6_03590C_A	-0.53	0	0	-0.46	0	0
C3_04460W_A	0.32	0.01	0	0.36	0	0
C3_04090W_A	0.14	0.03	0.01	0.1	0.28	0.17
CR_05780W_A	0.26	0	0	0.22	0.01	0
C7_01010W_A	0.26	0.62	0.46	0.86	0.09	0.04
C6_01620W_A	-0.42	0.04	0.02	-0.3	0.21	0.12
C4_00500W_A	0.11	0.42	0.26	0.17	0.23	0.13
C2_05650W_A	0	0.98	0.95	0.11	0.33	0.21
C3_05330C_A	-0.79	0	0	-1.37	0	0
C2_01550W_A	0.07	0.47	0.31	-0.03	0.81	0.72
C1_14050C_A	0.17	0.3	0.17	0.13	0.42	0.28
C3_04610W_A	-0.35	0.12	0.05	-0.2	0.4	0.27
C3_07630C_A	1.02	0	0	1.45	0	0
CR_04540C_A	-0.15	0.69	0.55	0.15	0.7	0.59
C1_03380W_A	0.11	0.11	0.05	-0.09	0.29	0.18
CR_07370W_A	0.32	0.21	0.11	0.58	0.01	0
C4_02100C_A	0.62	0	0	0.02	0.93	0.88
CR_09410W_A	-0.21	0.37	0.22	-0.13	0.62	0.49
C3_03140C_A	0.59	0.01	0	-0.22	0.5	0.36
C1_10520W_A	0.15	0.66	0.5	0.3	0.33	0.21
CR_06770C_A	-0.27	0.02	0.01	-0.43	0	0
C7_03440W_A	-0.45	0	0	-1.14	0	0
C2_05910W_A	0.36	0	0	0.64	0	0
C1_14560C_A	0.13	0.55	0.38	-0.38	0.05	0.02
C3_03260W_A	0.03	0.78	0.66	0.06	0.6	0.47
C4_05420C_A	-0.06	0.79	0.67	-0.08	0.75	0.64
C6_02230W_A	-0.62	0	0	-0.41	0.01	0
C2_02000W_A	0.13	0.3	0.17	0.33	0	0
C4_03000C_A	-0.38	0.02	0.01	-1	0	0
C1_10730W_A	0.25	0.08	0.03	0.58	0	0
C3_07940W_A	0.12	0.31	0.17	0.14	0.3	0.19
C5_03630C_A	-0.86	0	0	-1.03	0	0
C2_01390W_A	0.24	0.02	0.01	0.35	0	0
C2_03590C_A	-0.41	0.01	0	-0.43	0.01	0
CR_03350C_A	-0.09	0.46	0.3	-0.2	0.08	0.04
C4_05650W_A	-0.45	0.01	0	-0.43	0.01	0
C6_03880W_A	0.07	0.79	0.67	0.47	0.01	0
C3_00720W_A	0.1	0.62	0.46	-0.79	0	0
C1_04780C_A	0.09	0.52	0.35	0.12	0.35	0.23
C1_11430W_A	-0.01	0.97	0.94	0.23	0.18	0.1
C1_09050W_A	-0.25	0.14	0.06	-0.41	0.02	0.01
C1_13110C_A	0.17	0.11	0.04	0.75	0	0
C3_07860C_A	0.12	0.42	0.26	-0.07	0.68	0.55

C2_08360C_A	0.42	0.32	0.18	0.19	0.77	0.67
C3_00570C_A	-0.8	0	0	-1.2	0	0
CR_08410W_A	-0.06	0.72	0.58	-0.06	0.73	0.62
C2_07770C_A	0.22	0.68	0.54	0.64	0.24	0.14
C2_04410W_A	0.27	0.1	0.04	0.23	0.18	0.1
C2_06020W_A	0.24	0.01	0	-0.4	0	0
CR_08270W_A	-0.2	0.67	0.51	-0.32	0.63	0.5
CR_07090W_A	0.42	0	0	0.37	0	0
C1_01840C_A	0.6	0	0	0.3	0.09	0.04
C3_07770C_A	0.73	0	0	0.92	0	0
C2_02880C_A	0.01	0.97	0.94	0.31	0	0
C4_06630C_A	NA	NA	NA	0.12	0.67	0.55
C2_06300W_A	-0.08	0.81	0.7	-0.07	0.84	0.77
C2_01980C_A	0.54	0	0	0.68	0	0
C2_01750C_A	0.63	0	0	1.62	0	0
C4_04840C_A	-0.63	0	0	-0.75	0	0
C6_02280W_A	-0.23	0.02	0	-0.36	0	0
C5_01570C_A	0.7	0.03	0.01	0.85	0.01	0
C6_01460C_A	1.19	0	0	1.51	0	0
C2_00800C_A	0.14	0.68	0.53	0.27	0.35	0.23
C3_05890W_A	-0.31	0.14	0.06	-0.07	0.78	0.68
CR_03520C_A	0.46	0	0	0.61	0	0
C2_04660C_A	-0.13	0.43	0.27	-0.08	0.72	0.61
C1_05390C_A	-0.13	0.35	0.2	0.05	0.72	0.61
C5_03290C_A	-0.35	0.03	0.01	-0.43	0.01	0
C2_10550C_A	0.03	0.8	0.68	-0.36	0	0
C3_01830C_A	0.14	0.22	0.11	0.07	0.62	0.48
C1_12810W_A	-0.19	0.31	0.17	0.03	0.9	0.85
C4_03380C_A	0.2	0.21	0.1	-0.05	0.79	0.7
C1_13590W_A	-0.65	0.15	0.07	-0.27	0.65	0.53
C3_02760C_A	-0.17	0.21	0.1	-0.31	0.02	0.01
C7_00160C_A	-0.47	0	0	-0.87	0	0
CR_04400W_A	0	1	0.99	0.11	0.82	0.74
C2_06220C_A	-0.22	0.31	0.18	-0.59	0	0
C2_06670C_A	-0.74	0	0	-0.72	0	0
C7_03180C_A	-0.06	0.57	0.4	-0.59	0	0
C5_05400W_A	0.62	0	0	0.76	0	0
C7_01870W_A	0.11	0.43	0.27	-0.05	0.78	0.68
C3_02250C_A	0	0.99	0.99	-0.01	0.99	0.97
C1_13620W_A	0.04	0.75	0.62	-0.84	0	0
C2_03770C_A	-0.35	0	0	-0.29	0.01	0
C6_00350W_A	0.13	0.21	0.1	0.23	0.03	0.01
C3_02080W_A	0.31	0	0	0.48	0	0

C6_00900C_A	-0.43	0.01	0	-0.4	0.02	0.01
C7_02240W_A	-0.21	0.69	0.55	0.16	0.79	0.7
C1_00760W_A	0.35	0	0	0.93	0	0
C3_04730C_A	0.21	0.66	0.5	-0.22	0.73	0.62
C2_08170W_A	0.15	0.77	0.65	0.14	0.8	0.71
C6_02340W_A	0.13	0.16	0.07	0.05	0.64	0.51
CR_08630W_A	0.21	0.1	0.04	0.26	0.04	0.02
C1_12190W_A	-0.22	0.24	0.12	-0.29	0.11	0.06
C7_04200C_A	0.09	0.48	0.32	0.14	0.21	0.12
C1_13100W_A	0	1	0.99	-0.13	0.65	0.52
CR_05020W_A	0.31	0.28	0.15	0.16	0.61	0.48
C5_02520W_A	0.15	0.57	0.41	1.1	0	0
C1_13350W_A	0.13	0.35	0.21	0.24	0.05	0.02
C7_01300C_A	-0.43	0.11	0.05	-0.37	0.18	0.1
C2_03820C_A	0.02	0.85	0.75	-0.59	0	0
C5_04810W_A	0.25	0	0	-0.03	0.71	0.59
C1_00650C_A	0.07	0.67	0.51	-0.01	0.97	0.94
C6_00600C_A	0.21	0.04	0.01	-0.04	0.78	0.68
C2_09310C_A	-0.22	0.35	0.2	-0.36	0.14	0.07
C6_03860C_A	-0.15	0.73	0.59	0.21	0.61	0.48
CR_03640C_A	-0.22	0.11	0.05	-0.31	0.02	0.01
C6_00190W_A	0.08	0.44	0.28	0.06	0.62	0.49
C3_03630W_A	0.35	0.12	0.05	0.41	0.06	0.03
C5_03440W_A	-0.82	0.01	0	-0.3	0.37	0.24
C6_03500C_A	0.36	0.47	0.31	1.01	0.07	0.03
C1_11920W_A	-0.15	0.37	0.22	-0.06	0.74	0.63
C1_04770C_A	-0.2	0.03	0.01	-1.24	0	0
C1_06970C_A	0.16	0.21	0.1	-0.03	0.82	0.74
CR_00180C_A	-0.32	0.08	0.03	-0.13	0.52	0.38
CR_02390W_A	-0.1	0.42	0.26	-0.19	0.08	0.04
CR_06010W_A	-0.58	0	0	-0.49	0.01	0
C3_02980C_A	0.03	0.94	0.88	-0.46	0.06	0.03
C6_02730W_A	0.43	0	0	0.95	0	0
C1_09460W_A	0.03	0.78	0.66	-0.15	0.16	0.08
C3_04420W_A	0	0.98	0.96	0.03	0.81	0.72
C2_08560W_A	-0.26	0.51	0.35	-0.63	0.06	0.03
C1_06040W_A	-0.23	0.32	0.19	0.07	0.76	0.66
CR_09340W_A	0.1	0.32	0.18	0.22	0.03	0.01
C1_08940C_A	0.13	0.31	0.17	-0.27	0.03	0.01
C1_13940W_A	0.37	0	0	0.05	0.68	0.56
C1_01650W_A	0.3	0	0	0.3	0	0
C1_10820C_A	-0.03	0.7	0.55	-0.28	0	0
C4_07010C_A	-0.6	0.01	0	-0.35	0.14	0.07

C2_09070C_A	-0.11	0.4	0.24	-0.17	0.18	0.1
C3_00210C_A	0.05	0.88	0.8	-0.1	0.79	0.69
C2_02210C_A	-0.22	0.49	0.32	0.16	0.6	0.47
C2_04140W_A	0.42	0	0	0.68	0	0
C4_00280W_A	0.51	0	0	0.55	0	0
C1_10270C_A	-0.56	0	0	-0.77	0	0
C2_10180W_A	-0.65	0.04	0.01	-0.78	0.02	0.01
C3_01170W_A	-0.31	0.09	0.04	0.33	0.08	0.04
C4_00470C_A	0.17	0.18	0.09	0.3	0.01	0
CR_10670W_A	0.53	0	0	0.56	0	0
C2_02750C_A	0.44	0.36	0.21	0.14	0.83	0.75
C6_00250W_A	-0.47	0	0	-0.99	0	0
C1_07160C_A	1.09	0	0	1.5	0	0
C7_03150W_A	0.08	0.9	0.82	0.16	0.79	0.69
C7_04250W_A	0.1	0.73	0.59	-0.05	0.88	0.82
CR_04910W_A	-0.09	0.8	0.69	0.43	0.09	0.04
C2_07940C_A	0.18	0.17	0.08	-0.12	0.38	0.26
CR_01140C_A	-0.25	0.01	0	-0.46	0	0
C2_01410C_A	-0.45	0	0	-0.59	0	0
C2_08690C_A	-0.22	0.39	0.24	0.02	0.95	0.92
CR_05030W_A	0.37	0.01	0	0.16	0.31	0.19
C3_04390W_A	0.37	0	0	0.32	0	0
CR_05130C_A	0.74	0	0	0.87	0	0
C2_07260C_A	0.16	0.19	0.09	0.01	0.97	0.95
CR_04420C_A	0.42	0.12	0.05	0.84	0	0
C5_02050W_A	0.82	0	0	0.86	0	0
C1_07200W_A	-0.06	0.63	0.47	-0.33	0	0
CR_09590W_A	-0.48	0	0	-0.37	0	0
C1_05670W_A	0.23	0	0	-0.12	0.24	0.14
C1_09860C_A	-0.05	0.75	0.62	0.08	0.52	0.38
C4_00360C_A	-0.42	0.02	0.01	-0.81	0	0
CR_04040C_A	0.21	0.23	0.11	0.19	0.28	0.17
C2_03160C_A	-0.04	0.73	0.59	-0.49	0	0
C1_08480C_A	-0.05	0.77	0.65	0.15	0.34	0.22
C2_01340W_A	0.09	0.46	0.29	-1.09	0	0
C2_00730C_A	0.08	0.46	0.3	0.34	0	0
C7_01640W_A	0.38	0	0	0.34	0	0
C5_02160W_A	0.03	0.95	0.91	-0.18	0.65	0.51
C4_04830W_A	0.25	0.01	0	0.33	0	0
C4_03930C_A	-0.7	0.02	0.01	-0.11	0.77	0.67
C3_05610W_A	0.02	0.94	0.88	-0.45	0	0
CR_02330C_A	-0.24	0.3	0.17	-0.44	0.05	0.02
C5_04610W_A	0.22	0.11	0.05	0.29	0.03	0.01

C4_04390W_A	-0.1	0.23	0.11	-0.67	0	0
C1_02250W_A	-0.49	0	0	-0.51	0	0
C1_13120C_A	0.46	0	0	0.55	0	0
C4_01420W_A	-0.15	0.56	0.4	0.06	0.82	0.74
C1_13980C_A	0.1	0.77	0.64	-0.14	0.65	0.52
C5_00120W_A	0.56	0	0	0.71	0	0
C4_02970C_A	0	0.99	0.97	0.04	0.78	0.69
CR_05330W_A	-0.65	0.14	0.06	-0.61	0.26	0.16
C2_03140C_A	-0.29	0.01	0	-0.29	0.03	0.01
C6_01910W_A	0.33	0	0	0.16	0.09	0.04
C3_07090W_A	0.13	0.44	0.28	0.35	0.01	0
C2_07250C_A	0.28	0.53	0.36	0.41	0.38	0.25
C6_04320C_A	-0.09	0.89	0.81	-0.04	0.96	0.93
C4_04040W_A	-0.07	0.68	0.53	-0.13	0.65	0.52
CR_06790C_A	-0.64	0	0	-1.29	0	0
C6_00380C_A	0.04	0.83	0.72	-0.13	0.43	0.3
C5_04330W_A	0.67	0	0	1.13	0	0
C3_00350W_A	0.17	0.37	0.22	0.44	0	0
C1_13820C_A	-0.6	0.08	0.03	0.25	0.49	0.35
C1_02430C_A	0.38	0	0	0.5	0	0
C2_01840C_A	-0.07	0.69	0.54	0.11	0.49	0.36
C2_01120W_A	1.35	0	0	1.16	0	0
C4_03600C_A	0.21	0.13	0.05	-0.12	0.45	0.32
C3_04890W_A	0.11	0.74	0.61	0.17	0.57	0.44
C2_01530C_A	0.17	0.02	0.01	0.17	0.06	0.03
C1_11600W_A	0.22	0.1	0.04	0.16	0.3	0.18
C5_04420W_A	0.53	0	0	0.55	0	0
C3_06730W_A	0.25	0.34	0.2	0.59	0.01	0
C4_07160W_A	0.06	0.88	0.81	-0.13	0.79	0.69
C3_05550C_A	0.28	0.03	0.01	-0.06	0.75	0.64
C6_04510C_A	0.08	0.56	0.39	-0.14	0.32	0.2
CR_00650W_A	0.07	0.88	0.79	-0.39	0.35	0.23
C1_00730C_A	0.33	0	0	0.64	0	0
C1_08570C_A	0.54	0	0	1.4	0	0
CR_04820W_A	-0.87	0.02	0.01	-0.83	0.17	0.09
C5_00190C_A	0.15	0.07	0.02	0.08	0.32	0.21
CR_10110W_A	-0.71	0	0	-1.46	0	0
C3_01100W_A	-0.81	0	0	-0.96	0	0
C3_04580C_A	0.31	0	0	-0.32	0	0
C6_01020W_A	0.03	0.95	0.91	-0.36	0.49	0.35
C5_01290C_A	-0.14	0.22	0.11	-0.17	0.15	0.08
C1_07700C_A	0.29	0.01	0	0.38	0	0
CR_03960C_A	0.25	0.18	0.09	0.47	0.01	0

C1_05010C_A	-0.03	0.86	0.78	0.05	0.77	0.67
C1_10040W_A	1.27	0	0	1.37	0	0
C1_02180W_A	-0.4	0.05	0.02	-0.66	0	0
C1_12540W_A	0	1	1	-0.14	0.52	0.38
C3_07820W_A	0.71	0	0	0.86	0	0
C3_06180C_A	0.44	0	0	0.48	0	0
C1_12330W_A	0.07	0.88	0.79	0.41	0.53	0.39
C1_12960C_A	0.03	0.82	0.71	0.23	0.04	0.02
C2_04510W_A	-0.23	0.09	0.04	-0.32	0.02	0.01
C1_01770W_A	-0.01	0.98	0.97	0.45	0.02	0.01
CR_01700C_A	-0.05	0.81	0.7	-0.13	0.46	0.32
C4_02930W_A	0.26	0.63	0.47	0.74	0.16	0.08
CR_09300C_A	0.01	0.97	0.94	-0.3	0	0
C4_06270C_A	-0.36	0	0	-0.52	0	0
C6_03800C_A	-0.25	0.07	0.03	-0.1	0.51	0.37
C2_05710C_A	-0.32	0	0	-0.56	0	0
C5_03560W_A	-0.08	0.71	0.57	0.13	0.49	0.35
C3_05230W_A	0.06	0.91	0.84	0.08	0.87	0.81
C3_00060W_A	-0.09	0.64	0.48	-0.2	0.23	0.13
C2_07670C_A	-0.27	0.06	0.02	0	0.99	0.98
CR_04480C_A	0.2	0.04	0.02	0.05	0.68	0.56
C2_04990W_A	-0.1	0.71	0.57	0.13	0.6	0.47
CR_07480W_A	0.7	0	0	0.84	0	0
CR_04550W_A	0.05	0.65	0.49	0.25	0.01	0
C6_03340C_A	0.46	0	0	0.49	0	0
C1_14030W_A	0.75	0	0	1.22	0	0
C6_02570C_A	0.38	0	0	0.48	0	0
C3_01660W_A	-0.07	0.82	0.71	-0.14	0.61	0.48
C7_02340C_A	-0.43	0	0	-0.3	0.06	0.03
CR_08290W_A	-0.08	0.54	0.38	-0.11	0.4	0.27
C3_01550C_A	0.21	0.01	0	-0.29	0	0
C5_01520C_A	0.02	0.91	0.84	-0.14	0.19	0.1
C4_05630W_A	0.18	0.06	0.02	0.25	0.02	0.01
C4_03450C_A	-0.3	0.02	0.01	0.06	0.76	0.65
CR_04110W_A	-0.7	0	0	-0.73	0	0
C1_13420C_A	0.06	0.62	0.46	0.14	0.27	0.16
C5_04910W_A	-1.16	0	0	-0.93	0	0
C5_02750C_A	-0.29	0.04	0.02	-0.17	0.4	0.27
C7_00380W_A	-0.12	0.42	0.26	-0.01	0.94	0.91
C1_04830W_A	0.76	0	0	0.78	0	0
CR_03830C_A	-0.18	0.14	0.06	-0.13	0.35	0.23
C2_06280C_A	-0.16	0.69	0.54	-0.35	0.35	0.23
C4_04440W_A	0.64	0	0	0.69	0	0

C7_03310W_A	0.41	0.32	0.18	2.18	0	0
CR_01090W_A	0.14	0.41	0.25	0.29	0.05	0.02
C6_02000W_A	-0.48	0.06	0.02	-0.88	0	0
CR_07290W_A	-0.37	0.27	0.14	-0.51	0.12	0.06
C3_00620C_A	-0.12	0.57	0.4	-0.14	0.5	0.36
C5_03960W_A	0.07	0.75	0.62	-0.08	0.7	0.59
CR_09490W_A	-0.55	0.01	0	-0.55	0	0
CR_10450C_A	0.02	0.92	0.86	0.13	0.44	0.31
C2_00340C_A	-0.19	0.03	0.01	-0.62	0	0
C3_04450C_A	0.11	0.84	0.74	0.78	0.2	0.11
C1_08450C_A	0.36	0	0	0.39	0	0
CR_07280W_A	-0.31	0.56	0.39	-0.01	0.99	0.98
C2_01380W_A	-1.05	0	0	-2.28	0	0
C3_04260W_A	0.45	0	0	0.99	0	0
C5_03020W_A	-0.71	0	0	-0.45	0.04	0.02
C2_05780C_A	0.08	0.51	0.35	0.02	0.87	0.81
C4_02620C_A	0	1	1	0.11	0.31	0.2
CR_03220C_A	0.31	0.31	0.17	0.34	0.25	0.15
C3_07240W_A	-0.16	0.78	0.66	0.06	0.94	0.9
C2_07290W_A	-0.11	0.24	0.13	-0.5	0	0
C4_00210W_A	-0.37	0.03	0.01	-0.34	0.06	0.03
BGI_novel_G000	0.07	0.77	0.65	-0.19	0.56	0.42
CR_09930W_A	0.16	0.17	0.08	-0.17	0.18	0.1
C1_08620W_A	-0.25	0.06	0.02	-0.47	0	0
C2_10630W_A	0.34	0.01	0	-0.96	0	0
C2_00680C_A	3.25	0	0	5.17	0	0
CR_05450C_A	0.1	0.63	0.47	0.07	0.74	0.63
C1_11340W_A	0.63	0.04	0.02	0.59	0.08	0.04
C7_02980C_A	0.03	0.86	0.77	-0.28	0.03	0.01
C1_09270W_A	0.12	0.69	0.54	-0.12	0.69	0.57
C7_02420C_A	0.09	0.77	0.65	-0.27	0.31	0.2
C2_08850C_A	-0.22	0.08	0.03	-0.38	0	0
CR_06520C_A	0.05	0.86	0.77	0.26	0.25	0.15
C4_04160W_A	-0.06	0.57	0.41	-0.43	0	0
C5_03750W_A	-0.34	0.5	0.34	0.21	0.72	0.61
CR_03320C_A	0.07	0.67	0.52	NA	NA	NA
CR_02780W_A	-0.74	0.06	0.02	-1.25	0	0
C1_05510C_A	0.2	0.51	0.34	0.36	0.18	0.1
CR_07000C_A	-0.17	0.15	0.07	-0.3	0.03	0.01
BGI_novel_G000	0.07	0.9	0.83	0.82	0.07	0.03
C1_12040W_A	0	1	0.99	0.09	0.84	0.77
C1_10760W_A	0.37	0	0	0.46	0	0
C4_00650W_A	0.32	0	0	0.06	0.71	0.6

C4_01970W_A	0.27	0.02	0.01	0.02	0.89	0.83
C1_05250W_A	NA	NA	NA	NA	NA	NA
CR_04290W_A	-0.04	0.89	0.81	0.02	0.96	0.93
C1_01200W_A	0.29	0.05	0.02	-0.05	0.82	0.74
C5_03500W_A	0.69	0	0	0.8	0	0
C5_03210C_A	-0.18	0.51	0.34	-0.13	0.66	0.53
C1_06790C_A	-0.22	0.09	0.03	-0.54	0	0
C5_02790C_A	-0.02	0.92	0.85	-0.24	0.02	0.01
C4_00100C_A	0.03	0.9	0.83	-0.15	0.49	0.35
C2_07750W_A	0.33	0.1	0.04	0.69	0	0
C2_10780C_A	0.67	0.04	0.01	0.71	0.02	0.01
C3_03760W_A	-0.04	0.86	0.77	-0.11	0.62	0.49
CR_10700W_A	-0.01	0.94	0.9	-0.11	0.43	0.29
C2_07370W_A	-0.13	0.53	0.36	-0.58	0	0
CR_10830C_A	0.25	0	0	0.12	0.43	0.3
C5_04390C_A	0.4	0.27	0.15	1.49	0.01	0
C7_02500C_A	0.86	0	0	1.29	0	0
C5_05330C_A	0.16	0.33	0.19	0.5	0	0
C1_09200W_A	-0.02	0.91	0.84	-0.69	0	0
C6_03040C_A	-0.08	0.7	0.55	0.04	0.84	0.78
CR_09690C_A	0.09	0.52	0.36	-1.33	0	0
C4_05270C_A	0.57	0.01	0	0.93	0	0
C2_04280W_A	0.29	0.21	0.11	-0.62	0.02	0.01
C2_02320C_A	0.02	0.88	0.8	0.11	0.27	0.16
C4_02580W_A	0.3	0.23	0.12	0.21	0.45	0.31
C5_00170W_A	0.2	0.03	0.01	-0.05	0.72	0.6
CR_05640C_A	-0.12	0.66	0.51	0.12	0.65	0.52
C1_04050C_A	0.07	0.67	0.52	NA	NA	NA
C1_05330C_A	0.14	0.56	0.39	0.53	0	0
C5_03160W_A	-0.25	0.08	0.03	-0.08	0.72	0.61
CR_06660W_A	-1.56	0	0	-2.93	0	0
C7_00340C_A	0.24	0	0	0.15	0.08	0.04
C3_05840W_A	-0.37	0.43	0.27	0.24	0.64	0.5
C1_05780W_A	0.1	0.32	0.18	0.06	0.63	0.5
C6_01300W_A	0.3	0.02	0.01	0.47	0	0
C1_09820C_A	NA	NA	NA	NA	NA	NA
C4_02130W_A	0.15	0.43	0.27	0.17	0.37	0.24
C1_01150C_A	-0.38	0.02	0.01	-0.39	0.02	0.01
C7_03030W_A	0.08	0.66	0.5	0.03	0.9	0.85
C3_05950W_A	-0.02	0.95	0.91	0.35	0.05	0.02
C1_03480C_A	-0.2	0.08	0.03	-0.16	0.24	0.14
C6_01130W_A	0.05	0.93	0.88	0.19	0.76	0.65
C3_04950W_A	0.41	0	0	0.55	0	0

C4_06490C_A	-0.88	0	0	-0.72	0	0
C4_01860C_A	0.88	0	0	0.59	0.03	0.01
C5_00820W_A	-0.31	0	0	-0.56	0	0
CR_08690C_A	0.3	0.09	0.04	0.72	0	0
CR_02910W_A	0.56	0	0	0.68	0	0
C7_01820C_A	0.26	0.04	0.01	0.17	0.28	0.17
C2_07600C_A	-0.47	0	0	-1.09	0	0
C5_03090W_A	-0.05	0.73	0.59	-0.03	0.83	0.76
C6_01680C_A	0.29	0.54	0.37	0.29	0.58	0.44
C4_03860C_A	0.26	0.17	0.08	0.49	0	0
C2_01480W_A	0	0.99	0.98	-0.04	0.72	0.61
C6_04600W_A	0.39	0.02	0.01	0.52	0	0
C1_12410C_A	-0.34	0.01	0	0.01	0.98	0.96
C3_01900C_A	-0.55	0	0	-1.01	0	0
C2_01160W_A	0.13	0.4	0.24	0.19	0.14	0.08
C1_03090W_A	0.03	0.76	0.63	-0.43	0	0
CR_04300W_A	-0.39	0	0	-0.64	0	0
C3_03990C_A	0.54	0.21	0.11	0.73	0.13	0.07
C6_01900C_A	0.41	0	0	0.52	0	0
C1_05530C_A	-0.02	0.89	0.81	-0.5	0	0
CR_08560C_A	-0.08	0.43	0.27	-0.17	0.08	0.04
CR_07530C_A	0.39	0.01	0	0.39	0.01	0
C6_01780C_A	-0.29	0.14	0.06	-0.3	0.11	0.05
C1_10950C_A	-0.21	0.38	0.23	0.46	0.03	0.01
C1_03350C_A	-0.18	0.09	0.04	0.05	0.69	0.57
C3_01620W_A	-0.09	0.72	0.58	0.28	0.15	0.08
C5_01190W_A	-0.26	0.06	0.02	0.21	0.13	0.07
C5_00580W_A	0.28	0.01	0	0.47	0	0
C7_00800C_A	-0.25	0.31	0.17	-0.11	0.69	0.57
C4_01220C_A	0.54	0.18	0.09	1.49	0	0
C2_05750W_A	-1.64	0	0	-2.18	0	0
C1_11740W_A	0.23	0.15	0.07	0.36	0.02	0.01
C6_01010W_A	0.05	0.92	0.86	-0.04	0.96	0.93
C6_03220W_A	-0.74	0	0	-0.08	0.81	0.72
C2_07620W_A	0.17	0.76	0.63	0.05	0.94	0.91
CR_08200C_A	0.25	0.04	0.01	-0.08	0.58	0.44
C2_08120W_A	-0.19	0.02	0.01	-0.57	0	0
C7_02090C_A	0.41	0.09	0.04	0.38	0.12	0.06
C5_00900C_A	-0.03	0.82	0.71	-0.04	0.79	0.7
C4_06820C_A	0.78	0	0	1.82	0	0
C2_04600C_A	-0.18	0.01	0	-0.57	0	0
C5_02360C_A	-0.32	0.5	0.33	-0.24	0.73	0.62
C3_01480C_A	0.03	0.94	0.89	-0.31	0.38	0.25

C3_05790C_A	0.15	0.73	0.6	0.1	0.82	0.74
C3_06550C_A	0.16	0.41	0.25	0.03	0.88	0.82
C6_01490C_A	0.03	0.95	0.91	0.22	0.63	0.5
C1_03560C_A	0.15	0.16	0.08	0	1	1
C7_01970C_A	0.14	0.07	0.03	-0.14	0.2	0.11
C2_10170C_A	0.2	0.2	0.1	0.51	0	0
CR_05200C_A	0.21	0.19	0.09	0.1	0.61	0.47
C2_05300C_A	-0.11	0.24	0.12	-0.24	0.01	0
C3_07330W_A	0.14	0.56	0.39	0.85	0	0
C1_01470W_A	-0.39	0	0	-0.13	0.41	0.28
C3_06810W_A	0.17	0.37	0.22	0.24	0.1	0.05
C2_10110W_A	-0.01	0.96	0.93	-0.43	0	0
C2_05100C_A	-0.1	0.37	0.22	-0.09	0.39	0.26
C1_06550W_A	0.33	0	0	0.04	0.66	0.53
C1_13240W_A	0.34	0.1	0.04	0.44	0.03	0.01
C3_04710W_A	-0.04	0.93	0.88	0.04	0.91	0.85
C3_06140W_A	0.25	0.1	0.04	-0.14	0.42	0.29
CR_01200W_A	0.11	0.74	0.6	0.21	0.45	0.31
C5_01010W_A	-0.49	0	0	-0.19	0.32	0.2
C5_04180W_A	-0.54	0	0	-0.82	0	0
C4_00450C_A	-0.63	0	0	-2	0	0
C5_05120W_A	0.08	0.34	0.2	0.11	0.27	0.16
C3_05150W_A	0.18	0.02	0.01	0.21	0.04	0.01
C1_02890C_A	-0.64	0.14	0.06	-0.41	0.43	0.29
C4_06440C_A	0.3	0.11	0.05	0.01	0.98	0.96
C2_03670W_A	0.1	0.85	0.75	0.21	0.69	0.57
CR_06340C_A	-0.07	0.44	0.28	-0.24	0.01	0
C2_05440W_A	0.27	0.04	0.01	0.57	0	0
C3_06790W_A	0.09	0.87	0.79	0.07	0.93	0.89
CR_10820W_A	-0.01	0.98	0.95	0.07	0.68	0.56
C1_01090C_A	0.34	0	0	0.2	0.17	0.09
C3_00130C_A	0.32	0	0	0.23	0.01	0
C1_06840C_A	-0.15	0.5	0.33	-0.31	0.1	0.05
CR_06740W_A	-0.36	0.1	0.04	-0.73	0	0
CR_05990C_A	0.01	0.98	0.96	0.3	0.01	0
C4_07200C_A	0.51	0	0	-0.28	0.04	0.02
C2_00240C_A	0.71	0	0	0.32	0.22	0.12
CR_02750C_A	NA	NA	NA	NA	NA	NA
C6_00110C_A	-0.39	0	0	-0.18	0.21	0.12
C6_01170W_A	-1.18	0	0	-1.06	0	0
C3_01740C_A	NA	NA	NA	0.12	0.67	0.55
C1_11510C_A	-0.52	0	0	-0.68	0	0
C4_05970W_A	0.21	0.03	0.01	0.18	0.09	0.04

C5_04140W_A	-0.28	0.54	0.37	-0.34	0.63	0.5
C6_03050C_A	-0.17	0.56	0.39	-0.27	0.31	0.2
C5_02400W_A	0.16	0.06	0.02	0.16	0.11	0.06
C2_06040C_A	0.33	0.17	0.08	0.24	0.35	0.23
CR_02410W_A	-0.18	0.29	0.16	-0.05	0.82	0.74
C2_08750W_A	0.17	0.75	0.62	-0.29	0.68	0.56
C1_06660W_A	-0.52	0	0	-0.55	0	0
CR_00020W_A	0.1	0.37	0.22	0.41	0	0
C6_03950C_A	-0.21	0.26	0.14	-0.2	0.24	0.14
C1_11000C_A	-1.52	0	0	-1.37	0	0
C2_08490W_A	-0.33	0	0	-0.48	0	0
C7_01150W_A	-0.26	0.16	0.07	-0.54	0	0
C1_10670C_A	-0.75	0	0	-1.18	0	0
CR_06250W_A	0.22	0.51	0.34	0.06	0.87	0.81
C1_12290C_A	0.55	0	0	0.75	0	0
CR_05380C_A	0.31	0	0	0.64	0	0
C1_05710C_A	-0.06	0.92	0.85	0.47	0.24	0.14
C1_00130C_A	0.41	0	0	0.17	0.18	0.1
C5_05340W_A	-0.14	0.26	0.14	-0.37	0	0
C1_08050W_A	0.05	0.75	0.61	0.42	0	0
C2_01660C_A	-0.22	0.17	0.08	-0.1	0.59	0.46
CR_03610C_A	-0.23	0	0	-0.51	0	0
C5_05220W_A	-0.04	0.92	0.86	0.41	0.19	0.1
C1_00060W_A	0.28	0	0	0.11	0.29	0.18
C5_03620W_A	-0.28	0.14	0.06	-0.36	0.06	0.03
C3_05770C_A	-0.36	0.02	0.01	-0.27	0.1	0.05
C1_10550C_A	0.29	0.21	0.1	-0.54	0.02	0.01
C1_03140W_A	0.38	0.12	0.05	0.39	0.12	0.06
C4_02400C_A	-0.8	0	0	-0.59	0.01	0
C6_04550C_A	0.6	0	0	0.29	0.02	0.01
C4_06660W_A	0.03	0.85	0.76	-0.23	0.03	0.01
C2_01170C_A	0.08	0.5	0.33	0.05	0.68	0.55
C5_00460C_A	0.28	0.06	0.02	0.03	0.9	0.85
C5_01960C_A	-0.17	0.07	0.03	-0.63	0	0
CR_07940W_A	0	1	0.99	-0.3	0.25	0.15
C1_05480C_A	0.12	0.74	0.6	0.55	0.04	0.02
CR_02950C_A	-0.01	0.97	0.94	0.21	0.08	0.04
C6_03300C_A	0.11	0.42	0.26	0.26	0.02	0.01
C2_06520C_A	-0.05	0.87	0.78	-0.22	0.41	0.28
CR_01980C_A	0.41	0	0	0.43	0	0
C6_01000C_A	-0.28	0.04	0.01	-0.13	0.39	0.26
C3_05040C_A	-0.18	0.29	0.16	-0.37	0.02	0.01
C7_03080W_A	0.33	0	0	0.46	0	0

C4_01790W_A	-0.17	0.47	0.31	0.12	0.67	0.54
C2_06850W_A	-0.67	0	0	-0.35	0.16	0.08
CR_05880W_A	-0.16	0.54	0.37	-0.06	0.81	0.73
C1_00400W_A	-1.27	0	0	-1.44	0	0
C6_02060W_A	0.16	0.14	0.06	0.16	0.14	0.07
C1_07390W_A	0.05	0.64	0.47	0.14	0.21	0.12
C3_05580C_A	0.78	0	0	-0.95	0	0
C6_01670W_A	0.02	0.9	0.83	-0.34	0	0
C1_06250W_A	-0.37	0.06	0.02	-0.14	0.55	0.41
CR_10480W_A	0.04	0.88	0.8	0.74	0	0
C4_00510C_A	-1.12	0	0	-1.68	0	0
C5_01210W_A	0.56	0	0	0.8	0	0
C1_12630C_A	-0.52	0.04	0.02	-0.3	0.29	0.18
CR_04160C_A	-0.9	0	0	-1.14	0	0
C7_00730W_A	-0.68	0	0	-0.1	0.52	0.38
C4_02760C_A	0.05	0.74	0.61	0.19	0.18	0.1
C2_06860W_A	-0.23	0.13	0.06	-0.37	0.01	0
C6_02120W_A	0.73	0	0	0.79	0	0
C2_04560W_A	-0.3	0.25	0.13	-0.14	0.62	0.49
C6_01930W_A	0.05	0.62	0.46	-0.19	0.1	0.05
C1_05770C_A	-0.36	0	0	-2.01	0	0
C2_00590W_A	0.04	0.82	0.72	0.15	0.3	0.19
C3_07590W_A	0.47	0.01	0	0.76	0	0
C4_04920W_A	0.08	0.59	0.42	0.16	0.27	0.17
C7_01720W_A	-0.03	0.85	0.76	0.01	0.98	0.96
C1_04200C_A	-0.18	0.33	0.19	-0.55	0	0
C1_07900W_A	-0.07	0.61	0.45	-0.65	0	0
CR_07030C_A	-0.91	0	0	-0.59	0.05	0.02
C2_06110W_A	0.26	0.02	0.01	0.26	0.03	0.01
C3_04700W_A	-0.21	0.18	0.09	-0.16	0.33	0.21
C2_03490C_A	0.12	0.6	0.43	-0.06	0.8	0.72
C6_03390W_A	-0.26	0.06	0.02	-0.38	0	0
C5_03310C_A	-0.02	0.97	0.94	1.19	0	0
C5_05490C_A	0.05	0.62	0.45	-0.29	0.01	0
C1_00710C_A	-0.03	0.77	0.65	-0.27	0	0
C5_04980W_A	0.08	0.87	0.79	-0.1	0.84	0.77
C3_07950C_A	0.56	0	0	0.76	0	0
CR_03060W_A	0.05	0.52	0.36	-0.18	0.04	0.02
C1_08000W_A	-0.29	0.45	0.28	0.12	0.77	0.67
C6_03240W_A	0.47	0.33	0.19	1.05	0.04	0.02
C2_10230W_A	-0.89	0	0	-0.67	0	0
CR_02000C_A	-0.56	0	0	-1.01	0	0
C2_10060C_A	-0.69	0	0	-0.81	0	0

C7_00940W_A	-0.19	0.06	0.02	-0.49	0	0
CR_04350C_A	0.41	0	0	0.34	0.01	0
C1_08580C_A	-0.02	0.93	0.87	-0.05	0.82	0.74
C4_02900C_A	0.05	0.81	0.7	0.46	0	0
C7_03250C_A	0.91	0	0	0.97	0	0
C4_04770C_A	-0.04	0.76	0.63	-0.4	0	0
CR_01930C_A	0.44	0.03	0.01	0.47	0.02	0.01
C1_02940C_A	0.3	0.02	0.01	0.35	0.01	0
CR_03360W_A	-0.78	0	0	-0.61	0	0
C1_04120C_A	-1.09	0	0	-0.75	0	0
CR_06380C_A	0.49	0	0	0.82	0	0
C1_14590C_A	-0.2	0.63	0.47	0.11	0.8	0.71
C1_12470W_A	0.07	0.9	0.83	0.22	0.69	0.57
C4_05080C_A	0.81	0	0	1.2	0	0
CR_01610C_A	0.36	0	0	0.54	0	0
C2_06960W_A	0.11	0.3	0.17	-0.3	0.01	0
C5_01690C_A	-0.1	0.34	0.2	-0.2	0.04	0.02
C3_06100C_A	-0.01	0.97	0.95	0.17	0.44	0.3
C1_06770W_A	0.29	0.01	0	0.38	0	0
CR_05510W_A	0.6	0	0	0.48	0	0
C2_01330C_A	-0.15	0.67	0.51	0.14	0.68	0.56
C6_03750C_A	0.25	0	0	0.26	0.02	0.01
C6_03430C_A	0.21	0.09	0.03	0.25	0.04	0.02
CR_01620C_A	-0.4	0	0	-0.66	0	0
C4_04010W_A	0.22	0.26	0.14	0.15	0.47	0.33
C7_01750W_A	0.09	0.81	0.7	0.25	0.42	0.28
CR_02530W_A	-0.38	0	0	-0.41	0	0
C2_03080W_A	0.35	0	0	0.38	0	0
C2_05510C_A	0.22	0.19	0.09	0.34	0.05	0.02
C5_00130C_A	-0.03	0.83	0.73	-0.14	0.27	0.16
C2_10750C_A	0.44	0	0	0.51	0	0
C2_09760W_A	-0.46	0.03	0.01	-0.25	0.29	0.18
CR_01900C_A	0.15	0.52	0.35	-0.34	0.13	0.07
C7_02880C_A	-0.1	0.86	0.77	0.15	0.83	0.75
C2_07190C_A	0.03	0.79	0.67	-0.26	0.01	0
C4_06970C_A	-0.04	0.95	0.9	-0.7	0.25	0.15
CR_08460W_A	0.28	0	0	0.44	0	0
C2_02590W_A	0.31	0	0	0.24	0.07	0.03
C1_13650C_A	-0.11	0.48	0.31	-0.08	0.66	0.54
C4_01260W_A	-0.27	0.12	0.05	0.01	0.97	0.95
C2_00480C_A	0.29	0.24	0.12	0.53	0.01	0
C1_13050W_A	-0.17	0.02	0.01	-0.68	0	0
C6_02480W_A	-0.18	0.23	0.12	-0.14	0.36	0.23

C1_13510C_A	0.1	0.48	0.32	-0.03	0.89	0.83
C2_01590W_A	0.5	0	0	0.75	0	0
C1_11200W_A	-0.73	0	0	-0.92	0	0
C6_01500C_A	-0.18	0.68	0.53	-0.2	0.63	0.5
C1_06150W_A	0.33	0.27	0.14	0.22	0.53	0.4
C5_01470C_A	0.16	0.71	0.57	1.22	0	0
C6_04340W_A	-0.09	0.67	0.51	-0.31	0.1	0.05
CR_08610W_A	-0.1	0.53	0.36	-0.38	0.02	0.01
C7_00620W_A	0.17	0.06	0.02	-0.16	0.06	0.03
C5_00210C_A	-0.36	0	0	-0.03	0.86	0.79
C1_00080C_A	0.07	0.57	0.41	-0.1	0.5	0.37
C4_05250W_A	-1.13	0	0	-0.58	0.02	0.01
C7_03550C_A	-0.27	0.48	0.32	0.06	0.94	0.91
C1_05890W_A	-0.07	0.89	0.82	-0.48	0.37	0.25
C3_01310W_A	-0.51	0.01	0	0.05	0.83	0.76
C4_06410W_A	0.01	0.96	0.92	-0.12	0.55	0.42
CR_01260W_A	-0.01	0.96	0.93	-0.02	0.9	0.85
C4_03150W_A	-0.04	0.83	0.72	-0.33	0.03	0.01
C2_08920W_A	0.31	0.08	0.03	0.36	0.04	0.02
C5_03570W_A	0.49	0	0	0.55	0	0
C1_11410C_A	-0.58	0.21	0.1	-0.06	0.93	0.89
C3_04960W_A	-0.36	0	0	-0.69	0	0
C3_04630W_A	0.51	0.01	0	0.71	0	0
C3_06360C_A	0.21	0.01	0	0.07	0.51	0.37
C7_02540W_A	-0.06	0.84	0.74	0.24	0.27	0.16
CR_01550C_A	-0.36	0.05	0.02	0	1	0.99
C5_05510C_A	-0.25	0.65	0.49	-0.15	0.82	0.74
C1_14140C_A	-0.92	0	0	-0.76	0	0
C1_14190C_A	-0.04	0.77	0.65	-0.48	0	0
C5_02240W_A	-0.26	0.47	0.31	-0.4	0.25	0.15
C3_07400W_A	-0.18	0.32	0.18	-0.26	0.15	0.08
C1_03300C_A	-0.09	0.37	0.22	-0.12	0.24	0.14
C5_00870C_A	0.37	0.42	0.26	0.88	0.03	0.01
C2_04190C_A	-0.22	0.14	0.06	-0.14	0.36	0.24
C3_03070W_A	-0.1	0.61	0.44	-0.36	0.01	0
CR_01330W_A	0.17	0.63	0.47	-0.06	0.86	0.8
CR_00680W_A	-0.42	0.08	0.03	0.03	0.9	0.84
C1_13930W_A	0.01	0.95	0.91	0.3	0.01	0
C6_00160W_A	-0.11	0.53	0.37	0.07	0.69	0.58
CR_06830C_A	0.04	0.88	0.8	-0.03	0.91	0.86
C2_07860W_A	-0.2	0.34	0.2	0.16	0.44	0.31
C2_08010W_A	0.14	0.17	0.08	0.28	0.01	0
C2_04680W_A	-0.36	0.04	0.01	-0.47	0.01	0

C2_04960C_A	-0.42	0.03	0.01	-0.48	0.01	0
CR_01400W_A	0.73	0	0	0.68	0	0
CR_05390W_A	0.45	0	0	1.04	0	0
C5_04800W_A	0.47	0	0	0.01	0.94	0.91
C4_02200C_A	0.32	0.29	0.16	0.39	0.19	0.11
C4_07260W_A	NA	NA	NA	NA	NA	NA
C3_06920W_A	0.13	0.17	0.08	0.44	0	0
C7_02280W_A	0.15	0.5	0.33	0.24	0.51	0.38
C2_02130C_A	0.44	0.37	0.22	1.16	0.02	0.01
C7_02370W_A	0.09	0.56	0.39	0.46	0	0
C1_03020C_A	-0.17	0.01	0	-0.55	0	0
C2_02610C_A	-0.73	0	0	-1.51	0	0
C1_09230C_A	0.04	0.82	0.71	0.12	0.42	0.28
C5_04700C_A	0.07	0.44	0.28	-0.28	0	0
C5_04320C_A	0.73	0	0	1.29	0	0
C2_03350W_A	0.29	0.46	0.3	1.19	0.04	0.02
CR_02130W_A	-0.35	0	0	-0.15	0.07	0.03
CR_00700W_A	-0.05	0.87	0.79	-0.41	0.1	0.05
C1_06430C_A	0.07	0.81	0.71	0.1	0.82	0.74
C4_02300W_A	-0.12	0.32	0.18	0.18	0.16	0.08
CR_04780W_A	-0.46	0.03	0.01	-0.25	0.27	0.17
C1_13320C_A	-0.36	0	0	-0.47	0	0
C2_05050C_A	-0.28	0.05	0.02	-0.59	0	0
C1_05520W_A	0.49	0	0	0.67	0	0
CR_07540C_A	-0.13	0.53	0.37	-0.25	0.5	0.36
C7_02080W_A	0.47	0	0	0.67	0	0
C2_10010C_A	0.23	0.01	0	-0.22	0.02	0.01
C7_00590W_A	-0.21	0.19	0.09	-0.19	0.23	0.13
C1_12950W_A	-0.35	0.04	0.01	-0.07	0.72	0.6
C4_02530W_A	-0.31	0.2	0.1	0.19	0.44	0.3
C1_09660W_A	0.66	0	0	0.8	0	0
C5_00450C_A	0.41	0.02	0	0.71	0	0
C1_05110C_A	0.11	0.14	0.06	-0.04	0.69	0.58
C6_00270W_A	0.12	0.37	0.22	-0.06	0.7	0.59
C1_11980W_A	-0.2	0.5	0.33	-0.04	0.9	0.85
C1_09070W_A	-0.25	0.07	0.03	-0.2	0.18	0.1
CR_05650W_A	-0.28	0.09	0.04	-0.04	0.82	0.74
C2_09080C_A	NA	NA	NA	NA	NA	NA
C7_03620C_A	-0.2	0.25	0.13	-0.14	0.5	0.36
C4_03500C_A	0.16	0.77	0.64	0.5	0.37	0.24
C3_06580W_A	-0.03	0.96	0.92	0.23	0.71	0.59
C2_09460C_A	-0.23	0.02	0.01	-0.36	0	0
C1_01590C_A	-0.05	0.61	0.44	-0.11	0.26	0.16

C1_01370C_A	-0.24	0	0	-0.59	0	0
C6_00790C_A	-1.01	0	0	-1.48	0	0
C4_04700W_A	-0.11	0.15	0.07	-0.66	0	0
C4_05260W_A	-0.4	0.01	0	-0.31	0.06	0.03
C2_06460W_A	-0.13	0.38	0.23	-0.05	0.78	0.69
C5_02150C_A	0.1	0.5	0.34	0.09	0.55	0.41
C2_05900W_A	0.38	0.4	0.24	-0.01	0.99	0.97
C4_04210C_A	0.28	0.42	0.26	0.27	0.45	0.32
C1_09700W_A	-0.04	0.93	0.88	0.39	0.56	0.43
C1_03250C_A	0.15	0.45	0.29	0.37	0.02	0.01
C2_00120C_A	-0.08	0.46	0.29	-0.52	0	0
C1_03610C_A	0.27	0.11	0.05	0.11	0.59	0.46
CR_00010C_A	-0.18	0.07	0.03	-0.29	0.01	0
C7_02020W_A	0.28	0.04	0.01	0.42	0	0
C6_04540C_A	0.43	0	0	0.41	0	0
C1_11950W_A	0.7	0.02	0.01	0.74	0.02	0.01
CR_00590W_A	0.08	0.62	0.46	0.1	0.55	0.41
C4_06310C_A	0.28	0.59	0.43	0.66	0.2	0.11
C5_02900W_A	-0.21	0.12	0.05	-0.19	0.31	0.2
C2_00230W_A	0.02	0.91	0.84	-0.28	0.09	0.04
BGI_novel_G000	-0.16	0.32	0.18	-0.51	0	0
CR_03940W_A	-0.54	0	0	-0.62	0	0
C4_06110C_A	0.25	0.01	0	0.25	0.01	0
C1_00780C_A	0.18	0.49	0.33	0.8	0	0
C1_09980C_A	0.45	0	0	0.9	0	0
C3_02410C_A	-0.12	0.55	0.39	-0.37	0.03	0.01
C1_09330W_A	-0.63	0.09	0.03	-0.66	0.1	0.05
C3_03360W_A	-0.25	0.01	0	-0.89	0	0
C3_00520W_A	0.64	0	0	0.8	0	0
C2_04320W_A	-0.02	0.96	0.93	0.07	0.82	0.74
CR_01300W_A	0.16	0.61	0.45	0.23	0.42	0.29
C4_04220W_A	-0.61	0.02	0.01	-1.25	0	0
C2_02520W_A	-0.02	0.95	0.91	0.1	0.69	0.58
C4_06340W_A	0.35	0	0	0.66	0	0
C7_01020C_A	-0.34	0	0	-0.36	0	0
C1_12580W_A	-0.05	0.83	0.72	-0.16	0.43	0.29
C3_03150W_A	-0.08	0.65	0.5	0.09	0.62	0.49
C1_12710C_A	-0.41	0.01	0	0.01	0.98	0.96
C1_00900W_A	-1.11	0	0	-1.25	0	0
C1_14390W_A	-0.53	0.07	0.03	-0.24	0.49	0.35
CR_08420W_A	0.35	0.01	0	0.28	0.06	0.03
C4_05330C_A	-0.53	0.02	0.01	-0.3	0.19	0.11
CR_10520C_A	0.12	0.67	0.52	0.36	0.11	0.06

C3_00340W_A	-0.39	0	0	-0.31	0.02	0.01
C7_03470W_A	0.05	0.93	0.87	0.09	0.86	0.79
C5_01640W_A	-0.15	0.2	0.1	-0.07	0.61	0.47
CR_09570W_A	-0.25	0.32	0.18	0.12	0.68	0.55
C1_00570C_A	-0.07	0.72	0.58	0.14	0.4	0.27
C1_10890C_A	0.47	0	0	-0.07	0.56	0.43
C6_02270C_A	-0.2	0.26	0.14	0.01	0.98	0.96
C4_04900W_A	-0.14	0.04	0.01	-0.61	0	0
C1_02580W_A	0.22	0.67	0.51	0.08	0.9	0.85
C1_03710C_A	0.28	0.04	0.01	0.64	0	0
C2_05760C_A	0.34	0.01	0	0.38	0.01	0
C2_00780W_A	0.07	0.77	0.65	0.25	0.17	0.09
C1_07470C_A	-0.27	0.26	0.14	-0.26	0.26	0.15
C6_01470W_A	0.12	0.79	0.67	-0.16	0.72	0.6
C1_01830C_A	0.4	0	0	0.55	0	0
C3_07480W_A	-0.05	0.71	0.57	-0.17	0.17	0.09
C2_00830C_A	0.01	0.94	0.89	0.02	0.89	0.83
C5_01990W_A	0.07	0.67	0.52	0.25	0.5	0.36
CR_07390C_A	-0.26	0	0	-0.43	0	0
C4_05400C_A	-0.82	0	0	-0.44	0.03	0.01
C1_04700C_A	-0.68	0	0	-1.34	0	0
C1_12830C_A	-0.25	0.08	0.03	-0.18	0.23	0.13
CR_07510W_A	-0.17	0.33	0.19	0	0.99	0.98
CR_09230C_A	0.47	0	0	0.7	0	0
C3_02310W_A	-0.2	0.11	0.05	-0.4	0	0
C1_04950C_A	-0.34	0.09	0.04	-0.71	0	0
C7_04130C_A	-0.19	0.03	0.01	-0.43	0	0
C7_02250W_A	NA	NA	NA	0.12	0.66	0.53
C2_01850W_A	0.06	0.55	0.39	0.06	0.57	0.43
C1_12550C_A	-0.48	0	0	0.04	0.82	0.74
C4_00270W_A	0.74	0	0	0.81	0	0
C3_00270C_A	0.44	0	0	0.53	0	0
C6_02220W_A	0.38	0	0	0.49	0	0
C1_08180C_A	-0.16	0.12	0.05	-0.15	0.19	0.1
C6_04520W_A	-0.01	0.96	0.92	-0.36	0	0
C1_06180W_A	0.07	0.45	0.29	0.13	0.19	0.11
C5_03400C_A	-0.31	0.01	0	-0.31	0.01	0
C5_01500C_A	-0.36	0.08	0.03	-0.03	0.9	0.84
C2_08290C_A	0.31	0	0	-0.1	0.28	0.17
C1_05400C_A	-0.12	0.7	0.55	0.19	0.47	0.34
C1_00450C_A	0.34	0.06	0.02	0.7	0	0
C4_05960W_A	0.01	0.94	0.89	0.21	0.04	0.02
C5_01940W_A	0.07	0.6	0.43	0.12	0.3	0.19

C3_04740C_A	-0.16	0.46	0.3	0.18	0.36	0.24
C1_13800C_A	-0.32	0.12	0.05	-0.42	0.04	0.02
C1_00200C_A	0.94	0.02	0.01	1.29	0	0
C2_01740C_A	-0.28	0.13	0.06	-0.22	0.25	0.15
C1_13430C_A	-0.43	0.19	0.09	-0.22	0.54	0.4
C1_01530C_A	-0.04	0.71	0.57	-0.41	0	0
C5_01110W_A	-0.26	0.11	0.05	0.25	0.11	0.05
C3_00470W_A	0.22	0.03	0.01	-0.69	0	0
C1_10190W_A	-0.15	0.37	0.22	-0.26	0.09	0.04
C1_12200W_A	0.84	0	0	0.82	0	0
C3_02620C_A	-0.08	0.85	0.76	-0.49	0.16	0.09
C2_09670C_A	-0.04	0.81	0.7	-0.09	0.5	0.36
C2_04340C_A	-0.06	0.87	0.79	-0.04	0.92	0.87
C2_06870C_A	-1.09	0	0	-1.38	0	0
C2_03780C_A	-0.05	0.76	0.63	-0.61	0	0
C4_01100C_A	-0.71	0	0	-0.82	0	0
C4_03370C_A	-0.47	0.04	0.01	-0.76	0	0
C2_03230C_A	0.08	0.46	0.3	-0.08	0.52	0.39
C2_08350C_A	-0.01	0.98	0.96	-0.13	0.82	0.74
C7_03420C_A	-0.94	0	0	-1.41	0	0
C3_07780C_A	0.11	0.24	0.12	0.04	0.74	0.63
C7_00060C_A	0.25	0.03	0.01	0.17	0.2	0.11
C2_04800C_A	0.22	0.25	0.13	0.22	0.26	0.15
C3_01800C_A	-0.12	0.47	0.31	-0.12	0.44	0.3
C3_02260C_A	-0.17	0.2	0.1	-0.03	0.86	0.8
C2_06190W_A	0.02	0.84	0.74	-0.16	0.11	0.05
C6_04140C_A	-0.34	0.01	0	-0.26	0.08	0.04
C1_14240W_A	0.06	0.55	0.39	-0.14	0.16	0.09
C6_02330W_A	0	0.99	0.97	0.09	0.58	0.44
C7_02780W_A	0.13	0.09	0.03	0.2	0.02	0.01
C4_00260W_A	0.19	0.04	0.01	-0.03	0.8	0.71
C1_13580W_A	0.94	0	0	1.21	0	0
C2_06310C_A	-0.02	0.84	0.75	-0.29	0	0
C1_08070W_A	-0.38	0	0	-1.14	0	0
C7_02510W_A	-0.35	0.06	0.02	-0.04	0.86	0.79
C3_06700C_A	0.3	0	0	0.2	0	0
C3_02820C_A	-0.04	0.85	0.75	0.2	0.29	0.18
C2_06770W_A	-0.27	0.03	0.01	-0.07	0.65	0.53
C3_06280W_A	-0.03	0.76	0.64	-0.15	0.13	0.07
C2_02390W_A	-0.11	0.72	0.58	-0.19	0.53	0.39
C2_09710C_A	0.39	0	0	0.12	0.25	0.15
C2_02200W_A	-0.03	0.86	0.77	-0.18	0.15	0.08
C6_02660C_A	0.67	0.06	0.02	0.61	0.14	0.07

CR_00270C_A	-0.03	0.92	0.86	0.46	0.01	0
C2_03690C_A	0	1	1	1.19	0	0
CR_06140W_A	-0.13	0.28	0.15	-0.04	0.76	0.66
C4_01140C_A	-0.02	0.94	0.89	-0.18	0.33	0.21
C2_04210W_A	0.02	0.97	0.95	-0.16	0.66	0.54
C3_00410C_A	0.01	0.99	0.98	-0.56	0.17	0.09
C5_00510W_A	-0.51	0.22	0.11	-0.82	0.06	0.03
C3_00420W_A	-0.16	0.14	0.06	-0.25	0.02	0.01
C4_03780C_A	-0.1	0.48	0.31	-0.22	0.05	0.02
C2_08480W_A	-0.94	0	0	-0.94	0	0
C3_06480C_A	0.23	0.11	0.05	0.43	0	0
C6_02910W_A	-0.11	0.73	0.59	-0.16	0.56	0.42
C1_12910W_A	NA	NA	NA	0.12	0.67	0.55
C5_04860C_A	-0.13	0.72	0.58	-0.12	0.73	0.62
C4_01670C_A	-0.4	0.03	0.01	-0.1	0.69	0.57
C4_06910W_A	-0.91	0.03	0.01	-1.21	0.02	0.01
C2_04060C_A	-0.1	0.66	0.51	-0.46	0.01	0.01
C2_02930C_A	-0.48	0	0	-0.55	0	0
C1_07410C_A	0.08	0.44	0.28	0.48	0	0
CR_01130W_A	0.07	0.67	0.52	0.18	0.19	0.1
C6_01940W_A	0.3	0.57	0.4	1.35	0	0
C3_03710W_A	0.18	0.24	0.13	-0.26	0.11	0.05
C3_05450C_A	0.17	0.21	0.11	0.28	0.03	0.01
C7_03860W_A	0.41	0	0	0.19	0.09	0.04
CR_01030W_A	-0.32	0.06	0.02	-0.2	0.32	0.2
C1_11770C_A	0.19	0.06	0.02	0.51	0	0
C1_01220C_A	0.1	0.62	0.45	0.41	0.01	0
CR_02190C_A	-0.33	0.02	0.01	-0.05	0.78	0.68
C7_03760W_A	-0.26	0.13	0.06	-0.11	0.55	0.42
C6_04100W_A	0.09	0.32	0.18	0.18	0.04	0.02
CR_08880C_A	-0.32	0.54	0.38	-0.39	0.55	0.41
C4_01310W_A	-0.4	0.18	0.09	-0.07	0.83	0.76
C2_06240W_A	-1.32	0	0	-0.4	0.45	0.31
C4_05210W_A	-0.25	0.06	0.02	-0.2	0.18	0.1
CR_09900C_A	-0.44	0	0	-0.35	0.02	0.01
C4_00660W_A	-0.23	0.02	0.01	-0.46	0	0
CR_02850C_A	-0.04	0.8	0.69	0.52	0	0
C3_01560W_A	-0.77	0	0	-0.83	0	0
C3_02160C_A	0.33	0	0	0.14	0.15	0.08
C5_04710W_A	0.14	0.51	0.35	0.17	0.41	0.27
C1_04820C_A	0.04	0.87	0.79	0.28	0.11	0.06
C2_02410W_A	0.05	0.75	0.61	-0.04	0.73	0.62
C1_00520W_A	0.38	0	0	0.43	0	0

CR_10280W_A	0.14	0.37	0.22	-0.15	0.38	0.25
C5_04210C_A	NA	NA	NA	NA	NA	NA
CR_06930W_A	-0.11	0.57	0.4	-0.15	0.39	0.27
C3_01690W_A	-0.14	0.39	0.24	-0.02	0.92	0.87
CR_10060W_A	-0.18	0.1	0.04	0.06	0.68	0.55
CR_06180W_A	0.49	0	0	0.69	0	0
C5_03050C_A	1.28	0	0	1.55	0	0
C2_05220C_A	0	0.99	0.98	0.05	0.73	0.62
C6_01610W_A	0.05	0.44	0.28	-0.18	0.04	0.02
C3_02000W_A	0.2	0.14	0.06	0.4	0	0
C1_04310C_A	0.03	0.94	0.89	0.28	0.31	0.2
C2_08390W_A	0.57	0	0	0.48	0	0
C2_07090C_A	-0.25	0.03	0.01	-0.57	0	0
C1_04020C_A	0.66	0	0	2.11	0	0
C2_01350C_A	0.23	0.14	0.06	0.45	0	0
C4_04650W_A	-0.12	0.64	0.48	-0.29	0.21	0.12
C5_03070W_A	-0.14	0.06	0.02	-0.54	0	0
C1_11900C_A	0	0.99	0.98	0.17	0.42	0.28
C1_05450W_A	NA	NA	NA	NA	NA	NA
C2_08340C_A	0.17	0.69	0.55	0.67	0.28	0.17
C1_00330C_A	0.23	0.06	0.02	0.09	0.58	0.44
C2_09930W_A	0.14	0.28	0.15	0.22	0.06	0.03
C6_01640W_A	-0.15	0.42	0.26	0.03	0.88	0.82
C1_05180C_A	0.03	0.95	0.9	0.34	0.32	0.2
C4_02710C_A	-0.2	0.03	0.01	-0.46	0	0
C2_01090C_A	0.03	0.88	0.8	-0.36	0.02	0.01
C6_02760W_A	-0.49	0.3	0.17	-0.11	0.88	0.82
C1_13990W_A	0.11	0.66	0.5	0.02	0.93	0.88
C1_03930W_A	0.05	0.79	0.67	-0.09	0.6	0.46
CR_05750W_A	0.66	0	0	1.11	0	0
C4_06090C_A	-0.05	0.82	0.71	-0.2	0.33	0.21
C2_10470C_A	0.04	0.78	0.65	0.18	0.17	0.09
C7_02160W_A	-0.4	0.07	0.02	0.3	0.18	0.1
C3_01500C_A	0.07	0.62	0.46	0.32	0	0
C5_04750C_A	-0.73	0	0	-1.58	0	0
CR_09120C_A	0.09	0.39	0.24	-0.01	0.96	0.92
C5_03900W_A	0.33	0	0	0.12	0.29	0.18
C4_01700C_A	-0.12	0.22	0.11	-0.49	0	0
C2_07470W_A	0.16	0.25	0.13	0.14	0.29	0.18
C4_00180W_A	0.12	0.5	0.33	-0.38	0.01	0
C2_08110W_A	-0.43	0	0	-0.38	0.01	0
C6_01350W_A	-0.66	0	0	-0.06	0.91	0.85
C4_06010C_A	0.21	0.39	0.24	0.17	0.52	0.38

C1_00870W_A	-0.2	0.1	0.04	-0.31	0.01	0
C1_03190C_A	-0.2	0	0	-1.29	0	0
C5_00980W_A	-0.14	0.32	0.18	-0.34	0.01	0
C5_04190W_A	1.64	0	0	1.67	0	0
C3_01360C_A	0.21	0.04	0.02	-0.08	0.58	0.44
C2_00940W_A	1	0	0	1.66	0	0
CR_10710C_A	0.23	0.08	0.03	0.35	0.01	0
C4_06800W_A	-0.32	0.01	0	-0.33	0.01	0
CR_02920C_A	-1.55	0	0	-3.28	0	0
C6_02160W_A	-0.28	0.18	0.08	0.15	0.54	0.4
CR_00860C_A	0.09	0.25	0.13	-0.23	0	0
C1_07240W_A	-0.13	0.39	0.23	0.27	0.04	0.02
C1_11660W_A	-0.48	0	0	-0.18	0.03	0.01
C2_09220W_A	1.55	0	0	3.35	0	0
C4_05110C_A	-0.29	0.08	0.03	-0.18	0.3	0.19
C1_03890W_A	0.07	0.67	0.51	NA	NA	NA
C3_03230C_A	-0.52	0	0	-1.13	0	0
C5_01140C_A	0.12	0.24	0.12	-0.01	0.96	0.93
C3_06680C_A	0.65	0.01	0	1.28	0	0
CR_02030C_A	-0.53	0.04	0.01	-0.27	0.32	0.2
C1_05750C_A	-0.06	0.67	0.51	0.17	0.22	0.12
C1_02830W_A	-0.38	0.02	0	-0.12	0.44	0.31
C2_03680W_A	0.52	0.01	0	0.23	0.36	0.24
C3_05930W_A	0.04	0.77	0.64	-0.06	0.62	0.49
C4_01720C_A	-0.21	0.06	0.02	-0.21	0.12	0.06
C1_07950C_A	-0.48	0.18	0.08	-0.18	0.67	0.55
C1_08410C_A	0.2	0.01	0	-0.32	0	0
C1_04740W_A	-0.02	0.93	0.87	0.35	0	0
C5_00200C_A	0.1	0.29	0.16	0.04	0.74	0.63
C1_06730W_A	-0.12	0.47	0.31	-0.06	0.76	0.66
C7_00110W_A	0.43	0.23	0.12	0.98	0	0
C1_00360W_A	0.82	0	0	1.33	0	0
C5_04930C_A	0.35	0.44	0.28	0.66	0.14	0.07
C7_00850W_A	-0.03	0.88	0.8	0.11	0.59	0.45
C7_03050W_A	0.11	0.42	0.26	0.16	0.25	0.15
C1_06070W_A	-0.41	0	0	-0.39	0	0
C1_13970C_A	0.07	0.72	0.58	0.09	0.6	0.46
C3_01230C_A	0.05	0.94	0.9	0.25	0.68	0.56
C2_05340C_A	0.05	0.76	0.63	-0.3	0.03	0.01
C3_01390C_A	0.2	0.1	0.04	0.55	0	0
C7_01330C_A	0.14	0.5	0.33	0.42	0.01	0
C3_05170W_A	0.7	0	0	0.69	0	0
C1_04370C_A	-0.2	0.02	0.01	-0.4	0	0

C2_04430W_A	-0.01	0.97	0.94	0.3	0.62	0.49
C1_07840W_A	0.56	0	0	0.57	0	0
C6_00100C_A	1.25	0	0	1.65	0	0
CR_09440C_A	-0.22	0.11	0.05	-0.01	0.96	0.94
C3_05830W_A	0.66	0	0	0.88	0	0
CR_06070W_A	0.17	0.06	0.02	-0.15	0.23	0.14
C3_02900W_A	0.33	0.03	0.01	-0.14	0.45	0.31
C7_02940C_A	-0.29	0	0	-0.41	0	0
C5_03760C_A	-0.13	0.24	0.12	0.09	0.49	0.35
C6_03650C_A	-0.01	0.97	0.94	0.08	0.49	0.35
C3_05990C_A	0.03	0.91	0.85	0	0.99	0.98
C2_05470W_A	0.05	0.62	0.46	-0.09	0.43	0.3
C7_03110W_A	-0.04	0.95	0.91	-0.07	0.91	0.86
C5_00920W_A	-0.28	0.25	0.13	-0.21	0.4	0.27
C2_05090W_A	-1.11	0	0	-0.86	0	0
C2_09270C_A	0.28	0	0	0.56	0	0
CR_05360C_A	-0.16	0.36	0.21	-0.01	0.97	0.95
C1_02080W_A	-0.45	0.11	0.04	-0.43	0.15	0.08
C1_10980W_A	0.51	0.28	0.15	1.07	0.06	0.03
CR_05480W_A	0.53	0	0	1.02	0	0
C4_00400W_A	0.06	0.65	0.49	0.14	0.22	0.12
C7_03780C_A	-0.32	0.22	0.11	0.36	0.2	0.11
C2_07360W_A	-0.25	0.22	0.11	0.06	0.81	0.72
C4_06760W_A	0.8	0	0	0.74	0	0
CR_03730C_A	0.55	0	0	0.26	0.15	0.08
C2_09280C_A	0.22	0.29	0.16	0.07	0.8	0.71
CR_03500W_A	-0.16	0.09	0.04	-0.35	0	0
C1_03600W_A	0.74	0	0	0.72	0	0
CR_00190W_A	-0.18	0.19	0.09	-0.11	0.46	0.33
C1_02120C_A	-0.1	0.29	0.16	0.12	0.16	0.08
C4_00710W_A	0.62	0	0	0.95	0	0
C1_12730W_A	-0.85	0	0	-1.05	0	0
C2_09510C_A	0.07	0.7	0.56	0.37	0.01	0
C5_02180C_A	-0.04	0.77	0.65	-0.05	0.69	0.57
C1_07170C_A	-0.2	0.37	0.22	-0.21	0.33	0.21
C1_06680W_A	0.06	0.73	0.59	0.12	0.46	0.32
C5_02780W_A	-0.39	0	0	-0.37	0.01	0
C4_06550C_A	-0.15	0.36	0.21	-0.17	0.28	0.17
C6_00260W_A	0.36	0	0	0.42	0	0
C2_06800C_A	-0.55	0.01	0	-0.34	0.11	0.05
C4_04710W_A	0.19	0.09	0.04	-0.07	0.64	0.51
C3_03320W_A	-0.31	0.28	0.15	0.05	0.87	0.81
C3_02660W_A	0.28	0.58	0.41	0.37	0.59	0.45

C1_10540C_A	0.07	0.75	0.61	-0.09	0.65	0.52
C1_12230W_A	-0.22	0.04	0.01	-0.13	0.38	0.25
C4_05860W_A	-0.16	0.27	0.14	-0.21	0.13	0.07
C1_09940W_A	-0.1	0.85	0.75	0.21	0.66	0.53
CR_06990W_A	0.18	0.72	0.58	-0.13	0.84	0.77
C4_02490W_A	0.11	0.65	0.49	0.19	0.35	0.23
C6_01860C_A	-0.09	0.58	0.42	-0.17	0.25	0.14
CR_00820C_A	-0.05	0.78	0.66	-0.04	0.85	0.78
CR_08040W_A	0.24	0.04	0.01	0.22	0.11	0.06
CR_02980C_A	-0.76	0	0	-0.82	0	0
C1_02280C_A	-0.1	0.64	0.48	0.13	0.56	0.43
CR_07100W_A	0.75	0	0	0.85	0	0
C3_05750C_A	-0.05	0.93	0.87	0.41	0.32	0.2
C2_00260C_A	0.36	0	0	0.5	0	0
C3_03780W_A	-0.15	0.61	0.45	-0.16	0.55	0.41
C6_03820C_A	0.02	0.92	0.86	0.13	0.34	0.22
CR_06530W_A	-0.06	0.74	0.61	0.08	0.63	0.5
C6_03550C_A	-0.49	0	0	-0.54	0.01	0
CR_01510C_A	NA	NA	NA	NA	NA	NA
C4_02630C_A	0.39	0.13	0.05	0.59	0.01	0
C4_02120W_A	1.1	0	0	1.27	0	0
C2_03990W_A	-0.04	0.79	0.67	-0.01	0.93	0.89
C4_03030C_A	-0.53	0	0	-0.29	0.04	0.02
C1_08140W_A	0.02	0.95	0.9	-0.02	0.95	0.92
C5_01980C_A	0.09	0.57	0.41	0.24	0.08	0.04
C3_02460C_A	-0.23	0.24	0.13	-0.14	0.45	0.31
C1_10800C_A	-0.11	0.23	0.12	-0.75	0	0
C4_03830W_A	-0.89	0	0	-0.95	0	0
C3_01930W_A	-0.05	0.93	0.87	0.17	0.69	0.58
C1_12610W_A	-0.29	0.01	0	-0.42	0	0
C4_06920C_A	NA	NA	NA	NA	NA	NA
C1_02460W_A	0.04	0.63	0.47	-0.37	0	0
C1_14090W_A	-0.08	0.33	0.19	-0.08	0.52	0.38
C3_06120C_A	-0.15	0.14	0.06	-0.3	0.01	0
C4_06080W_A	-0.14	0.81	0.7	0	1	1
C4_06500W_A	-0.25	0.01	0	-0.07	0.65	0.52
C2_07910C_A	-0.1	0.84	0.74	-0.12	0.82	0.74
CR_04890W_A	-0.21	0.48	0.31	-0.46	0.08	0.04
C1_04060W_A	0.28	0.27	0.15	0.32	0.18	0.1
C2_08990C_A	0.08	0.63	0.47	0.15	0.33	0.21
C4_02570C_A	0.08	0.68	0.54	0.05	0.82	0.73
C1_06120C_A	0.14	0.76	0.63	-0.23	0.64	0.5
C2_01620W_A	0.12	0.32	0.18	0.16	0.19	0.1

CR_01530C_A	-0.01	0.97	0.94	0.1	0.87	0.81
C1_01190C_A	0.39	0	0	0.3	0.03	0.01
C2_03340W_A	0.19	0.09	0.04	0.35	0	0
C7_02990W_A	0.09	0.65	0.49	0.13	0.49	0.35
C4_00010W_A	0.07	0.61	0.44	-0.03	0.82	0.74
C7_01900W_A	0.39	0	0	0.48	0	0
C3_04380C_A	-0.17	0.29	0.16	-0.89	0	0
C5_01620C_A	-0.07	0.81	0.7	-0.04	0.9	0.84
C3_06210C_A	-0.09	0.63	0.47	0.27	0.07	0.03
C3_07710W_A	0.09	0.59	0.43	0.19	0.19	0.11
C2_08800C_A	-0.28	0	0	-0.68	0	0
C2_07450C_A	-0.62	0	0	-0.91	0	0
C2_03060W_A	0.17	0.03	0.01	0.34	0	0
C1_14550C_A	-0.14	0.75	0.61	0.2	0.61	0.48
C2_04690C_A	-0.06	0.83	0.73	0.22	0.3	0.19
C1_03810C_A	-0.27	0.37	0.22	0.1	0.74	0.63
C7_03270W_A	-0.33	0.03	0.01	-0.18	0.26	0.16
C1_11240C_A	0	0.99	0.97	0.45	0	0
C1_09500W_A	0.01	0.99	0.98	-0.42	0.33	0.21
C5_02370C_A	-0.09	0.65	0.49	0.21	0.2	0.11
CR_01390W_A	-0.2	0.03	0.01	-0.53	0	0
C4_01760W_A	0.2	0.04	0.02	-0.24	0.03	0.01
CR_00670C_A	-0.06	0.83	0.72	0.17	0.49	0.35
CR_04960C_A	0.52	0	0	0.58	0	0
C4_04400C_A	NA	NA	NA	NA	NA	NA
C5_04510W_A	0.41	0	0	0.5	0	0
C6_01550C_A	-0.53	0	0	-0.88	0	0
CR_03980W_A	-0.19	0.43	0.27	-0.05	0.86	0.8
CR_00290W_A	0.46	0	0	2.08	0	0
C1_00020C_A	0.67	0	0	1.25	0	0
C1_00270W_A	-0.01	0.99	0.98	0.14	0.82	0.74
C7_00680W_A	0.42	0.11	0.05	0.54	0.02	0.01
CR_00760C_A	-0.01	0.98	0.95	-0.24	0.13	0.07
C1_01820C_A	0.02	0.94	0.88	0.1	0.62	0.49
CR_10790W_A	0.29	0.24	0.13	0.56	0.01	0
C3_03010C_A	0.47	0	0	0.52	0	0
CR_01560W_A	-0.41	0.04	0.02	-0.01	0.97	0.94
C1_11490C_A	-0.05	0.92	0.85	0.39	0.57	0.43
C6_02370C_A	-0.32	0.01	0	-0.4	0	0
C5_02230W_A	0.28	0	0	0.35	0	0
C2_08880W_A	-0.07	0.82	0.72	-0.27	0.47	0.33
CR_10620C_A	0.26	0.02	0.01	0.66	0	0
CR_00540C_A	0.11	0.38	0.23	0.23	0.04	0.02

C5_05320C_A	0.24	0	0	0.08	0.45	0.32
C7_01280C_A	-0.25	0.19	0.09	-0.09	0.67	0.55
C1_10290W_A	-0.15	0.47	0.31	-2.24	0	0
C2_09130C_A	0.21	0.39	0.24	0.35	0.1	0.05
C1_06870C_A	-0.71	0.04	0.02	-1.36	0	0
C2_10700C_A	-0.02	0.93	0.88	0.08	0.58	0.45
CR_05150W_A	-0.28	0.1	0.04	-0.45	0.01	0
CR_03880W_A	0.04	0.81	0.7	-0.2	0.12	0.06
C1_05070C_A	0.07	0.75	0.62	-0.13	0.54	0.4
C1_02450C_A	-0.66	0.03	0.01	-0.5	0.09	0.04
CR_07130C_A	-0.33	0.52	0.36	-0.17	0.8	0.71
C3_01490W_A	-0.18	0	0	-0.76	0	0
C7_00020C_A	0.26	0	0	0.5	0	0
C5_05010W_A	-0.39	0.4	0.24	-0.51	0.31	0.19
C7_01810W_A	0.06	0.53	0.36	-0.07	0.49	0.35
C3_06170C_A	NA	NA	NA	NA	NA	NA
C3_03650W_A	-1.15	0	0	-0.42	0.04	0.02
CR_10760C_A	-0.16	0.42	0.26	-0.45	0.02	0.01
CR_04800W_A	-0.03	0.92	0.85	0.39	0.04	0.02
CR_09830W_A	-0.01	0.96	0.92	-0.04	0.85	0.78
CR_00740C_A	2.49	0	0	3.56	0	0
C7_03300C_A	-0.45	0	0	-0.07	0.67	0.54
C6_01370W_A	-0.02	0.9	0.83	-0.05	0.73	0.62
C5_01430C_A	-0.4	0.04	0.01	-0.31	0.11	0.05
C4_06880C_A	-0.1	0.57	0.4	0.24	0.11	0.05
CR_09050C_A	-0.35	0.15	0.07	-0.63	0.01	0
CR_01430W_A	-1.25	0	0	-1.3	0	0
C3_02930W_A	0.08	0.89	0.81	-0.16	0.79	0.7
CR_04590C_A	0.02	0.82	0.72	-0.15	0.1	0.05
C2_05290C_A	0.56	0	0	0.58	0	0
C2_07300C_A	-0.58	0.04	0.01	-0.01	0.99	0.98
C4_06890W_A	0.17	0.55	0.39	-0.04	0.9	0.85
C5_03990W_A	0.2	0.22	0.11	-0.17	0.33	0.21
CR_01910C_A	0.56	0.22	0.11	0.54	0.35	0.23
C1_01580W_A	-0.23	0.03	0.01	-0.43	0	0
C7_02470C_A	0.25	0.03	0.01	0.13	0.38	0.25
C7_03330C_A	-0.02	0.85	0.76	-0.2	0.03	0.01
C2_09320C_A	-0.77	0	0	-1.43	0	0
C3_03470W_A	0.01	0.98	0.96	-0.09	0.81	0.73
C1_11070W_A	-0.49	0.01	0	-0.36	0.03	0.01
C3_06950W_A	-0.55	0.09	0.04	-0.35	0.32	0.21
C7_01070C_A	-0.21	0.19	0.09	-0.1	0.6	0.46
C3_07850W_A	0.43	0	0	0.12	0.46	0.32

CR_06610W_A	-0.16	0.78	0.66	0.08	0.9	0.85
C6_03600C_A	0.13	0.67	0.52	0.34	0.53	0.39
C4_07210W_A	0.51	0	0	0.58	0	0
CR_09180W_A	-0.19	0.67	0.51	0.36	0.35	0.22
C6_03110C_A	-0.48	0.06	0.02	-0.12	0.69	0.57
C3_04270C_A	0.09	0.61	0.45	-0.24	0.15	0.08
CR_02590C_A	-0.02	0.95	0.91	0.24	0.2	0.12
CR_09760W_A	-0.27	0.23	0.12	-0.18	0.44	0.31
CR_00520C_A	-0.02	0.9	0.83	-0.18	0.07	0.03
C4_00720W_A	0.37	0.03	0.01	0.22	0.29	0.18
C5_04670W_A	0.1	0.42	0.26	0.13	0.27	0.16
C5_02020C_A	0.09	0.61	0.44	-0.05	0.8	0.7
C7_02830C_A	-0.02	0.94	0.9	-0.02	0.94	0.9
C2_08900W_A	0.39	0.26	0.13	0.62	0.06	0.03
C2_02220C_A	-0.06	0.87	0.79	0.87	0.15	0.08
C5_00670C_A	-0.41	0	0	-0.27	0.13	0.07
C5_02060W_A	-0.39	0.04	0.02	-0.47	0.02	0.01
C1_03260W_A	-0.19	0.32	0.18	0.05	0.81	0.73
CR_04790W_A	0.16	0.59	0.42	0.68	0	0
C5_04050W_A	0.36	0	0	0.52	0	0
C3_04230W_A	-0.31	0.15	0.07	-0.29	0.18	0.1
C3_00120W_A	NA	NA	NA	NA	NA	NA
CR_10330W_A	0.09	0.87	0.79	0.1	0.86	0.8
C7_00560C_A	-0.1	0.72	0.58	0.02	0.94	0.9
C2_04670W_A	NA	NA	NA	0.12	0.67	0.55
C1_04900W_A	1.15	0	0	2.34	0	0
C3_07920W_A	0.33	0	0	0.26	0.02	0.01
C4_00970C_A	-0.71	0	0	-1.48	0	0
C4_01450W_A	-0.84	0	0	-1.25	0	0
C5_00370W_A	0.43	0	0	0.51	0	0
C6_02830W_A	-0.2	0.55	0.39	-0.34	0.25	0.15
C1_13380W_A	-0.55	0.1	0.04	0.19	0.61	0.47
C1_11350C_A	0.19	0.03	0.01	-0.11	0.29	0.18
C1_09280W_A	-0.17	0.35	0.2	0.02	0.93	0.88
C2_03050W_A	0.09	0.65	0.49	0.2	0.22	0.13
C1_11220C_A	-0.16	0.04	0.01	-0.45	0	0
C2_02040W_A	-0.09	0.6	0.43	0.1	0.58	0.44
C3_05660C_A	-0.06	0.9	0.83	-0.34	0.51	0.37
C2_03310C_A	0.07	0.74	0.6	0.3	0.07	0.03
C7_01160C_A	0.09	0.61	0.44	0.12	0.51	0.37
C3_07460W_A	-0.38	0.01	0	-0.34	0.02	0.01
C7_04330C_A	0.61	0	0	0.71	0	0
C1_11860W_A	0.72	0	0	0.63	0	0

CR_02340W_A	0.11	0.73	0.6	-0.12	0.69	0.57
CR_08680C_A	0.16	0.28	0.15	0.29	0.03	0.01
C1_11690W_A	-0.25	0.36	0.21	-0.35	0.21	0.12
C6_03360C_A	0.37	0.39	0.23	0.09	0.86	0.79
C4_02080W_A	0.13	0.76	0.64	0.53	0.13	0.07
C7_00530C_A	-0.18	0.49	0.32	0.2	0.45	0.32
C1_06160W_A	-0.37	0.46	0.29	-0.02	0.99	0.97
C1_05230W_A	0.04	0.92	0.85	-0.26	0.38	0.25
C6_02860W_A	0.37	0	0	0.28	0.02	0.01
C1_07110W_A	-0.3	0.01	0	-0.26	0.09	0.05
C4_06260W_A	-0.09	0.81	0.7	-0.28	0.42	0.29
C1_02530C_A	-0.49	0	0	-1.47	0	0
C6_00070C_A	-0.06	0.79	0.67	-0.3	0.11	0.05
C1_12090C_A	0.3	0.54	0.37	0.06	0.94	0.9
C3_03190C_A	0.27	0.04	0.01	0.15	0.33	0.22
C2_04470W_A	-0.15	0.79	0.67	-0.13	0.82	0.75
C2_01910W_A	0.12	0.74	0.61	0.11	0.76	0.66
C2_10450W_A	0.28	0.07	0.03	0.2	0.27	0.17
C5_02480W_A	-0.12	0.37	0.22	0.09	0.52	0.38
C1_09880C_A	-0.17	0.52	0.35	0.42	0.04	0.02
C1_09310C_A	0.21	0.31	0.18	0.36	0.06	0.02
C5_02980C_A	-0.16	0.57	0.41	-0.04	0.91	0.86
C5_01250W_A	-0.13	0.56	0.4	0.22	0.24	0.14
C3_07070C_A	0.21	0.66	0.5	1.41	0	0
C6_02950C_A	-0.02	0.97	0.94	-0.35	0.32	0.21
C4_00200C_A	-0.75	0	0	-0.91	0	0
C5_00700C_A	-0.19	0.25	0.13	0.44	0	0
C7_02220C_A	-0.17	0.58	0.41	-0.44	0.1	0.05
C3_01940C_A	0.47	0	0	0.45	0	0
C4_02960W_A	0.2	0.13	0.06	-0.67	0	0
C2_07330W_A	0.26	0	0	0.21	0.02	0.01
C6_02410W_A	0.21	0.06	0.02	0.1	0.41	0.28
CR_05980W_A	-0.06	0.61	0.45	0	1	0.99
C1_10020W_A	0.42	0	0	0.52	0	0
C7_03370C_A	0.16	0.12	0.05	0.25	0.01	0
C2_02760W_A	0.52	0	0	0.12	0.36	0.24
CR_05140W_A	0.72	0	0	1.33	0	0
C4_04560C_A	0.06	0.64	0.48	0.33	0	0
C1_03280W_A	0.01	0.96	0.93	-0.11	0.29	0.18
C4_01380W_A	-0.31	0.01	0	-0.29	0.05	0.02
C2_07630C_A	-0.89	0	0	-0.39	0	0
CR_08100C_A	-0.48	0	0	-0.62	0	0
C3_02580C_A	0.11	0.77	0.64	0.16	0.65	0.52

C6_02600W_A	0.06	0.57	0.41	0.17	0.09	0.04
C1_00750C_A	0.14	0.24	0.13	0.46	0	0
C1_03870C_A	-1.42	0	0	-1.69	0	0
C3_04670C_A	-0.1	0.28	0.15	-0.55	0	0
CR_06170W_A	0.19	0.17	0.08	0.17	0.21	0.12
C1_01030W_A	-0.25	0.01	0	-0.86	0	0
C1_05050C_A	-0.67	0	0	-0.6	0	0
C1_07350C_A	0.3	0.01	0	0.41	0	0
C4_04470W_A	0.91	0	0	1.1	0	0
C1_12530C_A	-0.29	0.12	0.05	-0.22	0.25	0.15
C1_02720W_A	-0.32	0.08	0.03	-0.22	0.29	0.18
C3_05460W_A	-0.19	0.29	0.16	-0.12	0.54	0.4
C4_04520W_A	-0.68	0	0	0.25	0.27	0.16
C7_00510W_A	-0.17	0.4	0.24	-0.68	0	0
C1_12480W_A	-0.01	0.99	0.98	-0.13	0.79	0.69
CR_00080W_A	0.13	0.28	0.15	0.28	0.02	0.01
C3_03290C_A	-0.03	0.94	0.89	-0.29	0.24	0.14
C2_04300C_A	-0.08	0.66	0.5	-0.24	0.13	0.07
C6_00410C_A	0.42	0	0	0.45	0	0
CR_10310W_A	-0.11	0.86	0.76	0.01	0.99	0.98
C1_10140C_A	0.56	0	0	0.67	0	0
C2_01730W_A	0.08	0.86	0.77	0.01	0.98	0.96
C2_06810C_A	-0.14	0.03	0.01	-0.61	0	0
C1_04760C_A	0.24	0.02	0.01	-0.17	0.19	0.11
C3_07080W_A	-0.09	0.66	0.51	-0.03	0.89	0.84
CR_03200C_A	-0.13	0.44	0.28	-0.05	0.78	0.68
C4_03670W_A	-0.34	0.03	0.01	-0.17	0.34	0.22
CR_00340C_A	-0.49	0.28	0.15	-0.52	0.43	0.29
C1_07870C_A	-0.13	0.13	0.06	-1.47	0	0
C7_01380W_A	0.2	0.61	0.45	0.77	0.01	0
C3_03280C_A	0.12	0.3	0.16	-0.24	0.06	0.02
CR_04570C_A	0.56	0	0	0.68	0	0
C6_01140C_A	0.19	0.21	0.11	0.35	0.01	0
C2_01870C_A	0	1	1	0.25	0.39	0.26
C1_06800W_A	-0.12	0.57	0.41	0.2	0.27	0.16
C5_03690W_A	0.2	0.48	0.32	0.61	0.01	0
C2_02980C_A	-0.36	0	0	-0.58	0	0
C1_04380W_A	0.13	0.5	0.34	0	0.99	0.98
C1_01920W_A	-0.03	0.9	0.82	-0.05	0.78	0.68
CR_06700C_A	-0.07	0.5	0.33	-0.06	0.68	0.55
CR_10230W_A	0.36	0	0	0.49	0	0
C2_06180C_A	-0.07	0.83	0.73	-0.22	0.65	0.52
CR_05740C_A	-0.14	0.48	0.31	-0.16	0.37	0.24

C5_03980W_A	-0.22	0.15	0.07	-0.39	0.01	0
C2_01250W_A	0.14	0.28	0.15	0.38	0	0
CR_02640W_A	-0.2	0.14	0.06	-0.35	0.01	0
C1_07140C_A	-0.15	0.28	0.15	-0.16	0.25	0.15
CR_06820W_A	-0.41	0.03	0.01	-0.62	0	0
C1_05420W_A	-0.04	0.86	0.77	0.04	0.86	0.79
C6_04040C_A	0.3	0	0	0.47	0	0
C2_05500W_A	0.61	0	0	0.68	0	0
CR_03280W_A	0.21	0.01	0	0.08	0.44	0.31
C4_05310W_A	-0.32	0.04	0.01	-0.38	0.02	0.01
C2_08910C_A	0.07	0.68	0.53	0.41	0	0
CR_10370W_A	0.03	0.77	0.65	0.18	0.04	0.02
CR_04070W_A	-0.32	0	0	-0.42	0	0
C3_07760C_A	0.7	0.01	0	1.26	0	0
C6_00780W_A	0.25	0.02	0	0.32	0	0
C2_04760W_A	0.13	0.31	0.17	0.28	0.01	0
C2_05250C_A	-0.16	0.02	0.01	-0.3	0	0
C5_05110C_A	0.16	0.16	0.07	0.32	0.01	0
C7_01210C_A	-0.23	0.24	0.12	0.05	0.82	0.74
C5_01100C_A	0.65	0	0	1.17	0	0
C2_08310W_A	-0.24	0.11	0.04	-0.79	0	0
C5_00350C_A	-0.06	0.66	0.51	-0.11	0.37	0.25
C6_02670C_A	0.34	0.01	0	0.91	0	0
C6_02920C_A	-0.04	0.8	0.68	0.09	0.53	0.39
C3_05530W_A	0.83	0	0	0.47	0	0
C1_12210W_A	-0.17	0.19	0.09	-0.06	0.71	0.59
C4_06400C_A	-0.07	0.58	0.41	0.03	0.81	0.72
C5_03380W_A	-0.01	0.98	0.95	0.31	0.13	0.07
C2_03300W_A	0.26	0.01	0	0.33	0.01	0
CR_08870W_A	-0.06	0.75	0.62	-0.04	0.81	0.73
C1_13790C_A	-0.06	0.8	0.69	0.08	0.68	0.56
C6_04440C_A	0.08	0.72	0.58	0.08	0.69	0.57
CR_00720W_A	NA	NA	NA	NA	NA	NA
C7_03240W_A	0.3	0	0	0.29	0	0
CR_03480W_A	0.57	0.15	0.07	0.71	0.09	0.04
C4_06650W_A	-0.04	0.78	0.65	0.18	0.29	0.18
C1_09060C_A	-0.88	0	0	-0.66	0	0
CR_02650C_A	-0.69	0	0	-0.29	0.25	0.15
C1_12840W_A	-0.47	0.18	0.08	0.26	0.5	0.36
C1_03840W_A	-0.56	0.1	0.04	-0.56	0.11	0.05
C2_09010W_A	-0.16	0.38	0.22	-0.17	0.37	0.24
C3_04290C_A	-0.05	0.73	0.59	-0.06	0.65	0.52
C1_05080W_A	0.16	0.68	0.54	0.09	0.83	0.76

C4_01530C_A	-0.04	0.63	0.47	-0.44	0	0
C1_00460W_A	-0.02	0.88	0.81	-0.01	0.94	0.91
C3_06520C_A	-0.1	0.71	0.56	0.17	0.48	0.35
C4_03770W_A	-0.04	0.88	0.79	-0.01	0.97	0.94
C3_01220W_A	0.13	0.58	0.42	0.32	0.07	0.03
CR_05660W_A	-1.44	0	0	-1.86	0	0
C2_04950C_A	0.4	0	0	0.35	0	0
C1_01080W_A	-0.09	0.68	0.54	0.07	0.76	0.66
C1_14200W_A	0.04	0.86	0.77	-0.01	0.95	0.92
C4_03290W_A	-0.15	0.11	0.05	0.01	0.96	0.93
C3_02150C_A	-0.78	0	0	-1.5	0	0
C2_08280W_A	-0.31	0.1	0.04	-0.2	0.38	0.26
C2_07410W_A	0.12	0.61	0.44	0.19	0.34	0.22
C3_05440C_A	-0.61	0	0	-0.3	0.21	0.12
C2_08730W_A	-0.4	0.01	0	-0.22	0.28	0.17
C2_01070W_A	-0.37	0	0	-0.7	0	0
C5_02080C_A	-1.78	0	0	-1.92	0	0
C4_03140C_A	0	1	0.99	0.06	0.82	0.74
C3_00430W_A	0.12	0.79	0.67	-0.19	0.69	0.58
C1_13070C_A	-0.35	0	0	-0.52	0	0
C4_02260C_A	-0.68	0	0	-0.43	0.01	0
C1_03440C_A	0.15	0.17	0.08	0.08	0.54	0.41
C6_02750C_A	-0.01	0.98	0.96	0.17	0.55	0.41
C3_01750C_A	0.05	0.72	0.58	0.21	0.05	0.02
C3_01990W_A	0.11	0.26	0.14	0.05	0.68	0.55
C4_00060W_A	0.11	0.47	0.3	-0.05	0.8	0.71
C1_06930W_A	0.97	0	0	0.96	0	0
C5_01490C_A	0.06	0.78	0.66	0.17	0.39	0.26
C1_04890W_A	0.19	0	0	0.07	0.43	0.3
C3_02270W_A	0.01	0.98	0.97	0.52	0.2	0.12
C2_07340W_A	-0.85	0	0	-0.56	0	0
C4_04980W_A	1.13	0	0	1.62	0	0
C2_05990C_A	0.54	0.17	0.08	0.85	0.03	0.01
C1_00670C_A	-0.22	0.22	0.11	-0.21	0.26	0.15
C5_00740W_A	-0.22	0.47	0.31	-0.27	0.37	0.24
C2_06690C_A	-0.39	0.06	0.02	0.07	0.77	0.67
C1_05840W_A	-0.4	0.12	0.05	-0.65	0.01	0
C7_00180W_A	-0.29	0.49	0.33	0.16	0.72	0.61
C7_04160W_A	-0.1	0.72	0.58	-0.56	0.03	0.01
C1_04600C_A	-0.04	0.65	0.49	-0.24	0	0
CR_05860W_A	0.56	0	0	0.69	0	0
C5_00260W_A	-0.44	0	0	-0.92	0	0
CR_03440W_A	0.01	0.99	0.98	-0.06	0.91	0.86

C2_03830W_A	-0.2	0.11	0.05	-0.06	0.7	0.58
C3_07140C_A	-0.45	0.06	0.02	-0.41	0.05	0.02
C1_05650W_A	0.12	0.49	0.33	0.14	0.4	0.27
C5_00070W_A	0.88	0	0	1.17	0	0
CR_02960W_A	0.44	0.04	0.01	0.1	0.7	0.58
C7_00790W_A	-0.21	0.25	0.13	-0.09	0.69	0.57
C4_05530W_A	0.46	0.01	0	1.09	0	0
C4_05390W_A	0	1	1	-0.18	0.08	0.04
C5_02860C_A	0.02	0.88	0.79	0.26	0	0
C3_06530W_A	0.09	0.72	0.58	0.35	0.08	0.04
C3_03510C_A	-0.13	0.35	0.21	0.04	0.82	0.74
C4_00880W_A	0.1	0.53	0.36	0.05	0.77	0.67
C7_01710W_A	0.25	0	0	-0.1	0.38	0.26
C2_06880C_A	-0.04	0.78	0.66	-1.83	0	0
C1_04690C_A	0.12	0.78	0.66	0.07	0.92	0.87
C3_05030W_A	0.04	0.77	0.64	0.2	0.04	0.02
C6_01850W_A	-0.3	0.01	0	-0.05	0.78	0.68
C6_02300C_A	-0.15	0.75	0.61	-0.22	0.62	0.48
C1_10560C_A	0.67	0	0	0.4	0.01	0
C5_01900C_A	0.88	0.04	0.01	1.27	0.02	0.01
C1_04450C_A	0.09	0.38	0.23	-0.1	0.42	0.29
CR_05260C_A	0.15	0.49	0.32	0.15	0.46	0.33
C3_02320W_A	0.06	0.49	0.32	-0.08	0.43	0.3
C2_06530W_A	-0.37	0.12	0.05	-0.17	0.51	0.37
C6_00990W_A	-0.01	0.96	0.92	-0.26	0.14	0.07
C4_03090W_A	-0.19	0.31	0.17	0.16	0.36	0.24
C7_04310C_A	0.81	0	0	1.1	0	0
CR_01920W_A	0.38	0.44	0.28	0.82	0.12	0.06
C1_02610W_A	-0.46	0	0	-0.46	0	0
CR_04030W_A	0.16	0.38	0.23	0.16	0.42	0.29
C4_07070W_A	-0.07	0.77	0.64	0.01	0.96	0.93
C3_04820C_A	0.52	0	0	0.63	0	0
C6_00530C_A	-0.29	0.29	0.16	0.04	0.91	0.86
C2_05660W_A	0.38	0	0	0.01	0.91	0.85
C5_01360W_A	-0.07	0.68	0.53	0	1	0.99
C1_06520C_A	0.19	0.1	0.04	0.09	0.54	0.41
C5_02580W_A	-0.15	0.41	0.25	0.11	0.58	0.45
C4_06990W_A	-0.06	0.85	0.76	0.78	0	0
C2_02020W_A	0.49	0	0	0.59	0	0
C6_02210W_A	0.99	0	0	1.82	0	0
C2_00820W_A	-0.33	0.08	0.03	-0.05	0.81	0.73
C6_02130C_A	-0.05	0.88	0.79	-0.44	0.05	0.02
C4_04820C_A	-0.23	0.03	0.01	-0.31	0	0

C1_07070C_A	0.17	0.36	0.21	0.15	0.46	0.33
C1_01760W_A	0.52	0	0	0.57	0	0
CR_01000C_A	-0.12	0.41	0.26	-0.01	0.95	0.92
C1_00940W_A	-0.05	0.82	0.71	-0.1	0.56	0.43
C1_13520C_A	-0.15	0.5	0.34	0.04	0.87	0.81
C4_01120C_A	-0.22	0.33	0.19	-0.08	0.74	0.63
C1_04650W_A	0.76	0.07	0.02	0.53	0.32	0.2
C6_02490C_A	-0.05	0.76	0.63	0.52	0	0
C1_12100C_A	0.16	0.55	0.39	0.01	0.98	0.97
C1_01540W_A	0.3	0	0	0.35	0	0
CR_07500W_A	0.06	0.88	0.8	-0.11	0.75	0.65
CR_08380C_A	-0.15	0.75	0.62	0.38	0.58	0.45
C2_01690W_A	0.27	0	0	0.15	0.2	0.11
C1_08740C_A	1.08	0	0	1.2	0	0
C2_10570W_A	0.02	0.92	0.85	0.17	0.17	0.09
C2_04330C_A	0.35	0	0	0.27	0.05	0.02
C2_01580W_A	-0.02	0.94	0.88	-0.27	0.13	0.06
C7_00990W_A	0.11	0.25	0.13	-0.4	0	0
C3_02240C_A	-0.19	0.1	0.04	-0.38	0	0
C3_05000W_A	0.7	0	0	0.91	0	0
C2_04390W_A	0.71	0.11	0.04	0.85	0.1	0.05
C7_00250C_A	0.16	0.13	0.06	0.23	0.05	0.02
C7_03210W_A	-0.16	0.52	0.36	-0.12	0.61	0.48
C3_07700W_A	-0.04	0.75	0.62	-0.09	0.49	0.35
BGI_novel_G000(	-0.09	0.68	0.53	-0.28	0.11	0.05
C2_02530W_A	0.44	0.3	0.17	0.74	0.23	0.13
C2_00010W_A	0.01	0.94	0.88	0.05	0.73	0.62
C6_03560W_A	0.03	0.89	0.82	0	0.99	0.97
C6_04080W_A	-0.19	0.03	0.01	-0.21	0.06	0.03
C5_03970W_A	-0.34	0.04	0.01	-0.03	0.88	0.82
C4_02000C_A	-0.31	0.01	0	-0.17	0.15	0.08
C6_00500C_A	-0.07	0.75	0.62	0.07	0.75	0.64
C2_07720C_A	0.69	0	0	0.89	0	0
CR_03290C_A	0.47	0.3	0.17	0.79	0.09	0.05
C3_03850C_A	0.05	0.83	0.73	0.59	0	0
C1_12770W_A	-0.3	0.3	0.17	-0.39	0.19	0.11
C3_02350W_A	-1.08	0	0	-0.88	0.02	0.01
C1_11940C_A	-0.17	0.5	0.33	0.14	0.57	0.44
C1_13770C_A	-0.29	0.28	0.15	-0.02	0.97	0.94
C3_01140W_A	-0.1	0.78	0.66	-0.08	0.8	0.71
C1_05790W_A	-0.28	0.4	0.25	-0.17	0.62	0.48
CR_03790C_A	-0.08	0.85	0.76	0.8	0	0
C7_03520W_A	0.07	0.68	0.53	0.05	0.79	0.7

C3_00550C_A	0.41	0	0	0.06	0.55	0.41
C1_06060C_A	0.21	0.2	0.09	0.24	0.12	0.06
C5_04850W_A	-0.79	0.02	0.01	-0.53	0.15	0.08
CR_07570W_A	0	1	0.99	-0.08	0.69	0.57
C4_04430W_A	0.58	0	0	0.82	0	0
C3_01050C_A	0.69	0.12	0.05	0.08	0.92	0.87
C1_13310W_A	0.04	0.83	0.73	-0.11	0.51	0.37
CR_04180C_A	-0.09	0.49	0.32	0.13	0.32	0.2
C7_02180C_A	0.32	0.06	0.02	0.33	0.04	0.02
CR_00480W_A	0.17	0.13	0.06	0.16	0.2	0.11
C2_02800W_A	-0.16	0.51	0.35	-0.12	0.63	0.5
C2_04880C_A	0.32	0.09	0.04	0.25	0.22	0.12
C1_07680W_A	-0.73	0.01	0	-0.79	0.02	0.01
CR_04830C_A	-0.73	0	0	-0.49	0.03	0.01
C4_07240W_A	0.13	0.27	0.15	0.13	0.29	0.18
C1_09960W_A	-0.32	0.02	0	-0.28	0.03	0.01
C3_07640C_A	0.79	0	0	1.36	0	0
C2_02580W_A	0.03	0.95	0.9	-0.2	0.55	0.41
C4_01680W_A	0.23	0.3	0.16	0.24	0.28	0.17
CR_08830W_A	-0.07	0.67	0.52	0.12	0.81	0.73
C1_00830W_A	0.21	0.32	0.18	0.22	0.3	0.19
BGI_novel_G000(	0.48	0	0	1.07	0	0
C4_04280C_A	0.11	0.63	0.47	0.41	0.03	0.01
C3_02210C_A	0.12	0.28	0.15	0.03	0.82	0.74
C3_05360C_A	0.22	0.14	0.06	0.44	0	0
C2_09790W_A	-0.37	0.03	0.01	-0.4	0.01	0
C1_07010W_A	-0.1	0.59	0.43	-0.05	0.8	0.71
C2_06950C_A	0	1	1	-0.07	0.72	0.6
C1_14080W_A	-0.17	0.15	0.07	-0.43	0	0
C3_03570C_A	-1.47	0	0	-2.12	0	0
C2_00650W_A	0.08	0.68	0.53	-0.11	0.55	0.41
C2_08650W_A	0.11	0.52	0.36	0.16	0.29	0.18
C4_02510W_A	-0.1	0.65	0.5	0.01	0.96	0.94
C3_06670C_A	1.28	0	0	1.57	0	0
C1_03540C_A	0.02	0.92	0.86	0.02	0.92	0.87
CR_08020C_A	-0.12	0.53	0.36	-0.69	0	0
C2_00110W_A	0.67	0	0	1.14	0	0
CR_03680C_A	-0.58	0	0	-0.52	0.01	0
C2_02720W_A	0.11	0.26	0.14	0.14	0.15	0.08
C2_08980C_A	0.31	0.08	0.03	0.24	0.19	0.11
C4_04230W_A	0.28	0.16	0.08	0.2	0.34	0.22
C2_06570C_A	-0.09	0.44	0.27	0.04	0.76	0.66
C1_02500W_A	0.12	0.71	0.56	0.61	0.01	0

C2_09100C_A	-0.41	0	0	-0.49	0	0
C1_10220C_A	0.2	0.12	0.05	0.88	0	0
C6_00440C_A	-0.39	0	0	-2.39	0	0
C2_00600C_A	-0.18	0.4	0.24	0.08	0.71	0.6
CR_08940W_A	-0.25	0.21	0.1	0	0.99	0.98
CR_02160W_A	-0.26	0.04	0.01	-0.34	0.01	0
C7_00820W_A	0.45	0	0	0.81	0	0
C4_00590C_A	0.26	0	0	0.17	0.07	0.03
CR_03870W_A	-0.2	0.31	0.18	-0.42	0.04	0.02
C5_03330C_A	0.02	0.94	0.9	0.14	0.46	0.33
C1_09680W_A	0.57	0	0	0.39	0	0
C3_05160C_A	-0.92	0	0	-0.96	0	0
C1_09990W_A	0.34	0	0	0.15	0.17	0.09
CR_09310W_A	-0.15	0.2	0.1	-0.18	0.12	0.06
C4_02750W_A	-0.5	0.27	0.14	-0.77	0.21	0.12
C4_01930C_A	-0.04	0.92	0.86	0.51	0.09	0.04
CR_08360C_A	0.01	0.95	0.91	-0.42	0	0
C1_14430C_A	0.41	0	0	0.36	0.01	0
C6_01320W_A	0.05	0.65	0.49	-0.01	0.97	0.95
C1_05190C_A	0.1	0.78	0.66	0.37	0.18	0.1
BGI_novel_G000(	0.04	0.8	0.69	0.02	0.93	0.89
C4_01300W_A	-0.31	0	0	-1.22	0	0
C5_02840C_A	0.03	0.81	0.7	0.14	0.28	0.17
C4_00050W_A	0.07	0.57	0.41	0.21	0.07	0.03
C1_05120W_A	0.15	0.55	0.39	0.36	0.09	0.05
CR_01230C_A	0.07	0.74	0.6	0.09	0.66	0.53
C1_09650W_A	0.47	0	0	0.5	0	0
C1_04320W_A	0	0.99	0.98	0.05	0.69	0.57
C7_02300W_A	0.07	0.67	0.51	-0.56	0	0
C1_02870W_A	-0.12	0.57	0.41	-0.13	0.52	0.38
C4_04640C_A	0.1	0.7	0.55	-0.03	0.93	0.89
C7_02120C_A	-0.31	0	0	-0.39	0	0
C1_14490C_A	0.43	0	0	0.53	0	0
C1_00910W_A	-0.64	0.01	0	-0.24	0.38	0.25
C7_03990C_A	-0.06	0.74	0.6	0.03	0.87	0.81
CR_02430C_A	0.16	0.23	0.11	-0.1	0.53	0.39
C2_00220C_A	-0.2	0.09	0.04	0.1	0.45	0.32
C2_00090W_A	0.01	0.97	0.93	-0.03	0.91	0.86
C3_01060W_A	-0.07	0.67	0.52	-0.13	0.65	0.52
C5_04100W_A	0.19	0.09	0.03	0.13	0.25	0.15
C2_10660W_A	-0.35	0	0	-0.34	0	0
CR_00050W_A	-0.17	0.61	0.45	0.04	0.92	0.87
C1_09320C_A	0.39	0.01	0	0.82	0	0

C3_07000W_A	0.11	0.44	0.28	-0.09	0.54	0.4
C1_01700W_A	-0.25	0.05	0.02	-0.17	0.61	0.48
C5_00440C_A	0	1	1	0.01	0.94	0.91
CR_00690C_A	-0.02	0.96	0.92	-0.16	0.46	0.32
C2_01540W_A	0.35	0.02	0.01	0.69	0	0
C3_01970C_A	0.01	0.99	0.98	0.07	0.92	0.88
C2_10790C_A	0.02	0.91	0.85	-0.27	0.13	0.07
C3_01850W_A	-0.1	0.32	0.18	-0.4	0	0
C1_12680W_A	-0.56	0	0	-0.79	0	0
C5_02540C_A	0.11	0.77	0.65	-0.05	0.9	0.85
C3_04760C_A	0.36	0	0	0.48	0	0
CR_07200W_A	0.02	0.94	0.89	0.16	0.38	0.26
C2_08470C_A	0.08	0.86	0.77	-0.24	0.55	0.41
CR_02200C_A	0.09	0.47	0.31	-0.14	0.26	0.16
CR_03050C_A	-0.24	0.12	0.05	-0.17	0.28	0.17
C3_05270C_A	-0.32	0.06	0.02	-0.02	0.91	0.86
C2_00620C_A	-0.34	0.2	0.1	-0.2	0.49	0.35
CR_02570C_A	0.71	0	0	0.78	0	0
C3_00940W_A	0.22	0.02	0.01	0.33	0	0
CR_07160C_A	0.34	0.01	0	0.75	0	0
C1_03490W_A	0.52	0	0	0.31	0	0
C7_00910C_A	0.26	0.02	0	0.13	0.32	0.2
C4_03330W_A	0.13	0.45	0.29	0.45	0	0
C4_06940C_A	0.08	0.73	0.6	0.15	0.5	0.36
C1_05370C_A	-0.17	0.37	0.22	-0.2	0.28	0.17
C4_02870C_A	-0.09	0.37	0.22	-0.49	0	0
C2_01140C_A	0.07	0.67	0.52	0.12	0.67	0.55
C1_01640W_A	0.33	0	0	-0.11	0.38	0.25
C2_10120W_A	0.02	0.94	0.89	0.01	0.96	0.93
C5_01680C_A	0.12	0.48	0.31	0.65	0	0
C1_00530C_A	0.6	0	0	0.62	0	0
C1_12570C_A	-0.54	0.01	0	0.07	0.79	0.7
CR_04710W_A	-0.45	0.32	0.18	-0.28	0.6	0.47
C2_10740C_A	-0.25	0.11	0.05	-0.13	0.44	0.31
C7_03910W_A	-0.32	0.15	0.07	0.13	0.6	0.47
C5_00660C_A	-0.38	0	0	-1.37	0	0
C7_04270C_A	0.5	0	0	0.36	0	0
CR_03920C_A	-0.02	0.94	0.89	0.05	0.81	0.73
C1_00580W_A	0.04	0.86	0.77	0.08	0.73	0.62
CR_10020C_A	0.45	0	0	0.45	0	0
CR_01970C_A	0.06	0.67	0.52	0.13	0.23	0.14
C2_01190C_A	-0.17	0.57	0.4	-0.17	0.56	0.42
C1_06200W_A	0.48	0	0	0.52	0	0

CR_08180C_A	-0.22	0.19	0.09	-0.25	0.12	0.06
C1_05920W_A	-0.22	0.67	0.51	0.71	0.09	0.04
CR_06980W_A	-0.55	0.05	0.02	-0.42	0.14	0.07
C1_04480C_A	-0.02	0.89	0.82	-1.52	0	0
C3_07030C_A	-0.42	0.03	0.01	-0.17	0.45	0.31
C1_06000W_A	1	0.01	0	2.42	0	0
C5_00630C_A	-0.19	0.16	0.08	-0.11	0.5	0.36
CR_07180W_A	-0.38	0	0	-1.43	0	0
C2_02830C_A	0.64	0	0	0.7	0	0
C7_03630C_A	-0.3	0.08	0.03	-0.58	0	0
C1_01420C_A	0.21	0.21	0.11	0.82	0	0
C2_00310W_A	NA	NA	NA	NA	NA	NA
C2_01180W_A	-0.32	0.02	0.01	-0.56	0	0
CR_00930W_A	-0.01	0.99	0.97	0.23	0.73	0.62
C3_01450C_A	-0.35	0.03	0.01	-0.36	0.04	0.02
C2_03220C_A	0.51	0	0	1.15	0	0
CR_06560C_A	0.32	0.12	0.05	0.79	0	0
CR_08490W_A	-0.9	0	0	-1.06	0	0
C5_03870C_A	-0.39	0.02	0.01	-0.48	0.01	0
C3_03770C_A	-0.08	0.6	0.44	0	1	0.99
C1_08060W_A	-0.14	0.15	0.07	-0.02	0.89	0.84
C2_09720W_A	0.02	0.8	0.69	-0.09	0.36	0.24
C3_04660C_A	-0.05	0.92	0.86	0.42	0.28	0.17
CR_04950W_A	-0.34	0.03	0.01	-0.05	0.81	0.72
C7_02570C_A	0.08	0.48	0.32	0.24	0.02	0.01
C4_01250W_A	0.85	0	0	1.31	0	0
C1_07370C_A	0.24	0.33	0.19	0.33	0.15	0.08
CR_01960C_A	-0.23	0.16	0.07	-0.27	0.12	0.06
C4_00640W_A	0.21	0.68	0.53	0.33	0.64	0.51
CR_05540C_A	0.01	0.97	0.93	-0.13	0.54	0.4
C1_06580W_A	-0.19	0	0	-0.5	0	0
C3_04190W_A	0.98	0.02	0	2.15	0	0
CR_03340C_A	0.04	0.87	0.78	-0.02	0.93	0.89
C3_06850W_A	-0.82	0	0	-1.46	0	0
C1_09810W_A	0.33	0	0	0.3	0.01	0
C1_00290W_A	0.61	0	0	0.92	0	0
C1_10180C_A	0.44	0	0	0.26	0.07	0.03
C1_08270C_A	0.02	0.92	0.86	-0.54	0	0
C7_01850C_A	-0.01	0.97	0.94	0.1	0.32	0.2
C4_04790W_A	0.19	0.73	0.6	0.96	0.04	0.02
CR_06470W_A	0.19	0.07	0.03	0.05	0.71	0.59
C2_00470W_A	-0.19	0.06	0.02	-0.66	0	0
C5_02270W_A	0.37	0	0	0.12	0.27	0.16

C1_09130W_A	0.7	0	0	0.79	0	0
C1_08920W_A	-0.25	0.03	0.01	-0.23	0.04	0.02
C7_00630C_A	0.87	0.02	0.01	0.11	0.85	0.78
C6_03250W_A	0.28	0	0	0.55	0	0
C1_07150W_A	-0.49	0.27	0.14	-0.34	0.5	0.36
C4_02190C_A	-0.13	0.82	0.72	0.16	0.81	0.73
CR_06920W_A	-0.35	0.37	0.22	-0.51	0.19	0.11
C6_01810W_A	0.71	0.09	0.04	0.57	0.28	0.17
C3_05080W_A	-0.3	0.31	0.17	-0.06	0.85	0.78
C2_03940C_A	-0.29	0.08	0.03	-0.54	0	0
C1_01990W_A	-0.07	0.67	0.52	-0.01	0.99	0.98
CR_06490C_A	0	1	1	-0.48	0	0
C7_02430C_A	-0.03	0.97	0.94	-0.38	0.56	0.42
C1_02520W_A	-0.05	0.92	0.85	-0.48	0.37	0.24
C6_03910C_A	0.14	0.66	0.5	0.08	0.81	0.73
C3_06560W_A	0.11	0.55	0.39	0.28	0.08	0.04
CR_06540W_A	-0.82	0	0	-0.75	0	0
C6_01220C_A	-0.01	0.95	0.91	0.23	0.08	0.04
BGI_novel_G000	-0.03	0.96	0.92	0.6	0.07	0.03
C2_04440W_A	0.71	0.11	0.05	0.24	0.73	0.62
C3_02630C_A	-0.14	0.53	0.37	0.21	0.27	0.16
CR_06420W_A	0.18	0.27	0.15	0.39	0	0
C1_14110C_A	0	1	0.99	-0.56	0	0
C4_00700C_A	0	0.99	0.98	0.16	0.31	0.19
C7_02480W_A	-0.04	0.87	0.78	0.12	0.55	0.42
C5_00430W_A	-0.6	0	0	-1.07	0	0
CR_07730W_A	-0.26	0.49	0.33	0.09	0.82	0.74
C4_02500C_A	-0.43	0.14	0.06	-0.05	0.9	0.84
CR_10140W_A	0.27	0	0	0.5	0	0
C6_04610C_A	-0.16	0.32	0.18	-1.73	0	0
C2_05490W_A	-0.43	0.19	0.09	-0.28	0.43	0.3
C7_00870W_A	0	1	1	1.35	0	0
C3_01720C_A	0.74	0	0	0.83	0	0
C2_06650C_A	-0.86	0	0	-0.7	0	0
C4_07130W_A	-0.16	0.16	0.08	-0.18	0.13	0.07
C4_02640C_A	-0.21	0.57	0.41	0.68	0.02	0.01
C4_04130W_A	0.07	0.63	0.47	-0.08	0.57	0.44
C5_02110W_A	-1.78	0	0	-1.92	0	0
C2_10130W_A	0.2	0.59	0.43	-0.03	0.94	0.91
C1_06560W_A	-0.14	0.45	0.29	-0.14	0.47	0.33
C2_00660C_A	-0.03	0.97	0.94	0.73	0.06	0.03
CR_02880W_A	-0.57	0.11	0.04	0.09	0.83	0.76
CR_08670C_A	0.58	0	0	0.94	0	0

C1_06670W_A	-0.54	0	0	-0.39	0.01	0
C1_05220C_A	0.2	0.71	0.57	0.03	0.97	0.94
C5_03120W_A	0.21	0.16	0.08	0.1	0.56	0.43
CR_05560W_A	0.32	0.03	0.01	0.24	0.13	0.07
C7_03560W_A	-0.12	0.78	0.65	-0.48	0.37	0.25
C5_03240W_A	-0.14	0.24	0.12	-0.66	0	0
CR_10650W_A	-0.73	0	0	-0.42	0.03	0.01
C2_08140C_A	-0.29	0.01	0	-0.33	0	0
C3_06000W_A	-0.29	0.05	0.02	-0.58	0	0
C2_09920W_A	-0.2	0.51	0.34	0.09	0.79	0.7
C6_00520W_A	0.19	0.6	0.44	0.32	0.3	0.19
C2_07170C_A	-0.14	0.43	0.27	-0.12	0.53	0.39
C7_02010C_A	0.23	0.64	0.48	0.83	0.05	0.02
CR_07620W_A	0.09	0.47	0.3	0.09	0.52	0.38
C1_02130C_A	0.74	0	0	0.58	0	0
C7_01180W_A	0.03	0.9	0.82	-0.09	0.68	0.55
C1_06810W_A	-0.25	0.01	0	-0.12	0.34	0.22
CR_09810W_A	-0.32	0.31	0.17	0.14	0.68	0.56
C1_10870W_A	-0.19	0	0	-0.55	0	0
C4_02280W_A	0.08	0.55	0.38	-0.13	0.29	0.18
C2_03330C_A	0.08	0.47	0.31	0.23	0.02	0.01
CR_00360C_A	-0.16	0.11	0.04	-0.41	0	0
C4_02890C_A	-0.18	0.55	0.38	0.13	0.66	0.53
C6_02350C_A	-0.66	0	0	-0.35	0.06	0.03
C5_02260C_A	-0.07	0.74	0.6	-0.07	0.7	0.59
CR_02930W_A	-0.2	0.15	0.07	-0.08	0.82	0.74
CR_07700W_A	0.3	0.28	0.15	1.06	0	0
C2_05950C_A	0.57	0.01	0	0.75	0	0
C4_02110W_A	0.55	0	0	0.72	0	0
C4_05070C_A	0.68	0.01	0	1.55	0	0
CR_08910C_A	0.26	0	0	0.41	0	0
C6_03070C_A	-0.06	0.87	0.78	-0.26	0.62	0.48
C3_07020W_A	0.22	0.02	0.01	0.01	0.95	0.91
C1_12800W_A	-0.21	0.71	0.56	-0.18	0.79	0.7
CR_01500W_A	0.07	0.66	0.5	0.29	0.02	0.01
C1_11620W_A	-0.17	0.57	0.4	-0.58	0.02	0.01
C1_02200C_A	0.13	0.6	0.44	0.21	0.35	0.23
C1_05680C_A	0.07	0.59	0.42	0	0.99	0.97
C1_10860C_A	0.15	0.52	0.35	0.14	0.53	0.39
C3_01430W_A	-1.14	0	0	-0.94	0.02	0.01
C1_01180C_A	0.23	0.67	0.51	0.18	0.79	0.69
C2_05790C_A	-0.36	0	0	-0.31	0.01	0
C4_06620C_A	-0.46	0.33	0.19	-1.24	0.03	0.01

CR_07450C_A	0.54	0	0	0.74	0	0
C1_10480W_A	-0.02	0.92	0.86	0.01	0.98	0.96
C1_08030W_A	0.48	0	0	0.66	0	0
C1_05030C_A	0.29	0.02	0.01	0.6	0	0
CR_01540W_A	-0.05	0.85	0.76	0.09	0.7	0.59
C1_13840W_A	-0.02	0.94	0.89	0.29	0.02	0.01
C1_05140W_A	0.16	0.11	0.05	0.72	0	0
C2_09840W_A	-0.38	0.27	0.14	-0.5	0.11	0.05
C4_04180C_A	-0.14	0.58	0.42	0.36	0.07	0.03
C2_00400C_A	0.09	0.49	0.32	0.24	0.03	0.01
C4_02850W_A	0.07	0.65	0.5	-0.05	0.76	0.66
C3_02280C_A	0.73	0	0	1.32	0	0
C1_12450C_A	0.11	0.42	0.26	-0.28	0.03	0.01
C1_12010C_A	0.18	0.5	0.33	0.29	0.2	0.11
C1_04070C_A	-0.1	0.65	0.48	-0.01	0.97	0.94
C3_04020C_A	0.17	0.29	0.16	0.3	0.04	0.02
C5_05500C_A	0	1	0.99	-0.03	0.8	0.71
C4_03840C_A	-0.19	0.44	0.28	-0.02	0.95	0.92
C1_07460C_A	0.2	0.2	0.1	0.17	0.31	0.19
C5_00050W_A	0.54	0	0	0.49	0	0
C4_03620C_A	0.22	0.32	0.18	-0.16	0.55	0.41
C1_11060C_A	-0.12	0.11	0.05	-0.49	0	0
C3_06430W_A	-0.03	0.84	0.74	0.06	0.7	0.59
C4_00790C_A	-0.23	0.34	0.19	0.09	0.73	0.62
CR_09520C_A	-0.43	0.01	0	-0.44	0.01	0
C2_06120C_A	0.1	0.8	0.68	0.14	0.67	0.55
C4_03170W_A	-0.16	0.47	0.3	-0.18	0.39	0.27
C4_05000W_A	0.15	0.49	0.32	0.28	0.12	0.06
CR_03450W_A	-0.41	0.4	0.25	-0.26	0.68	0.56
CR_00610W_A	0.42	0.08	0.03	0.29	0.28	0.17
C4_07090C_A	0.09	0.54	0.37	0.26	0.03	0.01
C5_02460C_A	1.57	0	0	2.53	0	0
C4_05150W_A	-0.06	0.55	0.38	-0.09	0.42	0.29
C3_05130C_A	0.08	0.89	0.81	0.02	0.97	0.95
C4_05790W_A	0.58	0	0	0.85	0	0
C1_02710W_A	0.02	0.83	0.72	-0.2	0.01	0.01
CR_10720W_A	0.28	0.09	0.04	0.24	0.18	0.1
C6_03450C_A	-0.06	0.53	0.36	-0.19	0.03	0.01
C3_05940C_A	-0.4	0.1	0.04	-0.01	0.97	0.94
C1_10580C_A	0.7	0.12	0.05	2.08	0	0
CR_09060W_A	0.05	0.94	0.89	0.23	0.72	0.61
C4_01290W_A	0.02	0.94	0.89	0.28	0.04	0.02
C3_01510W_A	0.26	0.01	0	0.24	0.05	0.02

C7_03260C_A	0.2	0.46	0.29	0.24	0.3	0.18
CR_04250W_A	0.34	0.43	0.27	0.88	0.02	0.01
C3_05510W_A	-0.22	0.19	0.09	-0.12	0.58	0.44
C2_04310W_A	0.12	0.12	0.05	-0.23	0.03	0.01
C2_10070W_A	-0.18	0.67	0.52	0.29	0.68	0.56
C1_06720C_A	-0.22	0.08	0.03	0.02	0.89	0.83
CR_06810W_A	-0.11	0.29	0.16	-0.69	0	0
C2_00950C_A	0.29	0.01	0	0.33	0.01	0
C2_05080C_A	-0.27	0.33	0.19	-0.37	0.17	0.09
C4_00860C_A	2.2	0	0	3.37	0	0
C6_01150W_A	-0.17	0.28	0.15	-0.18	0.22	0.13
CR_08350W_A	0.83	0	0	0.67	0	0
C1_14630C_A	-0.43	0.03	0.01	-0.87	0	0
CR_03890W_A	-0.37	0.02	0	-0.2	0.19	0.1
C3_02330C_A	-0.28	0.55	0.39	-0.86	0.07	0.03
CR_10350C_A	0.28	0.03	0.01	0.38	0	0
C3_05470W_A	-0.35	0.04	0.02	-0.14	0.49	0.36
C1_11560C_A	0	1	0.99	0.1	0.56	0.43
C1_07510W_A	-0.23	0.18	0.09	-0.22	0.2	0.11
C6_02500C_A	-1.03	0	0	-1.34	0	0
C5_04940W_A	-0.14	0.68	0.53	0.37	0.17	0.09
C3_04370C_A	0.15	0.39	0.24	0.04	0.85	0.78
C3_05810C_A	0.58	0	0	0.98	0	0
C2_02990C_A	0.26	0.01	0	0.36	0	0
C1_02840W_A	0.25	0.05	0.02	0	0.99	0.97
C2_09520C_A	0.32	0	0	0.42	0	0
C2_01300C_A	-0.4	0	0	-0.39	0	0
C1_02760W_A	0.48	0	0	0.51	0	0
C3_00830C_A	-0.17	0.19	0.09	-0.07	0.64	0.51
C1_06890C_A	-0.06	0.45	0.29	-0.45	0	0
C4_01190W_A	0.29	0.01	0	1.18	0	0
C2_03240C_A	0.1	0.37	0.22	0.21	0.09	0.04
C6_02430W_A	-0.26	0.41	0.25	0.11	0.61	0.48
CR_07750C_A	-0.22	0.17	0.08	-0.1	0.59	0.46
CR_04330W_A	-0.13	0.51	0.35	-0.02	0.94	0.9
C5_04200W_A	NA	NA	NA	NA	NA	NA
C2_07580W_A	0.05	0.9	0.82	-0.19	0.56	0.43
CR_08080W_A	-0.02	0.98	0.96	0.33	0.61	0.47
CR_03530W_A	0.41	0	0	0.72	0	0
C2_05330C_A	-0.19	0.54	0.38	0.06	0.85	0.78
C3_03920W_A	-0.72	0.01	0	-0.78	0	0
C1_07930C_A	-0.01	0.98	0.96	-0.59	0.01	0
C5_00500W_A	-0.41	0	0	-0.5	0	0

CR_00130C_A	0.26	0	0	0.31	0	0
CR_10030W_A	0.74	0	0	0.84	0	0
C7_01990C_A	0.09	0.76	0.64	0.08	0.78	0.68
C4_01730C_A	0.08	0.58	0.42	-0.17	0.24	0.14
C6_01400W_A	-0.03	0.96	0.93	-0.28	0.62	0.48
C2_05550W_A	0.22	0.29	0.15	0.07	0.78	0.68
C3_01350C_A	-0.24	0.07	0.03	-0.36	0	0
CR_06270W_A	0.43	0.08	0.03	0.44	0.08	0.04
C1_04750W_A	-0.58	0	0	-0.38	0.05	0.02
C1_07250C_A	0.24	0.02	0.01	-0.18	0.18	0.1
C2_04230W_A	0.29	0	0	0.23	0.02	0.01
C1_11450C_A	-0.33	0	0	-0.76	0	0
C1_00440W_A	0.09	0.38	0.23	0.47	0	0
C1_09790C_A	-0.16	0.34	0.2	0.01	0.97	0.95
C5_05360C_A	-0.07	0.61	0.44	-0.19	0.16	0.09
C2_07710W_A	0	0.99	0.98	0.19	0.75	0.65
C1_09440W_A	0.21	0.27	0.14	0.15	0.47	0.34
C6_04310W_A	0.02	0.95	0.91	-0.12	0.6	0.47
C4_02740W_A	0.23	0.25	0.13	0.75	0	0
C1_04160C_A	-0.18	0.5	0.34	0.01	0.98	0.97
C5_03610W_A	-0.16	0.27	0.14	-1.11	0	0
C3_01370C_A	0.43	0.31	0.17	0.49	0.29	0.18
C3_05980C_A	-0.06	0.85	0.76	0.05	0.87	0.8
C7_01340W_A	-0.07	0.64	0.48	0.11	0.49	0.35
C7_00390W_A	0	0.98	0.96	-0.17	0.04	0.02
C3_06870W_A	-0.14	0.04	0.02	-0.31	0	0
CR_09470W_A	0.1	0.61	0.44	0.8	0.01	0
C7_04110W_A	0.22	0.16	0.07	0.25	0.09	0.04
C2_05390C_A	-0.59	0.15	0.07	-0.33	0.49	0.35
CR_09940W_A	0.61	0.13	0.06	0.76	0.09	0.04
C1_08330C_A	0.88	0.03	0.01	1.81	0	0
C7_03790W_A	-0.54	0	0	-0.46	0	0
C2_05350C_A	0.08	0.86	0.77	-0.22	0.57	0.44
C3_04220C_A	0.01	0.97	0.94	0.02	0.94	0.9
C1_03500W_A	0.44	0	0	0.56	0	0
C4_00920C_A	-0.32	0.42	0.26	-0.6	0.14	0.07
C3_03210W_A	-0.2	0.15	0.07	-0.31	0.03	0.01
C2_07610C_A	0.25	0.01	0	0.3	0	0
C7_01700W_A	0.94	0	0	1.58	0	0
C4_05850C_A	-0.61	0	0	-1.38	0	0
C1_03000W_A	-0.47	0.04	0.01	-0.63	0.01	0
C3_07320W_A	0.11	0.4	0.24	0.04	0.76	0.66
C1_12050W_A	0.09	0.18	0.09	0.23	0	0

C7_01370W_A	-0.27	0.33	0.19	-0.08	0.79	0.7
C1_13020C_A	0.68	0	0	0.89	0	0
C2_03100W_A	0.17	0.05	0.02	-0.23	0.01	0.01
C1_06980C_A	-0.27	0.04	0.02	-0.48	0	0
C4_01440W_A	0.12	0.33	0.19	0.26	0.02	0.01
C7_01620C_A	0	0.99	0.98	-0.01	0.96	0.93
C1_10320W_A	-0.11	0.57	0.41	-0.05	0.8	0.71
C3_01200W_A	-1.42	0	0	-1.88	0	0
C6_03660C_A	0.5	0	0	0.25	0.03	0.01
CR_03720W_A	0.1	0.22	0.11	0.21	0.03	0.01
C7_00490C_A	-0.26	0.07	0.03	-0.61	0	0
C2_09260C_A	0.33	0	0	0.58	0	0
C4_03570W_A	0.78	0.01	0	0.23	0.56	0.43
C5_01240W_A	-0.37	0.05	0.02	-0.78	0	0
C4_05770C_A	0.05	0.84	0.74	0.09	0.69	0.57
C3_01460C_A	0.36	0.07	0.03	0.65	0	0
C5_01590W_A	-0.27	0.04	0.01	-0.32	0.02	0.01
C1_11210C_A	0.14	0.31	0.17	0.41	0	0
C4_03110W_A	0.62	0	0	0.57	0	0
C6_02840C_A	0.1	0.64	0.48	0.05	0.82	0.74
C2_07020C_A	-0.04	0.92	0.85	0.26	0.27	0.16
C2_04540C_A	0.27	0.16	0.07	0.16	0.45	0.31
C2_04160W_A	0.04	0.79	0.67	0.01	0.97	0.95
CR_10320W_A	NA	NA	NA	NA	NA	NA
C7_01390W_A	-0.17	0.73	0.59	0.84	0.02	0.01
CR_03550W_A	-0.36	0.09	0.03	-0.12	0.62	0.48
C4_03960W_A	0.39	0	0	0.05	0.73	0.62
C4_05030C_A	0.22	0.04	0.01	0.38	0	0
C1_12080W_A	-0.05	0.78	0.66	-0.08	0.64	0.51
C2_03910C_A	0.38	0	0	0.17	0.31	0.2
CR_09880W_A	-0.1	0.36	0.21	-0.01	0.95	0.92
C2_04970W_A	-0.03	0.94	0.89	-0.5	0.08	0.04
C4_06810C_A	0.65	0	0	0.69	0	0
C6_00740W_A	-0.01	0.94	0.89	-0.03	0.86	0.79
C5_00710W_A	0.05	0.92	0.86	0.24	0.56	0.42
C4_06210C_A	-0.8	0	0	-1.11	0	0
C3_01040C_A	0.05	0.91	0.84	0.29	0.36	0.23
C3_04570C_A	-0.18	0.67	0.52	0.22	0.75	0.65
C2_02950W_A	-0.22	0	0	-0.34	0	0
C1_03310W_A	0.01	0.94	0.9	-0.13	0.21	0.12
C2_01440C_A	0.09	0.75	0.61	0.14	0.56	0.42
C5_04690C_A	-0.13	0.75	0.62	-0.31	0.6	0.46
C1_01880C_A	-0.22	0.24	0.12	-0.65	0	0

C4_05540W_A	0.23	0.02	0.01	0.08	0.52	0.38
C1_11280W_A	0.12	0.56	0.4	0.39	0.02	0.01
C6_00030W_A	-0.17	0.22	0.11	-0.23	0.1	0.05
C4_04550C_A	0.64	0	0	1	0	0
C2_02910W_A	1.38	0	0	4.7	0	0
C1_13960W_A	0.33	0	0	0.3	0.01	0
C1_03270W_A	0	1	0.99	0.26	0.12	0.06
C6_02380W_A	-0.62	0	0	-0.39	0	0
C3_06720W_A	-0.46	0.01	0	-0.53	0	0
C1_02050C_A	-0.39	0	0	-0.86	0	0
C2_07060W_A	0.25	0	0	-0.08	0.51	0.37
C5_02970W_A	-0.03	0.9	0.83	-0.12	0.49	0.35
CR_05970C_A	0.02	0.88	0.8	0	0.98	0.96
C2_07200W_A	-0.24	0.39	0.24	-0.19	0.52	0.38
C1_03240W_A	-0.33	0	0	-0.25	0.04	0.02
C2_02670C_A	0.34	0.23	0.12	0.33	0.27	0.16
C5_01040W_A	0.39	0.18	0.09	0.74	0	0
C5_01350W_A	0.24	0.16	0.07	0.23	0.18	0.1
C1_01940C_A	-0.06	0.75	0.62	-0.01	0.97	0.94
C2_03000C_A	-0.25	0.3	0.17	-0.08	0.78	0.68
C6_01180C_A	-0.12	0.21	0.1	-0.42	0	0
C1_07880C_A	-0.8	0	0	-1.36	0	0
CR_09650W_A	0.32	0	0	0.3	0.01	0
C1_08610C_A	-0.9	0	0	-0.8	0	0
C5_04790C_A	0.07	0.83	0.73	-0.06	0.84	0.77
C1_01440C_A	0.22	0.39	0.24	0.37	0.38	0.25
C4_01050C_A	-0.37	0.47	0.31	-0.25	0.71	0.6
C6_02960W_A	-0.38	0.06	0.02	-0.09	0.72	0.61
C2_01570W_A	0.27	0.39	0.24	0.42	0.16	0.09
C1_01750W_A	0.3	0.01	0	-0.12	0.33	0.21
C1_11670W_A	-0.28	0	0	-0.67	0	0
CR_04640W_A	-0.35	0.47	0.31	-0.12	0.83	0.75
CR_04560C_A	-0.07	0.73	0.59	-0.04	0.84	0.77
C5_01170W_A	-0.27	0.55	0.39	-0.25	0.62	0.49
C4_02160C_A	-0.41	0.03	0.01	-0.32	0.09	0.04
C1_03720C_A	0.24	0.11	0.05	0.26	0.05	0.02
C1_09110W_A	-0.56	0.02	0.01	-0.08	0.8	0.71
C2_03150C_A	0.24	0.17	0.08	0.19	0.27	0.16
C1_13170C_A	-0.03	0.82	0.72	-0.26	0.03	0.01
C2_01860C_A	0.03	0.9	0.82	0.11	0.56	0.42
C3_03270W_A	0.12	0.54	0.38	0.12	0.53	0.39
C7_02200W_A	-0.06	0.85	0.76	-0.25	0.34	0.22
C1_11960C_A	0.39	0.34	0.2	0.41	0.35	0.23

C1_02360C_A	-0.15	0.78	0.66	-0.84	0.14	0.07
C5_00640C_A	0.19	0.22	0.11	0.48	0	0
C1_12520W_A	-0.34	0.05	0.02	-0.08	0.7	0.58
C1_10460W_A	-0.03	0.9	0.82	-0.21	0.18	0.1
C2_02730W_A	-0.05	0.72	0.58	0.19	0.08	0.04
C3_00220W_A	-0.75	0.02	0.01	-1.52	0	0
C7_01550W_A	-0.27	0.01	0	-0.02	0.88	0.82
C1_10110W_A	-0.02	0.96	0.92	-0.27	0.28	0.17
C7_03360W_A	-0.12	0.14	0.06	-1.52	0	0
C4_00340W_A	0.42	0	0	0.35	0	0
C5_03140C_A	0.39	0	0	0.49	0	0
CR_04760C_A	-0.54	0	0	-0.77	0	0
C1_11800C_A	-0.22	0.44	0.28	-0.15	0.59	0.45
C2_10870W_A	0.05	0.78	0.66	0.1	0.58	0.45
C6_00400C_A	0.22	0.32	0.18	0.37	0.06	0.03
C7_01000C_A	NA	NA	NA	NA	NA	NA
C3_05120C_A	-0.19	0.19	0.09	-0.02	0.92	0.87
C6_00620W_A	-0.68	0	0	-1.56	0	0
C1_13370W_A	-0.5	0.03	0.01	-0.53	0.03	0.01
C4_03460C_A	0.37	0.36	0.21	0.64	0.08	0.04
CR_00530W_A	-0.01	0.99	0.98	0.09	0.88	0.82
C7_03460W_A	0.28	0.44	0.28	0.16	0.67	0.55
C1_04620W_A	-0.25	0.06	0.02	0.13	0.44	0.31
C1_01560W_A	-0.06	0.67	0.52	-0.02	0.91	0.87
CR_09380W_A	0.21	0.01	0	0.4	0	0
CR_10780C_A	-0.04	0.81	0.69	-0.19	0.12	0.06
C3_04550C_A	0.03	0.76	0.63	-0.25	0.01	0
CR_10630W_A	-0.16	0.65	0.49	0.33	0.26	0.16
C1_09490C_A	-0.42	0	0	-1.25	0	0
C4_01240C_A	0.46	0.01	0	1	0	0
C7_01930C_A	-0.36	0.03	0.01	0.24	0.14	0.07
C2_02360C_A	0.36	0.14	0.06	0.29	0.26	0.16
C6_00810C_A	-0.01	0.97	0.94	-0.04	0.96	0.93
C1_02380C_A	-0.27	0.44	0.28	-0.03	0.95	0.91
C1_03800W_A	-0.46	0.13	0.06	-0.21	0.53	0.39
C7_03380W_A	-0.33	0.25	0.13	-0.18	0.56	0.42
C1_14530W_A	0.38	0.01	0	0.58	0	0
CR_04970C_A	-0.05	0.88	0.8	-0.1	0.89	0.83
CR_02350C_A	-0.01	0.97	0.94	-0.48	0	0
C5_02040W_A	0.22	0.02	0	0.15	0.17	0.09
CR_04880W_A	-0.28	0.05	0.02	-0.07	0.68	0.55
C5_02890W_A	0.37	0	0	0.45	0	0
C7_00710W_A	-0.16	0.02	0.01	-0.52	0	0

C3_07410C_A	0.06	0.47	0.3	-0.41	0	0
CR_10600C_A	0.44	0	0	0.32	0	0
C2_07830W_A	-0.21	0.31	0.17	-0.43	0.02	0.01
C3_06620W_A	-0.33	0.03	0.01	0.09	0.62	0.48
C1_00810W_A	-1.05	0	0	-1.54	0	0
CR_00750C_A	-0.1	0.67	0.51	0.02	0.95	0.91
C4_00820W_A	0.07	0.78	0.66	0.31	0.17	0.09
C3_00790W_A	-0.13	0.46	0.3	-0.1	0.59	0.45
CR_02820W_A	0.52	0	0	0.52	0	0
C2_02270C_A	-0.3	0.01	0	-0.34	0.01	0
C7_01800C_A	0.56	0	0	0.27	0	0
C6_01290C_A	0.56	0.01	0	1.19	0	0
C1_02440C_A	-0.19	0.41	0.25	0.25	0.23	0.14
C7_00030W_A	0.52	0	0	0.37	0	0
C1_08980C_A	-0.15	0.03	0.01	-0.18	0.03	0.01
CR_04580W_A	-0.16	0.08	0.03	-0.4	0	0
C3_00960W_A	0.15	0.41	0.26	0.44	0	0
C5_00950C_A	-0.12	0.45	0.29	0	0.99	0.98
CR_08110W_A	-0.23	0.4	0.24	-0.32	0.19	0.1
C2_01430W_A	-0.24	0	0	-0.55	0	0
C2_02150C_A	-0.09	0.68	0.54	0.25	0.2	0.11
C3_04470W_A	-0.09	0.34	0.2	-0.21	0.03	0.01
C4_01750C_A	-0.11	0.09	0.03	-0.21	0	0
C2_07030C_A	-0.13	0.24	0.13	-0.25	0.01	0.01
C7_03320C_A	0.01	0.97	0.93	0.14	0.25	0.15
CR_04150W_A	-0.07	0.9	0.83	0.26	0.65	0.52
CR_05080W_A	0.17	0.08	0.03	0.11	0.36	0.24
C5_04000W_A	0.18	0.14	0.06	0.15	0.24	0.14
CR_09770C_A	0.07	0.62	0.46	-0.14	0.25	0.15
CR_09040W_A	0.66	0.02	0.01	0.82	0	0
C4_04760C_A	-0.14	0.31	0.17	-0.2	0.2	0.11
C5_02010C_A	-0.2	0.34	0.2	-0.2	0.36	0.24
C5_02610C_A	-0.44	0.02	0.01	-0.22	0.31	0.19
CR_01420W_A	-0.43	0	0	-0.17	0.19	0.1
C7_02360W_A	0.01	0.98	0.97	0.47	0.48	0.34
C1_06860W_A	-0.07	0.8	0.68	-0.02	0.98	0.96
C4_06670W_A	0.1	0.75	0.61	0.4	0.09	0.04
C2_09300W_A	-0.13	0.39	0.24	-0.01	0.98	0.96
C1_10280C_A	0	1	1	0.06	0.61	0.48
C4_03510C_A	0.48	0.28	0.15	0.24	0.67	0.55
C2_07310W_A	0.33	0	0	0.36	0	0
C3_03610W_A	0.06	0.79	0.67	0.09	0.69	0.57
C2_00330C_A	0.05	0.7	0.56	-0.08	0.54	0.4

C1_13720W_A	-0.35	0.01	0	0.04	0.86	0.79
C6_04260C_A	-0.43	0.04	0.01	-0.32	0.16	0.08
C2_06760C_A	-0.49	0.07	0.03	-0.79	0	0
CR_04490C_A	0.22	0.19	0.09	-0.38	0.02	0.01
C7_00860W_A	0.47	0	0	0.46	0	0
C4_04720W_A	-0.66	0	0	-1.78	0	0
C5_04890C_A	-0.45	0.07	0.03	-0.06	0.85	0.79
C1_12920C_A	0.14	0.4	0.25	0.15	0.36	0.24
C2_09330C_A	0.07	0.67	0.52	NA	NA	NA
C7_03770C_A	-0.07	0.75	0.62	-0.29	0.11	0.06
C2_08870C_A	0.32	0	0	1	0	0
C3_03640W_A	0.49	0.01	0	0.12	0.63	0.5
C3_07890W_A	0.1	0.53	0.36	0.08	0.65	0.52
C6_03890C_A	0.19	0.1	0.04	0.23	0.07	0.03
C4_06860C_A	0.73	0	0	0.84	0	0
C2_05030C_A	0.56	0	0	0.79	0	0
C5_05410C_A	0.17	0.03	0.01	-0.31	0	0
C5_02660C_A	-0.24	0.01	0	-0.24	0.01	0
C3_01120W_A	0.19	0.52	0.36	0.66	0	0
CR_02600W_A	0.08	0.85	0.76	0.14	0.71	0.6
C7_00200W_A	-0.02	0.93	0.87	-0.06	0.8	0.7
C7_03040W_A	-0.28	0.29	0.16	-0.37	0.16	0.09
C2_02230C_A	0	1	1	-0.48	0.45	0.32
C3_07670W_A	-0.12	0.17	0.08	-0.49	0	0
C3_04680W_A	-0.11	0.33	0.19	-0.57	0	0
C4_06130W_A	-0.02	0.84	0.74	0.02	0.85	0.79
C6_03420W_A	-0.11	0.67	0.51	-0.43	0.04	0.02
C4_06040W_A	0.23	0.44	0.27	0.36	0.17	0.09
CR_07590W_A	0.67	0	0	0.41	0	0
C5_00010W_A	-0.06	0.74	0.6	0.12	0.46	0.33
C6_00670W_A	-0.33	0	0	-0.67	0	0
C7_01960W_A	0.15	0.42	0.26	0.18	0.29	0.18
C1_04350C_A	-0.11	0.73	0.59	0.19	0.49	0.35
CR_02220C_A	0.2	0.05	0.02	-1.78	0	0
C2_01420C_A	-0.64	0	0	-0.52	0	0
C4_06770W_A	0.15	0.37	0.22	0.08	0.66	0.53
C6_02290C_A	-0.54	0.01	0	0.07	0.78	0.68
C2_10560C_A	0.06	0.53	0.36	0.15	0.12	0.06
C2_08640C_A	-0.42	0.01	0	-0.21	0.22	0.12
C7_03120W_A	0.08	0.48	0.31	-0.08	0.52	0.38
C6_01480W_A	0.15	0.71	0.56	-0.1	0.82	0.75
C1_02570C_A	-0.37	0.27	0.14	-0.53	0.11	0.05
CR_00850C_A	0.33	0.01	0	0.22	0.11	0.05

C3_05570W_A	-0.19	0.24	0.13	-0.2	0.22	0.13
C5_00250C_A	-0.3	0.17	0.08	-0.47	0.02	0.01
C1_01320W_A	-0.25	0.03	0.01	0.04	0.81	0.72
C5_01420W_A	-0.03	0.95	0.91	0.12	0.79	0.7
C7_03450C_A	-0.29	0.02	0.01	-0.13	0.33	0.21
CR_10260W_A	-0.16	0.66	0.51	-0.12	0.72	0.61
C2_10810W_A	-0.42	0.02	0	-0.57	0	0
C3_04750W_A	0.22	0.25	0.13	0.24	0.19	0.1
C1_00770C_A	0.2	0.44	0.28	0.52	0.01	0
CR_09370W_A	-0.76	0	0	-1.05	0	0
C2_05620W_A	-0.24	0.37	0.22	-0.1	0.87	0.8
C2_06170C_A	-0.91	0	0	-1.37	0	0
C4_05190C_A	-0.18	0.27	0.15	-0.13	0.49	0.35
C5_00220W_A	-0.01	0.95	0.9	-1.1	0	0
C1_13440C_A	-0.64	0.12	0.05	-0.73	0.12	0.06
C1_10910C_A	-0.12	0.53	0.37	-0.26	0.1	0.05
C4_07000W_A	-0.01	0.99	0.97	0.34	0.62	0.48
C4_00800W_A	-0.54	0.03	0.01	-0.08	0.78	0.69
C2_00100C_A	0.92	0	0	0.91	0	0
C3_01860C_A	-0.1	0.34	0.2	-0.1	0.35	0.23
C2_02350C_A	0.02	0.9	0.83	-0.05	0.75	0.65
C6_02200C_A	0.5	0.28	0.15	1.9	0	0
C2_00290W_A	0.18	0.43	0.27	0.32	0.13	0.07
C4_01130C_A	-0.34	0.25	0.13	-0.41	0.17	0.09
C2_06290C_A	0.33	0	0	0.82	0	0
CR_08500W_A	-0.34	0.01	0	-0.46	0	0
C3_02770C_A	0.29	0.06	0.02	0.28	0.07	0.03
CR_07670W_A	-0.36	0.01	0	-0.19	0.27	0.16
C2_00200W_A	-0.21	0.16	0.08	-0.17	0.28	0.17
C5_03680W_A	-0.14	0.34	0.2	-0.31	0.01	0
C3_04360W_A	0.06	0.72	0.58	-0.02	0.91	0.87
C3_00560C_A	-0.5	0	0	-0.64	0	0
C4_04290W_A	0.19	0.1	0.04	0.38	0	0
C4_05780C_A	0.46	0.1	0.04	-0.51	0.13	0.06
C4_06470W_A	0.87	0	0	1.57	0	0
C3_03490W_A	0.13	0.42	0.26	-0.27	0.08	0.04
C2_03200W_A	-0.74	0	0	-0.93	0	0
C2_00080C_A	0.35	0.05	0.02	0.53	0	0
C7_03810W_A	-0.02	0.86	0.78	0.26	0.04	0.02
C2_04180C_A	0	1	0.99	0.12	0.62	0.49
C1_05910W_A	-0.3	0.07	0.03	0.21	0.22	0.13
C1_10260C_A	-0.67	0	0	-0.8	0	0
CR_00490W_A	0.02	0.82	0.72	-0.03	0.76	0.65

CR_06320C_A	0.2	0.45	0.29	0.5	0.02	0.01
CR_02240C_A	0.34	0.01	0	-1.08	0	0
C4_03100W_A	0.95	0	0	-0.02	0.97	0.95
C5_04300C_A	0.04	0.66	0.5	-0.32	0	0
C2_01720C_A	0.04	0.88	0.79	0.25	0.22	0.13
C5_04290C_A	-0.17	0.09	0.04	-0.05	0.69	0.57
C4_00890W_A	0.13	0.08	0.03	-0.17	0.08	0.03
C3_01190C_A	-0.39	0.01	0	-0.03	0.91	0.86
C4_00230W_A	-0.05	0.78	0.65	-0.04	0.77	0.67
C1_04360C_A	0.42	0.02	0.01	0.97	0	0
C5_04770W_A	-0.01	0.95	0.91	-0.02	0.91	0.86
C4_02010C_A	-0.7	0	0	-0.81	0	0
C1_12560C_A	0.29	0.54	0.38	0.46	0.49	0.35
CR_06060W_A	-0.11	0.69	0.54	0.02	0.94	0.9
C2_05930W_A	0.02	0.98	0.95	0.36	0.22	0.12
CR_08400C_A	-0.2	0.1	0.04	-0.2	0.09	0.04
C4_06680C_A	-0.1	0.3	0.17	-0.02	0.86	0.8
C7_04000W_A	0.66	0	0	0.56	0	0
C2_01990C_A	-0.1	0.5	0.33	-0.12	0.47	0.33
C6_02740W_A	0.14	0.32	0.18	0.3	0.02	0.01
CR_06460W_A	-0.23	0.66	0.51	0.15	0.8	0.71
CR_00990W_A	0.76	0	0	0.93	0	0
C3_02890C_A	0.2	0.65	0.49	-0.35	0.45	0.32
C2_07730W_A	0.08	0.48	0.32	-0.18	0.09	0.04
CR_06310W_A	0.21	0.69	0.55	0.68	0.28	0.17
C4_05740C_A	0	0.98	0.96	0.19	0.13	0.07
CR_03180W_A	-0.27	0.07	0.03	-0.15	0.32	0.21
CR_04060C_A	0.06	0.53	0.37	-0.27	0.01	0
C4_05100C_A	0.31	0	0	0.6	0	0
C1_07550W_A	-0.18	0.13	0.06	-0.25	0.04	0.02
C6_00890W_A	-0.24	0.07	0.03	0.01	0.95	0.92
CR_03540W_A	0.14	0.47	0.3	0.08	0.71	0.59
C6_04030W_A	0.03	0.84	0.74	-0.08	0.62	0.49
CR_03930C_A	-0.22	0.16	0.07	-0.02	0.91	0.85
C4_04950W_A	-0.38	0.43	0.27	0.1	0.87	0.8
C4_06220C_A	0.14	0.66	0.5	0	1	0.99
C1_00970W_A	-0.37	0.3	0.17	-0.18	0.65	0.53
C4_04990C_A	0.07	0.71	0.57	0.12	0.5	0.37
C1_11400C_A	-0.07	0.64	0.48	0.41	0	0
C2_04750W_A	0.54	0.01	0	1.17	0	0
C3_06860C_A	0.3	0	0	0.44	0	0
C3_07350W_A	0.37	0	0	0.49	0	0
C4_05370W_A	0.04	0.85	0.75	-0.18	0.31	0.19

C5_01090C_A	0.01	0.93	0.87	0.05	0.72	0.61
C1_05430W_A	0.14	0.29	0.16	0.14	0.35	0.22
C1_06780W_A	0.03	0.84	0.74	-0.25	0.01	0
C2_04080W_A	-1.26	0	0	-1.17	0	0
CR_09920W_A	0.08	0.83	0.73	0.12	0.73	0.62
C3_07930C_A	0.37	0.46	0.3	0.4	0.56	0.42
C3_02530W_A	1.52	0	0	2.05	0	0
C1_00230C_A	-0.08	0.67	0.52	-0.15	0.39	0.26
C1_07360W_A	0.28	0.05	0.02	0.09	0.62	0.49
C6_02930W_A	-0.33	0	0	-0.33	0	0
C5_03710C_A	0.1	0.82	0.71	0.71	0.03	0.01
C3_03250W_A	-0.33	0.09	0.04	-0.73	0	0
C1_13330C_A	-0.53	0	0	-0.35	0	0
C2_09780C_A	0.69	0	0	0.23	0.04	0.02
CR_02670C_A	0.11	0.55	0.39	-0.06	0.76	0.66
CR_06160C_A	-0.47	0.12	0.05	-0.42	0.19	0.11
CR_00100C_A	0	0.99	0.97	-0.14	0.2	0.11
C3_07750W_A	-0.65	0	0	-0.5	0	0
C4_02250C_A	0.31	0	0	0.45	0	0
C2_04200W_A	-0.71	0	0	-0.65	0	0
C5_01480W_A	-0.64	0.12	0.05	0.09	0.86	0.79
C3_00280C_A	-0.03	0.87	0.78	0.03	0.87	0.81
C2_05360C_A	0.72	0.01	0	1.14	0	0
C6_00480C_A	-0.32	0.53	0.37	-1.69	0	0
C1_03200C_A	-0.01	0.98	0.96	0.08	0.69	0.57
CR_00040C_A	0.44	0	0	0.69	0	0
C3_05430W_A	-0.07	0.78	0.66	-0.06	0.79	0.7
C3_05740C_A	0.16	0.38	0.23	0.38	0.01	0
C4_06900W_A	0.1	0.84	0.74	-0.12	0.86	0.79
C7_00210C_A	0.51	0	0	0.56	0	0
C1_01910W_A	0.08	0.46	0.3	-0.23	0.04	0.02
C3_03430C_A	-0.18	0.46	0.3	-0.75	0	0
CR_08590W_A	0.07	0.62	0.46	0.01	0.96	0.94
C3_00230C_A	0.05	0.85	0.75	-0.13	0.71	0.6
C1_05540C_A	0.06	0.69	0.54	-0.12	0.41	0.28
C1_09360C_A	-0.06	0.67	0.52	0.09	0.47	0.33
CR_02210W_A	-0.49	0	0	-0.32	0.03	0.01
C1_11050W_A	0.36	0.4	0.24	0.42	0.35	0.23
C3_00440W_A	-0.01	0.98	0.95	0.26	0.39	0.26
C1_04300C_A	0.37	0	0	-0.22	0.01	0.01
C2_01060C_A	-0.58	0.09	0.04	0.23	0.54	0.41
C4_04630C_A	-0.19	0.73	0.59	-0.03	0.97	0.95
C5_01050C_A	-0.18	0.03	0.01	-0.26	0	0

CR_05240C_A	0.25	0.03	0.01	0.25	0.03	0.01
C1_04040C_A	-1.15	0	0	-1.27	0	0
CR_00890C_A	-0.06	0.88	0.81	0.11	0.79	0.69
C1_00410C_A	0.14	0.47	0.3	0.32	0.08	0.04
C3_01160W_A	0.05	0.77	0.64	0.25	0.06	0.03
C1_00220W_A	0.66	0	0	0.07	0.47	0.33
C4_05380C_A	0.3	0.37	0.22	0.48	0.13	0.06
C1_13540W_A	-0.03	0.91	0.84	0.04	0.87	0.81
C1_14580C_A	0.13	0.66	0.5	-0.11	0.72	0.61
C2_01150W_A	0.2	0.22	0.11	0.29	0.06	0.03
C1_04270C_A	0.09	0.86	0.77	0.01	0.99	0.98
C1_14380C_A	-0.05	0.79	0.68	-0.02	0.93	0.89
C2_07400C_A	-0.37	0	0	-2.49	0	0
C1_07530W_A	NA	NA	NA	NA	NA	NA
CR_05790C_A	0.39	0	0	0.27	0	0
C3_02570W_A	1.11	0	0	2.03	0	0
C1_01680C_A	-0.42	0	0	-0.21	0.13	0.06
C2_00460W_A	0.26	0.61	0.44	-0.12	0.84	0.76
C2_08770C_A	-0.02	0.98	0.95	0.41	0.26	0.15
CR_03040C_A	-0.04	0.83	0.73	-0.68	0	0
C4_02090C_A	-0.14	0.57	0.41	-0.16	0.52	0.38
C4_00600C_A	0.85	0	0	1.47	0	0
C4_01800W_A	0.51	0	0	0.65	0	0
C7_02260W_A	NA	NA	NA	NA	NA	NA
C1_05380C_A	-0.22	0.19	0.09	0.02	0.91	0.86
C1_05640C_A	-0.52	0	0	-0.04	0.88	0.82
CR_02170W_A	-0.43	0	0	-0.91	0	0
C1_14210C_A	0.73	0	0	1.05	0	0
C1_09390W_A	-0.23	0.46	0.29	-0.19	0.53	0.39
C6_01690W_A	0.43	0	0	0.82	0	0
C1_05990C_A	0.72	0	0	0.8	0	0
C4_03340C_A	0.17	0.7	0.56	0.27	0.53	0.39
C1_02600W_A	-1.51	0	0	-2.46	0	0
C2_03950W_A	-0.15	0.11	0.05	-0.33	0	0
CR_03560W_A	0.17	0.37	0.22	0.09	0.66	0.53
C2_05680W_A	0.2	0.08	0.03	0.29	0.01	0.01
C6_00590W_A	0.09	0.62	0.45	0.24	0.12	0.06
C2_03460C_A	0.03	0.87	0.78	0.15	0.31	0.19
C1_06130C_A	1.57	0	0	1.91	0	0
C6_03260W_A	0.44	0	0	1.58	0	0
C1_08130C_A	0.04	0.86	0.77	0.34	0.05	0.02
C3_04070C_A	0.51	0	0	0.36	0.01	0
C1_12490W_A	-0.04	0.76	0.63	-0.48	0	0

C6_01830W_A	-0.04	0.79	0.68	0.27	0.02	0.01
C5_01580C_A	-0.01	0.93	0.87	-0.53	0	0
CR_09860W_A	-0.14	0.29	0.16	0.06	0.66	0.53
C7_02630W_A	0.12	0.39	0.24	0.31	0.01	0
C1_01000C_A	-0.1	0.54	0.37	-0.19	0.22	0.12
CR_01720W_A	0.19	0.22	0.11	0.17	0.33	0.21
C7_00770W_A	-0.19	0.56	0.39	-0.65	0.03	0.01
C1_00390W_A	-0.06	0.77	0.65	-0.45	0.01	0
C5_05200C_A	-0.31	0	0	-0.87	0	0
C1_09800C_A	0.7	0	0	0.4	0.02	0.01
C7_00750W_A	-0.02	0.94	0.9	0.09	0.6	0.46
CR_05950C_A	0.07	0.62	0.46	-0.2	0.13	0.07
CR_07360W_A	0.03	0.86	0.77	0.17	0.19	0.1
CR_01750C_A	0.2	0.05	0.02	0.29	0	0
C3_06040W_A	0.26	0.57	0.41	0.22	0.68	0.56
C2_09530W_A	0.71	0	0	0.43	0.01	0
C1_12650C_A	0.41	0.03	0.01	0.26	0.22	0.13
C2_00960C_A	0.06	0.59	0.42	0.44	0	0
C2_01400C_A	-0.45	0.02	0.01	-0.32	0.09	0.05
CR_05530C_A	0	0.99	0.97	0.07	0.65	0.52
C6_04620C_A	0.62	0.14	0.06	1	0.09	0.04
C1_08290C_A	-0.8	0	0	-0.8	0	0
C7_01470C_A	0.06	0.61	0.44	0.45	0	0
C5_03600W_A	-0.16	0.21	0.11	-0.19	0.14	0.08
C1_08890C_A	-0.56	0.15	0.07	-0.13	0.79	0.69
CR_04900C_A	0	0.99	0.98	0.84	0.15	0.08
C5_03000C_A	-0.51	0	0	-0.75	0	0
C3_03200C_A	0	1	1	0.2	0.73	0.62
C1_08860C_A	-0.08	0.62	0.46	-0.04	0.83	0.75
C1_07640C_A	-0.72	0.07	0.03	-0.66	0.14	0.07
C2_01200C_A	0.05	0.64	0.48	-0.13	0.25	0.15
C5_02490C_A	-0.1	0.39	0.24	-0.35	0	0
C2_04500W_A	0.03	0.79	0.67	0.07	0.59	0.45
CR_06480C_A	-0.57	0.03	0.01	-0.23	0.49	0.36
C4_00630C_A	1.02	0	0	1.18	0	0
C4_04140W_A	0.2	0.46	0.3	0.7	0	0
CR_06710C_A	-0.17	0.27	0.15	-0.04	0.84	0.77
C4_01880W_A	0.33	0	0	0.35	0	0
CR_06570C_A	0.28	0	0	0.27	0.01	0
C5_02700W_A	0.52	0	0	0.56	0	0
C3_00730W_A	0.02	0.93	0.87	-0.06	0.69	0.57
C4_03760W_A	-0.27	0.61	0.44	-0.23	0.75	0.64
C7_01880C_A	-0.48	0.23	0.12	-0.19	0.68	0.55

C5_03880C_A	0.23	0.68	0.53	0.24	0.71	0.59
C1_09920W_A	0.09	0.66	0.51	0.17	0.38	0.26
CR_08520C_A	-0.23	0.44	0.28	-0.18	0.55	0.41
C7_00920C_A	0.24	0.21	0.1	0.37	0.05	0.02
C4_02040W_A	0.35	0.01	0	0.69	0	0
C1_08730W_A	0.35	0	0	0.29	0.02	0.01
C7_04280C_A	0.14	0.12	0.05	0.27	0.01	0.01
C4_03900C_A	-0.21	0.43	0.27	0.07	0.81	0.73
C5_01670W_A	0	1	0.99	0.1	0.88	0.83
C4_04780W_A	0.42	0	0	-0.25	0.01	0
C3_07270C_A	0.03	0.77	0.64	0.12	0.24	0.14
C1_01810C_A	0.54	0	0	0.63	0	0
C3_04320W_A	-0.35	0.05	0.02	-0.23	0.23	0.13
C4_02880C_A	-0.32	0.03	0.01	-0.31	0.04	0.02
C3_07650C_A	-0.28	0.22	0.11	0.01	0.99	0.97
C7_02450W_A	0.31	0.01	0	0.38	0	0
C4_06840W_A	0	0.99	0.98	0.23	0.22	0.13
C5_03480C_A	0.32	0.01	0	0.21	0.14	0.07
C1_01450W_A	0.34	0	0	0.47	0	0
C1_11930W_A	-0.01	0.98	0.95	0.08	0.57	0.43
C1_04510W_A	-0.01	0.98	0.96	0.29	0.19	0.11
C1_10240C_A	1.08	0	0	2.06	0	0
C2_08100W_A	0.08	0.37	0.22	-0.16	0.09	0.04
C5_02560C_A	-0.06	0.73	0.59	0.03	0.88	0.82
C4_03360C_A	0.24	0.01	0	0.02	0.86	0.79
C2_02460W_A	0.05	0.62	0.46	-0.24	0.01	0
C3_05010C_A	0.08	0.72	0.58	0.05	0.83	0.75
C7_00720W_A	-0.23	0.06	0.02	-0.03	0.87	0.8
C6_01520W_A	0.09	0.79	0.68	-0.47	0.13	0.07
C3_00640W_A	-0.02	0.91	0.84	0.27	0.01	0
C3_06010W_A	0.2	0	0	-0.21	0.02	0.01
C7_03570W_A	0.72	0	0	1.37	0	0
C3_06970W_A	-0.2	0.06	0.02	-0.12	0.42	0.29
C1_06090C_A	-0.1	0.62	0.45	-0.14	0.47	0.33
C2_03180C_A	0.14	0.11	0.05	-0.08	0.36	0.23
C3_03860W_A	0.27	0.07	0.03	0.21	0.24	0.14
C3_01080W_A	-0.48	0.11	0.05	-0.38	0.19	0.11
C4_00580W_A	0.01	0.98	0.95	-0.17	0.41	0.28
C1_05560W_A	0.39	0	0	0.4	0	0
C5_01020C_A	-0.57	0.22	0.11	-0.17	0.78	0.69
C4_02920W_A	-0.19	0.17	0.08	-0.33	0.01	0.01
C2_03840C_A	-0.14	0.63	0.47	0.06	0.83	0.75
C2_09600C_A	0.13	0.56	0.39	0.06	0.79	0.69

C2_00280C_A	-0.07	0.85	0.75	-0.11	0.75	0.65
CR_07560W_A	0.72	0	0	1	0	0
C2_06940C_A	0.52	0	0	0.54	0	0
C3_00500C_A	0.01	0.97	0.95	-0.02	0.93	0.89
C3_05900W_A	-0.35	0.32	0.18	0.16	0.65	0.52
C3_00770C_A	0.06	0.57	0.41	0.24	0.01	0
C1_14020W_A	0.35	0.45	0.29	0.55	0.39	0.26
C4_00330C_A	0.4	0.03	0.01	0.88	0	0
C1_13300C_A	0.12	0.14	0.06	0.25	0	0
C5_04640C_A	-0.28	0.02	0.01	-0.21	0.18	0.1
C1_11680C_A	-0.28	0.54	0.38	-0.17	0.74	0.63
C4_04170C_A	0.14	0.32	0.18	0.02	0.91	0.86
C1_00840C_A	-0.45	0	0	-0.42	0	0
C3_00160C_A	0.21	0.11	0.04	0.41	0	0
C3_04880W_A	0.47	0	0	0.8	0	0
C2_06930C_A	0.05	0.93	0.88	0.04	0.96	0.94
C1_01610C_A	0.2	0.62	0.46	0.26	0.52	0.38
CR_00250W_A	0.36	0	0	0.63	0	0
C3_05860C_A	-0.2	0.4	0.24	-0.4	0.06	0.03
CR_03300C_A	0.04	0.95	0.9	-0.02	0.97	0.96
C3_05380W_A	-0.49	0.01	0	-0.48	0.02	0.01
C1_14040W_A	0.04	0.77	0.64	-0.13	0.28	0.18
C3_03620C_A	NA	NA	NA	NA	NA	NA
C2_08930W_A	0.28	0	0	0.43	0	0
C6_04250W_A	0.24	0.04	0.01	0.22	0.08	0.04
C6_00540W_A	-0.11	0.56	0.4	-0.09	0.65	0.52
C1_14440C_A	0.59	0	0	0.64	0	0
C1_05870W_A	-0.07	0.8	0.69	-0.39	0.07	0.03
C4_05060W_A	0.27	0.06	0.02	0.38	0.01	0
C4_06020C_A	-0.17	0.33	0.19	-0.05	0.8	0.71
CR_04360C_A	-0.55	0	0	-0.92	0	0
C1_14250C_A	0.05	0.75	0.62	-0.15	0.28	0.17
C2_06540C_A	0.29	0	0	0.11	0.31	0.2
C6_00980C_A	0.16	0.46	0.29	-0.29	0.18	0.1
C4_06950W_A	0.03	0.95	0.92	-0.54	0.12	0.06
C1_10210C_A	0.02	0.9	0.82	-0.24	0.05	0.02
C2_02710C_A	-0.18	0.28	0.15	0.09	0.56	0.43
C1_04210C_A	0.45	0	0	0.53	0	0
C6_04380W_A	1.15	0	0	-1.01	0	0
C3_01520C_A	-0.5	0.03	0.01	-0.21	0.19	0.11
C5_04490C_A	-0.06	0.77	0.64	-0.47	0	0
C2_06490W_A	0.28	0.02	0.01	0.41	0	0
C4_04240C_A	0.27	0.01	0	0.45	0	0

C5_04620C_A	0	0.99	0.97	-0.07	0.71	0.6
C1_14460W_A	0.44	0	0	0.41	0	0
CR_08950W_A	0.28	0	0	0.38	0	0
C2_10310C_A	-1.15	0	0	-1.05	0	0
C2_05940C_A	0.26	0.12	0.05	0.52	0	0
C7_03530C_A	-0.46	0.1	0.04	-0.14	0.67	0.54
C4_04500C_A	0.03	0.86	0.76	-0.73	0	0
C3_01960C_A	0.23	0.02	0.01	0.33	0	0
C1_04580C_A	-0.11	0.54	0.37	-0.4	0.01	0
C2_10300C_A	0.14	0.65	0.49	0.25	0.35	0.23
CR_02440W_A	-0.03	0.97	0.94	-0.43	0.48	0.34
C5_03320C_A	-0.29	0.45	0.29	0.24	0.51	0.37
C2_09060C_A	-0.11	0.13	0.06	-0.62	0	0
C7_02310C_A	0.28	0.32	0.18	0.54	0.03	0.01
C7_00440C_A	0.29	0	0	0.04	0.74	0.64
C2_04940C_A	-0.03	0.93	0.88	-0.13	0.68	0.56
BGI_novel_G000[illegible]	0.19	0.52	0.35	0.23	0.41	0.28
C7_01740C_A	0.06	0.78	0.66	0.26	0.13	0.07
CR_03690W_A	0.01	0.95	0.91	0.06	0.71	0.59
C3_07690C_A	-0.11	0.47	0.31	-0.1	0.47	0.33
C1_06410W_A	-0.16	0.52	0.35	-0.01	0.97	0.94
C6_02070C_A	-0.19	0	0	-0.62	0	0
C3_00100W_A	-0.01	0.98	0.96	0.01	0.96	0.94
C1_03910C_A	-0.32	0.18	0.08	0.44	0.06	0.03
C3_03380W_A	-0.32	0.01	0	-0.26	0.05	0.02
CR_02110W_A	-0.14	0.28	0.15	0.14	0.22	0.13
C3_02340W_A	0.03	0.95	0.9	0.25	0.39	0.26
C1_03050W_A	0.15	0.23	0.12	0.22	0.06	0.03
C2_08820C_A	-0.46	0.21	0.1	-0.63	0.29	0.18
C6_03320W_A	-0.42	0	0	-0.39	0	0
C3_05680W_A	-0.06	0.77	0.65	0	0.99	0.99
C3_07220C_A	0.04	0.78	0.66	0.21	0.06	0.03
C3_03870C_A	1.22	0	0	0.85	0	0
C1_09670C_A	0.19	0.28	0.15	0.26	0.13	0.06
CR_03260W_A	-0.11	0.7	0.55	0.24	0.31	0.2
C5_02170C_A	0.09	0.31	0.17	-0.14	0.08	0.04
