# Supplementary material for: Tunicamycin Potentiates Antifungal Drug Tolerance via Aneuploidy in Candida albicans
Source: mBio. 2021 Aug 31;12(4):e02272-21. doi: 10.1128/mBio.02272-21 (PMC8406271; doi:10.1128/mBio.02272-21)
Supplement: TABLE S4 [file mbio.02272-21-st004.pdf]

Table S4. Gene ontology enrichment of genes differentially expressed in sub-MIC and supraMIC TUN concentrations

Genes induced by 1 μg/ml of tunicamycin

GOID	GO_term	Corrected P-value
51234	establishment of localization	1.43E-22
16192	vesicle-mediated transport	2.40E-22
6810	transport	1.92E-21
51179	localization	3.09E-21
48193	Golgi vesicle transport	5.25E-20
44281	small molecule metabolic process	8.95E-19
1901135	carbohydrate derivative metabolic process	9.76E-14
9056	catabolic process	1.62E-13
6508	proteolysis	8.29E-13
1901564	organonitrogen compound metabolic process	3.33E-12
1901565	organonitrogen compound catabolic process	4.36E-12
44248	cellular catabolic process	9.46E-12
9987	cellular process	3.04E-11
1901575	organic substance catabolic process	4.30E-11
44257	cellular protein catabolic process	1.21E-10
51603	proteolysis involved in cellular protein catabolic process	2.10E-10
30163	protein catabolic process	2.34E-10
10498	proteasomal protein catabolic process	4.45E-10
44283	small molecule biosynthetic process	8.76E-10
19941	modification-dependent protein catabolic process	9.05E-10
6511	ubiquitin-dependent protein catabolic process	9.05E-10
43161	proteasome-mediated ubiquitin-dependent protein catabolic process	1.40E-09
1901137	carbohydrate derivative biosynthetic process	3.55E-09
9100	glycoprotein metabolic process	9.96E-09
43632	modification-dependent macromolecule catabolic process	1.22E-08

6888	endoplasmic reticulum to Golgi vesicle-mediated transport	1.41E-08
6890	retrograde vesicle-mediated transport, Golgi to endoplasmic reticulum	5.44E-08
71852	fungal-type cell wall organization or biogenesis	6.44E-08
1902652	secondary alcohol metabolic process	6.95E-08
44255	cellular lipid metabolic process	8.39E-08
6811	ion transport	9.81E-08
71554	cell wall organization or biogenesis	1.08E-07
30036	actin cytoskeleton organization	1.10E-07
46165	alcohol biosynthetic process	1.11E-07
19538	protein metabolic process	1.18E-07
6066	alcohol metabolic process	1.39E-07
16126	sterol biosynthetic process	2.03E-07
16128	phytosteroid metabolic process	2.03E-07
44107	cellular alcohol metabolic process	2.03E-07
6694	steroid biosynthetic process	2.03E-07
8204	ergosterol metabolic process	2.03E-07
43413	macromolecule glycosylation	2.09E-07
6486	protein glycosylation	2.09E-07
70085	glycosylation	2.09E-07
16129	phytosteroid biosynthetic process	2.57E-07
1902653	secondary alcohol biosynthetic process	2.57E-07
44108	cellular alcohol biosynthetic process	2.57E-07
6696	ergosterol biosynthetic process	2.57E-07
97384	cellular lipid biosynthetic process	2.57E-07
9057	macromolecule catabolic process	3.44E-07
44265	cellular macromolecule catabolic process	3.72E-07
6892	post-Golgi vesicle-mediated transport	3.73E-07
1901617	organic hydroxy compound biosynthetic process	6.04E-07
30029	actin filament-based process	6.12E-07
8152	metabolic process	7.77E-07
9101	glycoprotein biosynthetic process	9.06E-07
16125	sterol metabolic process	1.19E-06
6629	lipid metabolic process	1.49E-06
1901615	organic hydroxy compound metabolic process	1.88E-06
8202	steroid metabolic process	2.05E-06

30447	filamentous growth	2.34E-06
8610	lipid biosynthetic process	2.41E-06
40007	growth	3.60E-06
36503	ERAD pathway	4.56E-06
51641	cellular localization	7.22E-06
30448	hyphal growth	7.60E-06
6820	anion transport	9.29E-06
6896	Golgi to vacuole transport	9.41E-06
5975	carbohydrate metabolic process	9.53E-06
71702	organic substance transport	1.02E-05
44267	cellular protein metabolic process	1.39E-05
8643	carbohydrate transport	1.44E-05
43254	regulation of protein-containing complex assembly	1.96E-05
34976	response to endoplasmic reticulum stress	2.76E-05
1901698	response to nitrogen compound	3.14E-05
30433	ubiquitin-dependent ERAD pathway	4.99E-05
6082	organic acid metabolic process	7.82E-05
110053	regulation of actin filament organization	8.92E-05
19637	organophosphate metabolic process	8.93E-05
7010	cytoskeleton organization	0.00013
31505	fungal-type cell wall organization	0.00014
45229	external encapsulating structure organization	0.00018
71555	cell wall organization	0.00018
43436	oxoacid metabolic process	0.00021
44419	biological process involved in interspecies interaction between organisms	0.00021
7015	actin filament organization	0.00028
51649	establishment of localization in cell	0.00032
44237	cellular metabolic process	0.00035
1902903	regulation of supramolecular fiber organization	0.00036
42546	cell wall biogenesis	0.00041
51301	cell division	0.00049
32940	secretion by cell	0.00049
46903	secretion	0.00049
6793	phosphorus metabolic process	0.00054
19752	carboxylic acid metabolic process	0.00055

6091	generation of precursor metabolites and energy	0.00063
140352	export from cell	0.00084
48284	organelle fusion	0.00104
10243	response to organonitrogen compound	0.00119
44089	positive regulation of cellular component biogenesis	0.0013
44182	filamentous growth of a population of unicellular organisms	0.00133
32535	regulation of cellular component size	0.00146
90066	regulation of anatomical structure size	0.00146
46907	intracellular transport	0.00154
70887	cellular response to chemical stimulus	0.00158
36211	protein modification process	0.00162
6464	cellular protein modification process	0.00162
30832	regulation of actin filament length	0.00166
30833	regulation of actin filament polymerization	0.00166
8064	regulation of actin polymerization or depolymerization	0.00166
6796	phosphate-containing compound metabolic process	0.00191
46034	ATP metabolic process	0.00216
32271	regulation of protein polymerization	0.00239
70972	protein localization to endoplasmic reticulum	0.00239
51716	cellular response to stimulus	0.00363
9272	fungal-type cell wall biogenesis	0.00364
32956	regulation of actin cytoskeleton organization	0.00391
32970	regulation of actin filament-based process	0.00391
90407	organophosphate biosynthetic process	0.00578
50896	response to stimulus	0.00596
6487	protein N-linked glycosylation	0.00668
44264	cellular polysaccharide metabolic process	0.00676
44087	regulation of cellular component biogenesis	0.00686
9250	glucan biosynthetic process	0.00805
60627	regulation of vesicle-mediated transport	0.00857
44262	cellular carbohydrate metabolic process	0.00917
30866	cortical actin cytoskeleton organization	0.00922
61024	membrane organization	0.00923

15980	energy derivation by oxidation of organic compounds	0.00943
5976	polysaccharide metabolic process	0.01005
97435	supramolecular fiber organization	0.01005
34637	cellular carbohydrate biosynthetic process	0.01087
46467	membrane lipid biosynthetic process	0.0117
9247	glycolipid biosynthetic process	0.01359
42221	response to chemical	0.01395
16051	carbohydrate biosynthetic process	0.01473
30865	cortical cytoskeleton organization	0.01548
55086	nucleobase-containing small molecule metabolic process	0.01582
7033	vacuole organization	0.01666
61640	cytoskeleton-dependent cytokinesis	0.01795
61919	process utilizing autophagic mechanism	0.02038
15985	energy coupled proton transport, down electrochemical gradient	0.02419
15986	ATP synthesis coupled proton transport	0.02419
6099	tricarboxylic acid cycle	0.02419
7034	vacuolar transport	0.02538
31334	positive regulation of protein-containing complex assembly	0.02544
1902905	positive regulation of supramolecular fiber organization	0.028
44396	actin cortical patch organization	0.02883
9145	purine nucleoside triphosphate biosynthetic process	0.02883
9205	purine ribonucleoside triphosphate metabolic process	0.02883
9206	purine ribonucleoside triphosphate biosynthetic process	0.02883
9607	response to biotic stimulus	0.03564
9117	nucleotide metabolic process	0.03635
6887	exocytosis	0.03852
6897	endocytosis	0.03893
44282	small molecule catabolic process	0.0402
72599	establishment of protein localization to endoplasmic reticulum	0.04039
281	mitotic cytokinesis	0.04207
1902600	proton transmembrane transport	0.04574

1903509	liposaccharide metabolic process	0.04633
6664	glycolipid metabolic process	0.04633
8652	cellular amino acid biosynthetic process	0.05258
9199	ribonucleoside triphosphate metabolic process	0.05584
9201	ribonucleoside triphosphate biosynthetic process	0.05584
6914	autophagy	0.05808
70787	conidiophore development	0.06157
33692	cellular polysaccharide biosynthetic process	0.06299
1901607	alpha-amino acid biosynthetic process	0.06488
9150	purine ribonucleotide metabolic process	0.06488
51493	regulation of cytoskeleton organization	0.06629
72521	purine-containing compound metabolic process	0.06671
44036	cell wall macromolecule metabolic process	0.06735
6506	GPI anchor biosynthetic process	0.06783
6620	posttranslational protein targeting to endoplasmic reticulum membrane	0.06974
31204	posttranslational protein targeting to membrane, translocation	0.07213
6643	membrane lipid metabolic process	0.07259
6520	cellular amino acid metabolic process	0.07546
1901605	alpha-amino acid metabolic process	0.07601
6661	phosphatidylinositol biosynthetic process	0.07916
71704	organic substance metabolic process	0.07971
35966	response to topologically incorrect protein	0.08057
51130	positive regulation of cellular component organization	0.08202
44042	glucan metabolic process	0.08273
6073	cellular glucan metabolic process	0.08273
6163	purine nucleotide metabolic process	0.08452
51666	actin cortical patch localization	0.0876
51274	beta-glucan biosynthetic process	0.08974
271	polysaccharide biosynthetic process	0.09619
1901136	carbohydrate derivative catabolic process	0.09911

Genes induced by 4 μg/ml of tunicamycin

GOID	GO_term	Corrected P-value
16192	vesicle-mediated transport	5.66E-29
48193	Golgi vesicle transport	2.12E-26
51234	establishment of localization	1.61E-19
6810	transport	1.87E-18
51179	localization	2.56E-18
9056	catabolic process	4.62E-15
44248	cellular catabolic process	1.00E-14
44257	cellular protein catabolic process	1.05E-13
30163	protein catabolic process	1.31E-13
51603	proteolysis involved in cellular protein catabolic process	2.56E-13
19941	modification-dependent protein catabolic process	4.34E-13
6511	ubiquitin-dependent protein catabolic process	4.34E-13
6508	proteolysis	6.65E-13
1901565	organonitrogen compound catabolic process	1.24E-12
6888	endoplasmic reticulum to Golgi vesicle-mediated transport	2.56E-12
10498	proteasomal protein catabolic process	7.17E-12
30036	actin cytoskeleton organization	9.74E-12
43161	proteasome-mediated ubiquitin-dependent protein catabolic process	1.03E-11
43632	modification-dependent macromolecule catabolic process	1.19E-11
30029	actin filament-based process	2.16E-11
1901575	organic substance catabolic process	6.03E-10
6892	post-Golgi vesicle-mediated transport	1.54E-09
71852	fungal-type cell wall organization or biogenesis	2.32E-09
71554	cell wall organization or biogenesis	4.47E-09
9057	macromolecule catabolic process	4.49E-09
7163	establishment or maintenance of cell polarity	5.30E-09
44265	cellular macromolecule catabolic process	6.49E-09
32535	regulation of cellular component size	2.20E-08

90066	regulation of anatomical structure size	2.20E-08
7010	cytoskeleton organization	2.87E-08
110053	regulation of actin filament organization	3.02E-08
51301	cell division	3.90E-08
43254	regulation of protein-containing complex assembly	7.34E-08
6890	retrograde vesicle-mediated transport, Golgi to endoplasmic reticulum	8.54E-08
6896	Golgi to vacuole transport	2.35E-07
70887	cellular response to chemical stimulus	2.56E-07
44089	positive regulation of cellular component biogenesis	2.87E-07
32956	regulation of actin cytoskeleton organization	3.64E-07
32970	regulation of actin filament-based process	3.64E-07
40007	growth	9.29E-07
30447	filamentous growth	9.44E-07
44087	regulation of cellular component biogenesis	1.01E-06
51641	cellular localization	1.02E-06
1902903	regulation of supramolecular fiber organization	1.06E-06
30832	regulation of actin filament length	2.32E-06
30833	regulation of actin filament polymerization	2.32E-06
8064	regulation of actin polymerization or depolymerization	2.32E-06
51649	establishment of localization in cell	2.47E-06
23052	signaling	2.72E-06
7165	signal transduction	4.67E-06
10499	proteasomal ubiquitin-independent protein catabolic process	6.22E-06
46907	intracellular transport	8.32E-06
7034	vacuolar transport	8.70E-06
42221	response to chemical	1.00E-05
1901135	carbohydrate derivative metabolic process	1.41E-05
32271	regulation of protein polymerization	1.71E-05
61919	process utilizing autophagic mechanism	1.72E-05
910	cytokinesis	1.85E-05
6897	endocytosis	1.89E-05
44281	small molecule metabolic process	1.90E-05
16050	vesicle organization	2.25E-05
34976	response to endoplasmic reticulum stress	2.81E-05

61640	cytoskeleton-dependent cytokinesis	2.92E-05
7015	actin filament organization	3.94E-05
51493	regulation of cytoskeleton organization	4.43E-05
51716	cellular response to stimulus	4.68E-05
61024	membrane organization	4.89E-05
51130	positive regulation of cellular component organization	6.02E-05
1902905	positive regulation of supramolecular fiber organization	6.08E-05
31334	positive regulation of protein-containing complex assembly	6.36E-05
281	mitotic cytokinesis	7.42E-05
48284	organelle fusion	7.42E-05
6914	autophagy	7.79E-05
35556	intracellular signal transduction	8.04E-05
31505	fungal-type cell wall organization	8.27E-05
61025	membrane fusion	8.88E-05
8643	carbohydrate transport	0.0001
45229	external encapsulating structure organization	0.0001
71555	cell wall organization	0.0001
51495	positive regulation of cytoskeleton organization	0.00011
6811	ion transport	0.00014
1901137	carbohydrate derivative biosynthetic process	0.00016
30838	positive regulation of actin filament polymerization	0.00017
71702	organic substance transport	0.00021
90174	organelle membrane fusion	0.00028
5975	carbohydrate metabolic process	0.00033
34727	piecemeal microautophagy of the nucleus	0.00033
30448	hyphal growth	0.00038
7154	cell communication	0.00039
16237	lysosomal microautophagy	0.0004
43248	proteasome assembly	0.00041
44396	actin cortical patch organization	0.00044
9145	purine nucleoside triphosphate biosynthetic process	0.00044
9205	purine ribonucleoside triphosphate metabolic process	0.00044

9206	purine ribonucleoside triphosphate biosynthetic process	0.00044
44804	autophagy of nucleus	0.00051
6820	anion transport	0.00052
50896	response to stimulus	0.00065
43413	macromolecule glycosylation	0.00069
6486	protein glycosylation	0.00069
70085	glycosylation	0.00069
30866	cortical actin cytoskeleton organization	0.00083
44182	filamentous growth of a population of unicellular organisms	0.00084
62197	cellular response to chemical stress	0.00086
36503	ERAD pathway	0.0009
32940	secretion by cell	0.00093
46903	secretion	0.00093
60627	regulation of vesicle-mediated transport	0.00105
51128	regulation of cellular component organization	0.0011
6891	intra-Golgi vesicle-mediated transport	0.00117
9199	ribonucleoside triphosphate metabolic process	0.00117
9201	ribonucleoside triphosphate biosynthetic process	0.00117
34614	cellular response to reactive oxygen species	0.00135
30433	ubiquitin-dependent ERAD pathway	0.00142
32506	cytokinetic process	0.00142
9100	glycoprotein metabolic process	0.00157
6754	ATP biosynthetic process	0.00159
30865	cortical cytoskeleton organization	0.00162
51049	regulation of transport	0.0018
6906	vesicle fusion	0.00198
15985	energy coupled proton transport, down electrochemical gradient	0.00208
15986	ATP synthesis coupled proton transport	0.00208
9101	glycoprotein biosynthetic process	0.00229
45010	actin nucleation	0.00269
1901071	glucosamine-containing compound metabolic process	0.00286
9144	purine nucleoside triphosphate metabolic process	0.00286

40008	regulation of growth	0.003
32273	positive regulation of protein polymerization	0.00434
97435	supramolecular fiber organization	0.00447
1901698	response to nitrogen compound	0.00626
10033	response to organic substance	0.00643
42546	cell wall biogenesis	0.0069
10638	positive regulation of organelle organization	0.00785
7033	vacuole organization	0.00884
1902407	assembly of actomyosin apparatus involved in mitotic cytokinesis	0.00985
1903475	mitotic actomyosin contractile ring assembly	0.00985
912	assembly of actomyosin apparatus involved in cytokinesis	0.00985
915	actomyosin contractile ring assembly	0.00985
6950	response to stress	0.01015
44264	cellular polysaccharide metabolic process	0.01099
34599	cellular response to oxidative stress	0.0111
44262	cellular carbohydrate metabolic process	0.01146
32879	regulation of localization	0.01222
10570	regulation of filamentous growth	0.01328
6040	amino sugar metabolic process	0.01359
9142	nucleoside triphosphate biosynthetic process	0.01359
72593	reactive oxygen species metabolic process	0.01375
32505	reproduction of a single-celled organism	0.01456
5976	polysaccharide metabolic process	0.01684
1900428	regulation of filamentous growth of a population of unicellular organisms	0.01735
6022	aminoglycan metabolic process	0.0196
6030	chitin metabolic process	0.0196
48278	vesicle docking	0.02051
302	response to reactive oxygen species	0.02097
19954	asexual reproduction	0.02218
140352	export from cell	0.02406
30010	establishment of cell polarity	0.02407
9272	fungal-type cell wall biogenesis	0.02505
1902410	mitotic cytokinetic process	0.02704
1901615	organic hydroxy compound metabolic process	0.0313
6066	alcohol metabolic process	0.03211
32502	developmental process	0.03373

33554	cellular response to stress	0.0338
6620	posttranslational protein targeting to endoplasmic reticulum membrane	0.03658
6979	response to oxidative stress	0.04085
70972	protein localization to endoplasmic reticulum	0.04381
32231	regulation of actin filament bundle assembly	0.04651
160	phosphorelay signal transduction system	0.05332
3	reproduction	0.05494
35967	cellular response to topologically incorrect protein	0.06924
46034	ATP metabolic process	0.07616
16482	cytosolic transport	0.07628
6887	exocytosis	0.07867
9141	nucleoside triphosphate metabolic process	0.09258
34315	regulation of Arp2/3 complex-mediated actin nucleation	0.09539
6037	cell wall chitin metabolic process	0.09539
31032	actomyosin structure organization	0.09716
44837	actomyosin contractile ring organization	0.09716
36211	protein modification process	0.09775
6464	cellular protein modification process	0.09775
35966	response to topologically incorrect protein	0.09822
10035	response to inorganic substance	0.09881

Genes repressed by 1 µg/ml of tunicamycin

GOID	GO_term	Corrected P-value
22613	ribonucleoprotein complex biogenesis	6.15E-61
42254	ribosome biogenesis	6.38E-61
6364	rRNA processing	1.11E-54
34470	ncRNA processing	1.29E-54
16072	rRNA metabolic process	2.11E-53
6396	RNA processing	6.15E-49
34660	ncRNA metabolic process	6.73E-49
42274	ribosomal small subunit biogenesis	9.30E-47

30490	maturation of SSU-rRNA	2.80E-46
462	maturation of SSU-rRNA from tricistronic rRNA transcript (SSU-rRNA, 5.8S rRNA, LSU-rRNA)	1.68E-45
16070	RNA metabolic process	7.06E-41
44085	cellular component biogenesis	1.48E-39
1901360	organic cyclic compound metabolic process	1.46E-37
6725	cellular aromatic compound metabolic process	3.10E-34
6139	nucleobase-containing compound metabolic process	4.46E-34
46483	heterocycle metabolic process	5.29E-33
90304	nucleic acid metabolic process	2.30E-32
469	cleavage involved in rRNA processing	5.64E-30
42273	ribosomal large subunit biogenesis	5.64E-30
10467	gene expression	1.30E-28
90501	RNA phosphodiester bond hydrolysis	2.25E-28
34641	cellular nitrogen compound metabolic process	4.85E-28
478	endonucleolytic cleavage involved in rRNA processing	1.39E-27
479	endonucleolytic cleavage of tricistronic rRNA transcript (SSU-rRNA, 5.8S rRNA, LSU-rRNA)	1.39E-27
90502	RNA phosphodiester bond hydrolysis, endonucleolytic	1.89E-27
90305	nucleic acid phosphodiester bond hydrolysis	4.14E-26
966	RNA 5'-end processing	3.04E-25
967	rRNA 5'-end processing	5.77E-25
34471	ncRNA 5'-end processing	7.86E-25
42255	ribosome assembly	1.64E-24
472	endonucleolytic cleavage to generate mature 5'-end of SSU-rRNA from (SSU-rRNA, 5.8S rRNA, LSU-rRNA)	3.82E-24
27	ribosomal large subunit assembly	1.15E-22
71826	ribonucleoprotein complex subunit organization	1.31E-18
22618	ribonucleoprotein complex assembly	3.25E-18
71840	cellular component organization or biogenesis	4.68E-17
70925	organelle assembly	3.52E-16

460	maturation of 5.8S rRNA	9.88E-16
466	maturation of 5.8S rRNA from tricistronic rRNA transcript (SSU-rRNA, 5.8S rRNA, LSU-rRNA)	9.88E-16
71704	organic substance metabolic process	1.62E-14
6807	nitrogen compound metabolic process	5.74E-14
480	endonucleolytic cleavage in 5'-ETS of tricistronic rRNA transcript (SSU-rRNA, 5.8S rRNA, LSU-rRNA)	5.86E-14
44238	primary metabolic process	9.21E-14
9987	cellular process	2.05E-12
463	maturation of LSU-rRNA from tricistronic rRNA transcript (SSU-rRNA, 5.8S rRNA, LSU-rRNA)	2.38E-12
470	maturation of LSU-rRNA	3.61E-12
44237	cellular metabolic process	9.63E-11
43170	macromolecule metabolic process	9.95E-10
8152	metabolic process	8.49E-09
34622	cellular protein-containing complex assembly	2.14E-07
447	endonucleolytic cleavage in ITS1 to separate SSU-rRNA from 5.8S rRNA and LSU-rRNA from tricistronic rRNA transcript (SSU-rRNA, 5.8S rRNA, LSU-rRNA)	5.44E-07
65003	protein-containing complex assembly	1.33E-06
43933	protein-containing complex subunit organization	4.72E-06
33750	ribosome localization	1.72E-05
54	ribosomal subunit export from nucleus	1.72E-05
71428	rRNA-containing ribonucleoprotein complex export from nucleus	2.40E-05
6403	RNA localization	2.56E-05
51169	nuclear transport	9.02E-05
6913	nucleocytoplasmic transport	9.02E-05
22607	cellular component assembly	9.87E-05
71166	ribonucleoprotein complex localization	0.0001
71426	ribonucleoprotein complex export from nucleus	0.0001
6405	RNA export from nucleus	0.00013
6611	protein export from nucleus	0.00013

9126	purine nucleoside monophosphate metabolic process	0.00014
9127	purine nucleoside monophosphate biosynthetic process	0.00014
9167	purine ribonucleoside monophosphate metabolic process	0.00014
9168	purine ribonucleoside monophosphate biosynthetic process	0.00014
51168	nuclear export	0.00016
50658	RNA transport	0.00019
51236	establishment of RNA localization	0.00019
96	sulfur amino acid metabolic process	0.0002
9069	serine family amino acid metabolic process	0.0002
50657	nucleic acid transport	0.00024
9124	nucleoside monophosphate biosynthetic process	0.0005
9451	RNA modification	0.00057
9113	purine nucleobase biosynthetic process	0.00076
6189	'de novo' IMP biosynthetic process	0.00084
9123	nucleoside monophosphate metabolic process	0.00117
55	ribosomal large subunit export from nucleus	0.00194
9156	ribonucleoside monophosphate biosynthetic process	0.00381
9161	ribonucleoside monophosphate metabolic process	0.00381
31503	protein-containing complex localization	0.00631
32259	methylation	0.00797
1510	RNA methylation	0.01301
51656	establishment of organelle localization	0.01845
6555	methionine metabolic process	0.01892
43414	macromolecule methylation	0.01918
46112	nucleobase biosynthetic process	0.01966
46040	IMP metabolic process	0.02808
46083	adenine metabolic process	0.02808
6188	IMP biosynthetic process	0.02808
71035	nuclear polyadenylation-dependent rRNA catabolic process	0.0293
15931	nucleobase-containing compound transport	0.03367

43633	polyadenylation-dependent RNA catabolic process	0.03572
1902626	assembly of large subunit precursor of preribosome	0.03676
46084	adenine biosynthetic process	0.03676
154	rRNA modification	0.03781
9112	nucleobase metabolic process	0.04885
9303	rRNA transcription	0.04885
97	sulfur amino acid biosynthetic process	0.05739
1901362	organic cyclic compound biosynthetic process	0.06822
9070	serine family amino acid biosynthetic process	0.07266
42440	pigment metabolic process	0.07853
6534	cysteine metabolic process	0.08855

Genes repressed by 4 μg/ml of tunicamycin

GOID	GO_term	Corrected P-value
10467	gene expression	1.03E-30
22613	ribonucleoprotein complex biogenesis	1.76E-30
42254	ribosome biogenesis	2.02E-30
42255	ribosome assembly	4.97E-29
9987	cellular process	5.68E-28
34641	cellular nitrogen compound metabolic process	1.35E-27
34660	ncRNA metabolic process	4.96E-25
27	ribosomal large subunit assembly	9.36E-24
6364	rRNA processing	5.08E-23
34470	ncRNA processing	7.64E-23
16072	rRNA metabolic process	4.06E-22
71704	organic substance metabolic process	2.45E-21
71826	ribonucleoprotein complex subunit organization	4.59E-21
22618	ribonucleoprotein complex assembly	7.04E-21
1901360	organic cyclic compound metabolic process	2.17E-20
44085	cellular component biogenesis	4.03E-20

6396	RNA processing	5.03E-20
70925	organelle assembly	9.65E-20
44238	primary metabolic process	1.02E-19
42274	ribosomal small subunit biogenesis	1.41E-18
9058	biosynthetic process	3.47E-18
1901566	organonitrogen compound biosynthetic process	4.85E-18
44249	cellular biosynthetic process	5.73E-18
30490	maturation of SSU-rRNA	6.01E-18
1901576	organic substance biosynthetic process	7.54E-18
6807	nitrogen compound metabolic process	1.19E-17
462	maturation of SSU-rRNA from tricistronic rRNA transcript (SSU-rRNA, 5.8S rRNA, LSU-rRNA)	4.01E-17
43604	amide biosynthetic process	7.29E-17
43043	peptide biosynthetic process	1.52E-16
16070	RNA metabolic process	1.96E-16
44237	cellular metabolic process	3.32E-16
6412	translation	8.76E-16
43603	cellular amide metabolic process	1.47E-15
44271	cellular nitrogen compound biosynthetic process	1.93E-15
8152	metabolic process	3.94E-15
6518	peptide metabolic process	4.04E-15
6725	cellular aromatic compound metabolic process	1.19E-14
6139	nucleobase-containing compound metabolic process	1.92E-13
46483	heterocycle metabolic process	4.37E-13
42273	ribosomal large subunit biogenesis	8.46E-11
43170	macromolecule metabolic process	4.35E-10
90304	nucleic acid metabolic process	1.52E-09
34645	cellular macromolecule biosynthetic process	3.13E-09
34622	cellular protein-containing complex assembly	5.85E-09
9059	macromolecule biosynthetic process	6.24E-09
469	cleavage involved in rRNA processing	8.37E-09
90501	RNA phosphodiester bond hydrolysis	3.74E-08
65003	protein-containing complex assembly	5.81E-08
460	maturation of 5.8S rRNA	1.98E-07

466	maturation of 5.8S rRNA from tricistronic rRNA transcript (SSU-rRNA, 5.8S rRNA, LSU-rRNA)	1.98E-07
44281	small molecule metabolic process	2.01E-07
90502	RNA phosphodiester bond hydrolysis, endonucleolytic	2.04E-07
44283	small molecule biosynthetic process	2.88E-07
478	endonucleolytic cleavage involved in rRNA processing	3.45E-07
479	endonucleolytic cleavage of tricistronic rRNA transcript (SSU-rRNA, 5.8S rRNA, LSU-rRNA)	3.45E-07
90305	nucleic acid phosphodiester bond hydrolysis	4.28E-07
43933	protein-containing complex subunit organization	6.63E-07
966	RNA 5'-end processing	2.43E-06
34471	ncRNA 5'-end processing	4.08E-06
8202	steroid metabolic process	4.10E-06
9126	purine nucleoside monophosphate metabolic process	5.18E-06
9127	purine nucleoside monophosphate biosynthetic process	5.18E-06
9167	purine ribonucleoside monophosphate metabolic process	5.18E-06
9168	purine ribonucleoside monophosphate biosynthetic process	5.18E-06
16125	sterol metabolic process	6.26E-06
967	rRNA 5'-end processing	6.82E-06
16126	sterol biosynthetic process	6.98E-06
6694	steroid biosynthetic process	6.98E-06
16129	phytosteroid biosynthetic process	7.96E-06
1902653	secondary alcohol biosynthetic process	7.96E-06
44108	cellular alcohol biosynthetic process	7.96E-06
6696	ergosterol biosynthetic process	7.96E-06
97384	cellular lipid biosynthetic process	7.96E-06
71840	cellular component organization or biogenesis	7.96E-06
9124	nucleoside monophosphate biosynthetic process	8.87E-06

472	endonucleolytic cleavage to generate mature 5'-end of SSU-rRNA from (SSU-rRNA, 5.8S rRNA, LSU-rRNA)	1.86E-05
16128	phytosteroid metabolic process	1.90E-05
44107	cellular alcohol metabolic process	1.90E-05
8204	ergosterol metabolic process	1.90E-05
1902652	secondary alcohol metabolic process	2.20E-05
9123	nucleoside monophosphate metabolic process	3.16E-05
1901617	organic hydroxy compound biosynthetic process	3.53E-05
9156	ribonucleoside monophosphate biosynthetic process	3.73E-05
9161	ribonucleoside monophosphate metabolic process	3.73E-05
96	sulfur amino acid metabolic process	6.87E-05
46165	alcohol biosynthetic process	0.0001
22607	cellular component assembly	0.00014
6082	organic acid metabolic process	0.0002
9069	serine family amino acid metabolic process	0.00032
9113	purine nucleobase biosynthetic process	0.00063
6520	cellular amino acid metabolic process	0.00091
46112	nucleobase biosynthetic process	0.00097
43436	oxoacid metabolic process	0.00143
9112	nucleobase metabolic process	0.00155
6534	cysteine metabolic process	0.00235
1901615	organic hydroxy compound metabolic process	0.0024
71428	rRNA-containing ribonucleoprotein complex export from nucleus	0.00324
19752	carboxylic acid metabolic process	0.00503
35690	cellular response to drug	0.00577
6189	'de novo' IMP biosynthetic process	0.00646
6066	alcohol metabolic process	0.01004
46040	IMP metabolic process	0.01065
46083	adenine metabolic process	0.01065
6188	IMP biosynthetic process	0.01065
51029	rRNA transport	0.01154
6407	rRNA export from nucleus	0.01154
42440	pigment metabolic process	0.01726

6403	RNA localization	0.02063
447	endonucleolytic cleavage in ITS1 to separate SSU-rRNA from 5.8S rRNA and LSU-rRNA from tricistronic rRNA transcript (SSU-rRNA, 5.8S rRNA, LSU-rRNA)	0.02193
46148	pigment biosynthetic process	0.02722
50658	RNA transport	0.02973
51236	establishment of RNA localization	0.02973
1901362	organic cyclic compound biosynthetic process	0.03132
19344	cysteine biosynthetic process	0.03211
3333	amino acid transmembrane transport	0.03511
50657	nucleic acid transport	0.03673
6144	purine nucleobase metabolic process	0.03904
6405	RNA export from nucleus	0.04061
97	sulfur amino acid biosynthetic process	0.04291
51169	nuclear transport	0.04404
6913	nucleocytoplasmic transport	0.04404
6563	L-serine metabolic process	0.04803
51168	nuclear export	0.05021
480	endonucleolytic cleavage in 5'-ETS of tricistronic rRNA transcript (SSU-rRNA, 5.8S rRNA, LSU-rRNA)	0.05221
6555	methionine metabolic process	0.06754
71166	ribonucleoprotein complex localization	0.07236
71426	ribonucleoprotein complex export from nucleus	0.07236
55086	nucleobase-containing small molecule metabolic process	0.07341
6611	protein export from nucleus	0.08889
