# Supplementary material for: Tunicamycin Potentiates Antifungal Drug Tolerance via Aneuploidy in Candida albicans
Source: mBio. 2021 Aug 31;12(4):e02272-21. doi: 10.1128/mBio.02272-21 (PMC8406271; doi:10.1128/mBio.02272-21)
Supplement: TABLE S1 [file mbio.02272-21-st001.docx]

Table S1. Strains used in this study

| Strain | Genotype | Parent | Source |
| --- | --- | --- | --- |
| SC5314 | Wild type |  | (1) |
| YCA641 | cmp1::FRT/cmp1::NAT1 flp | SC5314 | (2) |
| YCA623 | cnb1::FRT/cnb::NAT1 flp | SC5314 | (2) |
| YCA736 | crz1::FRT/crz1::NAT1 flp | SC5314 | (2) |
| YCA892 | mkk2::FRT/mkk2::NAT1 flp | SC5314 | This study |
| YCA1127 | mkc1::FRT/mkc1::NAT1 flp | SC5314 | This study |
| YCA1125 | swi4::FRT/swi4::NAT1 flp | SC5314 | This study |
| YCA1121 | swi6::FRT/swi6::NAT1 flp | SC5314 | This study |
| YCA1109 | RLM1/rlm1::NAT1 flp | SC5314 | This study |
| YCA1126 | rlm1::FRT/rlm1::NAT1 flp | SC5314 | This study |
| YCA693 | ALG7/alg7::NAT1 flp | SC5314 | This study |
| YCA40 | RTA2/rta2::NAT1 flp | SC5314 | This study |
| YCA424 | rta2::FRT/rta2::NAT1 flp | SC5314 | This study |
| YCA251 | RTA3/rta3::NAT1 flp | SC5314 | This study |
| YCA487 | rta3::FRT/rta3::NAT1 flp | SC5314 | This study |
| YCA245 | RNR1/rnr1::NAT1 flp | SC5314 | (3) |
| YCA259 | RNR21/rnr21::NAT1 flp | SC5314 | (3) |

**References**

1. Noble SM, Johnson AD. 2005. Strains and strategies for large-scale gene deletion studies of the diploid human fungal pathogen Candida albicans. Eukaryot Cell 4:298-309.

2. Xu Y, Lu H, Zhu S, Li WQ, Jiang YY, Berman J, Yang F. 2021. Multifactorial Mechanisms of Tolerance to Ketoconazole in Candida albicans. Microbiol Spectr doi:10.1128/Spectrum.00321-21:e0032121.

3. Yang F, Teoh F, Tan ASM, Cao Y, Pavelka N, Berman J. 2019. Aneuploidy Enables Cross-Adaptation to Unrelated Drugs. Mol Biol Evol 36:1768-1782.
