# Supplementary material for: Tunicamycin Potentiates Antifungal Drug Tolerance via Aneuploidy in Candida albicans
Source: mBio. 2021 Aug 31;12(4):e02272-21. doi: 10.1128/mBio.02272-21 (PMC8406271; doi:10.1128/mBio.02272-21)
Supplement: TABLE S2 [file mbio.02272-21-st002.docx]

**Table S2. Sequences of primers used in this study**

| Primer name | Primer sequence (5' to 3') |
| --- | --- |
| Gene deletions | |
| BP1639 | CAGATCGTACAATAAAGCTTTGAAG |
| BP1640 | TGCGTCTATTTATGTAGGATGAAAG |
| CaMKK2-US-F | GTCGGAACGTTGTGCATTAC |
| NAT1-CaMKK2-US-R | GTATAGGAACTTCCTCGAGGGATGGCAATCAACGTAAACTAATCTC |
| NAT1-CaMKK2-DS-F | AGATCCACTAGTTCTAGAGCGGGTTAAAGTGGTGTGGGAATTAAATG |
| CaMKK2-DS-R | CGATAACACAGATGGAATAGAATTAGG |
| CaMKC2-US-F | CAAACATTCGTATTAAGCCG |
| NAT1-CaMKC1-US-R | GTATAGGAACTTCCTCGAGGGCTGATTTTGTGTATGCTCGTG |
| NAT1-CaMKC1-DS-F | AGATCCACTAGTTCTAGAGCGGGGGGTATGTTACTCTCGAATTAG |
| CaMKC1-DS-R | CTCCATTATCGAGCGACC |
| CaSWI4-US-F | GTTCCAAAATCCCTAACACC |
| NAT1-CaSWI4-US-R | GTATAGGAACTTCCTCGAGGGGGGTGATACAAACAATCGATG |
| NAT1-CaSWI4-DS-F | AGATCCACTAGTTCTAGAGCGGCGGAACAAAATTGAAAGAGG |
| CaSWI4-DS-R | GGGACTTCTTGGTTAATAATACG |
| CaSWI6-US-F | CTTCATTACTTGCTATTCCACC |
| NAT1-CaSWI6-US-R | GTATAGGAACTTCCTCGAGGGCTTGAGCCAGCGTTTATAATG |
| NAT1-CaSWI6-DS-F | AGATCCACTAGTTCTAGAGCGGGGCTCTCTATACCAAACCCAG |
| CaSWI6-DS-R | GAACAGAAAATCAACTGGAGC |
| CaRLM1-US-F | GAGGAAAGAACGGAAATAAGC |
| NAT1-CaRLM1-US-R | GTATAGGAACTTCCTCGAGGGGTAGATGACGTTAGAGGGGTG |
| NAT1-CaRLM1-DS-F | AGATCCACTAGTTCTAGAGCGGGCATGATTTTCACAAGTGAATAG |
| CaRLM1-DS-R | GCAAATGAAACCAAAGAGTTC |
| CaALG7-US-F | CCAGTGAAAATCCACGTG |
| NAT1-CaALG7-US-R | GTATAGGAACTTCCTCGAGGGGCTGAACCTGTCTAAGGAACC |
| NAT1-CaALG7-DS-F | AGATCCACTAGTTCTAGAGCGGGCAAATATAGAAAGGAGTCGTG |
| CaALG7-DS-R | GCATCTCTACCTGGTGGC |
| CaRTA2-US-F | GCTTATTGTAGAGATCTTCGTTATGG |
| NAT1-CaRTA2-US-R | GTATAGGAACTTCCTCGAGGGAGTTGAAGGGTGGGAATGAAC |
| NAT1-CaRTA2-DS-F | AGATCCACTAGTTCTAGAGCGGGTTATGCATTAGATTAAGTATTGTTCTAGATAG |
| CaRTA2-DS-R | GGATGGGTTGAGCTTTACG |
| CaRTA3-US-F | GATCCACACGGAACTCG |
| NAT1-CaRTA3-US-R | GTATAGGAACTTCCTCGAGGGGATTTTGGTTAACCGGGTG |
| NAT1-CaRTA3-DS-F | AGATCCACTAGTTCTAGAGCGGCTAAGGGGTGGTATGATTCC |
| CaRTA3-DS-R | GCCTAGCTTAACATAGCACAGC |
| Diagnostic PCR for deletions | |
| CaMKK2-USD-F | GTCGTCAGTCGTTTCAGATGTAG |
| CaMKK2-DSD-R | GAATCAGCCAGTTCCAGATG |
| CaMKC1-USD-F | CTGCGTAATATTGCCCTACC |
| CaMKC1-DSD-R | GAAGACAACCAGCTCCACAC |
| CaSWI4-USD-F | GAAACTCACATAATTCGATCATC |
| CaSWI4-DSD-R | CCTCCTTCAGCAGTGACC |
| CaSWI6-USD-F | GGTTTCGGAAAACGTAAGG |
| CaSWI6-DSD-R | GGTGACCCGACTGATTATTG |
| CaRLM1-USD-F | GGTAATCCTACCGGCTGTAG |
| CaRLM1-DSD-R | GCTATAGCCCTAAGTCACGC |
| CaALG7-USD-F | GCCGGGTATATCAAGGAGTC |
| CaALG7-DSD-R | GGCTTGGATTTATCGACG |
| CaRTA2-USD-F | GGACCAGCCGCATAAAC |
| CaRTA2-DSD-R | CTGAATAACACGCTTTGATTTG |
| CaRTA3-USD-F | GCTTGGTGAAGGCTATGG |
| CaRTA3-DSD-R | CGTTAATAATTTCCTTCGGTCTC |
